# Proteomics profiling identifies extracellular vesicles’ cargo associated with tumour cell induced platelet aggregation

**DOI:** 10.1186/s12885-022-10068-7

**Published:** 2022-09-29

**Authors:** Niamh McNamee, Laura Rodriguez de la Fuente, Maria Jose Santos-Martinez, Lorraine O’Driscoll

**Affiliations:** 1 School of Pharmacy and Pharmaceutical Sciences, Trinity College Dublin, Dublin, Ireland; 2 Trinity Biomedical Sciences Institute, Trinity College Dublin, Dublin, Ireland; 3 Trinity St. James’s Cancer Institute, Trinity College Dublin, Dublin, Ireland; 4 School of Medicine, Trinity College Dublin, Dublin, Ireland

**Keywords:** Triple-negative breast cancer, Extracellular vesicles, Platelets, Aggregation, Thrombosis

## Abstract

**Background:**

Cancer patients have an increased risk of developing venous thromboembolism, with up to 30% dying within a month of their development. Some cancer cells are known to induce platelet aggregation, and this interaction is understood to contribute to thrombosis and haematogenous metastasis. Many researchers have reported on extracellular vesicles (EVs) released from platelets. However, less is known about how cancer cells’ EVs may affect platelet function. Here EVs released by triple-negative breast cancer (TNBC) cell line variants were extensively investigated in this regard.

**Methods:**

EVs were separated from conditioned media of TNBC Hs578T and Hs578Ts(i)_8_ cells using filtration and ultracentrifugation and were characterised by nanoparticle tracking analysis, immunoblots, and transmission electron microscopy. Blood samples from consenting donors were procured, and their platelets collected by differential centrifugation. Light transmission aggregometry and optical microscopy evaluated the potential interaction of TNBC cells and their EVs with platelets. Global proteomic analysis was performed on the EVs, by in-solution digestion and mass spectrometry. Data analysis included the use of Perseus, FunRich, and Vesiclepedia. Immunoblotting was used as a secondary method to investigate some key EV cargo proteins identified by the global proteomics approach.

**Results:**

Both TNBC cell variants induced platelet aggregation. Increasing cell numbers significantly reduced the time taken for platelet aggregation to occur. EVs released by the cells also resulted in platelet aggregation. The time to induce platelet aggregation was EV dose-dependent. Proteomics profiling and immunoblotting of the EVs’ cargo identified candidate proteins (including uPAR and PDGFRβ) that may be involved during this process.

**Conclusions:**

TNBC cells induce platelet aggregation. Furthermore, the cell-free EVs induced this undesirable effect. A number of EV cargo proteins were identified that may be relevant as therapeutic targets.

**Supplementary Information:**

The online version contains supplementary material available at 10.1186/s12885-022-10068-7.

## Introduction

Cancer patients account for 15-30% of all patients diagnosed with venous thromboembolism (VTE). Tumour cell-induced platelet aggregation is an important determinant of cancer-associated thrombosis (CAT), which has risk factors including age, gender, type and stage of cancer, and increased expression of tissue factor (TF) [1]. It is well established that tumours express TF which, in addition to being the main activator of the extrinsic pathway of the coagulation cascade, can also promote tumour growth and metastasis [2]. Cancer cells can also contribute to the hypercoagulable state by releasing pro-coagulating factors such as heparanase, and through the shedding of extracellular vesicles (EVs) that carry high levels of TF [3].

Extracellular vesicles (EVs) are lipid-bilayer-enclosed nanoparticles released by most, if not all, cells. Their cargo may include proteins, RNAs, DNA, and lipids, reflecting the content of their cells of origin. Ourselves and other have shown that EVs’ bioactive cargo is instrumental in their role in cell-to-cell communication, mediating a broad range of physiological and pathological activities [4–8]. EVs have traditionally been categorised based on sub-cellular origin and size as exosomes and microvesicles/ectosomes [9, 10]. EVs are detectable in a broad range of biofluids including cultured cells’ conditioned medium (CM) and blood [11]. These important sources of EVs are commonly studied, but we must accept that once in a biofluid we cannot claim the EVs’ exact origin and exit route(s) from their donor cells. Thus, the generally accepted collectively term for exosomes and microvesicles is EVs [12]. However, some studies still refer to EVs from platelets as microparticles.

A decade ago, we showed that platelets increase cancer cells’ survival, proliferation, and resistance to anti-cancer drugs [13]. In addition, the TF present on EVs, mainly platelet EVs, and thrombin generation have been identified as two potential activators of platelet aggregation in cancer patients. Specifically, a study in gastric cancer reported that platelet microparticles are increased in numbers in plasma from stage IV patients compared to earlier stage disease [14]. Another study reported TF activity to be significantly higher in plasma samples -that contained platelets and microparticles- from cancer patients compared to healthy individuals [15]. Furthermore, it has been demonstrated that microparticles from plasma of patients with early-stage prostate cancer had higher TF-specific pro-coagulant activity compared with the plasma of healthy controls [16]. Gomes et al. [17] demonstrated that EVs derived from MDA-MB-231 and MCF-7 cell lines induce platelet aggregation by TF-dependent and undefined independent mechanisms. Therefore, although TF has been heavily implicated in CAT, other cancer cell-derived proteins may also be involved in platelet aggregation.

Previously using a TNBC cell line (Hs578T) and its aggressive clonal variant (Hs578Ts(i)_8_) as model systems, we investigated the potential of EVs to influence the phenotype of “recipient”/secondary cancer and normal endothelial cells. The results indicated that the EVs from both, but particularly from the more aggressive cells transmitted the same traits to all secondary cell lines analysed (i.e., increasing their proliferation, migration, and inducing neovascularisation/angiogenesis), reflecting the innate phenotypes of the cells from which the EVs are released. However, the potential contribution of TNBC to platelet aggregation has not previously been investigated. Thus, here we investigated and established that the TNBC cell line variants, Hs578T and Hs578Ts(i)_8_ and their derived EVs induce platelet aggregation. Afterwards, to better understand the process involved, the proteome cargo of the EVs was analysed to potentially identify proteins -additional to TF- that could be involved in this process.

## Methods

### Cell culture and collection of extracellular vesicles

Hs578T was obtained from the American Tissue Culture Collected (ATCC) and its isogenic subclone, Hs578Ts(i)_8_ was isolated from Hs578T and gifted to us by Dr. Susan McDonnell, UCD. The cells were cultured at 37 °C/5% CO_2_ in DMEM (Sigma, Cat. #: D5671), 10% FBS, 2 mM L-Glutamine and 10 μg/ml of insulin (Sigma; Cat. #:I9278). EVs were collected from their cell conditioned medium by differential ultracentrifugation as previously described [18].

### Immunoblotting

Immunoblotting was performed as previously described [19], using 10 μg of EVs or cell lysates. Primary anti-human antibodies used were to: PDGFRβ (1 μg/mL) (R&Dsystems, Cat. #:MAB1263), Glypican-1 (1 in 1000) (Abcam, Cat. #:ab199343), uPAR (1 μg/mL) (R&Dsystems, Cat. #:MAB807), CD97 (1 μg/mL) (R&Dsystems, Cat. #: MAB2529) MCAM/MUC18 (1 μg/mL) (R&D Systems, Cat. #:MAB932), Cyr61 (1 μg/mL) (R&D Systems, Cat. #:MAB4055). Secondary antibodies were: anti-rabbit (1:1000; Cell Signaling Technology, Cat. #: 7074) or anti-mouse (1:1000, Cell Signaling Technology; Cat. #: 7076). Hs578Ts(i)_8_ cell lysate was included in all gels as the control and densitometric analysis was performed using Fiji software.

### Nanoparticle tracking analysis

EV size and concentration was measured using NanoSight NS500 as we previously described [19]. EVs were captured at 30 frame/s speed and six 60 second videos were recorded. Samples were diluted (1 in 100) in 1 mL of 0.22 μm-filtered PBS. The NTA system is equipped with a 405 nm laser, and NanoSight 3.2 software was used.

### Transmission electron microscopy (TEM)

EVs were prepared for TEM analysis as we previously described [20]. Briefly, 20 μl of EVs were placed on carbon-coated grids (Ted-Pella B 300 M, Mason Technology Ltd., Cat. #: 01813-F) and allowed settle for 10 minutes. Samples were then fixed using 4% glutaraldehyde and contrasted with 2% phosphotungstic acid. EVs were imaged at 100 kV using a JOEL JEM-2100 TEM (JOEL USA Inc. Peabody, MA, USA).

### Blood collection and preparation of platelets

The research included here was performed in accordance with the Declaration of Helsinki and approval for this study was obtained from the School of Pharmacy and Pharmaceutical Sciences’ Level 1 Research Ethics Committee (Trinity College Dublin; Reference No. of Study: 2015-06-01 MS). Following informed consent, blood was withdrawn from healthy volunteers who had not taken any medication known to interfere with platelets function for at least 2 weeks prior to the study. Washed platelets (WP) were prepared by differential centrifugation as previously described [21]. Briefly, blood was drawn, mixed with 3.15% sodium citrate, and centrifuged at 250 g for 20 mins. The resulting platelet rich plasma (PRP) was centrifuged, in the presence of prostacyclin, at 900 g for 10 mins and the resultant pellet resuspended in Tyrode’s buffer (Sigma, Cat. #: T2937). Platelets were counted using a Beckman Coulter Z1 series Coulter Counter (Labplan, Ireland) and adjusted to a final concentration of 2.5 × 10^8^ platelets/mL [22].

### Preparation of TNBC cells for platelet aggregation experiments

Hs578T and Hs578Ts(i)_8_ cell line variants were prepared when their cell confluency reached 80-90%. Cells were detached with DPBS/EDTA (7 mM), collected, and centrifuged at 300 g for 5 mins. The resultant cell pellet was washed and resuspended in Tyrode’s solution. The cells were then counted using a Beckman Coulter Z1 series Coulter Counter (Labplan, Ireland) [23, 24].

### Light transmission aggregometry (LTA)

Platelet aggregation was measured using an eight-channel Platelet Aggregation Profiler Model PAP-8E from (Biodata Corporation, Ireland). Briefly, WP (2.5 × 10^8^ platelets/mL) were placed under stirring for 2 min at 37 °C. TNBC cells or their corresponding EVs were then added to the cuvettes at a range of concentrations and platelet response monitored by the software for 30 mins. For controls, instead of adding cells or EVs, Tyrode’s solution and PBS were used, respectively. At the beginning of each set of experiments, 2 μg/mL of collagen was added to the WP to confirm the ability of the donors’ platelets to aggregate [23–25].

### Optical microscopy

Following aggregometer experiments with TNBC cell line variants or their corresponding EVs, platelets suspensions were fixed with 2% paraformaldehyde (Sigma, Cat. #: P6148) and incubated under stirring conditions at 37C for 30 minutes. Samples were then mounted on a microscope slide, using a Cytospin 4 (Thermo Shandon, Fisher Scientific, Ireland) and micrographs taken at a magnification of 10X using an Olympus CKX41 microscope (Mason Technology Ltd., Ireland) [23–25].

### In-solution digestion of EVs for mass spectrometry (MS) analysis

EV protein (50 μg) was prepared and analysed by MS analysis. Preparation method was based on a previously described method [26], further optimised by us as follows: Fresh 100 mM DL-dithiothreitol (Sigma, Cat. #:D0632) was prepared with HPLC-grade water (Sigma-Aldrich, Cat. #:900682) and added to each sample to give a final concentration of 5 mM. Samples were vortexed and heated at 60 °C for 5 mins to remove the disulphide bonds. For alkylation of the samples, 200 mM of iodoacetamide (Sigma, Cat. #: I1149) was added to the samples to give a final concentration of 10 mM, vortexed and incubated in the dark for 30 mins. 50 μl of reduced and alkylated sample was added to one vial of trypsin singles (Sigma, Cat. #:T7575), vortexed and allowed to digest overnight at 37 °C on a thermomixer at 350 rpm. To stop the digest, acetic acid (Sigma, Cat. #:338826) was added to the sample (i.e., a volume of 1% of the sample volume). Digested proteins were bound and desalted using C18 ZipTips (Merck Millipore, Cat. #:ZTC18S096), washing the samples with 0.1% Trifluoroacetic acid (TFA) (Sigma, Cat. #:T0699). Once washed, the samples were eluted from the ZipTip and dispensed in 12 μl of 50% acetonitrile (Sigma, Cat. #:494445) in 0.1% TFA. The samples were then dried using a Speedvac vacuum concentrator and resuspended in 10 μl of MS buffer (2.5% acetonitrile, 0.5% acetic acid).

### Mass spectrometry

Experiments were performed based on a previously described method [27]. Peptide fractions were analysed on a quadrupole Orbitrap (Q-Exactive, Thermo Scientific) mass spectrometer equipped with a reversed-phase NanoLC UltiMate 3000 HPLC system (Dionex LC Packings, now Thermo Scientific). Peptide samples were loaded onto C18 reversed phase columns (10 cm length, 75 μm inner diameter) and eluted with a linear gradient from 1 to 27% buffer B containing 0.5% AA 97% ACN in 60 min at a flow rate of 250 nL/min. The injection volume was 5 μl. The mass spectrometer was operated in data dependent mode, automatically switching between MS and MS2 acquisition. Survey full scan MS spectra (m/z 300–1200) were acquired in the Orbitrap with a resolution of 70,000. MS2 spectra had a resolution of 17,500. The twelve most intense ions were sequentially isolated and fragmented by higher-energy C-trap dissociation.

### Protein identification

Raw data from the Orbitrap Q-Exactive was processed using MaxQuant version 1.6.3.4 [28, 29], incorporating the Andromeda search engine [30]. To identify peptides and proteins, MS/MS spectra were matched to the Uniprot *homo sapiens* database (2018_12) containing 73,928 entries. All searches were performed with tryptic specificity allowing two missed cleavages. The database searches were performed with carbamidomethyl I as fixed modification and acetylation (protein N terminus) and oxidation (M) as variable modifications. Mass spectra were searched using the default setting of MaxQuant namely a false discovery rate of 1% on the peptide and protein level. For the generation of label free quantitative (LFQ) ion intensities for protein profiles, signals of corresponding peptides in different nano-HPLC MS/MS runs were matched by MaxQuant in a maximum time window of 1 min [31].

### Analysis of data using Perseus software

The data generated using MaxQuant was uploaded into Perseus software v1.6.15.0 to identify EVs proteins and perform statistical analysis and visualisation. Data were filtered from contaminants such as proteins only identified by site modifications which may not be accurate and were transformed into log scale. Rows were categorised into Hs578T EVs or Hs578Ts(i)_8_ EVs. To increase the confidence of the data set, proteins that were identified in at least three out of the six EV samples (i.e., three biological repeats for each variants’-derived EVs) were analysed further, filtering for the valid values. A two-sample t-test was performed to compare the cell line variant-derived EVs with a threshold of 0.05, identifying proteins that are significantly different between the two variants’ EVs. Hierarchical clustering was performed by generating z-scores of normalised LFQ intensities. Values were then clustered using the Euclidean distance method and a heatmap was produced to visualise the intensity levels of the proteins among the two populations of EVs, with green representing low intensity and red representing high intensity Principal component analysis was performed using Perseus’ built-in tool, using the data before it was filtered and transformed (see Supplemental Material for the loading plot of PCA analysis). FunRich, a functional enrichment software tool (v3.1.3), was used to analyse the cellular component and biological pathways associated with proteins identified in the EVs [32]. Vesiclepedia database was downloaded (12th July 2022) and compared with the proteins identified in the TNBC EVs using the FunRich software.

### Statistical analysis

All results presented were obtained from at least three independent experiments. Where more repeats were performed, that is noted in the corresponding figure legends. Paired t-test analysis was performed using GraphPad Prism version 9.1.9. ANOVA was used to compare more than two groups. Data are expressed as means ± standard error of the mean (SEM). Statistical significance was considered when * *p <* 0.05; ** *p <* 0.01; *** *p <* 0.001.

## Results

### Characterisation of EVs released from TNBC cell line variants

**Fig. 1:**
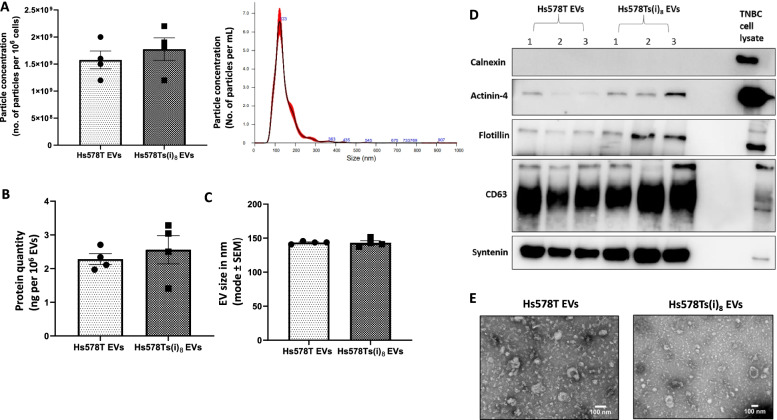
Extracellular vesicles isolated from the Hs578T and Hs578Ts(i)_8_ cell line variants were characterised by NTA (**A**, **B**), with a representative distribution profile (of Hs578T) EVs shown. Bradford assay was used to quantify protein (**C**) normalised to 10^6^ EVs. A representative immunoblot with 1-3 representing 3 independent EVs collections (**D**), and TEM analysis (**E**). TEM images are taken at 20,000X magnification with scale bar of 100 nm. Graphs represent mean ± SEM of *n* = 4 independent experiments

EVs separated from isogenic TNBC cell line variants Hs578T and Hs578Ts(i)_8_ after five days of culturing in EVs-dFBS medium were characterised by NTA (Fig. 1 (A, B)) to estimate the yield (i.e., quantity of EVs released/10^6^ cells) and size (modal) of the EVs released by both cell line variants. A representative NTA profile is also shown (Fig. 1 (A)). Hs578T EVs had a size range of 140.8-145.8 nm and Hs578Ts(i)_8_ EVs ranged between 137.3-151.6 nm. Bradford assay measured the protein quantities of the isolates (Fig. 1 (C)). No significant difference was observed in protein quantities between the two variants’ isolates. Immunoblot analysis supported the presence of EVs, based on the detection of common EV-positive markers including flotillin, syntenin and CD63 (Fig. 1 (D); see Supplemental Fig. 1 (D) for full-length blots). Actinin-4 was also detected, suggesting that the pool of EVs did not only contain small EVs, a characterisation step recommended by MISEV2018 guidelines. Calnexin was used as a purity control and EV-negative marker. TEM analysis also confirmed the successful separation of EVs of various sizes (Fig. 1 (E)).

### Tumour cell-induced platelet aggregation (TCIPA) by TNBC cells line variants

**Fig. 2:**
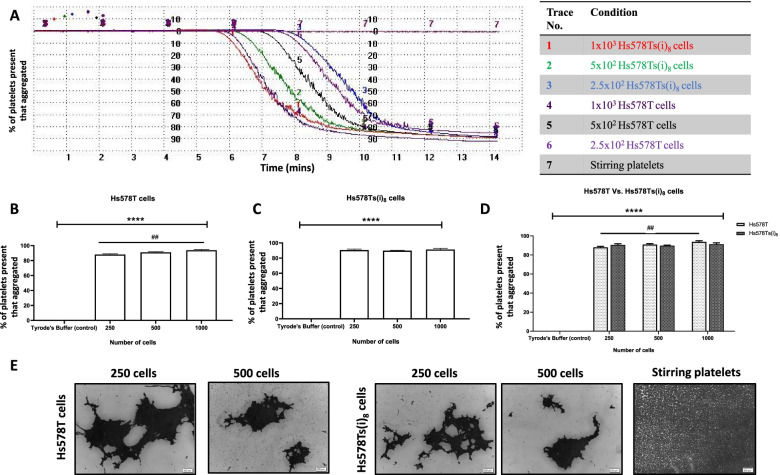
TNBC cells induced platelet aggregation. Representative traces from light transmission aggregometry showing platelet aggregation induced by TNBC cells at different numbers (**A**). Statistical analysis of the effect of TNBC cells on platelet function (**B**, **C**). Data generated with Hs578T and Hs578Ts(i)_8_ cells was co-analysed together to determine if there were significant differences between the two cell line variants (**C**). Percentage of platelets available that aggregation is graphed. Graphs represent mean ± SEM from *n* = 4 independent experiments. One-way ANOVA was performed for (**B**) and (**C**) and a two-way ANOVA performed for (**D**). ****p < 0.0001 vs control (platelets stirred in the absence of cancer cells) and ##*p* < 0.01 (i.e., this significant result is from the ANOVA tests that were performed, with an ## used instead of ** to highlight the significant difference between 1000 and 250 cells. Representative micrographs from optical microscopy demonstrating the presence of big aggregates in the presence of both cell lines and the absence of aggregates in stirring platelets. Scale bar 200 μm (**E**). For complete two-way ANOVA results see Supplemental Material

After incubating washed platelets (WP) of the donors for two minutes, TNBC cells were added, and their effect on platelet function monitored by the PAP-8 software until a plateau was reached (30 minutes) (Fig. 2 (A)). Both cell line variants induced platelet aggregation at all concentrations tested (Fig. 2 (B, C)), and these effects were confirmed by optical microscopy (Fig. 2 (E)). When the two cell line variants were compared, there was no significant differences between their TCIPA-inducing effects (Fig. 2 (D)).

### Platelet aggregation induced by TNBC cell line variant EVs

**Fig. 3:**
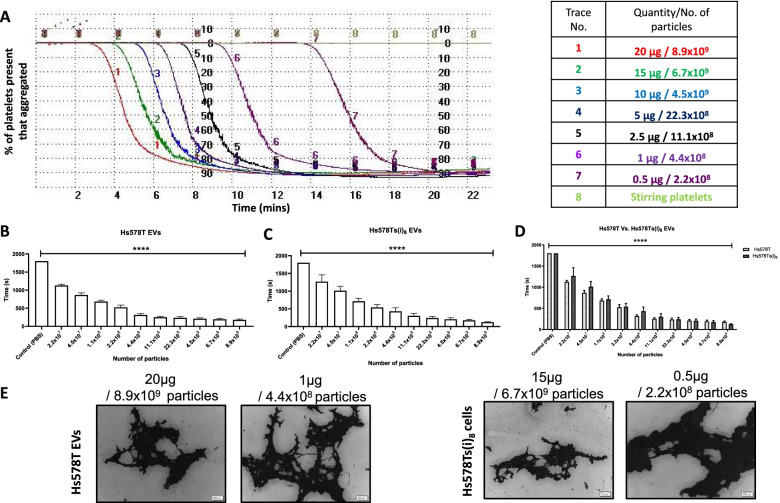
EVs from TNBC cells induced platelet aggregation. Representative traces from light transmission aggregometry showing platelet aggregation induced by EVs at various concentrations (**A**) Statistical analysis of the effect of EVs from TNBC cells on platelet function. Data generated with Hs578T EVs and Hs578Ts(i)_8_ EVs was co-analysed together to determine if there were significant differences between the effect of EVs isolated from the conditioned media from the cell line variants tested. Graphs represent mean ± SEM from n = 4 independent experiments. One-way ANOVA was carried out for (**B**) and (**C**) and a two-way ANOVA for (**D**). ****p < 0.0001 vs control (platelets stirred in the absence of EVs). Representative micrographs from optical microscopy demonstrating the presence of platelet aggregates in the presence of EVs at different concentrations. Scale bar 200 μm (**E**). For full two-way ANOVA results see Supplemental Material

After confirming that the TNBC cell line variants induce platelet aggregation, EVs from each cell line were then tested (Fig. 3 (A)). EVs from both cell line variants significantly induced platelet aggregation (Fig. 3 (B, C)), with a delayed time for the onset of platelet aggregation (increase in the lag phase) observed in an EVs concentration-dependent manner. The effect was also confirmed by optical microscopy (Fig. 3 (E)). As with their cells of origin, there were no significant differences when the effect of both EVs sources on platelet aggregation were compared (Fig. 3 (D)). This suggests that potentially EVs from all TNBCs, whether they are more or less aggressive, may contribute to platelet aggregation and thrombosis.

### Global proteomic profiling of TNBC cell line variant EVs

**Fig. 4:**
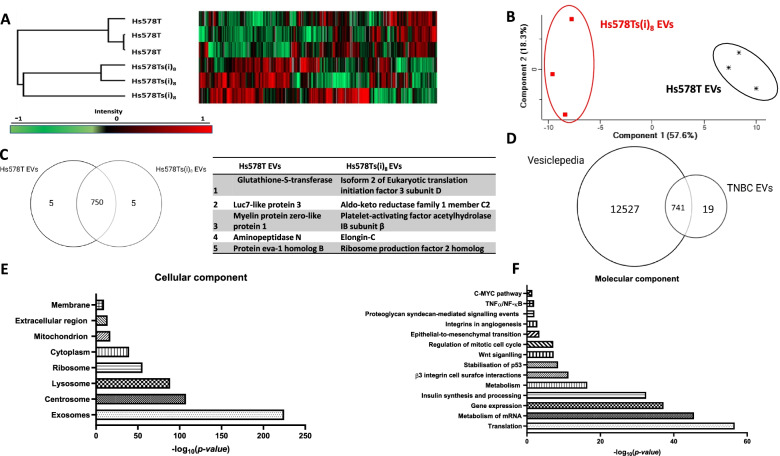
Perseus software was used to perform statistical analysis on the MS data. **A** Heatmap of unsupervised clustering where normalisation was based on z scores and the data for each row was normalised by the mean value calculated for the same row. Green represents low protein intensity and red representing high protein intensity. **B** Principal component analysis was performed. **C** Venn diagram was created to represent the total proteins identified in the two cell variants’-derived EVs and the *n* = 5 proteins that are specific to each. **D** The total vesiclepedia database (as of 12 July 2022) was compared with the proteins identified. Cellular (**E**) and biological (**F**) components significantly associated with the proteins identified in TNBC EVs. All analysis was performed using *n* = 3 biological repeats

Considering the results from our earlier studies [18, 33] and from this study, evidence suggests that EVs from TNBC seems to be involved in many problems associated with cancer including cancer cell proliferation, migration, inducing neovascularisation/angiogenesis, drug-resistance, and platelet aggregation. Thus, to identify specific factors that may contribute to these issues, proteomic profiling was performed on EVs from both the Hs578T and Hs578Ts(i)_8_ cells. Unsupervised hierarchical clustering showed that the two EVs populations have distinct patterns of protein levels, clearly demonstrated at the top and bottom of the heatmap (Fig. 4 (A)). Principal component analysis (PCA) demonstrates that the two EVs populations cluster separately from each other, illustrating that they are different from each other in terms of protein cargo (Fig. 4 (B)). A Venn diagram illustrates that the EVs contained 760 proteins in total between the two variants, with five being exclusive to each (Fig. 4 (C)). The proteins identified in the TNBC EVs were compared with the total number of proteins reported on the Vesiclepedia database. Nineteen proteins present (see Supplemental Materials for list of previously unidentified proteins) in the TNBC EVs were not previously documented to exist in EVs (Fig. 4 (D)). Gene enrichment analysis identified that the following cellular components are associated with the proteins identified in the TNBC EVs (Fig. 4 (E)): exosomes, centrosomes, lysosomes, ribosomes, cytoplasm, mitochondrion, extracellular region, and membrane. Biological component analysis revealed cellular functions that are associated with the proteins identified (Fig. 4 (F)).

### Identification of proteins carried by EVs that may be involved in TCIPA

**Fig. 5:**
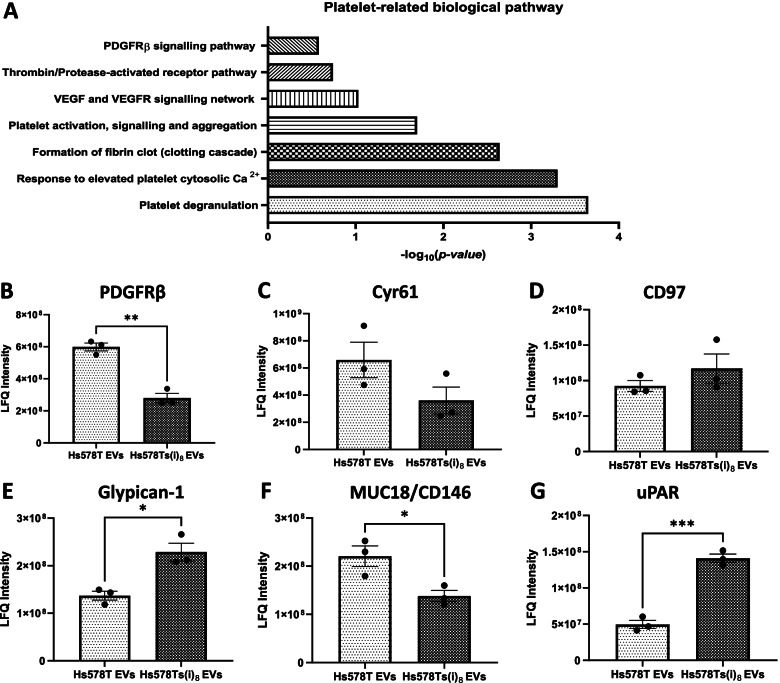
Functional analysis was performed using FunRich. **A** Identified homeostasis-related pathways that were significantly associated with the TNBC EVs. Label-free quantification (LFQ) are graphed for (**B**) PDGFRβ, (**C**) Cyr61, (**D**) CD97, (**E**) Glypican-1, (**F**) MUC18/CD146, and (**G**) uPAR. LFQ intensities are summed peptide intensities which have a normalisation factor applied to enable comparison across all samples. Graphs represent mean ± SEM and are *n = 3* independent experiments. Student’s t-test was used as statistical test. *P < 0.05, **P < 0.01, ***P < 0.001

Functional analysis of the proteomics data showed that the EVs carry proteins that are involved in haemostasis and thrombosis, as well as cancer progression, and thus may play a role during TCIPA (Fig. 5 (A)) as well as others of the adverse effects. Key proteins were selected from the proteomic data following a review of the literature on the topic of platelet aggregation and activity. These proteins included platelet-derived growth factor receptor β (PDGFRβ) which, incidentally, was at significantly (*p* = 0.001) higher amounts in the Hs578T EVs compared to Hs578Ts(i)_8_ EVs (Fig. 5 (B)). Cyr61 was detected in both EVs populations, with a higher quantity in the Hs578T EVs compared to the Hs578Ts(i)_8_ EVs (Fig. 5 (C)). Conversely, CD97 was at a slightly greater quantity in the Hs578Ts(i)_8_ EVs compared to the Hs578T EVs (Fig. 5 (D)). Glypican-1 was at a significantly (*p* = 0.012) higher quantity in the Hs578Ts(i)_8_ EVs compared to the Hs578T EVs (Fig. 5 (E)). Cell surface glycoprotein MUC18 (MUC18, also known as CD146) was detected in both TNBC EVs, with significantly (*p* = 0.028) higher abundance in Hs578T EVs (Fig. 5 (F)). Urokinase-type plasminogen activator receptor (uPAR) was at significantly higher amounts in Hs578Ts(i)_8_ EVs compared to Hs578T EVs (Fig. 5 (G)).

### Validation of platelet-related proteins in TNBC cell line variant

**Fig. 6:**
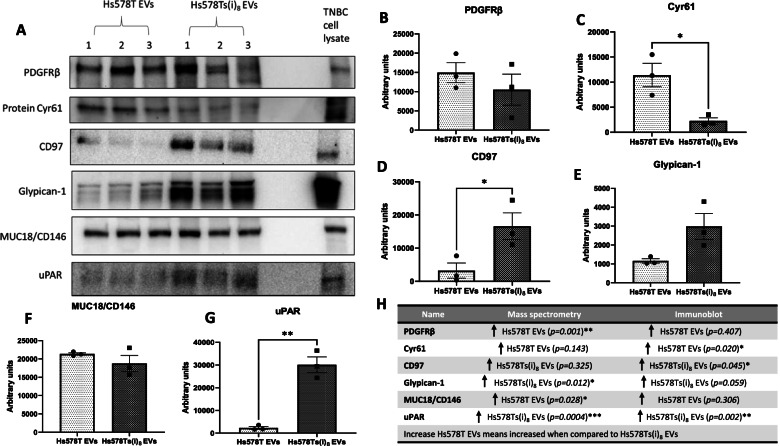
Immunoblot image are shown (**A**), along with densitometric analysis of (**B**) PDGFRβ, (**C**) Cyr61, (**D**) CD97, (**E**) Glypican-1, (**F**) MUC18/CD146 and (**G**) uPAR. (**H**) A summary of proteins detected by mass spectrometry and immunoblot. Graphs represent mean ± SEM of *n =* 3 independent biological experiments, shown as 1,2,3. Unpaired t-test was used as statistical test. *P < 0.05, **P < 0.01, ***P < 0.001

Validation of the mass spectrometry results was attempted by immunoblot (Fig. 6 (A); see supplemental material for full-length blots), which confirmed the presence of all six proteins chosen. Using this method, PDGFRβ was confirmed as at a higher amount in the Hs578T EVs, although not significantly (Fig. 6 (B)). Cyr61 was also found be at a higher amount in the Hs578T EVs, this time significantly (Fig. 6 (C)). CD97 was almost undetected in Hs578T EVs using immunoblots and was at significantly higher amounts in the Hs578Ts(i)_8_ EVs (Fig. 6 (D)). Greater (although not significantly) quantities of glypican-1 were detected with the Hs578Ts(i)_8_ EVs (Fig. 6 (E)). MUC18/CD146 showed no significant difference between the two EVs populations (Fig. 6 (F)), while a significantly great amount of uPAR was found with Hs578Ts(i)_8_ EVs compared to Hs578T EVs (Fig. 6 (G)). A summary of all protein levels detected by mass spectrometry and immunoblot followed by densitometry is shown (Fig. 6 (H)).

## Discussion

Tumour cell-induced platelet aggregation (TCIPA) is a well-established phenomenon in which cancer cells interact with platelets, leading to their aggregation. This exploitation of platelets allows cancer cells to survive in the vasculature, evade cell death and metastasise to distant secondary organs [34]. As ourselves and others have shown, platelets provide a platform for tumour cells to metastasise and are involved in multiple processes such as vasculature remodelling and survival in the bloodstream [13] (and reviewed [35]). In breast cancer, multiple mechanisms of cancer cell-induced aggregation have been proposed. For example, MCF-7 cells have been shown to induce platelet aggregation via the GPIb-IX, ADP, GPIIb/IIIa pathways [36]. Other mechanisms have also been studied in breast cancer cell lines [37, 38]. These biological events caused by the interaction of the cancer cells with platelets presents clinically as thrombosis and is a leading cause of cancer-related deaths. Therefore, our investigation involved determining if EVs (not just the whole cells themselves) released from Hs578T cells and its more aggressive subclone name could induce platelet aggregation and identifying novel proteins that could be the cause of this and indeed the other adverse effects we have found to be transmitted by these EVs [18, 33].

We first set out to establish if the TNBC cell line variants caused TCIPA. Both cell line variants induced aggregation when added to platelets, although, there was no significant difference between the effects among the two cell line variants. Next, having successfully collected and characterised EVs from both cell line variants, the EVs derived from both variants were tested for their ability to induce aggregation. Reflecting the activities of their cells of origin, the EVs released from both cell line variants caused platelet aggregation and in a dose-dependent manner. When the numbers of EVs added were increased, the time for platelet aggregation to occur was reduced. This suggests that EVs from all TNBCs, whether they are more or less aggressive, may contribute to platelets aggregation and thrombosis. This study is one of only few studies that have investigated the role of cancer cell EVs on platelet aggregation – and the only study that performed proteomics profiling of the EV cargo to identify proteins that may be causally involved. Of the studies that investigated cancer cell derived EVs, as mentioned above, Gomes et al. [17] used MDA-MB-231 and MCF-7 EVs to report breast cancer EVs are capable of causing platelet aggregation through direct platelet interaction. Although using different methods than we used to measure platelet aggregation and not following through with proteomics profiling, the main finding from their study was that both TF-dependent and TF-independent mechanisms were involved in MDA-MB-231 EVs-induced platelet aggregation. EVs from the less aggressive ER+ MCF-7 cells caused less aggregation. Another group established that MDA-MB-231-derived EVs can generate thrombin due to bound TF [39].

Global proteomic profiling of the Hs578T and Hs578Ts(i)_8_ EVs showed they contained common EV protein markers such as tetraspanins CD9 and CD81 with the proteins identified in both EVs being associated with the exosomal compartment upon cellular component analysis. When compared to each other, each variants’ EVs had five exclusive proteins i.e. that were undetected in the other variants’ EVs. Interestingly, these ten proteins have not been extensively studied, with only a small number of studies indicating their roles in cellular functions and with no link to platelet activity. For example, Hs578T-derived EVs contained aminopeptidase N or CD13, a type II metalloproteinase that can exist in the membrane and in a soluble form [40]. This protein has not been extensively studied and has not yet been linked to platelet function. Of potential interest, however, one study reported that CD13 levels positively correlate with neo-angiogenesis when staining breast carcinoma-derived tumour cells [41]. Some of the exclusive proteins found in the Hs578Ts(i)_8_ derived EVs included aldo-keto reductase family 1 member C2, elongin-c, and ribosome production factor 2 homolog (RPF2). No reports have been made to date linking RPF2 to TNBC disease or having a role with platelet functions. Again of interest, silencing the protein, suggest that RPF2 may play a role in the epithelial-to-mesenchymal (EMT) transition in colorectal cancer cells [42].

When compared to the vesiclepedia database, which includes all proteins reported therein in EVs, the TNBC EVs contained nineteen proteins that have not previously been reported. These proteins include ATP synthase subunit B, septins, elongin-c and histone H3. Of particular interest, platelet-related pathways were also associated with the vesicular proteins, with TF, an established pro-coagulant marker of EVs being identified in both TNBC EVs. With the well-studied association between cancer and thrombosis, we wanted to confirm the presence of some pro-coagulant proteins in the TNBC EVs (see Supplemental Material for full list of proteins identified).

In addition to confirming EV-carried TF in this study, we identified the presence, some for the first time, of several proteins as EVs cargo and which have been reported to have a potential role in platelet activation and function. These are PDGFRβ, Cyr61, CD97, Glypican-1, MUC18/CD146 and uPAR. Immunoblotting was used to confirm the presence of each protein in the EVs as it is a routinely available method unlike mass spectrometry. However, immunoblotting is at best semi-quantitative Thus some differences between the two methods were observed, but the same trends were observed in the levels of the proteins detected by both mass spectrometry and immunoblot. For example, Cyr61, a heparin-binding extracellular matrix-associated protein, has previously been shown to be present in MCF-7 and MDA-MB-231 TNBC cells but that study did not involve analysis of EVs [43] and it has been established that platelets can bind to microtiter wells coated with purified Cyr61 protein [44]. Our discovery of the presence of Cyr61 in the TNBC EVs leads to the possibility that the EVs bearing Cyr61 could bind to and activate platelets via Cyr61. In addition, CD97, an adhesion G-protein coupled receptor (GPCR), has been identified in the tumour cell-to-platelet interactions [45], although, again, those studies did not involve EVs. This study demonstrated that prostate cancer cells, DU145/Ras were shown to bind to platelets via CD97 which aided the metastasis of the tumour cells. With CD97 carried by Hs578T and Hs578Ts(i)_8_ EVs, we postulate that this protein may be involved in platelet activation. All six proteins were shown to be carried by both TNBC EVs by immunoblot, backing up the proteomic profiling and identifying new proteins that, to our knowledge, have not been reported to be within EVs before this study. Similar EV studies but with platelets from TNBC patients are now warranted.

Overall, this study has shown that TNBC EVs contain protein cargo which may be involved in TCIPA. We demonstrated that EVs, like their cancer cells of origin, can induce platelet aggregation in a dose-dependent manner and carry proteins that have not been identified in the EVs. Future studies should investigate the functional relevance of some of the proteins identified as targeting those proteins may be a future treatment strategy to prevent cancer-associated thrombosis.

## Supplementary Information

**Additional file 1.**

## Data Availability

The datasets generated and analysed during this study is available from the corresponding author on reasonable request.
