# Supplementary material for: Proteomics profiling identifies extracellular vesicles’ cargo associated with tumour cell induced platelet aggregation
Source: BMC Cancer. 2022 Sep 29;22:1023. doi: 10.1186/s12885-022-10068-7 (PMC9520807; doi:10.1186/s12885-022-10068-7)
Supplement: Supplementary file 1 — Additional file 1. [file 12885_2022_10068_MOESM1_ESM.pdf]

Supplementary Material

Blot 1:

Calnexin

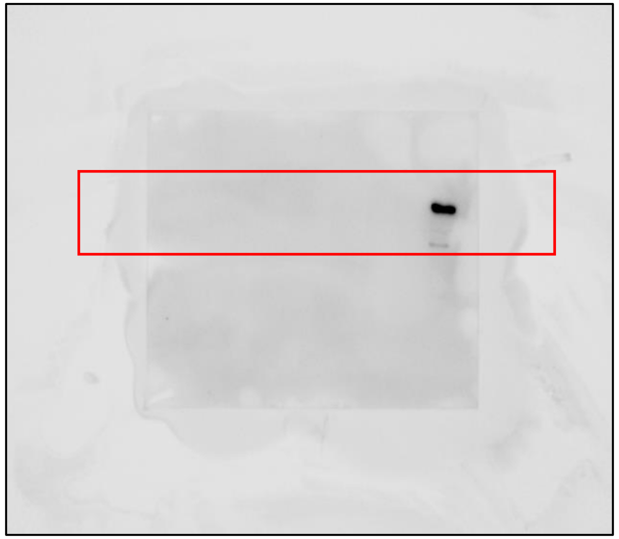

Syntenin

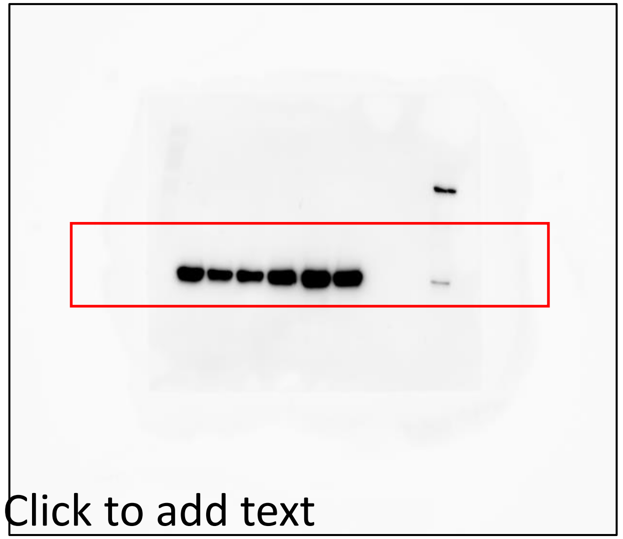

Flotillin

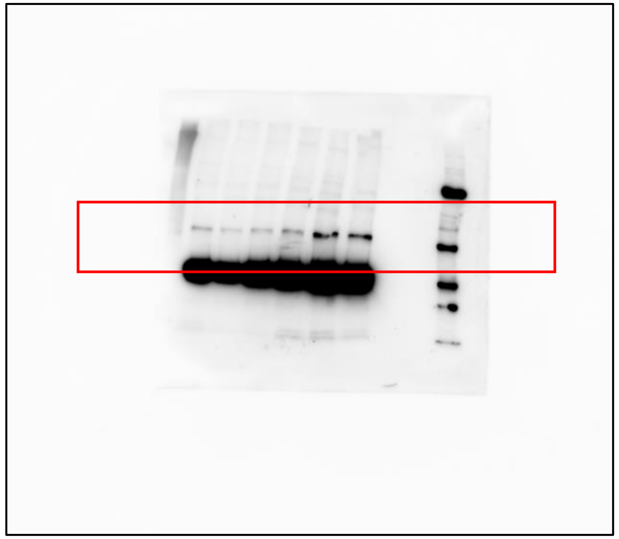

Blot 2:

Actinin-4

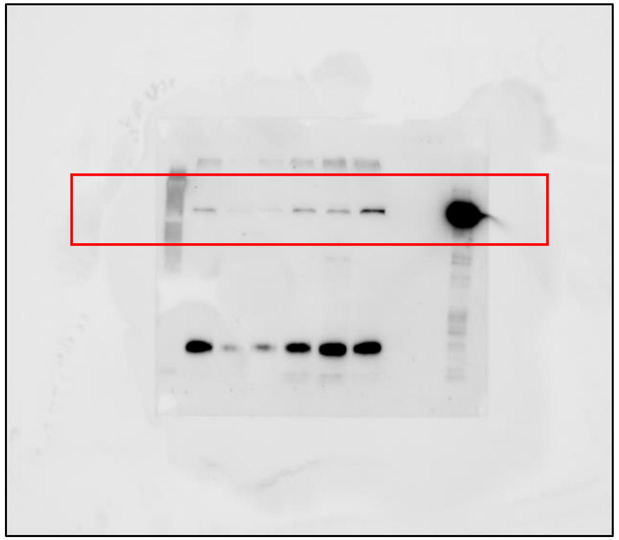

CD63

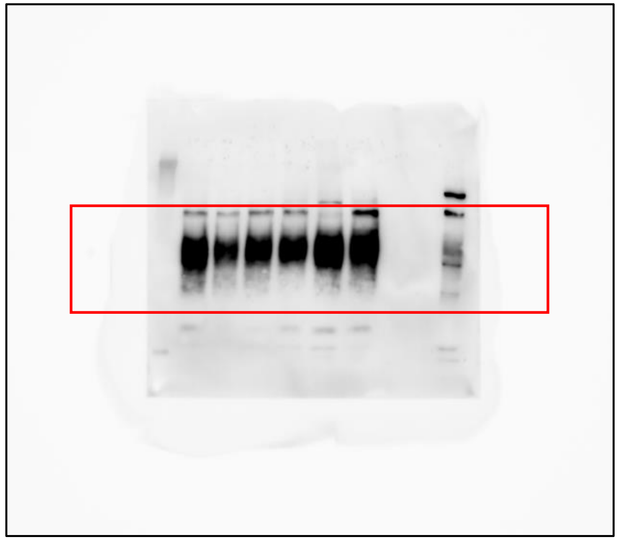

Figure S_1D

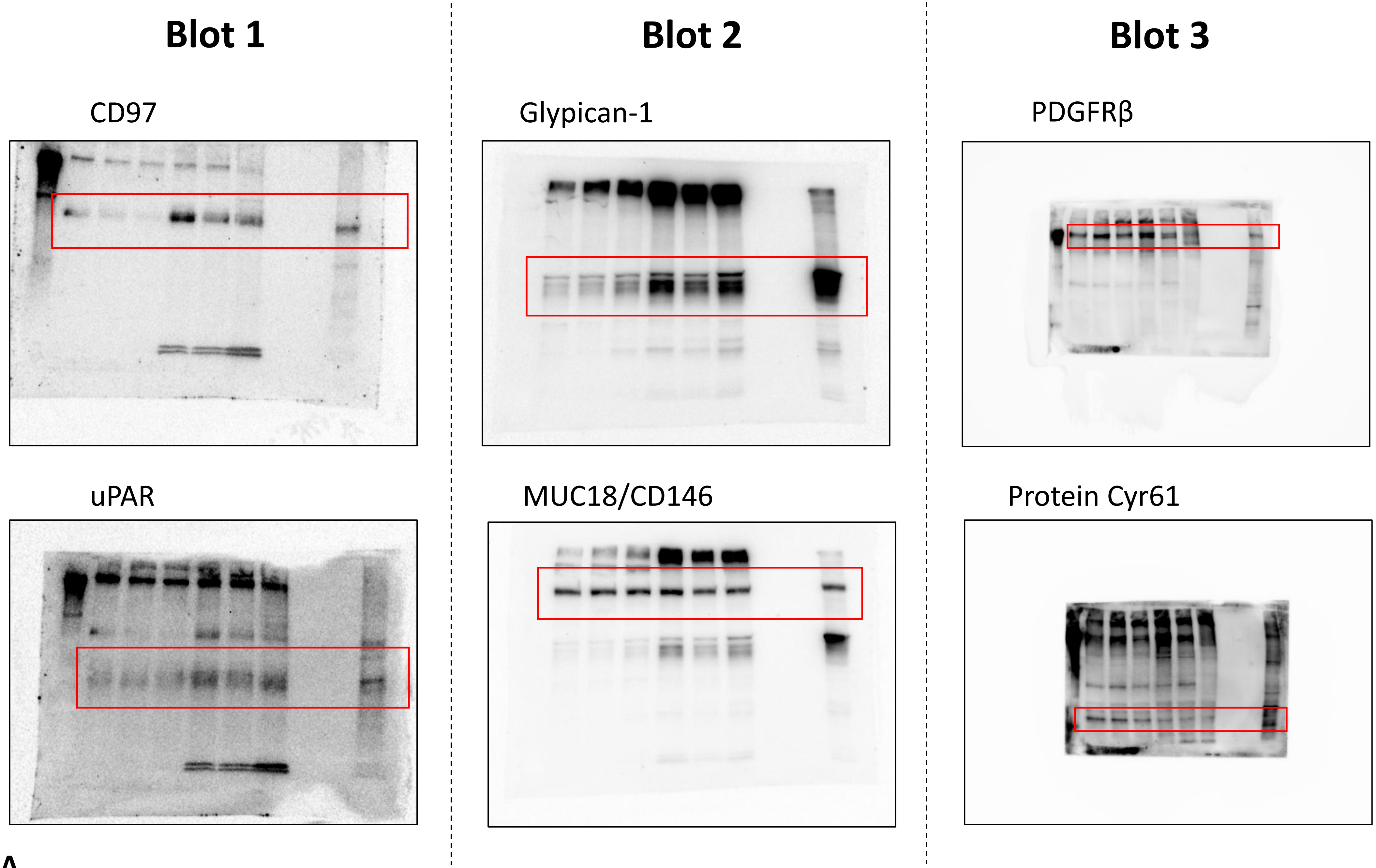

Blot 1
CD97
uPAR
Blot 2
Glypican-1
MUC18/CD146
Blot 3
PDGFRβ
Protein Cyr61

Figure S_6A

Supplement to Figure 2 (D): Two-way ANOVA statistical results

Compare cell means regardless of rows and columns

Number of families 1

Number of comparisons per family 28

Alpha 0.05

Tukey's multiple comparisons test	Mean Diff.	95.00% CI of diff.	Below threshold?	Summary	Adjusted P Value			
Tyrode's Buffer (control):Hs578T vs. Tyrode's Buffer (control):Hs578Ts(i)8	0.000	-4.465 to 4.465	No	ns	>0.9999			
Tyrode's Buffer (control):Hs578T vs. 250:Hs578T	-88.00	-92.47 to -83.53	Yes	****	<0.0001			
Tyrode's Buffer (control):Hs578T vs. 250:Hs578Ts(i)8	-90.50	-94.97 to -86.03	Yes	****	<0.0001			
Tyrode's Buffer (control):Hs578T vs. 500:Hs578T	-91.00	-95.47 to -86.53	Yes	****	<0.0001			
Tyrode's Buffer (control):Hs578T vs. 500:Hs578Ts(i)8	-89.75	-94.22 to -85.28	Yes	****	<0.0001			
Tyrode's Buffer (control):Hs578T vs. 1000:Hs578T	-93.75	-98.22 to -89.28	Yes	****	<0.0001			

Tyrode's Buffer (control):Hs578T vs. 1000:Hs578Ts(i)8	-91.25	-95.72 to -86.78	Yes	****	<0.0001			
Tyrode's Buffer (control):Hs578Ts(i)8 vs. 250:Hs578T	-88.00	-92.47 to -83.53	Yes	****	<0.0001			
Tyrode's Buffer (control):Hs578Ts(i)8 vs. 250:Hs578Ts(i)8	-90.50	-94.97 to -86.03	Yes	****	<0.0001			
Tyrode's Buffer (control):Hs578Ts(i)8 vs. 500:Hs578T	-91.00	-95.47 to -86.53	Yes	****	<0.0001			
Tyrode's Buffer (control):Hs578Ts(i)8 vs. 500:Hs578Ts(i)8	-89.75	-94.22 to -85.28	Yes	****	<0.0001			
Tyrode's Buffer (control):Hs578Ts(i)8 vs. 1000:Hs578T	-93.75	-98.22 to -89.28	Yes	****	<0.0001			
Tyrode's Buffer (control):Hs578Ts(i)8 vs. 1000:Hs578Ts(i)8	-91.25	-95.72 to -86.78	Yes	****	<0.0001			
250:Hs578T vs. 250:Hs578Ts(i)8	-2.500	-6.965 to 1.965	No	ns	0.5922			
250:Hs578T vs. 500:Hs578T	-3.000	-7.465 to 1.465	No	ns	0.3724			
250:Hs578T vs. 500:Hs578Ts(i)8	-1.750	-6.215 to 2.715	No	ns	0.8908			
250:Hs578T vs. 1000:Hs578T	-5.750	-10.22 to -1.285	Yes	**	0.0056			
250:Hs578T vs. 1000:Hs578Ts(i)8	-3.250	-7.715 to 1.215	No	ns	0.2808			
250:Hs578Ts(i)8 vs. 500:Hs578T	-0.5000	-4.965 to 3.965	No	ns	>0.9999			
250:Hs578Ts(i)8 vs. 500:Hs578Ts(i)8	0.7500	-3.715 to 5.215	No	ns	0.9991			

250:Hs578Ts(i)8 vs. 1000:Hs578T	-3.250	-7.715 to 1.215	No	ns	0.2808			
250:Hs578Ts(i)8 vs. 1000:Hs578Ts(i)8	-0.7500	-5.215 to 3.715	No	ns	0.9991			
500:Hs578T vs. 500:Hs578Ts(i)8	1.250	-3.215 to 5.715	No	ns	0.9802			
500:Hs578T vs. 1000:Hs578T	-2.750	-7.215 to 1.715	No	ns	0.4781			
500:Hs578T vs. 1000:Hs578Ts(i)8	-0.2500	-4.715 to 4.215	No	ns	>0.9999			
500:Hs578Ts(i)8 vs. 1000:Hs578T	-4.000	-8.465 to 0.4652	No	ns	0.1025			
500:Hs578Ts(i)8 vs. 1000:Hs578Ts(i)8	-1.500	-5.965 to 2.965	No	ns	0.9477			
1000:Hs578T vs. 1000:Hs578Ts(i)8	2.500	-1.965 to 6.965	No	ns	0.5922			
Test details	Mean 1	Mean 2	Mean Diff.	SE of diff.	N1	N2	q	DF
Tyrode's Buffer (control):Hs578T vs. Tyrode's Buffer (control):Hs578Ts(i)8	0.000	0.000	0.000	1.348	4	4	0.000	24.00
Tyrode's Buffer (control):Hs578T vs. 250:Hs578T	0.000	88.00	-88.00	1.348	4	4	92.31	24.00
Tyrode's Buffer (control):Hs578T vs. 250:Hs578Ts(i)8	0.000	90.50	-90.50	1.348	4	4	94.93	24.00
Tyrode's Buffer (control):Hs578T vs. 500:Hs578T	0.000	91.00	-91.00	1.348	4	4	95.45	24.00
Tyrode's Buffer (control):Hs578T vs. 500:Hs578Ts(i)8	0.000	89.75	-89.75	1.348	4	4	94.14	24.00

Tyrode's Buffer (control):Hs578T vs. 1000:Hs578T	0.000	93.75	-93.75	1.348	4	4	98.34	24.00
Tyrode's Buffer (control):Hs578T vs. 1000:Hs578Ts(i)8	0.000	91.25	-91.25	1.348	4	4	95.72	24.00
Tyrode's Buffer (control):Hs578Ts(i)8 vs. 250:Hs578T	0.000	88.00	-88.00	1.348	4	4	92.31	24.00
Tyrode's Buffer (control):Hs578Ts(i)8 vs. 250:Hs578Ts(i)8	0.000	90.50	-90.50	1.348	4	4	94.93	24.00
Tyrode's Buffer (control):Hs578Ts(i)8 vs. 500:Hs578T	0.000	91.00	-91.00	1.348	4	4	95.45	24.00
Tyrode's Buffer (control):Hs578Ts(i)8 vs. 500:Hs578Ts(i)8	0.000	89.75	-89.75	1.348	4	4	94.14	24.00
Tyrode's Buffer (control):Hs578Ts(i)8 vs. 1000:Hs578T	0.000	93.75	-93.75	1.348	4	4	98.34	24.00
Tyrode's Buffer (control):Hs578Ts(i)8 vs. 1000:Hs578Ts(i)8	0.000	91.25	-91.25	1.348	4	4	95.72	24.00
250:Hs578T vs. 250:Hs578Ts(i)8	88.00	90.50	-2.500	1.348	4	4	2.622	24.00
250:Hs578T vs. 500:Hs578T	88.00	91.00	-3.000	1.348	4	4	3.147	24.00
250:Hs578T vs. 500:Hs578Ts(i)8	88.00	89.75	-1.750	1.348	4	4	1.836	24.00
250:Hs578T vs. 1000:Hs578T	88.00	93.75	-5.750	1.348	4	4	6.031	24.00
250:Hs578T vs. 1000:Hs578Ts(i)8	88.00	91.25	-3.250	1.348	4	4	3.409	24.00
250:Hs578Ts(i)8 vs. 500:Hs578T	90.50	91.00	-0.5000	1.348	4	4	0.5245	24.00

250:Hs578Ts(i)8 vs. 500:Hs578Ts(i)8	90.50	89.75	0.7500	1.348	4	4	0.7867	24.00
250:Hs578Ts(i)8 vs. 1000:Hs578T	90.50	93.75	-3.250	1.348	4	4	3.409	24.00
250:Hs578Ts(i)8 vs. 1000:Hs578Ts(i)8	90.50	91.25	-0.7500	1.348	4	4	0.7867	24.00
500:Hs578T vs. 500:Hs578Ts(i)8	91.00	89.75	1.250	1.348	4	4	1.311	24.00
500:Hs578T vs. 1000:Hs578T	91.00	93.75	-2.750	1.348	4	4	2.885	24.00
500:Hs578T vs. 1000:Hs578Ts(i)8	91.00	91.25	-0.2500	1.348	4	4	0.2622	24.00
500:Hs578Ts(i)8 vs. 1000:Hs578T	89.75	93.75	-4.000	1.348	4	4	4.196	24.00
500:Hs578Ts(i)8 vs. 1000:Hs578Ts(i)8	89.75	91.25	-1.500	1.348	4	4	1.573	24.00
1000:Hs578T vs. 1000:Hs578Ts(i)8	93.75	91.25	2.500	1.348	4	4	2.622	24.00

Supplement to Figure 3 (D): Two-way ANOVA statistical results

Compare cell means regardless of rows and columns

Number of families 1

Number of comparisons per family 231

Alpha 0.05

Tukey's multiple comparisons test	Mean Diff.	95.00% CI of diff.	Below threshold?	Summary	Adjusted P Value			
Control (PBS): Hs578T vs. Control (PBS):Hs578Ts(i)8	0.000	-357.0 to 357.0	No	ns	>0.9999			
Control (PBS): Hs578T vs. 2.2x107: Hs578T	671.8	314.8 to 1029	Yes	****	<0.0001			
Control (PBS): Hs578T vs. 2.2x107:Hs578Ts(i)8	532.5	175.5 to 889.5	Yes	****	<0.0001			
Control (PBS): Hs578T vs. 4.5x107: Hs578T	932.0	575.0 to 1289	Yes	****	<0.0001			
Control (PBS): Hs578T vs. 4.5x107:Hs578Ts(i)8	788.0	431.0 to 1145	Yes	****	<0.0001			
Control (PBS): Hs578T vs. 1.1x108: Hs578T	1116	758.5 to 1472	Yes	****	<0.0001			
Control (PBS): Hs578T vs. 1.1x108:Hs578Ts(i)8	1085	728.3 to 1442	Yes	****	<0.0001			
Control (PBS): Hs578T vs. 2.2x108: Hs578T	1275	917.8 to 1632	Yes	****	<0.0001			
Control (PBS): Hs578T vs. 2.2x108:Hs578Ts(i)8	1261	904.3 to 1618	Yes	****	<0.0001			

Control (PBS): Hs578T vs. 4.4x108: Hs578T	1485	1128 to 1842	Yes	****	<0.0001			
Control (PBS): Hs578T vs. 4.4x108:Hs578Ts(i)8	1369	1012 to 1725	Yes	****	<0.0001			
Control (PBS): Hs578T vs. 11.1x108: Hs578T	1547	1190 to 1904	Yes	****	<0.0001			
Control (PBS): Hs578T vs. 11.1x108:Hs578Ts(i)8	1498	1141 to 1854	Yes	****	<0.0001			
Control (PBS): Hs578T vs. 22.3x108: Hs578T	1564	1207 to 1921	Yes	****	<0.0001			
Control (PBS): Hs578T vs. 22.3x108:Hs578Ts(i)8	1560	1203 to 1917	Yes	****	<0.0001			
Control (PBS): Hs578T vs. 4.5x109: Hs578T	1591	1234 to 1948	Yes	****	<0.0001			
Control (PBS): Hs578T vs. 4.5x109:Hs578Ts(i)8	1594	1237 to 1950	Yes	****	<0.0001			
Control (PBS): Hs578T vs. 6.7x109: Hs578T	1608	1251 to 1965	Yes	****	<0.0001			
Control (PBS): Hs578T vs. 6.7x109:Hs578Ts(i)8	1625	1268 to 1982	Yes	****	<0.0001			
Control (PBS): Hs578T vs. 8.9x109: Hs578T	1622	1265 to 1979	Yes	****	<0.0001			
Control (PBS): Hs578T vs. 8.9x109:Hs578Ts(i)8	1676	1319 to 2032	Yes	****	<0.0001			
Control (PBS):Hs578Ts(i)8 vs. 2.2x107: Hs578T	671.8	314.8 to 1029	Yes	****	<0.0001			
Control (PBS):Hs578Ts(i)8 vs. 2.2x107:Hs578Ts(i)8	532.5	175.5 to 889.5	Yes	****	<0.0001			
Control (PBS):Hs578Ts(i)8 vs. 4.5x107: Hs578T	932.0	575.0 to 1289	Yes	****	<0.0001			

Control (PBS):Hs578Ts(i)8 vs. 4.5x107:Hs578Ts(i)8	788.0	431.0 to 1145	Yes	****	<0.0001			
Control (PBS):Hs578Ts(i)8 vs. 1.1x108: Hs578T	1116	758.5 to 1472	Yes	****	<0.0001			
Control (PBS):Hs578Ts(i)8 vs. 1.1x108:Hs578Ts(i)8	1085	728.3 to 1442	Yes	****	<0.0001			
Control (PBS):Hs578Ts(i)8 vs. 2.2x108: Hs578T	1275	917.8 to 1632	Yes	****	<0.0001			
Control (PBS):Hs578Ts(i)8 vs. 2.2x108:Hs578Ts(i)8	1261	904.3 to 1618	Yes	****	<0.0001			
Control (PBS):Hs578Ts(i)8 vs. 4.4x108: Hs578T	1485	1128 to 1842	Yes	****	<0.0001			
Control (PBS):Hs578Ts(i)8 vs. 4.4x108:Hs578Ts(i)8	1369	1012 to 1725	Yes	****	<0.0001			
Control (PBS):Hs578Ts(i)8 vs. 11.1x108: Hs578T	1547	1190 to 1904	Yes	****	<0.0001			
Control (PBS):Hs578Ts(i)8 vs. 11.1x108:Hs578Ts(i)8	1498	1141 to 1854	Yes	****	<0.0001			
Control (PBS):Hs578Ts(i)8 vs. 22.3x108: Hs578T	1564	1207 to 1921	Yes	****	<0.0001			
Control (PBS):Hs578Ts(i)8 vs. 22.3x108:Hs578Ts(i)8	1560	1203 to 1917	Yes	****	<0.0001			
Control (PBS):Hs578Ts(i)8 vs. 4.5x109: Hs578T	1591	1234 to 1948	Yes	****	<0.0001			

Control (PBS):Hs578Ts(i)8 vs. 4.5x109:Hs578Ts(i)8	1594	1237 to 1950	Yes	****	<0.0001			
Control (PBS):Hs578Ts(i)8 vs. 6.7x109: Hs578T	1608	1251 to 1965	Yes	****	<0.0001			
Control (PBS):Hs578Ts(i)8 vs. 6.7x109:Hs578Ts(i)8	1625	1268 to 1982	Yes	****	<0.0001			
Control (PBS):Hs578Ts(i)8 vs. 8.9x109: Hs578T	1622	1265 to 1979	Yes	****	<0.0001			
Control (PBS):Hs578Ts(i)8 vs. 8.9x109:Hs578Ts(i)8	1676	1319 to 2032	Yes	****	<0.0001			
2.2x107: Hs578T vs. 2.2x107:Hs578Ts(i)8	-139.3	-496.2 to 217.7	No	ns	0.9970			
2.2x107: Hs578T vs. 4.5x107: Hs578T	260.3	-96.74 to 617.2	No	ns	0.4663			
2.2x107: Hs578T vs. 4.5x107:Hs578Ts(i)8	116.3	-240.7 to 473.2	No	ns	0.9998			
2.2x107: Hs578T vs. 1.1x108: Hs578T	443.8	86.76 to 800.7	Yes	**	0.0029			
2.2x107: Hs578T vs. 1.1x108:Hs578Ts(i)8	413.5	56.51 to 770.5	Yes	**	0.0083			
2.2x107: Hs578T vs. 2.2x108: Hs578T	603.0	246.0 to 960.0	Yes	****	<0.0001			
2.2x107: Hs578T vs. 2.2x108:Hs578Ts(i)8	589.5	232.5 to 946.5	Yes	****	<0.0001			
2.2x107: Hs578T vs. 4.4x108: Hs578T	813.3	456.3 to 1170	Yes	****	<0.0001			
2.2x107: Hs578T vs. 4.4x108:Hs578Ts(i)8	696.8	339.8 to 1054	Yes	****	<0.0001			
2.2x107: Hs578T vs. 11.1x108: Hs578T	875.5	518.5 to 1232	Yes	****	<0.0001			

2.2x107: Hs578T vs. 11.1x108:Hs578Ts(i)8	825.8	468.8 to 1183	Yes	****	<0.0001			
2.2x107: Hs578T vs. 22.3x108: Hs578T	892.3	535.3 to 1249	Yes	****	<0.0001			
2.2x107: Hs578T vs. 22.3x108:Hs578Ts(i)8	888.0	531.0 to 1245	Yes	****	<0.0001			
2.2x107: Hs578T vs. 4.5x109: Hs578T	919.5	562.5 to 1276	Yes	****	<0.0001			
2.2x107: Hs578T vs. 4.5x109:Hs578Ts(i)8	921.8	564.8 to 1279	Yes	****	<0.0001			
2.2x107: Hs578T vs. 6.7x109: Hs578T	936.5	579.5 to 1293	Yes	****	<0.0001			
2.2x107: Hs578T vs. 6.7x109:Hs578Ts(i)8	953.3	596.3 to 1310	Yes	****	<0.0001			
2.2x107: Hs578T vs. 8.9x109: Hs578T	950.0	593.0 to 1307	Yes	****	<0.0001			
2.2x107: Hs578T vs. 8.9x109:Hs578Ts(i)8	1004	646.8 to 1361	Yes	****	<0.0001			
2.2x107:Hs578Ts(i)8 vs. 4.5x107: Hs578T	399.5	42.51 to 756.5	Yes	*	0.0133			
2.2x107:Hs578Ts(i)8 vs. 4.5x107:Hs578Ts(i)8	255.5	-101.5 to 612.5	No	ns	0.5017			
2.2x107:Hs578Ts(i)8 vs. 1.1x108: Hs578T	583.0	226.0 to 940.0	Yes	****	<0.0001			
2.2x107:Hs578Ts(i)8 vs. 1.1x108:Hs578Ts(i)8	552.8	195.8 to 909.7	Yes	****	<0.0001			
2.2x107:Hs578Ts(i)8 vs. 2.2x108: Hs578T	742.3	385.3 to 1099	Yes	****	<0.0001			
2.2x107:Hs578Ts(i)8 vs. 2.2x108:Hs578Ts(i)8	728.8	371.8 to 1086	Yes	****	<0.0001			
2.2x107:Hs578Ts(i)8 vs. 4.4x108: Hs578T	952.5	595.5 to 1309	Yes	****	<0.0001			
2.2x107:Hs578Ts(i)8 vs. 4.4x108:Hs578Ts(i)8	836.0	479.0 to 1193	Yes	****	<0.0001			
2.2x107:Hs578Ts(i)8 vs. 11.1x108: Hs578T	1015	657.8 to 1372	Yes	****	<0.0001			

2.2x107:Hs578Ts(i)8 vs. 11.1x108:Hs578Ts(i)8	965.0	608.0 to 1322	Yes	****	<0.0001			
2.2x107:Hs578Ts(i)8 vs. 22.3x108: Hs578T	1032	674.5 to 1388	Yes	****	<0.0001			
2.2x107:Hs578Ts(i)8 vs. 22.3x108:Hs578Ts(i)8	1027	670.3 to 1384	Yes	****	<0.0001			
2.2x107:Hs578Ts(i)8 vs. 4.5x109: Hs578T	1059	701.8 to 1416	Yes	****	<0.0001			
2.2x107:Hs578Ts(i)8 vs. 4.5x109:Hs578Ts(i)8	1061	704.0 to 1418	Yes	****	<0.0001			
2.2x107:Hs578Ts(i)8 vs. 6.7x109: Hs578T	1076	718.8 to 1433	Yes	****	<0.0001			
2.2x107:Hs578Ts(i)8 vs. 6.7x109:Hs578Ts(i)8	1093	735.5 to 1449	Yes	****	<0.0001			
2.2x107:Hs578Ts(i)8 vs. 8.9x109: Hs578T	1089	732.3 to 1446	Yes	****	<0.0001			
2.2x107:Hs578Ts(i)8 vs. 8.9x109:Hs578Ts(i)8	1143	786.0 to 1500	Yes	****	<0.0001			
4.5x107: Hs578T vs. 4.5x107:Hs578Ts(i)8	-144.0	-501.0 to 213.0	No	ns	0.9955			
4.5x107: Hs578T vs. 1.1x108: Hs578T	183.5	-173.5 to 540.5	No	ns	0.9392			
4.5x107: Hs578T vs. 1.1x108:Hs578Ts(i)8	153.3	-203.7 to 510.2	No	ns	0.9904			
4.5x107: Hs578T vs. 2.2x108: Hs578T	342.8	-14.24 to 699.7	No	ns	0.0750			
4.5x107: Hs578T vs. 2.2x108:Hs578Ts(i)8	329.3	-27.74 to 686.2	No	ns	0.1077			
4.5x107: Hs578T vs. 4.4x108: Hs578T	553.0	196.0 to 910.0	Yes	****	<0.0001			
4.5x107: Hs578T vs. 4.4x108:Hs578Ts(i)8	436.5	79.51 to 793.5	Yes	**	0.0037			
4.5x107: Hs578T vs. 11.1x108: Hs578T	615.3	258.3 to 972.2	Yes	****	<0.0001			

4.5x107: Hs578T vs. 11.1x108:Hs578Ts(i)8	565.5	208.5 to 922.5	Yes	****	<0.0001			
4.5x107: Hs578T vs. 22.3x108: Hs578T	632.0	275.0 to 989.0	Yes	****	<0.0001			
4.5x107: Hs578T vs. 22.3x108:Hs578Ts(i)8	627.8	270.8 to 984.7	Yes	****	<0.0001			
4.5x107: Hs578T vs. 4.5x109: Hs578T	659.3	302.3 to 1016	Yes	****	<0.0001			
4.5x107: Hs578T vs. 4.5x109:Hs578Ts(i)8	661.5	304.5 to 1018	Yes	****	<0.0001			
4.5x107: Hs578T vs. 6.7x109: Hs578T	676.3	319.3 to 1033	Yes	****	<0.0001			
4.5x107: Hs578T vs. 6.7x109:Hs578Ts(i)8	693.0	336.0 to 1050	Yes	****	<0.0001			
4.5x107: Hs578T vs. 8.9x109: Hs578T	689.8	332.8 to 1047	Yes	****	<0.0001			
4.5x107: Hs578T vs. 8.9x109:Hs578Ts(i)8	743.5	386.5 to 1100	Yes	****	<0.0001			
4.5x107:Hs578Ts(i)8 vs. 1.1x108: Hs578T	327.5	-29.49 to 684.5	No	ns	0.1128			
4.5x107:Hs578Ts(i)8 vs. 1.1x108:Hs578Ts(i)8	297.3	-59.74 to 654.2	No	ns	0.2321			
4.5x107:Hs578Ts(i)8 vs. 2.2x108: Hs578T	486.8	129.8 to 843.7	Yes	***	0.0006			
4.5x107:Hs578Ts(i)8 vs. 2.2x108:Hs578Ts(i)8	473.3	116.3 to 830.2	Yes	***	0.0010			
4.5x107:Hs578Ts(i)8 vs. 4.4x108: Hs578T	697.0	340.0 to 1054	Yes	****	<0.0001			
4.5x107:Hs578Ts(i)8 vs. 4.4x108:Hs578Ts(i)8	580.5	223.5 to 937.5	Yes	****	<0.0001			
4.5x107:Hs578Ts(i)8 vs. 11.1x108: Hs578T	759.3	402.3 to 1116	Yes	****	<0.0001			
4.5x107:Hs578Ts(i)8 vs. 11.1x108:Hs578Ts(i)8	709.5	352.5 to 1066	Yes	****	<0.0001			

4.5x107:Hs578Ts(i)8 vs. 22.3x108: Hs578T	776.0	419.0 to 1133	Yes	****	<0.0001			
4.5x107:Hs578Ts(i)8 vs. 22.3x108:Hs578Ts(i)8	771.8	414.8 to 1129	Yes	****	<0.0001			
4.5x107:Hs578Ts(i)8 vs. 4.5x109: Hs578T	803.3	446.3 to 1160	Yes	****	<0.0001			
4.5x107:Hs578Ts(i)8 vs. 4.5x109:Hs578Ts(i)8	805.5	448.5 to 1162	Yes	****	<0.0001			
4.5x107:Hs578Ts(i)8 vs. 6.7x109: Hs578T	820.3	463.3 to 1177	Yes	****	<0.0001			
4.5x107:Hs578Ts(i)8 vs. 6.7x109:Hs578Ts(i)8	837.0	480.0 to 1194	Yes	****	<0.0001			
4.5x107:Hs578Ts(i)8 vs. 8.9x109: Hs578T	833.8	476.8 to 1191	Yes	****	<0.0001			
4.5x107:Hs578Ts(i)8 vs. 8.9x109:Hs578Ts(i)8	887.5	530.5 to 1244	Yes	****	<0.0001			
1.1x108: Hs578T vs. 1.1x108:Hs578Ts(i)8	-30.25	-387.2 to 326.7	No	ns	>0.9999			
1.1x108: Hs578T vs. 2.2x108: Hs578T	159.3	-197.7 to 516.2	No	ns	0.9852			
1.1x108: Hs578T vs. 2.2x108:Hs578Ts(i)8	145.8	-211.2 to 502.7	No	ns	0.9947			
1.1x108: Hs578T vs. 4.4x108: Hs578T	369.5	12.51 to 726.5	Yes	*	0.0344			
1.1x108: Hs578T vs. 4.4x108:Hs578Ts(i)8	253.0	-104.0 to 610.0	No	ns	0.5205			
1.1x108: Hs578T vs. 11.1x108: Hs578T	431.8	74.76 to 788.7	Yes	**	0.0044			
1.1x108: Hs578T vs. 11.1x108:Hs578Ts(i)8	382.0	25.01 to 739.0	Yes	*	0.0234			
1.1x108: Hs578T vs. 22.3x108: Hs578T	448.5	91.51 to 805.5	Yes	**	0.0024			
1.1x108: Hs578T vs. 22.3x108:Hs578Ts(i)8	444.3	87.26 to 801.2	Yes	**	0.0028			

1.1x108: Hs578T vs. 4.5x109: Hs578T	475.8	118.8 to 832.7	Yes	***	0.0009			
1.1x108: Hs578T vs. 4.5x109:Hs578Ts(i)8	478.0	121.0 to 835.0	Yes	***	0.0008			
1.1x108: Hs578T vs. 6.7x109: Hs578T	492.8	135.8 to 849.7	Yes	***	0.0005			
1.1x108: Hs578T vs. 6.7x109:Hs578Ts(i)8	509.5	152.5 to 866.5	Yes	***	0.0002			
1.1x108: Hs578T vs. 8.9x109: Hs578T	506.3	149.3 to 863.2	Yes	***	0.0003			
1.1x108: Hs578T vs. 8.9x109:Hs578Ts(i)8	560.0	203.0 to 917.0	Yes	****	<0.0001			
1.1x108:Hs578Ts(i)8 vs. 2.2x108: Hs578T	189.5	-167.5 to 546.5	No	ns	0.9196			
1.1x108:Hs578Ts(i)8 vs. 2.2x108:Hs578Ts(i)8	176.0	-181.0 to 533.0	No	ns	0.9587			
1.1x108:Hs578Ts(i)8 vs. 4.4x108: Hs578T	399.8	42.76 to 756.7	Yes	*	0.0132			
1.1x108:Hs578Ts(i)8 vs. 4.4x108:Hs578Ts(i)8	283.3	-73.74 to 640.2	No	ns	0.3101			
1.1x108:Hs578Ts(i)8 vs. 11.1x108: Hs578T	462.0	105.0 to 819.0	Yes	**	0.0015			
1.1x108:Hs578Ts(i)8 vs. 11.1x108:Hs578Ts(i)8	412.3	55.26 to 769.2	Yes	**	0.0087			
1.1x108:Hs578Ts(i)8 vs. 22.3x108: Hs578T	478.8	121.8 to 835.7	Yes	***	0.0008			
1.1x108:Hs578Ts(i)8 vs. 22.3x108:Hs578Ts(i)8	474.5	117.5 to 831.5	Yes	***	0.0009			
1.1x108:Hs578Ts(i)8 vs. 4.5x109: Hs578T	506.0	149.0 to 863.0	Yes	***	0.0003			
1.1x108:Hs578Ts(i)8 vs. 4.5x109:Hs578Ts(i)8	508.3	151.3 to 865.2	Yes	***	0.0003			
1.1x108:Hs578Ts(i)8 vs. 6.7x109: Hs578T	523.0	166.0 to 880.0	Yes	***	0.0001			

1.1x108:Hs578Ts(i)8 vs. 6.7x109:Hs578Ts(i)8	539.8	182.8 to 896.7	Yes	****	<0.0001			
1.1x108:Hs578Ts(i)8 vs. 8.9x109: Hs578T	536.5	179.5 to 893.5	Yes	****	<0.0001			
1.1x108:Hs578Ts(i)8 vs. 8.9x109:Hs578Ts(i)8	590.3	233.3 to 947.2	Yes	****	<0.0001			
2.2x108: Hs578T vs. 2.2x108:Hs578Ts(i)8	-13.50	-370.5 to 343.5	No	ns	>0.9999			
2.2x108: Hs578T vs. 4.4x108: Hs578T	210.3	-146.7 to 567.2	No	ns	0.8226			
2.2x108: Hs578T vs. 4.4x108:Hs578Ts(i)8	93.75	-263.2 to 450.7	No	ns	>0.9999			
2.2x108: Hs578T vs. 11.1x108: Hs578T	272.5	-84.49 to 629.5	No	ns	0.3794			
2.2x108: Hs578T vs. 11.1x108:Hs578Ts(i)8	222.8	-134.2 to 579.7	No	ns	0.7441			
2.2x108: Hs578T vs. 22.3x108: Hs578T	289.3	-67.74 to 646.2	No	ns	0.2749			
2.2x108: Hs578T vs. 22.3x108:Hs578Ts(i)8	285.0	-71.99 to 642.0	No	ns	0.2996			
2.2x108: Hs578T vs. 4.5x109: Hs578T	316.5	-40.49 to 673.5	No	ns	0.1487			
2.2x108: Hs578T vs. 4.5x109:Hs578Ts(i)8	318.8	-38.24 to 675.7	No	ns	0.1407			
2.2x108: Hs578T vs. 6.7x109: Hs578T	333.5	-23.49 to 690.5	No	ns	0.0963			
2.2x108: Hs578T vs. 6.7x109:Hs578Ts(i)8	350.3	-6.740 to 707.2	No	ns	0.0607			
2.2x108: Hs578T vs. 8.9x109: Hs578T	347.0	-9.990 to 704.0	No	ns	0.0666			
2.2x108: Hs578T vs. 8.9x109:Hs578Ts(i)8	400.8	43.76 to 757.7	Yes	*	0.0127			
2.2x108:Hs578Ts(i)8 vs. 4.4x108: Hs578T	223.8	-133.2 to 580.7	No	ns	0.7372			
2.2x108:Hs578Ts(i)8 vs. 4.4x108:Hs578Ts(i)8	107.3	-249.7 to 464.2	No	ns	>0.9999			

2.2x108:Hs578Ts(i)8 vs. 11.1x108: Hs578T	286.0	-70.99 to 643.0	No	ns	0.2937			
2.2x108:Hs578Ts(i)8 vs. 11.1x108:Hs578Ts(i)8	236.3	-120.7 to 593.2	No	ns	0.6474			
2.2x108:Hs578Ts(i)8 vs. 22.3x108: Hs578T	302.8	-54.24 to 659.7	No	ns	0.2055			
2.2x108:Hs578Ts(i)8 vs. 22.3x108:Hs578Ts(i)8	298.5	-58.49 to 655.5	No	ns	0.2259			
2.2x108:Hs578Ts(i)8 vs. 4.5x109: Hs578T	330.0	-26.99 to 687.0	No	ns	0.1057			
2.2x108:Hs578Ts(i)8 vs. 4.5x109:Hs578Ts(i)8	332.3	-24.74 to 689.2	No	ns	0.0996			
2.2x108:Hs578Ts(i)8 vs. 6.7x109: Hs578T	347.0	-9.990 to 704.0	No	ns	0.0666			
2.2x108:Hs578Ts(i)8 vs. 6.7x109:Hs578Ts(i)8	363.8	6.760 to 720.7	Yes	*	0.0410			
2.2x108:Hs578Ts(i)8 vs. 8.9x109: Hs578T	360.5	3.510 to 717.5	Yes	*	0.0451			
2.2x108:Hs578Ts(i)8 vs. 8.9x109:Hs578Ts(i)8	414.3	57.26 to 771.2	Yes	**	0.0081			
4.4x108: Hs578T vs. 4.4x108:Hs578Ts(i)8	-116.5	-473.5 to 240.5	No	ns	0.9997			
4.4x108: Hs578T vs. 11.1x108: Hs578T	62.25	-294.7 to 419.2	No	ns	>0.9999			
4.4x108: Hs578T vs. 11.1x108:Hs578Ts(i)8	12.50	-344.5 to 369.5	No	ns	>0.9999			
4.4x108: Hs578T vs. 22.3x108: Hs578T	79.00	-278.0 to 436.0	No	ns	>0.9999			
4.4x108: Hs578T vs. 22.3x108:Hs578Ts(i)8	74.75	-282.2 to 431.7	No	ns	>0.9999			
4.4x108: Hs578T vs. 4.5x109: Hs578T	106.3	-250.7 to 463.2	No	ns	>0.9999			
4.4x108: Hs578T vs. 4.5x109:Hs578Ts(i)8	108.5	-248.5 to 465.5	No	ns	>0.9999			

4.4x108: Hs578T vs. 6.7x109: Hs578T	123.3	-233.7 to 480.2	No	ns	0.9994			
4.4x108: Hs578T vs. 6.7x109:Hs578Ts(i)8	140.0	-217.0 to 497.0	No	ns	0.9968			
4.4x108: Hs578T vs. 8.9x109: Hs578T	136.8	-220.2 to 493.7	No	ns	0.9976			
4.4x108: Hs578T vs. 8.9x109:Hs578Ts(i)8	190.5	-166.5 to 547.5	No	ns	0.9160			
4.4x108:Hs578Ts(i)8 vs. 11.1x108: Hs578T	178.8	-178.2 to 535.7	No	ns	0.9522			
4.4x108:Hs578Ts(i)8 vs. 11.1x108:Hs578Ts(i)8	129.0	-228.0 to 486.0	No	ns	0.9989			
4.4x108:Hs578Ts(i)8 vs. 22.3x108: Hs578T	195.5	-161.5 to 552.5	No	ns	0.8963			
4.4x108:Hs578Ts(i)8 vs. 22.3x108:Hs578Ts(i)8	191.3	-165.7 to 548.2	No	ns	0.9132			
4.4x108:Hs578Ts(i)8 vs. 4.5x109: Hs578T	222.8	-134.2 to 579.7	No	ns	0.7441			
4.4x108:Hs578Ts(i)8 vs. 4.5x109:Hs578Ts(i)8	225.0	-132.0 to 582.0	No	ns	0.7286			
4.4x108:Hs578Ts(i)8 vs. 6.7x109: Hs578T	239.8	-117.2 to 596.7	No	ns	0.6211			
4.4x108:Hs578Ts(i)8 vs. 6.7x109:Hs578Ts(i)8	256.5	-100.5 to 613.5	No	ns	0.4942			
4.4x108:Hs578Ts(i)8 vs. 8.9x109: Hs578T	253.3	-103.7 to 610.2	No	ns	0.5186			
4.4x108:Hs578Ts(i)8 vs. 8.9x109:Hs578Ts(i)8	307.0	-49.99 to 664.0	No	ns	0.1864			
11.1x108: Hs578T vs. 11.1x108:Hs578Ts(i)8	-49.75	-406.7 to 307.2	No	ns	>0.9999			
11.1x108: Hs578T vs. 22.3x108: Hs578T	16.75	-340.2 to 373.7	No	ns	>0.9999			
11.1x108: Hs578T vs. 22.3x108:Hs578Ts(i)8	12.50	-344.5 to 369.5	No	ns	>0.9999			

11.1x108: Hs578T vs. 4.5x109: Hs578T	44.00	-313.0 to 401.0	No	ns	>0.9999			
11.1x108: Hs578T vs. 4.5x109:Hs578Ts(i)8	46.25	-310.7 to 403.2	No	ns	>0.9999			
11.1x108: Hs578T vs. 6.7x109: Hs578T	61.00	-296.0 to 418.0	No	ns	>0.9999			
11.1x108: Hs578T vs. 6.7x109:Hs578Ts(i)8	77.75	-279.2 to 434.7	No	ns	>0.9999			
11.1x108: Hs578T vs. 8.9x109: Hs578T	74.50	-282.5 to 431.5	No	ns	>0.9999			
11.1x108: Hs578T vs. 8.9x109:Hs578Ts(i)8	128.3	-228.7 to 485.2	No	ns	0.9990			
11.1x108:Hs578Ts(i)8 vs. 22.3x108: Hs578T	66.50	-290.5 to 423.5	No	ns	>0.9999			
11.1x108:Hs578Ts(i)8 vs. 22.3x108:Hs578Ts(i)8	62.25	-294.7 to 419.2	No	ns	>0.9999			
11.1x108:Hs578Ts(i)8 vs. 4.5x109: Hs578T	93.75	-263.2 to 450.7	No	ns	>0.9999			
11.1x108:Hs578Ts(i)8 vs. 4.5x109:Hs578Ts(i)8	96.00	-261.0 to 453.0	No	ns	>0.9999			
11.1x108:Hs578Ts(i)8 vs. 6.7x109: Hs578T	110.8	-246.2 to 467.7	No	ns	0.9999			
11.1x108:Hs578Ts(i)8 vs. 6.7x109:Hs578Ts(i)8	127.5	-229.5 to 484.5	No	ns	0.9991			
11.1x108:Hs578Ts(i)8 vs. 8.9x109: Hs578T	124.3	-232.7 to 481.2	No	ns	0.9994			
11.1x108:Hs578Ts(i)8 vs. 8.9x109:Hs578Ts(i)8	178.0	-179.0 to 535.0	No	ns	0.9540			
22.3x108: Hs578T vs. 22.3x108:Hs578Ts(i)8	-4.250	-361.2 to 352.7	No	ns	>0.9999			
22.3x108: Hs578T vs. 4.5x109: Hs578T	27.25	-329.7 to 384.2	No	ns	>0.9999			
22.3x108: Hs578T vs. 4.5x109:Hs578Ts(i)8	29.50	-327.5 to 386.5	No	ns	>0.9999			

22.3x108: Hs578T vs. 6.7x109: Hs578T	44.25	-312.7 to 401.2	No	ns	>0.9999			
22.3x108: Hs578T vs. 6.7x109:Hs578Ts(i)8	61.00	-296.0 to 418.0	No	ns	>0.9999			
22.3x108: Hs578T vs. 8.9x109: Hs578T	57.75	-299.2 to 414.7	No	ns	>0.9999			
22.3x108: Hs578T vs. 8.9x109:Hs578Ts(i)8	111.5	-245.5 to 468.5	No	ns	0.9999			
22.3x108:Hs578Ts(i)8 vs. 4.5x109: Hs578T	31.50	-325.5 to 388.5	No	ns	>0.9999			
22.3x108:Hs578Ts(i)8 vs. 4.5x109:Hs578Ts(i)8	33.75	-323.2 to 390.7	No	ns	>0.9999			
22.3x108:Hs578Ts(i)8 vs. 6.7x109: Hs578T	48.50	-308.5 to 405.5	No	ns	>0.9999			
22.3x108:Hs578Ts(i)8 vs. 6.7x109:Hs578Ts(i)8	65.25	-291.7 to 422.2	No	ns	>0.9999			
22.3x108:Hs578Ts(i)8 vs. 8.9x109: Hs578T	62.00	-295.0 to 419.0	No	ns	>0.9999			
22.3x108:Hs578Ts(i)8 vs. 8.9x109:Hs578Ts(i)8	115.8	-241.2 to 472.7	No	ns	0.9998			
4.5x109: Hs578T vs. 4.5x109:Hs578Ts(i)8	2.250	-354.7 to 359.2	No	ns	>0.9999			
4.5x109: Hs578T vs. 6.7x109: Hs578T	17.00	-340.0 to 374.0	No	ns	>0.9999			
4.5x109: Hs578T vs. 6.7x109:Hs578Ts(i)8	33.75	-323.2 to 390.7	No	ns	>0.9999			
4.5x109: Hs578T vs. 8.9x109: Hs578T	30.50	-326.5 to 387.5	No	ns	>0.9999			
4.5x109: Hs578T vs. 8.9x109:Hs578Ts(i)8	84.25	-272.7 to 441.2	No	ns	>0.9999			
4.5x109:Hs578Ts(i)8 vs. 6.7x109: Hs578T	14.75	-342.2 to 371.7	No	ns	>0.9999			
4.5x109:Hs578Ts(i)8 vs. 6.7x109:Hs578Ts(i)8	31.50	-325.5 to 388.5	No	ns	>0.9999			
4.5x109:Hs578Ts(i)8 vs. 8.9x109: Hs578T	28.25	-328.7 to 385.2	No	ns	>0.9999			

4.5x109:Hs578Ts(i)8 vs. 8.9x109:Hs578Ts(i)8	82.00	-275.0 to 439.0	No	ns	>0.9999			
6.7x109: Hs578T vs. 6.7x109:Hs578Ts(i)8	16.75	-340.2 to 373.7	No	ns	>0.9999			
6.7x109: Hs578T vs. 8.9x109: Hs578T	13.50	-343.5 to 370.5	No	ns	>0.9999			
6.7x109: Hs578T vs. 8.9x109:Hs578Ts(i)8	67.25	-289.7 to 424.2	No	ns	>0.9999			
6.7x109:Hs578Ts(i)8 vs. 8.9x109: Hs578T	-3.250	-360.2 to 353.7	No	ns	>0.9999			
6.7x109:Hs578Ts(i)8 vs. 8.9x109:Hs578Ts(i)8	50.50	-306.5 to 407.5	No	ns	>0.9999			
8.9x109: Hs578T vs. 8.9x109:Hs578Ts(i)8	53.75	-303.2 to 410.7	No	ns	>0.9999			
Test details	Mean 1	Mean 2	Mean Diff.	SE of diff.	N1	N2	q	DF
Control (PBS): Hs578T vs. Control (PBS):Hs578Ts(i)8	1800	1800	0.000	95.31	4	4	0.000	66.00
Control (PBS): Hs578T vs. 2.2x107: Hs578T	1800	1128	671.8	95.31	4	4	9.968	66.00
Control (PBS): Hs578T vs. 2.2x107:Hs578Ts(i)8	1800	1268	532.5	95.31	4	4	7.901	66.00
Control (PBS): Hs578T vs. 4.5x107: Hs578T	1800	868.0	932.0	95.31	4	4	13.83	66.00
Control (PBS): Hs578T vs. 4.5x107:Hs578Ts(i)8	1800	1012	788.0	95.31	4	4	11.69	66.00
Control (PBS): Hs578T vs. 1.1x108: Hs578T	1800	684.5	1116	95.31	4	4	16.55	66.00
Control (PBS): Hs578T vs. 1.1x108:Hs578Ts(i)8	1800	714.8	1085	95.31	4	4	16.10	66.00
Control (PBS): Hs578T vs. 2.2x108: Hs578T	1800	525.3	1275	95.31	4	4	18.92	66.00

Control (PBS): Hs578T vs. 2.2x108:Hs578Ts(i)8	1800	538.8	1261	95.31	4	4	18.71	66.00
Control (PBS): Hs578T vs. 4.4x108: Hs578T	1800	315.0	1485	95.31	4	4	22.04	66.00
Control (PBS): Hs578T vs. 4.4x108:Hs578Ts(i)8	1800	431.5	1369	95.31	4	4	20.31	66.00
Control (PBS): Hs578T vs. 11.1x108: Hs578T	1800	252.8	1547	95.31	4	4	22.96	66.00
Control (PBS): Hs578T vs. 11.1x108:Hs578Ts(i)8	1800	302.5	1498	95.31	4	4	22.22	66.00
Control (PBS): Hs578T vs. 22.3x108: Hs578T	1800	236.0	1564	95.31	4	4	23.21	66.00
Control (PBS): Hs578T vs. 22.3x108:Hs578Ts(i)8	1800	240.3	1560	95.31	4	4	23.14	66.00
Control (PBS): Hs578T vs. 4.5x109: Hs578T	1800	208.8	1591	95.31	4	4	23.61	66.00
Control (PBS): Hs578T vs. 4.5x109:Hs578Ts(i)8	1800	206.5	1594	95.31	4	4	23.65	66.00
Control (PBS): Hs578T vs. 6.7x109: Hs578T	1800	191.8	1608	95.31	4	4	23.86	66.00
Control (PBS): Hs578T vs. 6.7x109:Hs578Ts(i)8	1800	175.0	1625	95.31	4	4	24.11	66.00
Control (PBS): Hs578T vs. 8.9x109: Hs578T	1800	178.3	1622	95.31	4	4	24.06	66.00
Control (PBS): Hs578T vs. 8.9x109:Hs578Ts(i)8	1800	124.5	1676	95.31	4	4	24.86	66.00
Control (PBS):Hs578Ts(i)8 vs. 2.2x107: Hs578T	1800	1128	671.8	95.31	4	4	9.968	66.00
Control (PBS):Hs578Ts(i)8 vs. 2.2x107:Hs578Ts(i)8	1800	1268	532.5	95.31	4	4	7.901	66.00

Control (PBS):Hs578Ts(i)8 vs. 4.5x107: Hs578T	1800	868.0	932.0	95.31	4	4	13.83	66.00
Control (PBS):Hs578Ts(i)8 vs. 4.5x107:Hs578Ts(i)8	1800	1012	788.0	95.31	4	4	11.69	66.00
Control (PBS):Hs578Ts(i)8 vs. 1.1x108: Hs578T	1800	684.5	1116	95.31	4	4	16.55	66.00
Control (PBS):Hs578Ts(i)8 vs. 1.1x108:Hs578Ts(i)8	1800	714.8	1085	95.31	4	4	16.10	66.00
Control (PBS):Hs578Ts(i)8 vs. 2.2x108: Hs578T	1800	525.3	1275	95.31	4	4	18.92	66.00
Control (PBS):Hs578Ts(i)8 vs. 2.2x108:Hs578Ts(i)8	1800	538.8	1261	95.31	4	4	18.71	66.00
Control (PBS):Hs578Ts(i)8 vs. 4.4x108: Hs578T	1800	315.0	1485	95.31	4	4	22.04	66.00
Control (PBS):Hs578Ts(i)8 vs. 4.4x108:Hs578Ts(i)8	1800	431.5	1369	95.31	4	4	20.31	66.00
Control (PBS):Hs578Ts(i)8 vs. 11.1x108: Hs578T	1800	252.8	1547	95.31	4	4	22.96	66.00
Control (PBS):Hs578Ts(i)8 vs. 11.1x108:Hs578Ts(i)8	1800	302.5	1498	95.31	4	4	22.22	66.00
Control (PBS):Hs578Ts(i)8 vs. 22.3x108: Hs578T	1800	236.0	1564	95.31	4	4	23.21	66.00
Control (PBS):Hs578Ts(i)8 vs. 22.3x108:Hs578Ts(i)8	1800	240.3	1560	95.31	4	4	23.14	66.00

Control (PBS):Hs578Ts(i)8 vs. 4.5x109: Hs578T	1800	208.8	1591	95.31	4	4	23.61	66.00
Control (PBS):Hs578Ts(i)8 vs. 4.5x109:Hs578Ts(i)8	1800	206.5	1594	95.31	4	4	23.65	66.00
Control (PBS):Hs578Ts(i)8 vs. 6.7x109: Hs578T	1800	191.8	1608	95.31	4	4	23.86	66.00
Control (PBS):Hs578Ts(i)8 vs. 6.7x109:Hs578Ts(i)8	1800	175.0	1625	95.31	4	4	24.11	66.00
Control (PBS):Hs578Ts(i)8 vs. 8.9x109: Hs578T	1800	178.3	1622	95.31	4	4	24.06	66.00
Control (PBS):Hs578Ts(i)8 vs. 8.9x109:Hs578Ts(i)8	1800	124.5	1676	95.31	4	4	24.86	66.00
2.2x107: Hs578T vs. 2.2x107:Hs578Ts(i)8	1128	1268	-139.3	95.31	4	4	2.066	66.00
2.2x107: Hs578T vs. 4.5x107: Hs578T	1128	868.0	260.3	95.31	4	4	3.862	66.00
2.2x107: Hs578T vs. 4.5x107:Hs578Ts(i)8	1128	1012	116.3	95.31	4	4	1.725	66.00
2.2x107: Hs578T vs. 1.1x108: Hs578T	1128	684.5	443.8	95.31	4	4	6.585	66.00
2.2x107: Hs578T vs. 1.1x108:Hs578Ts(i)8	1128	714.8	413.5	95.31	4	4	6.136	66.00
2.2x107: Hs578T vs. 2.2x108: Hs578T	1128	525.3	603.0	95.31	4	4	8.948	66.00
2.2x107: Hs578T vs. 2.2x108:Hs578Ts(i)8	1128	538.8	589.5	95.31	4	4	8.747	66.00
2.2x107: Hs578T vs. 4.4x108: Hs578T	1128	315.0	813.3	95.31	4	4	12.07	66.00
2.2x107: Hs578T vs. 4.4x108:Hs578Ts(i)8	1128	431.5	696.8	95.31	4	4	10.34	66.00

2.2x107: Hs578T vs. 11.1x108: Hs578T	1128	252.8	875.5	95.31	4	4	12.99	66.00
2.2x107: Hs578T vs. 11.1x108:Hs578Ts(i)8	1128	302.5	825.8	95.31	4	4	12.25	66.00
2.2x107: Hs578T vs. 22.3x108: Hs578T	1128	236.0	892.3	95.31	4	4	13.24	66.00
2.2x107: Hs578T vs. 22.3x108:Hs578Ts(i)8	1128	240.3	888.0	95.31	4	4	13.18	66.00
2.2x107: Hs578T vs. 4.5x109: Hs578T	1128	208.8	919.5	95.31	4	4	13.64	66.00
2.2x107: Hs578T vs. 4.5x109:Hs578Ts(i)8	1128	206.5	921.8	95.31	4	4	13.68	66.00
2.2x107: Hs578T vs. 6.7x109: Hs578T	1128	191.8	936.5	95.31	4	4	13.90	66.00
2.2x107: Hs578T vs. 6.7x109:Hs578Ts(i)8	1128	175.0	953.3	95.31	4	4	14.14	66.00
2.2x107: Hs578T vs. 8.9x109: Hs578T	1128	178.3	950.0	95.31	4	4	14.10	66.00
2.2x107: Hs578T vs. 8.9x109:Hs578Ts(i)8	1128	124.5	1004	95.31	4	4	14.89	66.00
2.2x107:Hs578Ts(i)8 vs. 4.5x107: Hs578T	1268	868.0	399.5	95.31	4	4	5.928	66.00
2.2x107:Hs578Ts(i)8 vs. 4.5x107:Hs578Ts(i)8	1268	1012	255.5	95.31	4	4	3.791	66.00
2.2x107:Hs578Ts(i)8 vs. 1.1x108: Hs578T	1268	684.5	583.0	95.31	4	4	8.651	66.00
2.2x107:Hs578Ts(i)8 vs. 1.1x108:Hs578Ts(i)8	1268	714.8	552.8	95.31	4	4	8.202	66.00
2.2x107:Hs578Ts(i)8 vs. 2.2x108: Hs578T	1268	525.3	742.3	95.31	4	4	11.01	66.00
2.2x107:Hs578Ts(i)8 vs. 2.2x108:Hs578Ts(i)8	1268	538.8	728.8	95.31	4	4	10.81	66.00
2.2x107:Hs578Ts(i)8 vs. 4.4x108: Hs578T	1268	315.0	952.5	95.31	4	4	14.13	66.00
2.2x107:Hs578Ts(i)8 vs. 4.4x108:Hs578Ts(i)8	1268	431.5	836.0	95.31	4	4	12.40	66.00

2.2x107:Hs578Ts(i)8 vs. 11.1x108: Hs578T	1268	252.8	1015	95.31	4	4	15.06	66.00
2.2x107:Hs578Ts(i)8 vs. 11.1x108:Hs578Ts(i)8	1268	302.5	965.0	95.31	4	4	14.32	66.00
2.2x107:Hs578Ts(i)8 vs. 22.3x108: Hs578T	1268	236.0	1032	95.31	4	4	15.31	66.00
2.2x107:Hs578Ts(i)8 vs. 22.3x108:Hs578Ts(i)8	1268	240.3	1027	95.31	4	4	15.24	66.00
2.2x107:Hs578Ts(i)8 vs. 4.5x109: Hs578T	1268	208.8	1059	95.31	4	4	15.71	66.00
2.2x107:Hs578Ts(i)8 vs. 4.5x109:Hs578Ts(i)8	1268	206.5	1061	95.31	4	4	15.74	66.00
2.2x107:Hs578Ts(i)8 vs. 6.7x109: Hs578T	1268	191.8	1076	95.31	4	4	15.96	66.00
2.2x107:Hs578Ts(i)8 vs. 6.7x109:Hs578Ts(i)8	1268	175.0	1093	95.31	4	4	16.21	66.00
2.2x107:Hs578Ts(i)8 vs. 8.9x109: Hs578T	1268	178.3	1089	95.31	4	4	16.16	66.00
2.2x107:Hs578Ts(i)8 vs. 8.9x109:Hs578Ts(i)8	1268	124.5	1143	95.31	4	4	16.96	66.00
4.5x107: Hs578T vs. 4.5x107:Hs578Ts(i)8	868.0	1012	-144.0	95.31	4	4	2.137	66.00
4.5x107: Hs578T vs. 1.1x108: Hs578T	868.0	684.5	183.5	95.31	4	4	2.723	66.00
4.5x107: Hs578T vs. 1.1x108:Hs578Ts(i)8	868.0	714.8	153.3	95.31	4	4	2.274	66.00
4.5x107: Hs578T vs. 2.2x108: Hs578T	868.0	525.3	342.8	95.31	4	4	5.086	66.00
4.5x107: Hs578T vs. 2.2x108:Hs578Ts(i)8	868.0	538.8	329.3	95.31	4	4	4.886	66.00
4.5x107: Hs578T vs. 4.4x108: Hs578T	868.0	315.0	553.0	95.31	4	4	8.206	66.00
4.5x107: Hs578T vs. 4.4x108:Hs578Ts(i)8	868.0	431.5	436.5	95.31	4	4	6.477	66.00

4.5x107: Hs578T vs. 11.1x108: Hs578T	868.0	252.8	615.3	95.31	4	4	9.129	66.00
4.5x107: Hs578T vs. 11.1x108:Hs578Ts(i)8	868.0	302.5	565.5	95.31	4	4	8.391	66.00
4.5x107: Hs578T vs. 22.3x108: Hs578T	868.0	236.0	632.0	95.31	4	4	9.378	66.00
4.5x107: Hs578T vs. 22.3x108:Hs578Ts(i)8	868.0	240.3	627.8	95.31	4	4	9.315	66.00
4.5x107: Hs578T vs. 4.5x109: Hs578T	868.0	208.8	659.3	95.31	4	4	9.782	66.00
4.5x107: Hs578T vs. 4.5x109:Hs578Ts(i)8	868.0	206.5	661.5	95.31	4	4	9.816	66.00
4.5x107: Hs578T vs. 6.7x109: Hs578T	868.0	191.8	676.3	95.31	4	4	10.03	66.00
4.5x107: Hs578T vs. 6.7x109:Hs578Ts(i)8	868.0	175.0	693.0	95.31	4	4	10.28	66.00
4.5x107: Hs578T vs. 8.9x109: Hs578T	868.0	178.3	689.8	95.31	4	4	10.23	66.00
4.5x107: Hs578T vs. 8.9x109:Hs578Ts(i)8	868.0	124.5	743.5	95.31	4	4	11.03	66.00
4.5x107:Hs578Ts(i)8 vs. 1.1x108: Hs578T	1012	684.5	327.5	95.31	4	4	4.860	66.00
4.5x107:Hs578Ts(i)8 vs. 1.1x108:Hs578Ts(i)8	1012	714.8	297.3	95.31	4	4	4.411	66.00
4.5x107:Hs578Ts(i)8 vs. 2.2x108: Hs578T	1012	525.3	486.8	95.31	4	4	7.223	66.00
4.5x107:Hs578Ts(i)8 vs. 2.2x108:Hs578Ts(i)8	1012	538.8	473.3	95.31	4	4	7.022	66.00
4.5x107:Hs578Ts(i)8 vs. 4.4x108: Hs578T	1012	315.0	697.0	95.31	4	4	10.34	66.00
4.5x107:Hs578Ts(i)8 vs. 4.4x108:Hs578Ts(i)8	1012	431.5	580.5	95.31	4	4	8.614	66.00
4.5x107:Hs578Ts(i)8 vs. 11.1x108: Hs578T	1012	252.8	759.3	95.31	4	4	11.27	66.00

4.5x107:Hs578Ts(i)8 vs. 11.1x108:Hs578Ts(i)8	1012	302.5	709.5	95.31	4	4	10.53	66.00
4.5x107:Hs578Ts(i)8 vs. 22.3x108: Hs578T	1012	236.0	776.0	95.31	4	4	11.51	66.00
4.5x107:Hs578Ts(i)8 vs. 22.3x108:Hs578Ts(i)8	1012	240.3	771.8	95.31	4	4	11.45	66.00
4.5x107:Hs578Ts(i)8 vs. 4.5x109: Hs578T	1012	208.8	803.3	95.31	4	4	11.92	66.00
4.5x107:Hs578Ts(i)8 vs. 4.5x109:Hs578Ts(i)8	1012	206.5	805.5	95.31	4	4	11.95	66.00
4.5x107:Hs578Ts(i)8 vs. 6.7x109: Hs578T	1012	191.8	820.3	95.31	4	4	12.17	66.00
4.5x107:Hs578Ts(i)8 vs. 6.7x109:Hs578Ts(i)8	1012	175.0	837.0	95.31	4	4	12.42	66.00
4.5x107:Hs578Ts(i)8 vs. 8.9x109: Hs578T	1012	178.3	833.8	95.31	4	4	12.37	66.00
4.5x107:Hs578Ts(i)8 vs. 8.9x109:Hs578Ts(i)8	1012	124.5	887.5	95.31	4	4	13.17	66.00
1.1x108: Hs578T vs. 1.1x108:Hs578Ts(i)8	684.5	714.8	-30.25	95.31	4	4	0.4489	66.00
1.1x108: Hs578T vs. 2.2x108: Hs578T	684.5	525.3	159.3	95.31	4	4	2.363	66.00
1.1x108: Hs578T vs. 2.2x108:Hs578Ts(i)8	684.5	538.8	145.8	95.31	4	4	2.163	66.00
1.1x108: Hs578T vs. 4.4x108: Hs578T	684.5	315.0	369.5	95.31	4	4	5.483	66.00
1.1x108: Hs578T vs. 4.4x108:Hs578Ts(i)8	684.5	431.5	253.0	95.31	4	4	3.754	66.00
1.1x108: Hs578T vs. 11.1x108: Hs578T	684.5	252.8	431.8	95.31	4	4	6.406	66.00
1.1x108: Hs578T vs. 11.1x108:Hs578Ts(i)8	684.5	302.5	382.0	95.31	4	4	5.668	66.00
1.1x108: Hs578T vs. 22.3x108: Hs578T	684.5	236.0	448.5	95.31	4	4	6.655	66.00

1.1x108: Hs578T vs. 22.3x108:Hs578Ts(i)8	684.5	240.3	444.3	95.31	4	4	6.592	66.00
1.1x108: Hs578T vs. 4.5x109: Hs578T	684.5	208.8	475.8	95.31	4	4	7.059	66.00
1.1x108: Hs578T vs. 4.5x109:Hs578Ts(i)8	684.5	206.5	478.0	95.31	4	4	7.093	66.00
1.1x108: Hs578T vs. 6.7x109: Hs578T	684.5	191.8	492.8	95.31	4	4	7.312	66.00
1.1x108: Hs578T vs. 6.7x109:Hs578Ts(i)8	684.5	175.0	509.5	95.31	4	4	7.560	66.00
1.1x108: Hs578T vs. 8.9x109: Hs578T	684.5	178.3	506.3	95.31	4	4	7.512	66.00
1.1x108: Hs578T vs. 8.9x109:Hs578Ts(i)8	684.5	124.5	560.0	95.31	4	4	8.310	66.00
1.1x108:Hs578Ts(i)8 vs. 2.2x108: Hs578T	714.8	525.3	189.5	95.31	4	4	2.812	66.00
1.1x108:Hs578Ts(i)8 vs. 2.2x108:Hs578Ts(i)8	714.8	538.8	176.0	95.31	4	4	2.612	66.00
1.1x108:Hs578Ts(i)8 vs. 4.4x108: Hs578T	714.8	315.0	399.8	95.31	4	4	5.932	66.00
1.1x108:Hs578Ts(i)8 vs. 4.4x108:Hs578Ts(i)8	714.8	431.5	283.3	95.31	4	4	4.203	66.00
1.1x108:Hs578Ts(i)8 vs. 11.1x108: Hs578T	714.8	252.8	462.0	95.31	4	4	6.855	66.00
1.1x108:Hs578Ts(i)8 vs. 11.1x108:Hs578Ts(i)8	714.8	302.5	412.3	95.31	4	4	6.117	66.00
1.1x108:Hs578Ts(i)8 vs. 22.3x108: Hs578T	714.8	236.0	478.8	95.31	4	4	7.104	66.00
1.1x108:Hs578Ts(i)8 vs. 22.3x108:Hs578Ts(i)8	714.8	240.3	474.5	95.31	4	4	7.041	66.00
1.1x108:Hs578Ts(i)8 vs. 4.5x109: Hs578T	714.8	208.8	506.0	95.31	4	4	7.508	66.00
1.1x108:Hs578Ts(i)8 vs. 4.5x109:Hs578Ts(i)8	714.8	206.5	508.3	95.31	4	4	7.542	66.00

1.1x108:Hs578Ts(i)8 vs. 6.7x109: Hs578T	714.8	191.8	523.0	95.31	4	4	7.760	66.00
1.1x108:Hs578Ts(i)8 vs. 6.7x109:Hs578Ts(i)8	714.8	175.0	539.8	95.31	4	4	8.009	66.00
1.1x108:Hs578Ts(i)8 vs. 8.9x109: Hs578T	714.8	178.3	536.5	95.31	4	4	7.961	66.00
1.1x108:Hs578Ts(i)8 vs. 8.9x109:Hs578Ts(i)8	714.8	124.5	590.3	95.31	4	4	8.758	66.00
2.2x108: Hs578T vs. 2.2x108:Hs578Ts(i)8	525.3	538.8	-13.50	95.31	4	4	0.2003	66.00
2.2x108: Hs578T vs. 4.4x108: Hs578T	525.3	315.0	210.3	95.31	4	4	3.120	66.00
2.2x108: Hs578T vs. 4.4x108:Hs578Ts(i)8	525.3	431.5	93.75	95.31	4	4	1.391	66.00
2.2x108: Hs578T vs. 11.1x108: Hs578T	525.3	252.8	272.5	95.31	4	4	4.043	66.00
2.2x108: Hs578T vs. 11.1x108:Hs578Ts(i)8	525.3	302.5	222.8	95.31	4	4	3.305	66.00
2.2x108: Hs578T vs. 22.3x108: Hs578T	525.3	236.0	289.3	95.31	4	4	4.292	66.00
2.2x108: Hs578T vs. 22.3x108:Hs578Ts(i)8	525.3	240.3	285.0	95.31	4	4	4.229	66.00
2.2x108: Hs578T vs. 4.5x109: Hs578T	525.3	208.8	316.5	95.31	4	4	4.696	66.00
2.2x108: Hs578T vs. 4.5x109:Hs578Ts(i)8	525.3	206.5	318.8	95.31	4	4	4.730	66.00
2.2x108: Hs578T vs. 6.7x109: Hs578T	525.3	191.8	333.5	95.31	4	4	4.949	66.00
2.2x108: Hs578T vs. 6.7x109:Hs578Ts(i)8	525.3	175.0	350.3	95.31	4	4	5.197	66.00
2.2x108: Hs578T vs. 8.9x109: Hs578T	525.3	178.3	347.0	95.31	4	4	5.149	66.00
2.2x108: Hs578T vs. 8.9x109:Hs578Ts(i)8	525.3	124.5	400.8	95.31	4	4	5.946	66.00
2.2x108:Hs578Ts(i)8 vs. 4.4x108: Hs578T	538.8	315.0	223.8	95.31	4	4	3.320	66.00

2.2x108:Hs578Ts(i)8 vs. 4.4x108:Hs578Ts(i)8	538.8	431.5	107.3	95.31	4	4	1.591	66.00
2.2x108:Hs578Ts(i)8 vs. 11.1x108: Hs578T	538.8	252.8	286.0	95.31	4	4	4.244	66.00
2.2x108:Hs578Ts(i)8 vs. 11.1x108:Hs578Ts(i)8	538.8	302.5	236.3	95.31	4	4	3.506	66.00
2.2x108:Hs578Ts(i)8 vs. 22.3x108: Hs578T	538.8	236.0	302.8	95.31	4	4	4.492	66.00
2.2x108:Hs578Ts(i)8 vs. 22.3x108:Hs578Ts(i)8	538.8	240.3	298.5	95.31	4	4	4.429	66.00
2.2x108:Hs578Ts(i)8 vs. 4.5x109: Hs578T	538.8	208.8	330.0	95.31	4	4	4.897	66.00
2.2x108:Hs578Ts(i)8 vs. 4.5x109:Hs578Ts(i)8	538.8	206.5	332.3	95.31	4	4	4.930	66.00
2.2x108:Hs578Ts(i)8 vs. 6.7x109: Hs578T	538.8	191.8	347.0	95.31	4	4	5.149	66.00
2.2x108:Hs578Ts(i)8 vs. 6.7x109:Hs578Ts(i)8	538.8	175.0	363.8	95.31	4	4	5.397	66.00
2.2x108:Hs578Ts(i)8 vs. 8.9x109: Hs578T	538.8	178.3	360.5	95.31	4	4	5.349	66.00
2.2x108:Hs578Ts(i)8 vs. 8.9x109:Hs578Ts(i)8	538.8	124.5	414.3	95.31	4	4	6.147	66.00
4.4x108: Hs578T vs. 4.4x108:Hs578Ts(i)8	315.0	431.5	-116.5	95.31	4	4	1.729	66.00
4.4x108: Hs578T vs. 11.1x108: Hs578T	315.0	252.8	62.25	95.31	4	4	0.9237	66.00
4.4x108: Hs578T vs. 11.1x108:Hs578Ts(i)8	315.0	302.5	12.50	95.31	4	4	0.1855	66.00
4.4x108: Hs578T vs. 22.3x108: Hs578T	315.0	236.0	79.00	95.31	4	4	1.172	66.00
4.4x108: Hs578T vs. 22.3x108:Hs578Ts(i)8	315.0	240.3	74.75	95.31	4	4	1.109	66.00
4.4x108: Hs578T vs. 4.5x109: Hs578T	315.0	208.8	106.3	95.31	4	4	1.577	66.00

4.4x108: Hs578T vs. 4.5x109:Hs578Ts(i)8	315.0	206.5	108.5	95.31	4	4	1.610	66.00
4.4x108: Hs578T vs. 6.7x109: Hs578T	315.0	191.8	123.3	95.31	4	4	1.829	66.00
4.4x108: Hs578T vs. 6.7x109:Hs578Ts(i)8	315.0	175.0	140.0	95.31	4	4	2.077	66.00
4.4x108: Hs578T vs. 8.9x109: Hs578T	315.0	178.3	136.8	95.31	4	4	2.029	66.00
4.4x108: Hs578T vs. 8.9x109:Hs578Ts(i)8	315.0	124.5	190.5	95.31	4	4	2.827	66.00
4.4x108:Hs578Ts(i)8 vs. 11.1x108: Hs578T	431.5	252.8	178.8	95.31	4	4	2.652	66.00
4.4x108:Hs578Ts(i)8 vs. 11.1x108:Hs578Ts(i)8	431.5	302.5	129.0	95.31	4	4	1.914	66.00
4.4x108:Hs578Ts(i)8 vs. 22.3x108: Hs578T	431.5	236.0	195.5	95.31	4	4	2.901	66.00
4.4x108:Hs578Ts(i)8 vs. 22.3x108:Hs578Ts(i)8	431.5	240.3	191.3	95.31	4	4	2.838	66.00
4.4x108:Hs578Ts(i)8 vs. 4.5x109: Hs578T	431.5	208.8	222.8	95.31	4	4	3.305	66.00
4.4x108:Hs578Ts(i)8 vs. 4.5x109:Hs578Ts(i)8	431.5	206.5	225.0	95.31	4	4	3.339	66.00
4.4x108:Hs578Ts(i)8 vs. 6.7x109: Hs578T	431.5	191.8	239.8	95.31	4	4	3.558	66.00
4.4x108:Hs578Ts(i)8 vs. 6.7x109:Hs578Ts(i)8	431.5	175.0	256.5	95.31	4	4	3.806	66.00
4.4x108:Hs578Ts(i)8 vs. 8.9x109: Hs578T	431.5	178.3	253.3	95.31	4	4	3.758	66.00
4.4x108:Hs578Ts(i)8 vs. 8.9x109:Hs578Ts(i)8	431.5	124.5	307.0	95.31	4	4	4.555	66.00
11.1x108: Hs578T vs. 11.1x108:Hs578Ts(i)8	252.8	302.5	-49.75	95.31	4	4	0.7382	66.00
11.1x108: Hs578T vs. 22.3x108: Hs578T	252.8	236.0	16.75	95.31	4	4	0.2485	66.00

11.1x108: Hs578T vs. 22.3x108:Hs578Ts(i)8	252.8	240.3	12.50	95.31	4	4	0.1855	66.00
11.1x108: Hs578T vs. 4.5x109: Hs578T	252.8	208.8	44.00	95.31	4	4	0.6529	66.00
11.1x108: Hs578T vs. 4.5x109:Hs578Ts(i)8	252.8	206.5	46.25	95.31	4	4	0.6863	66.00
11.1x108: Hs578T vs. 6.7x109: Hs578T	252.8	191.8	61.00	95.31	4	4	0.9051	66.00
11.1x108: Hs578T vs. 6.7x109:Hs578Ts(i)8	252.8	175.0	77.75	95.31	4	4	1.154	66.00
11.1x108: Hs578T vs. 8.9x109: Hs578T	252.8	178.3	74.50	95.31	4	4	1.105	66.00
11.1x108: Hs578T vs. 8.9x109:Hs578Ts(i)8	252.8	124.5	128.3	95.31	4	4	1.903	66.00
11.1x108:Hs578Ts(i)8 vs. 22.3x108: Hs578T	302.5	236.0	66.50	95.31	4	4	0.9868	66.00
11.1x108:Hs578Ts(i)8 vs. 22.3x108:Hs578Ts(i)8	302.5	240.3	62.25	95.31	4	4	0.9237	66.00
11.1x108:Hs578Ts(i)8 vs. 4.5x109: Hs578T	302.5	208.8	93.75	95.31	4	4	1.391	66.00
11.1x108:Hs578Ts(i)8 vs. 4.5x109:Hs578Ts(i)8	302.5	206.5	96.00	95.31	4	4	1.424	66.00
11.1x108:Hs578Ts(i)8 vs. 6.7x109: Hs578T	302.5	191.8	110.8	95.31	4	4	1.643	66.00
11.1x108:Hs578Ts(i)8 vs. 6.7x109:Hs578Ts(i)8	302.5	175.0	127.5	95.31	4	4	1.892	66.00
11.1x108:Hs578Ts(i)8 vs. 8.9x109: Hs578T	302.5	178.3	124.3	95.31	4	4	1.844	66.00
11.1x108:Hs578Ts(i)8 vs. 8.9x109:Hs578Ts(i)8	302.5	124.5	178.0	95.31	4	4	2.641	66.00
22.3x108: Hs578T vs. 22.3x108:Hs578Ts(i)8	236.0	240.3	-4.250	95.31	4	4	0.06306	66.00
22.3x108: Hs578T vs. 4.5x109: Hs578T	236.0	208.8	27.25	95.31	4	4	0.4043	66.00

22.3x108: Hs578T vs. 4.5x109:Hs578Ts(i)8	236.0	206.5	29.50	95.31	4	4	0.4377	66.00
22.3x108: Hs578T vs. 6.7x109: Hs578T	236.0	191.8	44.25	95.31	4	4	0.6566	66.00
22.3x108: Hs578T vs. 6.7x109:Hs578Ts(i)8	236.0	175.0	61.00	95.31	4	4	0.9051	66.00
22.3x108: Hs578T vs. 8.9x109: Hs578T	236.0	178.3	57.75	95.31	4	4	0.8569	66.00
22.3x108: Hs578T vs. 8.9x109:Hs578Ts(i)8	236.0	124.5	111.5	95.31	4	4	1.654	66.00
22.3x108:Hs578Ts(i)8 vs. 4.5x109: Hs578T	240.3	208.8	31.50	95.31	4	4	0.4674	66.00
22.3x108:Hs578Ts(i)8 vs. 4.5x109:Hs578Ts(i)8	240.3	206.5	33.75	95.31	4	4	0.5008	66.00
22.3x108:Hs578Ts(i)8 vs. 6.7x109: Hs578T	240.3	191.8	48.50	95.31	4	4	0.7197	66.00
22.3x108:Hs578Ts(i)8 vs. 6.7x109:Hs578Ts(i)8	240.3	175.0	65.25	95.31	4	4	0.9682	66.00
22.3x108:Hs578Ts(i)8 vs. 8.9x109: Hs578T	240.3	178.3	62.00	95.31	4	4	0.9200	66.00
22.3x108:Hs578Ts(i)8 vs. 8.9x109:Hs578Ts(i)8	240.3	124.5	115.8	95.31	4	4	1.718	66.00
4.5x109: Hs578T vs. 4.5x109:Hs578Ts(i)8	208.8	206.5	2.250	95.31	4	4	0.03339	66.00
4.5x109: Hs578T vs. 6.7x109: Hs578T	208.8	191.8	17.00	95.31	4	4	0.2523	66.00
4.5x109: Hs578T vs. 6.7x109:Hs578Ts(i)8	208.8	175.0	33.75	95.31	4	4	0.5008	66.00
4.5x109: Hs578T vs. 8.9x109: Hs578T	208.8	178.3	30.50	95.31	4	4	0.4526	66.00
4.5x109: Hs578T vs. 8.9x109:Hs578Ts(i)8	208.8	124.5	84.25	95.31	4	4	1.250	66.00
4.5x109:Hs578Ts(i)8 vs. 6.7x109: Hs578T	206.5	191.8	14.75	95.31	4	4	0.2189	66.00
4.5x109:Hs578Ts(i)8 vs. 6.7x109:Hs578Ts(i)8	206.5	175.0	31.50	95.31	4	4	0.4674	66.00

4.5x109:Hs578Ts(i)8 vs. 8.9x109: Hs578T	206.5	178.3	28.25	95.31	4	4	0.4192	66.00
4.5x109:Hs578Ts(i)8 vs. 8.9x109:Hs578Ts(i)8	206.5	124.5	82.00	95.31	4	4	1.217	66.00
6.7x109: Hs578T vs. 6.7x109:Hs578Ts(i)8	191.8	175.0	16.75	95.31	4	4	0.2485	66.00
6.7x109: Hs578T vs. 8.9x109: Hs578T	191.8	178.3	13.50	95.31	4	4	0.2003	66.00
6.7x109: Hs578T vs. 8.9x109:Hs578Ts(i)8	191.8	124.5	67.25	95.31	4	4	0.9979	66.00
6.7x109:Hs578Ts(i)8 vs. 8.9x109: Hs578T	175.0	178.3	-3.250	95.31	4	4	0.04822	66.00
6.7x109:Hs578Ts(i)8 vs. 8.9x109:Hs578Ts(i)8	175.0	124.5	50.50	95.31	4	4	0.7493	66.00
8.9x109: Hs578T vs. 8.9x109:Hs578Ts(i)8	178.3	124.5	53.75	95.31	4	4	0.7976	66.00

Supplement to Figure 4: All proteins identified by Proteomic Profiling

LFQ intensity Hs578T EVs n=1	LFQ intensity Hs578T EVs n=2	LFQ intensity Hs578T EVs n=3	LFQ intensity Hs578Ts(i) 8 EVs n=1	LFQ intensity Hs578Ts(i) 8 EVs n=2	LFQ intensity Hs578Ts(i) 8 EVs n=3	N: Sequence coverage [%]	N: Mol. weight [kDa]	T: UNIPROT	T: Protein names	T: id	T: GENE
26.2752	27.9191	26.8445	24.1644	NaN	24.4885	15.6	100.56	A1L4H1-2	Isoform 2 of Soluble scavenger receptor cysteine-rich domain-containing protein SSC5D	12	SSC5D
27.1815	27.4736	27.5678	27.9174	27.7079	27.8081	13.1	104.1	Q99613-2	Isoform 2 of Eukaryotic translation initiation factor 3 subunit C	15	EIF3C
26.1543	25.9314	25.6807	25.4349	26.475	26.2877	10.7	23.063	B9A064	Immunoglobulin lambda-like polypeptide 5	16	IGLL5
28.6344	28.9125	28.9891	29.2887	29.0238	29.4452	41.7	47.463	O00231	26S proteasome non-ATPase regulatory subunit 11	21	PSMD11
27.5666	27.4699	27.3283	27.6709	27.6707	27.9365	21.1	52.904	O00232	26S proteasome non-ATPase regulatory subunit 12	22	PSMD12
29.7113	29.6327	29.9758	30.2275	29.7077	29.5976	63.1	26.922	O00299	Chloride intracellular channel protein 1	23	CLIC1
26.2602	26.4258	26.5518	26.5652	26.7334	26.748	15.1	37.563	O00303	Eukaryotic translation initiation factor 3 subunit F	24	EIF3F
25.965	25.3582	25.3977	25.2459	25.3097	25.3878	3.9	30.653	B8ZZF7	Sulfotransferase	25	SULT1C2
27.2309	26.199	26.6353	27.1649	25.9605	25.5542	15.6	66.86	O00391-2	Isoform 2 of Sulfhydryl oxidase 1	26	QSOX1
27.9788	27.6076	28.1462	27.9806	28.2617	28.0339	20	78.099	O00429-4	Isoform 3 of Dynamin-1-like protein	27	DNM1L
29.2822	28.8268	29.1598	29.6312	29.5266	29.3689	10.5	190.68	O00443	Phosphatidylinositol 4-phosphate 3-kinase C2 domain-containing subunit alpha	28	PIK3C2A
32.5674	32.7871	32.2523	31.9321	31.6519	32.427	44.8	214.84	O00468-6	Isoform 6 of Agrin	29	AGRN

27.2737	27.3902	27.5515	27.8565	27.1259	27.9922	24.5	34.577	O00487	26S proteasome non-ATPase regulatory subunit 14	31	PSMD14
28.1665	28.0234	28.1369	28.3044	28.1034	27.5707	30.1	31.761	O00560-3	Isoform 3 of Syntenin-1	32	SDCBP
26.9184	26.5591	26.8684	26.7242	26.6204	26.7188	11.4	66.049	O00567	Nucleolar protein 56	33	NOP56
NaN	23.9342	24.0266	26.7443	26.1328	25.7806	3.8	55.385	O00592-2	Isoform 2 of Podocalyxin	34	PODXL
29.1411	28.8212	29.7625	27.889	28.0284	29.0576	32.5	42.026	O00622	Protein CYR61	35	CYR61
27.3317	27.2463	27.3737	27.1576	26.7205	26.3839	25.9	37.978	O14657	Torsin-1B	38	TOR1B
30.7839	30.6844	30.505	30.3996	30.3283	30.0062	30.1	84.141	O14672	Disintegrin and metalloproteinase domain-containing protein 10	39	ADAM10
25.4415	25.6539	25.9335	25.5803	25.7116	25.3996	8.4	71.319	O14744-2	Isoform 2 of Protein arginine N-methyltransferase 5	40	PRMT5
29.0095	29.4421	29.05	28.7627	28.5686	28.814	23.3	101.3	E9PEP6	Neuropilin	41	NRP1
29.7875	30.5629	30.3874	29.3472	29.3765	29.6764	48.5	15.195	E9PN41	Tetraspanin-4 (Fragment)	42	TSPAN4
27.223	27.7901	27.0958	27.8142	27.3351	28.0888	28.2	27.887	O14818	Proteasome subunit alpha type-7	43	PSMA7
28.8178	28.864	28.5489	28.5257	28.5505	28.8969	22.8	38.287	O14828	Secretory carrier-associated membrane protein 3	44	SCAMP3
27.4357	27.8344	27.3772	27.6788	27.8412	27.6566	32.7	19.794	P19105	Myosin regulatory light chain 12A	46	MYL12A
27.76	27.7093	28.0669	28.0485	27.4718	27.2263	12.9	123.38	O14980	Exportin-1	48	XPO1
25.2701	25.4341	25.2955	26.0801	25.3343	25.5846	2.4	205.12	O15031	Plexin-B2	50	PLXNB2
NaN	26.0796	NaN	26.0197	25.9846	25.917	17.5	22.911	A0A087WU 14	Secretory carrier-associated membrane protein (Fragment)	53	SCAMP1
27.21	26.2427	26.9289	27.5633	26.2843	26.4899	13.2	40.949	O15143	Actin-related protein 2/3 complex subunit 1B	55	ARPC1B
27.2715	27.0408	27.1947	27.3648	27.2493	26.3281	27.3	34.333	O15144	Actin-related protein 2/3 complex subunit 2	56	ARPC2
31.7855	32.0809	31.5102	30.7396	30.3879	30.9907	33.3	399.73	O15230	Laminin subunit alpha-5	58	LAMA5
NaN	NaN	NaN	25.3003	24.4778	24.281	6	58.14	O15371-2	Isoform 2 of Eukaryotic translation initiation factor 3 subunit D	59	EIF3D
25.0237	25.0582	25.3311	25.2936	24.5	24.5264	3.9	60.632	O15460-2	Isoform IIa of Prolyl 4-hydroxylase subunit alpha-2	60	P4HA2

27.3846	27.0952	27.3218	27.5516	27.1702	27.0586	9.9	90.932	O43143	Pre-mRNA-splicing factor ATP-dependent RNA helicase DHX15	61	DHX15
29.4585	28.7603	29.3534	29.3611	29.4043	29.7575	27.6	55.938	A0A286YF22	D-3-phosphoglycerate dehydrogenase	64	PHGDH
28.1788	28.2729	28.2732	28.0466	28.6325	28.6587	24.2	60.977	O43242	26S proteasome non-ATPase regulatory subunit 3	65	PSMD3
24.7244	24.9128	24.9716	25.3392	25.5879	25.7764	16.1	19.81	O43324	Eukaryotic translation elongation factor 1 epsilon-1	68	EEF1E1
26.5023	26.673	26.8767	27.506	27.173	26.6634	27.4	34.684	H3BLU7	Aflatoxin B1 aldehyde reductase member 2 (Fragment)	69	AKR7A2
29.1055	29.0775	28.7901	28.3264	28.453	28.1349	33.6	15.609	A0A087WZU5	Tetraspanin-6	70	TSPAN6
26.73	26.6064	26.0668	27.2755	26.7928	26.7229	22.3	31.703	J3QT28	Mitotic checkpoint protein BUB3 (Fragment)	71	BUB3
NaN	24.9044	25.0759	24.9691	25.3665	25.6009	6.3	91.35	O43747	AP-1 complex subunit gamma-1	72	AP1G1
28.3776	27.8691	28.4162	29.0008	28.5817	28.257	25.4	62.942	O43776	Asparagine--tRNA ligase, cytoplasmic	74	NARS
25.7497	25.918	25.9421	NaN	NaN	NaN	28.1	22.042	E9PHS0	Glutathione S-transferase LANCL1 (Fragment)	75	LANCL1
35.9949	36.2678	35.8717	35.6972	35.7327	36.0405	65.5	52.747	O43854-2	Isoform 2 of EGF-like repeat and discoidin I-like domain-containing protein 3	76	EDIL3
26.4244	26.38	26.6492	26.5236	26.5385	26.5274	9.1	58.735	O60506-4	Isoform 4 of Heterogeneous nuclear ribonucleoprotein Q	79	SYNCRIP
27.6089	27.2943	27.4243	26.9102	25.9347	26.8514	13.4	84.784	O60568	Multifunctional procollagen lysine hydroxylase and glycosyltransferase LH3	80	PLOD3
25.3925	25.2523	26.0511	25.8315	25.4348	25.5402	8	60.029	O60684	Importin subunit alpha-7	81	KPNA6
28.3126	28.4219	28.7446	29.017	29.1501	29.0586	36.2	55.023	O60701	UDP-glucose 6-dehydrogenase	82	UGDH
27.985	27.9464	27.8794	28.2664	27.7813	27.7307	13.4	107.89	O60763	General vesicular transport factor p115	83	USO1
33.59	33.6323	33.5033	33.4815	34.1056	34.1215	46.8	13.952	Q99880	Histone H2B type 1-L	84	HIST1H2BL

NaN	24.5508	24.5449	26.1152	24.9941	25.3749	43.4	8.5829	H7BZF2	H/ACA ribonucleoprotein complex subunit DKC1 (Fragment)	85	DKC1
31.1613	31.2852	31.3877	31.5461	31.1399	30.8034	42.6	66.193	O75083	WD repeat-containing protein 1	88	WDR1
27.9187	27.2242	27.8727	28.0089	28.0158	28.5197	25.7	60.13	O75131	Copine-3	89	CPNE3
26.9824	27.2128	27.075	27.4738	27.9001	27.6979	6.8	254.41	O75165	DnaJ homolog subfamily C member 13	90	DNAJC13
NaN	24.8388	25.0889	25.0197	24.8721	25.1643	8.8	43.181	O75191-2	Isoform 2 of Xylulose kinase	91	XYLB
25.9135	25.4682	25.9652	26.1108	26.3454	26.4774	26.7	21.868	O75340	Programmed cell death protein 6	92	PDCD6
31.0859	30.9347	30.9823	31.1517	31.5383	31.5274	43.1	39.183	O75367-2	Isoform 1 of Core histone macro-H2A.1	94	H2AFY
25.9371	25.888	26.0492	26.1537	26.2741	25.6887	4.8	230.29	O75369-5	Isoform 5 of Filamin-B	95	FLNB
27.3872	27.0732	27.2831	27.7771	27.0599	27.575	13.8	44.703	A0A0C4DGI3	Citrate synthase	96	CS
30.3862	30.0775	29.7897	29.4482	29.9544	30.2936	50.6	10.058	O75531	Barrier-to-autointegration factor	98	BANF1
26.0357	26.1381	26.1755	26.5868	26.1467	25.8345	5.5	145.83	O75533	Splicing factor 3B subunit 1	99	SF3B1
27.6055	27.5841	27.6895	28.2893	27.5772	27.4838	11.5	244.5	O75643	U5 small nuclear ribonucleoprotein 200 kDa helicase	101	SNRNP200
30.1676	30.1111	30.3477	29.232	28.813	28.5761	35.9	46.561	O75718	Cartilage-associated protein	102	CRTAP
NaN	24.897	24.7109	24.8596	24.6684	24.4091	17.3	20.217	B1AJY5	26S proteasome non-ATPase regulatory subunit 10	105	PSMD10
27.8608	28.0378	27.615	27.7524	27.4253	26.8713	20.3	46.659	O75874	Isocitrate dehydrogenase [NADP] cytoplasmic	106	IDH1
28.8398	29.1783	29.1415	27.8758	28.3487	28.4404	45	14.846	A0A3B3IST1	Tetraspanin-9 (Fragment)	110	TSPAN9
25.5871	25.3745	25.4814	26.6638	25.9522	26.7421	19	18.535	I3L3C4	Ribosomal L1 domain-containing protein 1 (Fragment)	111	RSL1D1
24.7053	24.422	24.3822	24.3803	24.938	24.5429	22	13.775	H0YB13	Stanniocalcin-2 (Fragment)	113	STC2
26.5851	25.8462	25.8489	26.8286	26.3255	26.1339	6.8	93.629	G5E9C5	Phosphodiesterase	114	PDE5A
26.3315	26.2106	26.5997	26.7071	26.8517	27.1608	9	96.502	D6RHZ5	Protein transport protein Sec31A	116	SEC31A

32.3925	32.3379	32.3024	31.4322	31.7195	31.8017	41.6	39.641	O95084-2	Isoform 2 of Serine protease 23	117	PRSS23
26.7104	26.4068	26.5269	26.6814	27.4054	27.128	11	25.609	O95197-3	Isoform 3 of Reticulon-3	119	RTN3
24.5227	24.3425	24.7118	NaN	NaN	NaN	15.8	20.677	D6RDI2	Luc7-like protein 3 (Fragment)	120	LUC7L3
27.4788	27.3977	26.9721	NaN	NaN	NaN	12	20.23	Q9UEL6	Myelin protein zero-like protein 1 (Fragment)	121	MPZL1
27.9734	27.8592	28.2146	28.0894	27.4486	27.138	11.2	119.52	O95373	Importin-7	123	IPO7
28.9391	28.5715	28.7438	28.7991	28.6895	28.5134	12.1	13.051	O95445-2	Isoform 2 of Apolipoprotein M	124	APOM
29.8481	29.8994	29.9768	30.085	29.7026	28.965	22.2	70.678	O95834	Echinoderm microtubule-associated protein-like 2	128	EML2
28.7569	28.089	27.8316	26.5985	NaN	26.8646	28	48.191	E9PRU1	EGF-containing fibulin-like extracellular matrix protein 2	129	EFEMP2
25.3696	25.1691	25.206	26.6071	25.4389	25.0522	13.7	43.236	O96019-2	Isoform 2 of Actin-like protein 6A	130	ACTL6A
30.0637	29.7759	30.0579	30.0641	30.6886	30.6809	32.8	36.688	P00338	L-lactate dehydrogenase A chain	131	LDHA
26.3672	26.5108	26.4462	27.0341	26.5721	26.0872	11.6	54.861	P00352	Retinal dehydrogenase 1	132	ALDH1A1
26.8724	26.5951	26.5443	27.3649	27.3266	26.6375	5.7	122.2	P00450	Ceruloplasmin	135	CP
29.6916	29.516	29.6886	30.2563	29.0725	28.728	6.6	83.266	P00488	Coagulation factor XIII A chain	136	F13A1
27.3407	27.4726	27.1952	27.2761	26.9559	27.4286	15.6	69.227	P00533-4	Isoform 4 of Epidermal growth factor receptor	138	EGFR
31.3302	31.3569	31.4319	31.693	31.5315	31.1658	48.9	44.614	P00558	Phosphoglycerate kinase 1	139	PGK1
33.8217	33.973	34.0479	34.1331	34.1431	34.0486	16.3	65.408	E9PIT3	Prothrombin	140	F2
24.9058	NaN	NaN	25.5021	25.6817	26.3686	7	76.613	F5H2D0	Complement C1r subcomponent	141	C1R
27.5573	26.3729	26.1051	27.6253	28.5844	28.5659	15.3	38.451	A0A0C4DGL8	Haptoglobin	142	HP
27.3926	27.5868	27.5165	28.1609	27.7204	27.9879	5.9	54.731	P00742	Coagulation factor X	143	F10
29.4042	29.1886	29.0521	29.0381	29.4722	29.4182	7.7	90.568	P00747	Plasminogen	144	PLG
28.6669	28.7443	28.7813	29.2023	29.157	28.973	26.2	46.53	P00966	Argininosuccinate synthase	146	ASS1
31.694	31.2942	31.5396	31.2686	31.6631	31.599	8.4	52.602	P01008	Antithrombin-III	147	SERPINC1

24.1233	23.9345	24.1307	NaN	26.2992	24.3255	6.9	47.65	P01011	Alpha-1-antichymotrypsin	148	SERPINA3
33.3455	33.1745	32.3165	33.5592	33.7026	34.3161	4.7	163.29	P01023	Alpha-2-macroglobulin	149	A2M
31.3122	30.7214	30.8911	31.1759	31.3218	31.7603	5.8	187.15	P01024	Complement C3	150	C3
27.6617	27.7176	27.4222	27.6952	27.6479	28.183	2.2	188.3	P01031	Complement C5	151	C5
27.6827	27.7149	28.6594	27.6671	28.4094	27.9297	2.3	43.821	P01042-3	Isoform 3 of Kininogen-1	152	KNG1
27.0068	28.4482	28.2192	27.8994	NaN	NaN	21.4	10.959	A6XGL2	Insulin	153	INS
NaN	26.0691	26.1035	25.4364	27.8995	26.5405	18	31.983	A0A0A0MS07	Immunoglobulin heavy constant gamma 1 (Fragment)	157	IGHG1
27.8223	28.6047	28.7728	28.3722	28.7404	28.224	18.4	16.203	P02100	Hemoglobin subunit epsilon	158	HBE1
31.6785	32.6216	31.7455	28.9836	28.2388	29.0012	34.4	138.94	P02452	Collagen alpha-1(I) chain	159	COL1A1
28.8837	28.8777	28.9032	25.9132	25.9573	27.0583	11.9	138.56	P02461	Collagen alpha-1(III) chain	160	COL3A1
26.1979	25.63	25.3795	26.8609	26.3246	26.9675	10.1	39.731	A0A3B3ISV3	Collagen alpha-1(IV) chain (Fragment)	161	COL4A1
26.2785	NaN	28.055	26.1526	27.4494	25.693	23.1	51.561	P02533	Keratin, type I cytoskeletal 14	163	KRT14
30.2605	30.3834	30.243	29.677	32.0731	30.6482	51.7	30.777	P02647	Apolipoprotein A-I	166	APOA1
31.3279	32.0873	31.1021	31.4011	31.1535	31.1452	24.9	36.154	P02649	Apolipoprotein E	167	APOE
26.5793	27.567	26.6937	26.8657	26.7434	27.1234	16.2	10.852	P02656	Apolipoprotein C-III	168	APOC3
27.7341	25.7611	25.7269	28.0604	28.4467	28.3457	7.8	49.496	P02679-2	Isoform Gamma-A of Fibrinogen gamma chain	171	FGG
25.3156	25.4046	25.3601	24.74	27.6283	26.1633	34.7	10.811	E9PMR5	Myelin basic protein	172	MBP
28.3551	27.5295	27.1064	27.7662	29.0054	28.8022	3.8	63.173	P02748	Complement component C9	173	C9
26.9346	26.9334	26.5729	28.6494	28.0352	27.1597	8.7	11.552	J3KS17	Beta-2-glycoprotein 1 (Fragment)	174	APOH
36.55	36.5894	36.2847	36.2501	35.9602	36.4148	56.7	262.62	P02751	Fibronectin	175	FN1
27.1433	NaN	26.9419	26.1205	27.1543	26.891	10.1	22.974	Q5VY30	Retinol-binding protein	176	RBP4
32.9923	31.9582	32.4916	31.3202	32.1996	32.0642	8.2	39.34	P02765	Alpha-2-HS-glycoprotein	177	AHSG
34.5319	34.2779	34.3227	33.6935	35.7019	34.7495	54	69.366	P02768	Serum albumin	178	ALB
29.4339	30.9169	30.9061	30.5806	30.9296	31.1473	6.7	68.677	P02771	Alpha-fetoprotein	179	AFP
31.3057	31.5532	31.6004	31.044	31.412	31.0549	12	52.917	P02774	Vitamin D-binding protein	180	GC
29.3568	29.4308	29.3954	30.7638	30.4762	30.5622	25.5	84.87	P02786	Transferrin receptor protein 1	181	TFRC
31.4096	31.0795	31.1591	30.3193	31.7743	31.305	4.5	73.16	P02788-2	Isoform DeltaLf of Lactotransferrin	183	LTF

26.9436	26.5729	26.7984	26.1785	28.2547	27.0303	14.5	51.676	P02790	Hemopexin	184	HPX
27.764	26.5247	26.1066	26.5563	28.2114	27.2352	31.6	11.168	E9PRK8	Ferritin	185	FTH1
30.6236	30.2793	30.4727	30.7793	31.005	30.3169	5	54.305	P04004	Vitronectin	188	VTN
24.7512	25.0546	25.2529	25.1761	NaN	NaN	4.7	53.688	P04066	Tissue alpha-L-fucosidase	190	FUCA1
25.9503	25.6094	25.8574	29.0504	26.5849	27.3106	23.4	38.714	P04083	Annexin A1	192	ANXA1
29.3701	30.2177	29.8735	29.7282	29.085	29.5787	1.6	515.6	P04114	Apolipoprotein B-100	193	APOB
25.88	25.7866	25.8032	26.3539	26.0498	26.5667	9.6	27.278	A2A2V1	Major prion protein (Fragment)	194	PRNP
24.2783	NaN	24.6681	24.8928	24.6709	24.7975	6.8	48.534	P04181	Ornithine aminotransferase, mitochondrial	196	OAT
31.6358	31.5449	31.4281	31.5727	31.4357	31.5994	26.3	16.927	E9PIM6	Thy-1 membrane glycoprotein (Fragment)	198	THY1
31.5058	29.0043	32.6601	31.5757	31.7074	30.3081	53.3	66.038	P04264	Keratin, type II cytoskeletal 1	199	KRT1
33.5786	33.64	33.7261	34.4512	34.0029	34.0119	70.1	36.053	P04406	Glyceraldehyde-3-phosphate dehydrogenase	201	GAPDH
27.3815	27.0959	27.0981	27.3218	27.4332	27.5596	15.9	51.657	P04424	Argininosuccinate lyase	202	ASL
25.2415	25.2262	25.4435	25.4804	25.3374	25.6797	7	42.021	P19961-2	Isoform 2 of Alpha-amylase 2B	203	AMY2B
29.6249	29.5249	29.5672	29.6358	29.3787	29.6012	52.7	22.782	P04792	Heat shock protein beta-1	204	HSPB1
28.5778	28.5817	28.4424	29.3876	28.6944	28.5469	28.7	40.45	P04899	Guanine nucleotide-binding protein G(i) subunit alpha-2	205	GNAI2
28.273	28.0616	27.7858	28.8787	28.5196	28.7584	16.2	109.55	P05023-3	Isoform 3 of Sodium/potassium-transporting ATPase subunit alpha-1	207	ATP1A1
26.4476	26.6815	26.6356	26.5078	26.6826	26.7239	16.3	34.893	P05026-2	Isoform 2 of Sodium/potassium-transporting ATPase subunit beta-1	208	ATP1B1
31.3138	31.128	30.4429	31.5324	31.2099	31.4927	31.5	87.057	P05106	Integrin beta-3	210	ITGB3
29.2335	28.8338	29.534	29.8817	30.0237	30.6054	40.5	45.059	P05121	Plasminogen activator inhibitor 1	211	SERPINE1
26.5016	26.7365	26.7976	26.488	NaN	25.2152	3.1	49.757	P05155-2	Isoform 2 of Plasma protease C1 inhibitor	212	SERPING1

26.2243	25.9453	26.2874	26.1319	25.7322	25.9061	18.3	28.865	H0YJS4	Eukaryotic translation initiation factor 2 subunit 1 (Fragment)	214	EIF2S1
29.1951	28.9289	28.2404	29.0157	29.1023	29.247	84.3	11.665	P05387	60S acidic ribosomal protein P2	215	RPLP2
28.8861	28.6521	28.7291	29.0673	29.1066	29.1593	43.2	34.273	P05388	60S acidic ribosomal protein P0	216	RPLP0
27.3953	27.0243	27.186	26.2932	27.053	26.7247	39.1	22.537	P05452	Tetranectin	218	CLEC3B
30.7328	30.6038	30.3213	30.8931	30.3735	30.7519	51.8	40.688	P05534	HLA class I histocompatibility antigen, A-24 alpha chain	220	HLA-A
29.5054	28.9879	28.808	NaN	28.4637	28.6511	3.8	57.07	P05546	Heparin cofactor 2	221	SERPIND1
32.6856	32.8785	32.7062	32.723	32.6715	32.9851	34	88.414	P05556	Integrin beta-1	222	ITGB1
25.2872	26.1708	25.2863	25.0567	25.115	25.1728	2.2	106.98	A0A087WY X9	Collagen alpha-2(V) chain	224	COL5A2
26.79	27.2779	27.5751	27.4378	27.7115	27.3371	25.1	40.786	P06132	Uroporphyrinogen decarboxylase	225	UROD
29.8812	29.8098	29.4131	29.4791	29.574	29.268	15.7	80.64	P06396-2	Isoform 2 of Gelsolin	226	GSN
27.2286	27.2592	27.4624	28.3264	27.6009	27.6775	18.1	56.559	P06576	ATP synthase subunit beta, mitochondrial	228	ATP5F1B
30.3326	29.9047	30.2014	30.6516	30.4269	30.0802	46.8	47.168	P06733	Alpha-enolase	231	ENO1
30.1669	30.038	30.0674	30.2283	30.3894	30.0558	24.4	93.133	P06737-2	Isoform 2 of Glycogen phosphorylase, liver form	232	PYGL
27.3008	27.0496	27.5803	27.3786	27.2721	27.8024	26.4	29.464	P06748-2	Isoform 2 of Nucleophosmin	234	NPM1
29.8315	29.8686	29.7679	30.2821	29.7061	30.0842	31.9	111.13	P06756-3	Isoform 3 of Integrin alpha-V	236	ITGAV
26.8311	26.8708	27.0132	27.2028	26.7877	26.7852	9.1	53.162	P06858	Lipoprotein lipase	237	LPL
30.8361	30.3884	30.5698	30.8486	30.7355	27.5646	46.8	13.92	Q16778	Histone H2B type 2-E	238	HIST2H2BE
25.6713	26.456	25.7664	26.3147	27.2117	27.8702	18	52.948	P07099	Epoxide hydrolase 1	240	EPHX1
31.5591	31.2771	31.699	31.5201	31.9574	31.6099	27.2	36.638	P07195	L-lactate dehydrogenase B chain	241	LDHB
26.6348	26.4014	26.5156	27.0283	26.7365	26.3468	17.8	21.938	A0A087WU Q6	Glutathione peroxidase	242	GPX1
29.58	28.8917	29.1734	29.0497	29.2371	29.3493	22.7	20.863	P07305	Histone H1.0	243	H1F0
26.0057	25.7757	25.9034	26.687	25.9728	26.338	11.6	35.785	H7C469	Uncharacterized protein (Fragment)	244	Uncharacterize d

29.0057	28.6207	28.7134	30.6738	29.3928	29.7463	49.9	38.604	P07355	Annexin A2	245	ANXA2
29.3143	30.0723	29.9786	30.8153	30.9369	30.6121	56.6	47.766	Q5JP53	Tubulin beta chain	246	TUBB
29.9191	29.575	29.8688	29.6288	29.2874	29.0608	63.6	15.054	P07737	Profilin-1	247	PFN1
27.4166	27.4224	27.351	27.2973	NaN	NaN	18.1	30.005	P07738	Bisphosphoglycerate mutase	248	BPGM
29.6224	29.7074	29.7494	29.6385	29.7969	29.8904	24.4	170.59	P07814	Bifunctional glutamate/proline--tRNA ligase	250	EPRS
30.8889	30.7276	30.8901	31.1396	30.6348	30.4688	40.4	84.659	P07900	Heat shock protein HSP 90-alpha	252	HSP90AA1
25.4487	25.5866	25.8598	25.799	25.8147	27.1343	24.7	25.256	G3V576	Heterogeneous nuclear ribonucleoproteins C1/C2	253	HNRNPC
31.6322	32.1004	31.4417	30.9386	30.836	31.6977	34.9	198.04	P07942	Laminin subunit beta-1	254	LAMB1
35.4396	36.4292	35.645	34.584	34.5541	34.8794	66.3	129.38	P07996	Thrombospondin-1	255	THBS1
30.2871	30.8434	30.1683	26.9036	26.7922	27.4826	21.3	129.15	A0A087WTA8	Collagen alpha-2(I) chain	256	COL1A2
27.0457	26.7466	27.1008	29.8077	27.97	28.7489	29.4	75.872	P08133	Annexin A6	257	ANXA6
31.266	31.4519	31.3877	31.8374	31.2446	31.5711	39.3	57.944	P08195-2	Isoform 2 of 4F2 cell-surface antigen heavy chain	259	SLC3A2
32.5482	32.7294	32.7171	32.5036	32.3867	32.2181	43.8	83.263	P08238	Heat shock protein HSP 90-beta	260	HSP90AB1
27.0076	27.8368	27.0465	26.6823	26.3239	26.2883	17.8	65.764	P08253-2	Isoform 2 of 72 kDa type IV collagenase	261	MMP2
29.3135	28.9543	28.6251	29.7382	28.5596	29.0204	3.1	88.036	A0A3B3IU79	Integrin alpha-IIb (Fragment)	264	ITGA2B
29.3581	29.4932	29.6684	29.2602	29.3901	29.0829	4	40.124	P08567	Pleckstrin	265	PLEK
27.9915	27.6919	27.8994	28.156	27.9204	27.7856	19	51.556	P08621	U1 small nuclear ribonucleoprotein 70 kDa	267	SNRNP70
29.3378	29.371	29.5591	28.9204	28.5183	28.8518	18	114.54	P08648	Integrin alpha-5	268	ITGA5
28.7302	28.7523	28.4294	29.0641	28.9237	28.8333	31.1	49.653	B0YJC4	Vimentin variant 3	269	VIM
29.1282	29.3918	29.2589	29.6898	29.6373	29.5282	11.8	27.823	C9JMH6	Alpha-2-antiplasmin (Fragment)	270	SERPINF2
28.1354	28.5652	28.1824	28.3315	28.6671	29.3972	23.7	15.55	P08708	40S ribosomal protein S17	271	RPS17
27.6305	26.7967	26.9564	29.4658	27.8433	28.1195	23.1	35.936	P08758	Annexin A5	272	ANXA5
31.0079	30.9238	31.0534	31.4923	31.0881	31.139	66.9	29.404	C9J9K3	40S ribosomal protein SA (Fragment)	273	RPSA

28.1683	28.1984	28.5804	28.2554	28.2002	27.4917	37.1	23.356	P09211	Glutathione S-transferase P	274	GSTP1
29.3088	29.1903	29.0443	28.913	29.0681	29.3659	57	14.716	P09382	Galectin-1	275	LGALS1
25.8939	25.8207	25.8467	26.81	NaN	NaN	13.9	25.789	P09417	Dihydropteridine reductase	276	QDPR
24.8753	24.9714	25.1811	25.0241	25.6364	25.5909	18.4	18.311	Q5T7C4	High mobility group protein B1	277	HMGB1
27.0359	26.5234	26.8797	26.0039	26.5842	26.0628	16.2	34.632	P09486	SPARC	279	SPARC
30.2664	29.5706	29.7719	29.6146	30.0943	30.1713	28.4	23.662	P09496-2	Isoform Non-brain of Clathrin light chain A	280	CLTA
26.5132	25.3352	24.9612	24.81	25.3008	25.7378	9	23.181	P09497-2	Isoform Non-brain of Clathrin light chain B	281	CLTB
29.1885	29.2359	29.0352	28.3317	27.9043	27.8984	11.2	123.97	P09619	Platelet-derived growth factor receptor beta	283	PDGFRB
25.5614	25.6088	25.3243	25.3214	25.7374	25.6696	15.2	25.687	F8VZ49	Heterogeneous nuclear ribonucleoprotein A1 (Fragment)	284	HNRNPA1
29.5721	29.3008	28.9945	29.5515	29.9829	30.1239	6.9	187.67	F5GXS0	Complement C4-B	286	C4B
30.5976	30.2023	30.2214	30.7107	30.9439	30.8897	31.2	13.509	Q71UI9	Histone H2A.V	287	H2AFV
31.1903	30.9825	31.1494	31.4174	31.1034	30.7291	29.8	70.051	P0DMV9	Heat shock 70 kDa protein 1B	288	HSPA1B
27.1052	27.0803	26.7674	27.4436	27.1815	27.0402	22.1	16.837	P0DP25	Calmodulin-3	289	CALM3
26.1727	26.5422	26.4515	25.932	25.6711	26.2452	17.4	35.015	P10646	Tissue factor pathway inhibitor	293	TFPI
26.6211	26.0746	26.468	26.9094	26.3174	26.051	20.9	31.462	P10768	S-formylglutathione hydrolase	294	ESD
25.9865	26.0835	26.0543	26.248	26.8713	26.666	15.2	61.054	P10809	60 kDa heat shock protein, mitochondrial	295	HSPD1
28.5499	29.0249	28.5828	29.3274	28.9316	28.3683	28.8	48.803	P10909-4	Isoform 4 of Clusterin	296	CLU
24.6862	23.779	23.9701	23.9782	23.976	NaN	8	72.332	P11021	Endoplasmic reticulum chaperone BiP	298	HSPA5
31.7499	32.0364	31.3141	30.9655	30.7437	31.6292	43.2	177.6	P11047	Laminin subunit gamma-1	299	LAMC1
32.7723	32.8192	32.8566	33.1012	32.7872	32.6205	48.6	70.897	P11142	Heat shock cognate 71 kDa protein	300	HSPA8
27.4556	27.3167	26.9929	27.8853	27.7881	27.4245	12.2	54.083	P11166	Solute carrier family 2, facilitated glucose transporter member 1	301	SLC2A1

27.63	27.3412	27.8255	27.7694	27.5845	27.3855	17	61.39	A0A2R8Y6D0	Protein 4.1	302	EPB41
27.6459	27.4958	27.4788	27.8139	28.2281	27.76	15.8	96.695	P11216	Glycogen phosphorylase, brain form	303	PYGB
25.934	25.6884	26.0774	NaN	26.0459	26.3165	15.5	23.408	P11234	Ras-related protein Ral-B	304	RALB
29.7644	29.2595	29.3955	29.8975	30.1755	29.9651	28.4	90.725	P11387	DNA topoisomerase 1	305	TOP1
30.1041	30.1303	30.4189	31.3702	30.9319	30.8181	48.5	59.256	P11413	Glucose-6-phosphate 1-dehydrogenase	307	G6PD
27.7233	27.4441	27.7473	27.6179	27.7688	27.7517	12.5	101.56	P11586	C-1-tetrahydrofolate synthase, cytoplasmic	308	MTHFD1
25.95	27.3079	27.2924	27.8664	27.272	27.186	4.1	274.37	P11717	Cation-independent mannose-6-phosphate receptor	309	IGF2R
28.7011	28.3806	28.5913	28.852	28.4852	28.2393	11.2	39.724	P11766	Alcohol dehydrogenase class-3	310	ADH5
25.5148	25.3444	25.2868	25.6125	25.4166	26.6178	7.5	58.535	A0A087WTT1	Polyadenylate-binding protein	311	PABPC1
34.3394	33.6535	33.9524	33.5382	34.0575	34.4588	35	108.34	A0A087X0S5	Collagen alpha-1(VI) chain	313	COL6A1
33.944	33.0327	33.4198	32.9249	33.5281	34.0868	30.8	108.58	P12110	Collagen alpha-2(VI) chain	314	COL6A2
36.0607	35.2014	35.6151	35.2222	35.7072	36.3549	46.1	343.67	P12111	Collagen alpha-3(VI) chain	315	COL6A3
32.1281	32.3946	32.3696	32.6107	32.2121	31.7885	6.9	251.7	P12259	Coagulation factor V	316	F5
26.161	26.0841	26.1888	25.7171	25.6518	25.6157	8.9	50.957	H0Y4R1	Inosine-5-monophosphate dehydrogenase 2 (Fragment)	317	IMPDH2
26.7787	26.5993	26.3058	26.6591	26.6979	26.555	8.8	94.825	H9KV75	Alpha-actinin-1	319	ACTN1
29.942	30.1036	29.9436	30.1848	30.571	30.5043	37.9	69.842	P12956	X-ray repair cross-complementing protein 6	320	XRCC6
30.1908	30.2375	30.1022	30.4415	30.8183	30.9498	30.3	82.704	P13010	X-ray repair cross-complementing protein 5	321	XRCC5
26.9882	26.9692	26.6285	27.5011	27.4055	27.9937	9.7	21.717	P13224	Platelet glycoprotein Ib beta chain	322	GP1BB
26.9464	26.4693	26.4755	26.1679	26.2652	NaN	17.6	49.973	P13489	Ribonuclease inhibitor	324	RNH1
26.8968	26.6683	26.7927	26.3512	26.6403	26.7125	11.3	81.082	P13497-5	Isoform BMP1-6 of Bone morphogenetic protein 1	325	BMP1
31.6769	33.1951	31.7912	31.6496	30.1332	31.0526	8.3	372.82	P13611	Versican core protein	326	VCAN
30.9266	31.1478	30.988	30.4402	30.3114	30.2506	32.4	114.9	P13612	Integrin alpha-4	327	ITGA4

31.8103	31.8701	32.007	32.1811	31.9087	31.8499	41.3	95.337	P13639	Elongation factor 2	328	EEF2
29.7685	26.8721	30.0304	29.3363	30.4945	28.5879	36.1	58.826	P13645	Keratin, type I cytoskeletal 10	329	KRT10
25.6936	25.5077	25.4208	26.4472	26.1711	26.3019	9.1	58.957	P13674-3	Isoform 3 of Prolyl 4-hydroxylase subunit alpha-1	332	P4HA1
27.4869	27.7961	27.8251	26.9118	26.9317	27.5522	16	27.145	P13726-2	Isoform 2 of Tissue factor	334	F3
24.6956	24.5495	24.6143	25.0283	NaN	25.7713	4.3	76.482	P13807-2	Isoform 2 of Glycogen [starch] synthase, muscle	335	GYS1
30.522	30.9371	30.6368	30.0161	30.2701	30.6461	29.6	11.985	E9PNW4	Uncharacterized protein	338	Uncharacterize d
26.4233	26.5783	26.8772	26.1719	25.4983	24.5792	5.5	36.573	P14550	Aldo-keto reductase family 1 member A1	339	AKR1A1
33.1078	33.0637	33.2345	33.6225	33.3851	33.1657	72.1	57.936	P14618	Pyruvate kinase PKM	340	PKM
26.8336	26.7491	27.0494	25.8542	26.1136	26.1371	9.5	92.468	P14625	Endoplasmin	341	HSP90B1
25.3894	25.3617	25.6258	NaN	25.2159	NaN	8.9	17.546	J3QLE5	Small nuclear ribonucleoprotein-associated protein N (Fragment)	342	SNRPN
28.9329	28.7341	28.8382	29.2255	29.0882	29.1998	43.1	45.771	P14868-2	Isoform 2 of Aspartate--tRNA ligase, cytoplasmic	344	DARS
28.8175	28.474	28.7172	29.3347	28.5631	28.2089	22.8	35.853	P15121	Aldose reductase	345	AKR1B1
25.3579	26.0492	26.3703	NaN	NaN	NaN	4.6	109.54	P15144	Aminopeptidase N	346	ANPEP
27.5781	27.2678	27.0154	27.2328	26.641	27.541	17	39.304	P15151-3	Isoform Gamma of Poliovirus receptor	347	PVR
26.0584	25.4252	25.7594	26.5018	26.6625	25.5997	6.3	52.832	H3BR35	Eukaryotic peptide chain release factor GTP-binding subunit ERF3A (Fragment)	350	GSPT1
26.0669	27.4188	26.9395	25.3495	25.8245	26.2419	7.2	39.313	P15529-15	Isoform K of Membrane cofactor protein	352	CD46
29.9063	29.6706	29.7398	30.9449	30.9311	30.9392	47.5	27.295	P15559-2	Isoform 2 of NAD(P)H dehydrogenase [quinone] 1	353	NQO1
30.5765	30.6059	30.6608	30.7334	31.4072	31.3114	43.3	31.324	P15880	40S ribosomal protein S2	354	RPS2
29.4597	29.2516	29.0145	30.1988	29.692	29.9193	30.1	22.683	H0YD13	CD44 antigen	356	CD44
28.2344	28.1477	28.3122	28.7757	28.766	28.4992	42.2	30.375	P16152	Carbonyl reductase [NADPH] 1	357	CBR1
27.0209	27.2664	27.4	NaN	NaN	27.5543	30.8	22.35	P16402	Histone H1.3	358	HIST1H1D

32.9809	32.8615	32.9765	33.0789	33.5856	33.9491	31.9	21.364	P16403	Histone H1.2	359	HIST1H1C
23.8502	23.8653	23.8536	24.665	24.0381	24.1113	15.1	18.214	H3BQV5	1-phosphatidylinositol 4,5-bisphosphate phosphodiesterase gamma-2 (Fragment)	360	PLCG2
25.4321	25.1626	24.9844	26.9458	25.4998	25.6221	6.4	102.83	E7ESP4	Integrin alpha-2	363	ITGA2
26.3355	26.6341	26.5452	26.6071	27.0305	26.6225	13.8	63.723	A0A3B3IRI2	CTP synthase	367	CTPS1
27.3499	28.3471	27.8926	27.4682	27.2016	27.5754	10.7	67.541	P17813-2	Isoform Short of Endoglin	368	ENG
27.6398	27.4704	27.6617	28.086	27.9514	28.0265	13.2	85.018	P17858	ATP-dependent 6-phosphofructokinase, liver type	370	PFKL
27.4257	28.0668	27.7974	28.1247	28.09	28.1772	33.8	47.352	R4GNH3	26S proteasome regulatory subunit 6A	371	PSMC3
30.7502	31.1696	31.0863	30.9124	30.9917	30.7682	54	60.343	P17987	T-complex protein 1 subunit alpha	372	TCP1
27.4525	27.4412	27.1144	27.3895	28.034	27.9539	41.8	6.4725	F8WBS5	60S ribosomal protein L35a	373	RPL35A
28.8222	28.6399	28.4325	29.4364	29.029	29.3181	28.2	88.053	P18084	Integrin beta-5	374	ITGB5
30.2307	29.7367	30.3362	29.7027	30.1676	29.8149	48.3	20.511	P18085	ADP-ribosylation factor 4	375	ARF4
29.7267	29.6129	29.3423	29.1736	29.7809	29.9866	40.7	29.225	P18124	60S ribosomal protein L7	376	RPL7
26.9642	26.7868	26.6146	27.1728	27.1436	27.1482	9.7	116.72	P18206-2	Isoform 1 of Vinculin	377	VCL
28.3881	28.3479	28.2088	28.641	28.8397	28.8033	40.8	19.586	A0A087WXM6	60S ribosomal protein L17 (Fragment)	379	RPL17
28.4506	28.5655	28.6082	28.9391	27.8212	27.3359	38.2	28.804	P18669	Phosphoglycerate mutase 1	380	PGAM1
26.0149	26.2259	26.6183	26.3317	26.6294	26.1739	11.9	33.824	P19623	Spermidine synthase	385	SRM
31.5646	31.8677	31.5307	31.2864	31.9117	32.1042	11.3	105.21	Q5T985	Inter-alpha-trypsin inhibitor heavy chain H2	386	ITIH2
27.2193	27.2425	26.684	26.0642	27.0909	27.4323	3.2	101.39	P19827	Inter-alpha-trypsin inhibitor heavy chain H1	387	ITIH1
28.6144	28.5561	29.1064	28.7588	28.6706	28.6303	28.8	23.548	P20340-2	Isoform 2 of Ras-related protein Rab-6A	388	RAB6A
26.6202	26.8882	26.4832	26.7388	NaN	27.3128	18.7	26.489	P20618	Proteasome subunit beta type-1	389	PSMB1
27.3476	27.4664	NaN	27.9151	27.5772	27.3215	24.9	30.993	P20645	Cation-dependent mannose-6-phosphate receptor	390	M6PR
31.9674	32.3192	31.2625	32.4222	32.3937	29.1747	3	163.86	P20742	Pregnancy zone protein	392	PZP

27.7783	28.8822	28.1302	27.1204	27.0151	27.1597	6	183.56	P20908	Collagen alpha-1(V) chain	394	COL5A1
32.5532	32.4873	32.3134	32.5317	32.4874	32.3758	42	278.22	Q60FE5	Filamin-A	397	FLNA
29.2264	29.2188	29.4124	29.4082	29.5937	29.3385	19.6	98.398	P21399	Cytoplasmic aconitate hydratase	398	ACO1
29.4454	29.467	29.363	29.9459	29.6968	30.2308	31.9	63.367	P21589	5-nucleotidase	399	NT5E
27.3319	25.0673	26.5935	NaN	NaN	24.6339	27.6	9.5122	P21741-2	Isoform 2 of Midkine	400	MDK
30.9873	31.4395	31.0247	27.9309	27.5282	27.4988	44	41.654	P21810	Biglycan	401	BGN
28.6973	28.9822	29.2263	28.9164	NaN	28.922	6.3	17.764	A6NNI4	Tetraspanin	402	CD9
25.6087	25.0161	25.6839	25.7896	25.3529	25.7234	3.6	107.77	P22102	Trifunctional purine biosynthetic protein adenosine-3	405	GART
26.1935	26.6228	26.5118	26.2329	26.2453	26.6939	15.6	37.111	D6RF62	Multifunctional protein ADE2	406	PAICS
30.2658	30.2704	30.3684	30.7215	30.2013	29.9662	32.1	113.8	P22314-2	Isoform 2 of Ubiquitin-like modifier-activating enzyme 1	407	UBA1
NaN	25.7242	25.5853	26.2852	26.1543	26.2676	25.7	17.298	P22392	Nucleoside diphosphate kinase B	409	NME2
NaN	25.076	25.1622	25.2562	25.3722	25.5339	13.4	29.836	A0A087WUI2	Heterogeneous nuclear ribonucleoproteins A2/B1	410	HNRNPA2B1
30.5596	31.0854	30.6516	30.3662	31.0919	30.7573	24.6	77.213	P23142	Fibulin-1	412	FBLN1
27.2427	27.152	27.2009	27.498	27.2118	27.502	9.2	119.09	P23229-4	Isoform Alpha-6X2A of Integrin alpha-6	414	ITGA6
25.9163	26.1485	26.09	26.261	25.8585	25.9847	11.9	76.149	P23246	Splicing factor, proline- and glutamine-rich	415	SFPQ
NaN	25.8725	25.5284	25.769	26.1069	26.3689	17.5	51.169	P23258	Tubulin gamma-1 chain	416	TUBG1
27.2978	27.2742	27.4704	27.5962	27.0102	26.8195	20.5	48.851	P23381-2	Isoform 2 of Tryptophan--tRNA ligase, cytoplasmic	418	WARS
30.3532	30.619	30.57	30.6068	30.7857	30.8085	70.4	26.688	P23396	40S ribosomal protein S3	419	RPS3
31.9832	31.9337	32.009	32.0781	31.9354	31.8211	44.2	47.716	P23526	Adenosylhomocysteinase	420	AHCY
29.92	29.8662	29.5976	29.3478	29.5639	29.5279	63.3	18.502	P23528	Cofilin-1	421	CFL1
28.5653	28.3788	28.5228	28.7205	28.2266	28.0641	14.3	90.069	P23921	Ribonucleoside-diphosphate reductase large subunit	422	RRM1
26.8297	27.1086	26.9534	26.657	26.2764	26.0871	20.4	24.763	P24534	Elongation factor 1-beta	423	EEF1B2
30.0495	29.9677	28.4231	24.5434	NaN	24.1112	19.6	230.86	P24821-4	Isoform 4 of Tenascin	425	TNC

28.8533	28.9181	28.3495	28.2468	29.4377	29.6661	53.8	14.515	P25398	40S ribosomal protein S12	428	RPS12
25.9922	26.2192	26.1864	26.2176	26.5259	26.2182	6.6	54.493	P25705-2	Isoform 2 of ATP synthase subunit alpha, mitochondrial	429	ATP5F1A
27.1321	27.4912	27.3157	27.6931	27.3974	28.2459	23.6	29.555	P25786	Proteasome subunit alpha type-1	430	PSMA1
26.5865	27.0683	26.6302	27.2827	26.7889	27.4431	21	27.647	P25788-2	Isoform 2 of Proteasome subunit alpha type-3	432	PSMA3
26.8797	27.2889	26.7976	27.1795	27.6009	27.7037	34.5	24.526	H0YMZ1	Proteasome subunit alpha type (Fragment)	433	PSMA4
32.1658	32.3763	32.4409	32.4303	32.2005	32.8	24.5	116.61	P26006	Integrin alpha-3	434	ITGA3
26.4803	25.9756	26.2491	26.7572	26.6905	26.2488	12.7	67.819	P26038	Moesin	436	MSN
29.0304	28.5328	28.2445	28.7145	28.7042	29.0611	26.5	24.261	P26373	60S ribosomal protein L13	437	RPL13
26.9164	27.0437	27.2033	26.7798	26.9762	27.0348	15.6	83.434	P26639	Threonine--tRNA ligase, cytoplasmic	438	TARS
28.1984	28.0782	28.2698	28.2248	28.2867	28.3865	14.6	140.47	P26640	Valine--tRNA ligase	439	VARS
30.7162	30.6573	30.8823	30.6708	30.5033	30.2019	33.2	50.118	P26641	Elongation factor 1-gamma	440	EEF1G
28.569	28.3182	28.2661	28.6186	28.2253	28.3244	29	27.764	P27348	14-3-3 protein theta	442	YWHAQ
28.6932	28.8526	28.635	28.4843	28.8326	28.7316	45	22.975	X1WI28	60S ribosomal protein L10 (Fragment)	444	RPL10
27.1052	26.8311	27.1037	27.4303	28.3283	27.9385	8	236.02	F8VPD4	CAD protein	446	CAD
27.8603	28.2688	27.8531	28.4846	28.0433	28.3867	42.3	26.411	P28066	Proteasome subunit alpha type-5	448	PSMA5
NaN	26.0646	25.2447	25.4019	25.5721	26.1099	18.6	28.48	P28074	Proteasome subunit beta type-5	450	PSMB5
27.4761	27.2545	27.5963	27.6239	27.3619	26.9755	23.1	41.389	P28482	Mitogen-activated protein kinase 1	451	MAPK1
32.3122	32.367	32.281	31.7296	32.1617	31.6893	50.9	138.35	P29144	Tripeptidyl-peptidase 2	452	TPP2
27.314	27.7537	27.5135	26.7169	26.5712	26.7033	9.2	108.27	P29317	Ephrin type-A receptor 2	453	EPHA2
26.5541	26.1727	25.6788	25.5617	25.3127	25.5304	8.8	105.56	B1AKC9	Ephrin type-B receptor 2	454	EPHB2
27.4013	27.3295	27.4681	28.7365	28.2383	28.036	23.6	67.877	P29401	Transketolase	455	TKT
28.1357	28.0999	27.8647	27.4901	27.1756	27.2132	45.2	18.157	E9PPR1	Elongation factor 1-delta (Fragment)	456	EEF1D
25.8249	25.655	25.9787	26.1308	25.3069	25.1271	12.9	28.151	A0A0C4DFS0	MAWD binding protein, isoform CRA_d	457	PBLD
27.1744	27.21	27.0072	27.0154	27.1404	26.8956	21	25.035	P30041	Peroxiredoxin-6	458	PRDX6

28.7554	28.2716	28.2228	28.2193	28.2012	28.3125	54.5	17.818	P30050	60S ribosomal protein L12	460	RPL12
27.9776	27.4678	27.5604	27.9226	27.7376	27.7079	23.3	65.308	P30153	Serine/threonine-protein phosphatase 2A 65 kDa regulatory subunit A alpha isoform	461	PPP2R1A
27.1082	26.7677	27.2884	27.69	27.2532	26.6764	18	48.14	P30419-2	Isoform Short of Glycylpeptide N-tetradecanoyltransferase 1	462	NMT1
28.2464	28.1897	27.8872	28.4233	27.3718	28.0441	27.9	40.455	P30685	HLA class I histocompatibility antigen, B-35 alpha chain	463	HLA-B
26.235	26.4608	26.3817	26.7119	26.269	26.7884	31.7	41.094	P30501	HLA class I histocompatibility antigen, Cw-2 alpha chain	464	HLA-C
26.9475	26.7808	26.7521	28.295	27.4615	27.2434	29.7	17.605	C9J0K6	Sorcin	467	SRI
26.7533	26.7498	26.9839	26.7487	26.8018	26.8135	8.1	67.638	P30825	High affinity cationic amino acid transporter 1	468	SLC7A1
29.0507	28.3343	28.6986	28.8618	28.3533	28.2256	8	51.026	P31146	Coronin-1A	469	CORO1A
26.8209	26.5044	26.8035	26.9075	27.4775	27.0378	20.4	32.964	P31153-2	Isoform 2 of S-adenosylmethionine synthase isoform type-2	471	MAT2A
25.0094	25.182	25.0606	26.1534	25.141	25.2433	3.9	37.044	P31689-2	Isoform 2 of DnaJ homolog subfamily A member 1	473	DNAJA1
30.0696	29.864	30.1809	30.121	30.2171	30.3532	43.2	64.615	P31939	Bifunctional purine biosynthesis protein PURH	474	ATIC
26.8655	26.4184	26.2824	26.6299	26.8881	26.9874	17.5	47.087	E9PCY7	Heterogeneous nuclear ribonucleoprotein H	475	HNRNPH1
25.7745	25.7565	25.5135	25.8806	25.8251	25.5915	22.5	27.85	P31946-2	Isoform Short of 14-3-3 protein beta/alpha	476	YWHAB
26.4017	26.6857	26.3333	25.5669	26.6938	26.1484	12.7	62.639	P31948	Stress-induced-phosphoprotein 1	477	STIP1
NaN	NaN	25.0497	25.5138	25.3837	25.3429	19	11.74	P31949	Protein S100-A11	478	S100A11
28.2594	27.8525	27.4652	27.9035	28.3312	28.2727	39.6	21.863	P32969	60S ribosomal protein L9	480	RPL9
26.6451	26.5334	26.5333	26.4213	26.5505	26.5173	9.4	109.68	P33176	Kinesin-1 heavy chain	481	KIF5B
26.7103	26.9935	26.7599	27.3529	26.478	26.045	13.3	72.968	P33908	Mannosyl-oligosaccharide 1,2-alpha-mannosidase IA	482	MAN1A1

27.3053	26.8856	27.3266	27.3998	27.4047	27.348	22.7	49.027	P34896-2	Isoform 2 of Serine hydroxymethyltransferase, cytosolic	485	SHMT1
29.2695	29.2095	29.4057	29.6387	29.5589	29.4379	46.4	53.454	P34897-3	Isoform 3 of Serine hydroxymethyltransferase, mitochondrial	486	SHMT2
26.7344	26.4852	26.7971	26.9249	26.7394	26.5506	18.7	52.599	A0A087WTS8	Heat shock 70 kDa protein 4	487	HSPA4
26.8184	27.1537	27.0948	27.9867	27.6325	27.6591	19.7	61.68	P35052	Glypican-1	488	GPC1
26.0404	27.0077	26.2308	25.7774	26.4913	27.054	4.3	96.005	E7ES19	Thrombospondin-4	492	THBS4
30.2914	27.8611	31.4445	30.0666	29.574	28.3513	46.9	62.064	P35527	Keratin, type I cytoskeletal 9	493	KRT9
25.1922	25.3209	24.8363	24.219	NaN	24.8082	1.8	312.24	P35555	Fibrillin-1	494	FBN1
33.0499	32.8336	32.9475	32.9381	32.9189	32.646	41.1	226.53	P35579	Myosin-9	496	MYH9
26.9472	NaN	26.7739	26.9833	26.9295	27.4663	8.1	229	P35580	Myosin-10	497	MYH10
28.1576	28.0293	28.1375	28.396	27.9933	28.0086	18.2	99.045	P35606-2	Isoform 2 of Coatomer subunit beta	498	COPB2
29.5807	29.9344	29.6358	29.7343	29.2369	29.6004	44.9	19.438	P35613-3	Isoform 3 of Basigin	499	BSG
29.3539	29.0847	29.3697	28.439	28.8494	29.1282	37.4	24.145	P35625	Metalloproteinase inhibitor 3	500	TIMP3
27.0972	27.3383	27.3506	28.1984	27.1063	26.937	3.6	66.034	P35858	Insulin-like growth factor-binding protein complex acid labile subunit	501	IGFALS
29.2392	24.6041	30.1505	28.5623	30.121	26.8646	50.4	65.432	P35908	Keratin, type II cytoskeletal 2 epidermal	502	KRT2
28.0816	28.0598	27.9185	28.286	28.388	28.5273	34.6	48.633	P35998	26S proteasome regulatory subunit 7	503	PSMC2
30.1858	30.2898	29.8609	30.5445	30.9297	30.7761	39.6	47.697	P36578	60S ribosomal protein L4	505	RPL4
29.8767	30.0447	30.0563	29.8902	30.0099	29.4599	39.9	61.448	P36871	Phosphoglucomutase-1	506	PGM1
28.8479	29.13	29.2707	28.1536	29.0698	28.729	9.3	46.312	P36955	Pigment epithelium-derived factor	507	SERPINF1
29.2044	29.163	29.1061	29.3255	28.7297	28.5321	54.3	22.391	P37802	Transgelin-2	508	TAGLN2
25.9352	26.3175	26.3482	26.5729	26.7005	26.3876	17.5	37.54	P37837	Transaldolase	509	TALDO1
28.0562	27.7107	27.6943	28.0541	27.6917	27.9206	17	64.735	P38606-2	Isoform 2 of V-type proton ATPase catalytic subunit A	510	ATP6V1A
27.7414	27.881	27.7155	28.0383	28.4178	28.4525	23	73.68	P38646	Stress-70 protein, mitochondrial	511	HSPA9

26.5053	26.3312	25.88	NaN	26.7992	27.1823	29	16.06	P39019	40S ribosomal protein S19	512	RPS19
29.3057	29.4232	29.1612	29.5594	29.6721	29.8178	40.4	46.108	P39023	60S ribosomal protein L3	513	RPL3
28.2874	27.9535	27.5222	27.6193	28.1742	28.5378	8.4	140.06	A0A087X0K0	Collagen alpha-1(XV) chain	514	COL15A1
26.1523	26.6157	26.2297	26.2372	25.4629	26.7971	4.2	135.76	P39060-2	Isoform 3 of Collagen alpha-1(XVIII) chain	515	COL18A1
29.9578	30.1408	30.0682	29.8614	30.3257	30.2109	29.4	58.024	P40227	T-complex protein 1 subunit zeta	516	CCT6A
28.3116	27.8995	27.738	28.1025	28.3744	28.563	20.7	23.577	P40429	60S ribosomal protein L13a	518	RPL13A
26.8628	26.3485	26.4224	26.4151	26.5287	26.0537	10	83.125	P40763-3	Isoform 3 of Signal transducer and activator of transcription 3	519	STAT3
28.0708	27.6161	27.8399	28.3247	27.826	27.4268	29.9	51.109	P41091	Eukaryotic translation initiation factor 2 subunit 3	521	EIF2S3
30.1993	29.9224	30.428	29.309	29.165	29.0047	50.4	40.886	P41221-2	Isoform 2 of Protein Wnt-5a	522	WNT5A
28.7188	28.8935	28.9164	28.9664	29.1803	29.6085	16.9	83.165	P41250	Glycine--tRNA ligase	523	GARS
29.5327	29.6108	29.6344	29.7532	29.6622	29.6781	24.3	144.5	P41252	Isoleucine--tRNA ligase, cytoplasmic	524	IARS
25.4744	25.8304	NaN	25.9783	25.7544	25.503	6.7	87.334	P42224	Signal transducer and activator of transcription 1-alpha/beta	526	STAT1
25.0719	25.2808	25.087	28.7506	28.0666	27.2919	38.7	36.867	A0A0A0MSS8	Aldo-keto reductase family 1 member C3	528	AKR1C3
26.7371	26.7764	26.8815	26.8321	27.3895	27.9118	25	9.4771	Q71UM5	40S ribosomal protein S27-like	529	RPS27L
NaN	27.2029	27.1999	27.6121	27.2149	27.4327	15.4	14.551	P42766	60S ribosomal protein L35	530	RPL35
27.955	27.8756	27.9795	28.3539	27.4876	27.097	29.5	46.637	P43034	Platelet-activating factor acetylhydrolase IB subunit alpha	532	PAFAH1B1
27.7762	27.9104	27.4207	26.847	26.9908	27.2515	12.5	71.607	P43121	Cell surface glycoprotein MUC18	533	MCAM
25.6067	25.5253	NaN	26.6046	26.4728	26.5497	16.3	41.864	A0A0C4DFS8	Nicotinamide phosphoribosyltransferase	535	NAMPT
27.745	27.9465	27.8979	28.4426	28.2857	28.4025	23.2	47.366	P43686	26S proteasome regulatory subunit 6B	536	PSMC4

25.5561	25.7021	25.7903	25.6058	NaN	25.8956	5.2	73.457	P46063	ATP-dependent DNA helicase Q1	538	RECQL
25.4292	25.3657	25.3921	26.4696	26.1173	25.6153	5.7	69.614	P46087-3	Isoform 3 of Probable 28S rRNA (cytosine(4447)-C(5))-methyltransferase	539	NOP2
25.5714	25.1485	25.5529	25.5628	25.7432	26.1182	6.7	71.583	P46459-2	Isoform 2 of Vesicle-fusing ATPase	541	NSF
28.575	28.319	28.0834	28.5079	28.8403	28.9234	34.5	16.561	P46776	60S ribosomal protein L27a	543	RPL27A
30.5055	30.7022	30.7769	30.6802	30.8571	30.497	48.3	27.045	A0A2R8Y6J3	60S ribosomal protein L5 (Fragment)	544	RPL5
28.6797	27.6328	27.3331	28.9935	28.7598	28.8738	29.9	9.8864	G3V1B3	60S ribosomal protein L21	545	RPL21
26.8918	26.8309	26.616	26.4841	27.0083	26.7532	33.6	15.747	P46779	60S ribosomal protein L28	546	RPL28
30.4592	30.3503	30.513	30.3962	30.8051	31.1382	40.7	22.591	A0A024R4M0	40S ribosomal protein S9	547	RPS9
NaN	27.6856	27.5824	27.2386	28.1378	28.2925	31	22.391	M0R0F0	40S ribosomal protein S5 (Fragment)	548	RPS5
25.8972	26.4797	26.6158	26.4336	26.6409	26.7797	32.1	18.898	P46783	40S ribosomal protein S10	549	RPS10
25.9271	25.8439	26.0112	26.6158	25.9009	NaN	4.8	189.25	P46940	Ras GTPase-activating-like protein IQGAP1	550	IQGAP1
26.1806	26.1018	26.2914	26.8439	25.9654	25.7469	21.7	32.949	P47755	F-actin-capping protein subunit alpha-2	552	CAPZA2
27.1707	27.1686	27.4627	27.9776	27.2396	26.8646	28.5	29.295	B1AK87	F-actin-capping protein subunit beta	553	CAPZB
28.2133	28.4591	28.4301	28.7205	28.5888	28.8604	25.1	86.578	P47897-2	Isoform 2 of Glutamine--tRNA ligase	554	QARS
30.5762	30.2426	30.5819	30.6705	31.2642	30.8134	43.1	37.251	P48059	LIM and senescent cell antigen-like-containing domain protein 1	556	LIMS1
25.7237	26.0661	26.0529	26.8708	25.8555	25.5917	10.1	80.699	P48147	Prolyl endopeptidase	558	PREP
31.778	30.9701	31.1234	31.2185	30.4328	30.9177	11.7	25.588	K4DIA7	Tetraspanin (Fragment)	561	CD151
26.443	26.2371	26.4209	25.9926	26.6814	26.7279	17.4	32.551	R4GMR5	26S proteasome non-ATPase regulatory subunit 8	562	PSMD8
30.5279	30.7522	30.7355	30.5049	30.7626	30.6886	47.7	59.67	P48643	T-complex protein 1 subunit epsilon	563	CCT5
26.6804	26.3517	26.3293	27.2323	26.439	26.6126	7.3	81.742	P48960-2	Isoform 2 of CD97 antigen	567	CD97

28.4285	28.67	28.8739	29.133	28.5867	28.4511	18	53.801	P49189	4-trimethylaminobutyraldehyde dehydrogenase	568	ALDH9A1
NaN	24.2008	24.0228	24.2272	25.0682	25.2568	15.4	13.293	P49207	60S ribosomal protein L34	569	RPL34
32.3835	32.4401	32.5552	32.7075	32.2289	32.0704	45.2	273.42	P49327	Fatty acid synthase	570	FASN
31.0162	31.2952	31.1099	30.8454	31.0139	31.0106	49.7	60.533	P49368	T-complex protein 1 subunit gamma	571	CCT3
24.9157	25.2039	25.1225	25.1614	25.2545	25.8806	16.8	49.541	P49411	Elongation factor Tu, mitochondrial	572	TUFM
27.6205	27.2069	27.3016	27.7348	27.3371	27.189	12.1	106.81	P49588	Alanine--tRNA ligase, cytoplasmic	574	AARS
28.1522	27.9701	28.3743	28.3385	27.9947	27.8204	21.6	58.777	P49591	Serine--tRNA ligase, cytoplasmic	575	SARS
25.5933	26.7768	25.7199	24.6857	NaN	24.9031	8.4	103.25	F5H4Z8	Thrombospondin-3	578	THBS3
26.9957	26.8541	26.974	27.1394	26.6328	27.2807	14.2	68.058	P49748-2	Isoform 2 of Very long-chain specific acyl-CoA dehydrogenase, mitochondrial	579	ACADVL
27.8338	27.6465	27.7106	27.8763	27.5841	27.2894	9.2	35.126	P49888	Estrogen sulfotransferase	581	SULT1E1
26.4877	26.6717	26.7218	27.0298	26.8719	27.0382	13.6	65.928	P49915-2	Isoform 2 of GMP synthase [glutamine-hydrolyzing]	582	GMPS
25.653	26.1759	26.0056	26.9977	26.3547	27.3636	10.3	65.893	P50281	Matrix metalloproteinase-14	583	MMP14
NaN	24.5794	24.6975	25.2316	24.5082	24.4845	6.7	50.663	P50395	Rab GDP dissociation inhibitor beta	584	GDI2
25.5206	25.2157	25.4067	25.1118	25.3938	24.4908	12.9	23.092	E9PKH2	Serpin H1	585	SERPINH1
29.0632	28.995	29.0064	29.0687	29.0034	28.9614	16.8	39.829	P50552	Vasodilator-stimulated phosphoprotein	587	VASP
28.7154	28.2545	28.7837	28.8361	28.6956	29.0746	21.1	97.553	P50570-3	Isoform 3 of Dynamin-2	588	DNM2
28.5289	28.3728	28.4415	28.8729	29.0438	29.0291	36.3	14.558	E7EPB3	60S ribosomal protein L14	589	RPL14
30.9786	31.2957	31.1285	30.8527	31.0741	30.948	58.2	59.62	P50990	T-complex protein 1 subunit theta	590	CCT8
30.4681	30.9007	30.7375	30.4241	30.8148	30.7078	41	57.924	P50991	T-complex protein 1 subunit delta	591	CCT4
28.3152	27.7453	28.1533	28.579	28.3283	28.1566	33.3	23.482	P51148	Ras-related protein Rab-5C	592	RAB5C
28.3263	27.9523	28.2072	28.7928	28.536	28.5713	55.6	23.489	P51149	Ras-related protein Rab-7a	593	RAB7A
29.2229	28.741	29.4127	28.3023	29.5748	29.4953	11.5	42.272	P51570	Galactokinase	594	GALK1

28.109	28.0802	28.0858	28.3583	28.6395	28.9477	24.4	37.025	P51665	26S proteasome non-ATPase regulatory subunit 7	595	PSMD7
24.8612	25.0865	24.9858	25.7971	25.2922	25.7627	17.4	37.029	P51991-2	Isoform 2 of Heterogeneous nuclear ribonucleoprotein A3	599	HNRNPA3
28.651	28.4904	28.6442	28.6527	28.5874	28.2004	21.9	51.872	P52209-2	Isoform 2 of 6-phosphogluconate dehydrogenase, decarboxylating	600	PGD
NaN	NaN	NaN	26.3475	26.3498	26.7783	45.5	33.834	B4DK69	Aldo-keto reductase family 1 member C2	603	AKR1C2
28.6992	28.5779	28.6467	29.2373	28.5187	27.6172	29.4	32.922	P52907	F-actin-capping protein subunit alpha-1	604	CAPZA1
29.7791	29.742	29.829	29.9901	29.9495	29.717	37.8	120.84	P53396	ATP-citrate synthase	606	ACLY
27.7895	28.0447	28.1204	28.3205	28.0897	28.0536	21.6	107.14	P53618	Coatomer subunit beta	607	COPB1
29.2717	29.0581	29.1479	29.757	29.2637	29.1795	24.3	138.34	P53621	Coatomer subunit alpha	608	COPA
27.5504	27.9214	28.1485	27.8653	27.5117	26.7155	10.6	51.853	P53634	Dipeptidyl peptidase 1	609	CTSC
27.7529	27.8177	28.0862	28.277	27.9262	27.897	19.5	75.378	P54136	Arginine--tRNA ligase, cytoplasmic	612	RARS
25.6165	25.3906	25.6101	26.359	25.9038	25.511	9.8	51.086	A6NJA2	Ubiquitin carboxyl-terminal hydrolase 14	615	USP14
26.7416	26.8937	26.7852	28.0552	27.6873	27.8942	29.7	31.512	P54709	Sodium/potassium-transporting ATPase subunit beta-3	616	ATP1B3
28.0211	27.8094	28.0121	27.9993	27.6116	27.7251	11.6	107.78	P55060-3	Isoform 3 of Exportin-2	618	CSE1L
29.5972	29.6643	29.2225	30.1284	30.0781	30.0645	34	89.321	P55072	Transitional endoplasmic reticulum ATPase	619	VCP
25.9902	25.5092	26.7826	28.1634	27.1092	26.6352	7.1	28.648	P55083	Microfibril-associated glycoprotein 4	620	MFAP4
28.1363	28.0138	28.0351	28.6024	28.2712	27.9094	28.8	23.417	H0YHC3	Nucleosome assembly protein 1-like 1 (Fragment)	621	NAP1L1
29.1431	29.1099	29.541	29.1034	29.2873	29.3143	25.2	34.084	P55263-3	Isoform 3 of Adenosine kinase	623	ADK
26.7728	26.6518	26.8258	27.0664	26.831	26.9847	28	35.54	P55735	Protein SEC13 homolog	625	SEC13
27.62	27.6005	27.8475	27.9566	28.0444	27.9525	18.1	92.48	P55884	Eukaryotic translation initiation factor 3 subunit B	627	EIF3B

26.1084	26.1141	26.4348	26.4792	27.05	27.1753	6.9	101.11	P56192	Methionine--tRNA ligase, cytoplasmic	628	MARS
26.7704	26.7489	26.9761	27.0275	26.7365	26.1729	22.9	26.599	P56537	Eukaryotic translation initiation factor 6	629	EIF6
31.7934	30.6757	31.3596	30.5089	30.2772	30.3187	44.6	38.561	P58166	Inhibin beta E chain	630	INHBE
26.3892	26.6514	27.0154	26.8375	27.3277	26.9853	32.1	19.667	P59998	Actin-related protein 2/3 complex subunit 4	632	ARPC4
33.2623	33.1905	32.8997	32.7412	32.5166	32.5309	35.8	17.963	E9PJK1	Tetraspanin	633	CD81
26.6125	26.2807	26.286	26.5474	27.7098	26.7233	25.3	26.669	P60174-1	Isoform 2 of Triosephosphate isomerase	634	TPI1
26.6716	26.6302	26.7985	27.4062	26.9747	27.4174	23.3	41.357	E5RGA2	Eukaryotic translation initiation factor 3 subunit E	636	EIF3E
29.86	29.6949	29.4527	29.913	29.735	29.8788	38.6	16.29	F8W1R7	Myosin light polypeptide 6	637	MYL6
34.9022	35.058	35.0182	34.6799	35.0363	34.9016	69.1	41.736	P60709	Actin, cytoplasmic 1	638	ACTB
30.6912	30.5132	30.849	30.7534	30.6243	30.7481	52.5	46.153	P60842	Eukaryotic initiation factor 4A-I	639	EIF4A1
28.538	28.7446	28.5946	28.4278	28.4855	28.7284	25.2	13.373	P60866	40S ribosomal protein S20	640	RPS20
27.0034	26.2928	26.8046	26.8775	26.7851	27.0891	23.5	31.392	B1ALA9	Ribose-phosphate pyrophosphokinase 1	641	PRPS1
27.0746	27.7058	27.2778	27.8257	27.3605	28.3377	31.7	27.399	P60900	Proteasome subunit alpha type-6	642	PSMA6
24.2474	24.5012	24.5178	26.2196	NaN	24.5703	35.1	11.203	P60903	Protein S100-A10	643	S100A10
28.9756	28.4981	29.0571	29.3766	29.391	28.588	29.3	21.258	P60953	Cell division control protein 42 homolog	644	CDC42
26.0082	25.8473	26.0815	26.2248	27.0086	26.1525	26.6	23.668	P61006	Ras-related protein Rab-8A	646	RAB8A
28.1234	28.0196	28.3102	28.4509	28.037	27.875	48.1	23.545	P61019	Ras-related protein Rab-2A	647	RAB2A
26.5113	26.7641	26.8597	27.0149	26.5818	26.0029	30.1	20.9	P61081	NEDD8-conjugating enzyme Ubc12	649	UBE2M
27.5281	27.3945	27.576	28.0863	27.5713	27.3728	43.6	20.409	X6RFL8	Ras-related protein Rab-14 (Fragment)	650	RAB14
28.6305	28.6912	28.6572	29.2039	29.1932	28.7326	42.3	47.371	P61158	Actin-related protein 3	651	ACTR3
28.8922	28.6201	28.8971	29.4369	29.2439	28.7994	27.9	44.76	P61160	Actin-related protein 2	652	ACTR2
27.7925	27.3863	27.854	27.9445	27.3395	27.5586	22.9	37.433	R4GMT0	Alpha-centractin	653	ACTR1A
29.7271	30.0145	30.1556	29.8679	30.0693	29.8689	38.7	20.697	P84077	ADP-ribosylation factor 1	654	ARF1

NaN	24.9344	25.0632	24.9852	25.0151	25.0762	3.7	67.314	P61221	ATP-binding cassette sub-family E member 1	655	ABCE1
31.7058	31.6736	31.9024	31.8446	31.9526	31.5922	46.7	18.778	P61224-3	Isoform 3 of Ras-related protein Rap-1b	656	RAP1B
29.9534	30.0738	30.2185	30.4044	30.3302	30.3834	47.5	25.608	D6RG13	40S ribosomal protein S3a (Fragment)	657	RPS3A
26.9719	27.6497	27.6138	28.1293	28.1958	28.1686	36.4	12.63	J3QRI7	60S ribosomal protein L26 (Fragment)	658	RPL26
29.6081	29.4672	29.3704	29.9591	30.0766	30.2666	41.2	24.146	P61313	60S ribosomal protein L15	660	RPL15
29.0607	28.9253	29.1614	29.211	29.2462	29.1896	36	15.798	P61353	60S ribosomal protein L27	661	RPL27
25.4113	25.9322	25.618	26.0452	26.2503	26.9101	35	6.793	M0R0A1	60S ribosomal protein L37a	662	RPL37A
28.5549	27.6099	28.422	28.5289	27.8483	27.8373	36.8	21.768	P61586	Transforming protein RhoA	663	RHOA
28.2447	28.2364	27.4403	28.0474	28.1852	28.3559	35.3	13.714	P61769	Beta-2-microglobulin	664	B2M
28.456	29.1832	29.2078	29.4818	28.8061	28.5458	56.7	14.478	P61970	Nuclear transport factor 2	666	NUTF2
28.158	28.1644	28.2008	27.9814	28.1096	28.2427	17.4	41.807	Q5T6W2	Heterogeneous nuclear ribonucleoprotein K (Fragment)	667	HNRNPK
NaN	25.6609	25.3959	25.7052	25.3092	25.0021	19.8	28.302	P61981	14-3-3 protein gamma	668	YWHAG
26.0291	25.9937	25.9115	25.7227	25.5034	25.44	13.2	22.462	B7Z317	Tetraspanin	669	TSPAN5
NaN	26.795	26.689	26.5146	26.6488	27.5668	14.8	19.405	A0A2R8Y62 3	40S ribosomal protein S7 (Fragment)	670	RPS7
28.8101	28.8536	29.0445	29.08	28.8765	28.5915	29.7	37.512	P62136	Serine/threonine-protein phosphatase PP1-alpha catalytic subunit	671	PPP1CA
27.5894	27.6763	27.3602	28.1686	28.0755	28.1533	23.4	49.184	P62191	26S proteasome regulatory subunit 4	673	PSMC1
27.5237	27.4894	27.1114	27.6144	27.8466	27.9358	34.9	44.784	P62195-2	Isoform 2 of 26S proteasome regulatory subunit 8	674	PSMC5
31.0738	31.002	31.084	31.4098	31.7101	32.0248	55.3	24.205	P62241	40S ribosomal protein S8	675	RPS8
30.1263	30.1001	30.2126	30.5328	30.5267	30.5415	50	16.445	P62249	40S ribosomal protein S16	676	RPS16
27.4325	27.6788	27.5574	27.6393	27.6613	27.235	30.2	29.174	P62258	14-3-3 protein epsilon	677	YWHAE
27.9236	27.619	27.7324	27.4741	27.8016	28.1082	36.7	16.159	A0A2R8Y81 1	40S ribosomal protein S14 (Fragment)	678	RPS14
29.1437	28.8206	28.7278	28.5806	28.9281	29.1964	30.1	15.807	P62266	40S ribosomal protein S23	679	RPS23

28.63	28.5238	28.3029	28.8307	28.6291	29.561	40.8	17.718	P62269	40S ribosomal protein S18	680	RPS18
28.8333	29.0465	28.6502	29.6212	29.6301	29.2611	35.8	17.222	P62277	40S ribosomal protein S13	682	RPS13
30.0969	30.0957	30.0057	30.0558	30.774	30.8657	56.3	18.431	P62280	40S ribosomal protein S11	683	RPS11
26.8843	26.6591	27.1017	26.7959	27.3607	27.0636	32.6	20.082	P62330	ADP-ribosylation factor 6	688	ARF6
27.6903	28.3006	28.2263	28.7144	28.6772	28.7294	44	44.172	P62333	26S proteasome regulatory subunit 10B	689	PSMC6
30.063	29.5709	29.4	29.9956	29.9562	30.2383	40.2	29.995	P62424	60S ribosomal protein L7a	690	RPL7A
30.1904	30.4836	30.5208	30.5949	30.8263	30.8327	53.6	29.597	P62701	40S ribosomal protein S4, X isoform	691	RPS4X
32.485	32.4841	32.5027	32.4596	32.014	31.6828	54.1	42.009	P62736	Actin, aortic smooth muscle	692	ACTA2
26.4301	26.1236	25.8986	26.2689	26.6726	26.7929	47.1	7.9232	K7EMA7	60S ribosomal protein L23a	693	RPL23A
27.9793	28.5587	28.1283	28.3462	28.366	28.4419	17.4	24.968	A2A3R5	40S ribosomal protein S6	694	RPS6
34.7134	34.5192	34.7173	34.8097	35.3077	34.9857	54.4	11.367	P62805	Histone H4	695	HIST1H4A
29.3751	29.0136	29.376	29.2926	29.2356	29.2418	49.7	19.018	E7END7	Ras-related protein Rab-1A	696	RAB1A
31.451	30.9265	31.3813	31.4937	31.515	31.3257	43.5	24.423	P62826	GTP-binding nuclear protein Ran	697	RAN
27.2266	26.933	26.9204	27.1835	27.2343	27.3526	48.4	9.6694	C9JD32	60S ribosomal protein L23 (Fragment)	698	RPL23
25.6933	26.3823	26.257	26.3478	26.8073	27.3595	46.4	12.99	K7EM56	40S ribosomal protein S15	700	RPS15
27.9179	27.6995	28.0215	27.9495	28.7881	28.6973	20.8	15.069	P62847-2	Isoform 2 of 40S ribosomal protein S24	701	RPS24
27.8674	27.7214	27.5389	27.5582	26.0128	27.7917	28	13.742	P62851	40S ribosomal protein S25	702	RPS25
29.2329	29.0324	29.1317	28.8748	29.554	29.6365	37.4	13.015	P62854	40S ribosomal protein S26	703	RPS26
28.9548	28.8298	28.9818	29.602	29.2065	28.9532	33.1	36.297	P62873-2	Isoform 2 of Guanine nucleotide-binding protein G(I)/G(S)/G(T) subunit beta-1	705	GNB1
27.6501	26.8986	27.1007	28.0043	27.6975	27.1226	25.3	37.331	P62879	Guanine nucleotide-binding protein G(I)/G(S)/G(T) subunit beta-2	706	GNB2
28.7343	27.9276	28.0826	28.3037	28.5563	28.5891	58.8	12.656	E5RI99	60S ribosomal protein L30 (Fragment)	707	RPL30
27.5182	27.4804	27.4293	27.3546	27.7085	26.9897	29.5	24.831	P62906	60S ribosomal protein L10a	709	RPL10A
28.9634	28.5853	28.5916	28.8169	28.9812	29.0417	41.4	15.616	D3YTB1	60S ribosomal protein L32 (Fragment)	710	RPL32

NaN	27.9745	27.6453	27.6813	28.114	27.8292	23.4	19.024	Q5VVC8	60S ribosomal protein L11	711	RPL11
28.7415	28.8264	28.308	28.8333	29.2463	28.9346	35.1	22.389	E9PKZ0	60S ribosomal protein L8 (Fragment)	712	RPL8
29.6335	29.4873	29.5469	30.2361	29.8847	29.5899	53.3	18.012	P62937	Peptidyl-prolyl cis-trans isomerase A	713	PPIA
29.6413	29.2539	29.6675	29.7296	29.8531	29.4595	42.3	17.965	P62979	Ubiquitin-40S ribosomal protein S27a	715	RPS27A
25.5164	25.4286	25.4838	26.2647	26.0267	26.5381	22.9	21.935	P62995-3	Isoform 3 of Transformer-2 protein homolog beta	716	TRA2B
29.7228	29.1137	29.5801	29.835	29.7825	29.7586	49.5	21.45	P63000	Ras-related C3 botulinum toxin substrate 1	717	RAC1
26.2878	25.7414	26.0462	26.0813	26.7598	27.0549	8	70.916	A0A087WYD1	AP-2 complex subunit beta (Fragment)	718	AP2B1
28.0105	28.003	27.7258	28.6155	28.2674	28.1127	29	27.745	P63104	14-3-3 protein zeta/delta	719	YWHAZ
26.9728	26.5446	27.0509	26.424	26.6926	26.5701	24.7	10.35	Q96FJ2	Dynein light chain 2, cytoplasmic	721	DYNLL2
25.8965	25.8051	25.8561	26.1807	26.5558	26.44	35.9	7.5649	J3KT73	60S ribosomal protein L38	722	RPL38
25.952	NaN	25.375	26.0274	26.3945	26.4475	29.6	8.85	Q8WVC2	40S ribosomal protein S21	723	RPS21
27.9154	28.4399	28.3419	28.2051	28.2481	28.3581	34.2	16.019	I3L397	Eukaryotic translation initiation factor 5A (Fragment)	724	EIF5A
31.1408	31.1515	31.3602	31.5928	31.5228	31.6949	75.4	35.076	P63244	Receptor of activated protein C kinase 1	725	RACK1
29.0071	28.7868	29.2819	29.4374	29.1057	28.7419	55.7	35.594	P67775	Serine/threonine-protein phosphatase 2A catalytic subunit alpha isoform	727	PPP2CA
27.7907	28.0224	26.6443	26.6915	26.3047	25.9892	21	35.924	P67809	Nuclease-sensitive element-binding protein 1	728	YBX1
32.373	32.3164	32.402	32.7114	32.5553	32.2177	41.1	50.14	P68104	Elongation factor 1-alpha 1	730	EEF1A1
31.1654	30.3566	30.4545	31.5093	32.3713	31.6439	62.3	49.924	P68366	Tubulin alpha-4A chain	732	TUBA4A
33.3343	33.3031	33.4745	33.8083	34.0818	33.8946	65.2	49.83	P68371	Tubulin beta-4B chain	734	TUBB4B
24.8512	24.865	24.9911	25.3468	25.1214	25.2816	9	40.587	A0A2R8YDP2	Casein kinase II subunit alpha	735	CSNK2A1
NaN	NaN	NaN	24.8631	24.6537	24.3834	12.2	25.569	P68402	Platelet-activating factor acetylhydrolase IB subunit beta	736	PAFAH1B2

34.5896	34.2797	34.3047	33.8402	34.6541	34.5576	25.8	14.914	K7EK07	Histone H3 (Fragment)	737	H3F3B
31.1321	31.0129	31.4741	31.7515	33.7786	31.7567	36.7	15.998	P68871	Hemoglobin subunit beta	738	HBB
34.2658	34.6107	34.5939	34.616	34.8988	34.5039	50.7	15.257	P69905	Hemoglobin subunit alpha	739	HBA1
30.8879	31.1607	30.9945	30.8049	31.1044	31.0489	54.7	52.717	P78371-2	Isoform 2 of T-complex protein 1 subunit beta	741	CCT2
26.7676	26.1421	26.0373	26.3235	NaN	NaN	8.5	23.341	Q5TA02	Glutathione S-transferase omega-1 (Fragment)	742	GSTO1
29.3198	28.3307	29.1424	28.352	28.0483	29.4528	5.6	465.5	P78527-2	Isoform 2 of DNA-dependent protein kinase catalytic subunit	743	PRKDC
27.1432	27.1137	27.5936	27.6375	27.2021	27.1774	19.3	41.75	P78539-4	Isoform 4 of Sushi repeat-containing protein SRPX	744	SRPX
29.5591	29.3625	29.2673	29.679	29.6971	29.3366	24.5	48.607	P83110	Serine protease HTRA3	745	HTRA3
27.8251	27.6876	27.6067	28.1963	28.3283	28.275	33.1	14.369	C9JXB8	60S ribosomal protein L24	746	RPL24
25.9723	25.3026	24.7604	24.645	25.3322	25.2311	16	12.469	Q969Q0	60S ribosomal protein L36a-like	747	RPL36AL
27.8059	27.8484	27.6158	28.6341	28.6763	28.8048	25.9	23.134	J3QR09	Ribosomal protein L19	751	RPL19
28.8264	28.7619	27.8654	27.8609	27.9119	29.3177	7.9	468.83	P98160	Basement membrane-specific heparan sulfate proteoglycan core protein	752	HSPG2
36.0683	35.8074	36.0981	36.0562	36.3241	36.111	55.6	187.89	Q00610-2	Isoform 2 of Clathrin heavy chain 1	755	CLTC
25.5545	25.628	NaN	25.9001	25.6453	26.4327	9	21.493	Q00765	Receptor expression-enhancing protein 5	756	REEP5
27.0021	26.2147	26.2077	26.5355	26.1762	25.9807	6	36.229	H0YLA4	Sorbitol dehydrogenase	757	SORD
27.6042	27.5444	27.7043	27.9033	27.6021	27.9784	17.3	80.648	A0A1W2PP35	Heterogeneous nuclear ribonucleoprotein U (Fragment)	758	HNRNPU
26.4787	26.2897	26.1861	25.9254	26.3713	26.4315	44.4	15.371	J3KP15	Serine/arginine-rich-splicing factor 2 (Fragment)	760	SRSF2
27.3348	26.821	26.9179	27.245	27.0464	26.7593	17.9	51.83	Q01518-2	Isoform 2 of Adenylyl cyclase-associated protein 1	762	CAP1
27.6084	27.7862	28.0559	28.3231	27.5413	27.3483	18.5	57.293	Q01581	Hydroxymethylglutaryl-CoA synthase, cytoplasmic	763	HMGCS1

28.4022	28.097	28.1548	28.4531	28.2512	28.4935	16.5	85.595	Q01813	ATP-dependent 6-phosphofructokinase, platelet type	764	PFKP
25.1548	24.6819	24.8933	25.2753	25.0865	25.9817	1.9	292.26	Q02388-2	Isoform 2 of Collagen alpha-1(VII) chain	765	COL7A1
29.2894	29.1165	29.2294	29.7625	30.0603	30.1146	48.3	20.762	Q02543	60S ribosomal protein L18a	766	RPL18A
NaN	26.413	27.0415	26.8832	27.1422	26.9229	12.3	40.763	Q02750-2	Isoform 2 of Dual specificity mitogen-activated protein kinase kinase 1	767	MAP2K1
31.775	31.758	31.9856	30.9753	30.5001	29.682	45.5	83.549	Q02809	Procollagen-lysine,2-oxoglutarate 5-dioxygenase 1	769	PLOD1
29.5595	29.2749	29.0346	29.7385	29.7396	30.0514	30.2	32.728	Q02878	60S ribosomal protein L6	770	RPL6
28.5306	28.8103	28.5692	27.7347	27.0957	27.6087	30.3	20.471	Q03135	Caveolin-1	771	CAV1
25.8439	25.4769	25.3029	26.9677	27.0558	27.1757	19.1	36.978	Q03405	Urokinase plasminogen activator surface receptor	772	PLAUR
NaN	24.7379	25.0798	25.1519	24.6926	24.6132	10.9	42.868	Q2TAM5	RELA protein	773	RELA
27.6356	26.9661	27.8258	31.6751	31.0743	31.3721	47.4	36.788	Q04828	Aldo-keto reductase family 1 member C1	777	AKR1C1
26.0317	25.8576	25.6746	26.0776	25.1359	24.6977	13.8	28.218	Q04917	14-3-3 protein eta	778	YWHAH
26.29	25.6865	25.9342	26.5647	26.5023	26.6046	9.6	94.016	A0A0D9SFB1	Dynamin-1	780	DNM1
26.0545	26.4997	26.8151	25.7775	26.7358	26.4348	8	42.316	Q5T760	Serine/arginine-rich-splicing factor 11 (Fragment)	782	SRSF11
28.98	29.3653	28.8477	29.0779	29.3268	29.5639	6.6	75.077	A0A087WW43	Inter-alpha-trypsin inhibitor heavy chain H3	783	ITIH3
26.8717	26.0959	26.4579	26.5066	26.6534	26.8168	15	76.758	Q06210-2	Isoform 2 of Glutamine--fructose-6-phosphate aminotransferase [isomerizing] 1	784	GFPT1
29.58	29.7394	29.6623	30.0501	29.995	30.0953	65.8	22.11	Q06830	Peroxiredoxin-1	786	PRDX1
29.3032	29.2913	29.1036	29.5473	29.801	29.6192	39	18.091	Q07020-2	Isoform 2 of 60S ribosomal protein L18	787	RPL18
28.3982	28.0591	27.5571	28.5881	28.7215	28.982	4.9	504.6	Q07954	Prolow-density lipoprotein receptor-related protein 1	791	LRP1

28.3432	28.4769	28.4058	28.8774	28.4092	28.3313	16.1	140.96	Q08211	ATP-dependent RNA helicase A	793	DHX9
26.0354	25.5586	NaN	25.3451	25.8498	25.5249	8.7	21.932	C9JH92	Quinone oxidoreductase (Fragment)	794	CRYZ
35.4374	35.5822	35.3634	35.2631	34.7962	35.0113	46.5	65.33	Q08380	Galectin-3-binding protein	795	LGALS3BP
34.0801	34.1329	33.8627	33.4464	33.3959	33.8024	70.5	43.104	Q08431	Lactadherin	796	MFGE8
26.7015	26.7167	26.5706	26.807	26.658	26.9188	15.3	20.313	A0A2R8Y484	Leukocyte surface antigen CD47 (Fragment)	797	CD47
26.8097	27.5477	26.7338	26.2042	26.1357	26.0736	13.7	18.394	Q10589-2	Isoform 2 of Bone marrow stromal antigen 2	798	BST2
25.7234	25.7567	25.7807	NaN	NaN	25.4002	12.5	31.646	A0A0U1RQV3	EGF-containing fibulin-like extracellular matrix protein 1 (Fragment)	799	EFEMP1
26.7961	27.0696	27.0449	27.2996	26.7199	26.4112	13.1	84.861	B4DLR2	Prolyl endopeptidase FAP	802	FAP
26.3092	26.493	26.3286	26.6202	26.5144	26.9051	18.6	26.76	A8MU58	Aminoacyl tRNA synthase complex-interacting multifunctional protein 2	807	AIMP2
29.1628	29.3407	29.2738	29.4356	29.6152	29.9251	27	100.2	Q13200	26S proteasome non-ATPase regulatory subunit 2	808	PSMD2
26.4348	26.5635	26.3812	26.8554	26.8553	26.5764	7.4	79.473	Q13263-2	Isoform 2 of Transcription intermediary factor 1-beta	809	TRIM28
24.8585	25.2682	24.7956	NaN	NaN	24.9973	2.9	89.763	Q13308-5	Isoform 5 of Inactive tyrosine-protein kinase 7	811	PTK7
27.6881	27.7428	28.1003	28.282	27.947	27.8624	33.2	36.501	Q13347	Eukaryotic translation initiation factor 3 subunit I	813	EIF3I
31.3101	30.8741	31.3548	31.3804	31.8043	31.5698	44.5	51.419	Q13418	Integrin-linked protein kinase	815	ILK
28.4468	28.3164	28.6693	28.4873	28.5593	28.7072	46.2	50.432	Q13509	Tubulin beta-3 chain	817	TUBB3
25.5783	25.5209	25.3487	25.6417	25.5289	25.5209	8.3	55.102	Q13547	Histone deacetylase 1	818	HDAC1
NaN	NaN	24.9675	24.9135	24.7921	24.9717	14.9	29.666	E9PKL9	GDP-L-fucose synthase (Fragment)	820	TSTA3
27.6546	27.1683	27.0348	26.9431	28.7772	28.5408	24.3	31.895	Q13642-1	Isoform 1 of Four and a half LIM domains protein 1	821	FHL1
27.9014	28.495	28.2474	28.7202	27.7307	28.1458	22.8	63.664	Q13740-2	Isoform 2 of CD166 antigen	822	ALCAM
26.4598	26.5237	27.2014	26.8783	27.0038	27.0209	19.8	48.825	Q5STU3	Spliceosome RNA helicase DDX39B	823	DDX39B

26.8797	26.4789	26.8553	26.7406	27.141	26.982	48.5	49.906	Q13885	Tubulin beta-2A chain	824	TUBB2A
26.4523	NaN	26.8293	26.8519	26.8655	26.914	28.8	12.553	D6RF44	Heterogeneous nuclear ribonucleoprotein D0 (Fragment)	825	HNRNPD
27.8599	27.3383	27.5744	27.0428	27.0948	27.5478	18.5	49.204	A0A1W2PPX5	Lysosome membrane protein 2	826	SCARB2
29.5242	30.0046	29.307	26.5789	26.1267	27.2343	26	140.86	Q14112-2	Isoform 2 of Nidogen-2	827	NID2
24.0285	25.1945	24.6998	24.4383	25.309	25.3246	2.8	97.44	Q14118	Dystroglycan	828	DAG1
28.2168	28.2086	28.4715	28.7843	28.7335	28.5518	14.6	162.63	Q14152-2	Isoform 2 of Eukaryotic translation initiation factor 3 subunit A	829	EIF3A
26.1096	25.9017	26.0419	26.1543	26.1741	26.0301	8.9	73.91	Q14195-2	Isoform LCRMP-4 of Dihydropyrimidinase-related protein 3	831	DPYSL3
31.8752	31.701	31.6513	32.0596	32.5118	32.3613	33.2	532.4	Q14204	Cytoplasmic dynein 1 heavy chain 1	832	DYNC1H1
27.5552	27.2209	27.5587	28.0013	27.2722	27.2437	38.6	46.402	Q14240	Eukaryotic initiation factor 4A-II	833	EIF4A2
26.5452	25.9339	26.097	25.8558	25.9963	26.2808	2.2	111.18	Q14289-2	Isoform 2 of Protein-tyrosine kinase 2-beta	835	PTK2B
25.6281	25.7883	25.5982	26.1005	25.8599	25.8718	4.6	287.28	Q14315-2	Isoform 2 of Filamin-C	836	FLNC
26.4526	26.2383	26.5443	26.4644	26.5668	NaN	27.8	26.698	A0A0U1RQH7	RNA-binding protein 39 (Fragment)	839	RBM39
27.472	27.7347	27.6275	27.0491	26.8461	26.5423	4.2	506.27	Q14517	Protocadherin Fat 1	840	FAT1
28.1383	27.103	27.0252	27.7851	27.5022	27.5365	6.2	59.864	Q14520-2	Isoform 2 of Hyaluronan-binding protein 2	841	HABP2
28.6422	28.0897	27.7779	27.8637	28.7358	28.7518	2.1	93.851	Q14624-4	Isoform 4 of Inter-alpha-trypsin inhibitor heavy chain H4	842	ITIH4
29.7338	29.5081	29.7567	28.7696	28.1588	27.6733	25.4	109.44	Q14697-2	Isoform 2 of Neutral alpha-glucosidase AB	843	GANAB
32.704	32.5254	32.3652	32.019	32.2172	32.3968	48.6	99.326	Q14764	Major vault protein	845	MVP
27.6	27.0986	27.1952	27.7743	27.2087	26.8682	20.5	8.3237	F6XGT7	Prostaglandin reductase 1	846	PTGR1
27.4617	27.3531	27.5837	27.6807	27.3003	27.4154	13.4	97.169	Q14974	Importin subunit beta-1	847	KPNB1
27.3481	27.7652	27.754	27.9641	27.7023	27.9989	29.6	45.531	Q15008	26S proteasome non-ATPase regulatory subunit 6	849	PSMD6

28.5675	28.6547	28.8081	29.3175	28.9662	28.709	40.2	36.94	B5MCX3	Septin-2	850	SEPTIN2
NaN	24.2731	NaN	24.5595	24.4931	24.6335	3.7	105.38	Q15029-2	Isoform 2 of 116 kDa U5 small nuclear ribonucleoprotein component	851	EFTUD2
27.4868	27.7385	27.7216	27.0066	26.8326	26.6204	29	17	E5RIP4	Zinc transporter ZIP14 (Fragment)	852	SLC39A14
NaN	26.7412	25.4703	24.6603	24.862	24.6845	41.5	87.253	Q15063-3	Isoform 3 of Periostin	855	POSTN
32.5532	32.1864	31.9703	30.6073	30.5456	30.8034	43.1	90.423	Q15063-5	Isoform 5 of Periostin	856	POSTN
28.1549	28.4467	27.937	24.6457	24.392	25.0413	23.6	47.972	Q15113	Procollagen C-endopeptidase enhancer 1	858	PCOLCE
26.0111	25.9014	26.4394	25.8658	25.9927	NaN	1.1	531.78	Q15149	Plectin	859	PLEC
28.5781	28.5282	28.6123	28.9471	29.1146	28.6252	41	16.476	Q15185-4	Isoform 4 of Prostaglandin E synthase 3	861	PTGES3
NaN	26.0176	25.8356	26.2696	25.9282	25.6989	18.6	29.508	C9JYS8	Non-POU domain-containing octamer-binding protein (Fragment)	862	NONO
27.2439	27.2858	27.0921	27.444	27.277	27.4638	15.2	37.497	Q15365	Poly(rC)-binding protein 1	864	PCBP1
27.3382	27.3639	27.4521	27.8542	27.5078	27.7472	33.8	16.997	F8VXH9	Poly(rC)-binding protein 2 (Fragment)	865	PCBP2
NaN	NaN	NaN	25.4807	25.4019	25.4017	55.4	6.968	R4GMY8	Elongin-C	866	ELOC
27.2803	26.7078	27.5051	26.8862	27.4675	26.6799	22.3	57.455	Q15389-2	Isoform 2 of Angiopoietin-1	868	ANGPT1
25.4359	25.4388	25.4588	26.7519	25.3579	25.5993	5.8	135.58	Q15393	Splicing factor 3B subunit 3	869	SF3B3
NaN	23.8773	24.8584	23.8956	24.9132	24.9677	6.7	25.545	Q15404-2	Isoform 2 of Ras suppressor protein 1	870	RSU1
27.3229	27.5838	27.3514	27.6826	26.794	26.7557	12	33.252	Q15493	Regucalcin	872	RGN
29.5758	29.951	30.0958	29.9124	29.7046	29.1382	29.9	30.691	Q15555-4	Isoform 4 of Microtubule-associated protein RP/EB family member 2	873	MAPRE2
33.6561	33.3443	33.6485	32.9595	32.7274	33.0485	52.3	74.68	Q15582	Transforming growth factor-beta-induced protein ig-h3	874	TGFBI
29.8899	29.6996	29.9982	29.6894	29.1312	29.1011	11.6	56.598	Q15758	Neutral amino acid transporter B(0)	875	SLC1A5
25.9868	26.5013	26.0803	26.2386	26.2464	26.2684	33	11.309	Q15836	Vesicle-associated membrane protein 3	876	VAMP3
28.5222	28.3696	28.7461	28.9588	28.5892	28.4228	39.9	24.488	Q15907	Ras-related protein Rab-11B	877	RAB11B

28.0366	27.642	27.8131	28.6001	27.8069	27.7615	26.4	48.715	E7ES33	Septin-7	879	SEPTIN7
27.5017	26.7965	26.3568	24.5023	25.3696	NaN	24.4	28.86	Q16270-2	Isoform 2 of Insulin-like growth factor-binding protein 7	881	IGFBP7
29.1681	28.9883	29.2556	29.3691	29.2741	28.375	20.8	86.254	Q16394	Exostosin-1	882	EXT1
26.3842	26.0719	26.6791	26.7493	26.4216	26.7176	12.8	51.311	Q16401-2	Isoform 2 of 26S proteasome non-ATPase regulatory subunit 5	883	PSMD5
30.0892	29.8069	29.9549	30.4889	29.8485	29.7139	53	58.162	Q16555-2	Isoform 2 of Dihydropyrimidinase-related protein 2	885	DPYSL2
27.1383	26.723	26.8921	26.9791	26.7763	26.8196	16.3	46.938	E9PC52	Histone-binding protein RBBP7	887	RBBP7
NaN	25.699	NaN	25.9173	26.0713	25.6986	24.2	15.257	Q16629-3	Isoform 3 of Serine/arginine-rich splicing factor 7	889	SRSF7
28.8345	28.7745	28.82	28.4957	28.46	28.3623	26.6	54.529	Q16658	Fascin	891	FSCN1
33.8775	32.9567	32.3127	33.783	32.7138	33.9404	49.6	13.988	Q16777	Histone H2A type 2-C	894	HIST2H2AC
26.2553	25.4368	25.2273	25.7779	26.4002	26.4723	2.2	84.621	Q16853	Membrane primary amine oxidase	895	AOC3
28.7239	28.6061	28.7174	28.5193	27.7176	27.6737	18.4	78.921	Q32P28-4	Isoform 4 of Prolyl 3-hydroxylase 1	899	P3H1
29.1398	29.1774	28.6571	29.7527	29.9625	29.8599	24.5	49.775	Q3ZCM7	Tubulin beta-8 chain	900	TUBB8
26.4073	26.7773	26.984	27.063	26.8076	26.9447	19.8	18.986	Q3ZCW2	Galectin-related protein	901	LGALSL
25.8374	25.7659	26.0998	25.7665	NaN	26.0186	13.8	26.439	Q53H96-2	Isoform 2 of Pyrroline-5-carboxylate reductase 3	902	PYCR3
27.8173	27.7659	27.7783	28.1705	27.7376	27.3727	18.6	44.348	Q58FF8	Putative heat shock protein HSP 90-beta 2	903	HSP90AB2P
25.4098	25.3711	NaN	25.7375	25.7458	25.6294	9.4	61.206	Q5SSJ5	Heterochromatin protein 1-binding protein 3	905	HP1BP3
29.2413	28.8749	28.7634	29.2838	29.9376	29.7366	9.1	571.85	Q5T4S7-3	Isoform 3 of E3 ubiquitin-protein ligase UBR4	906	UBR4
26.5033	26.8552	26.621	27.318	26.7784	26.9749	14.9	41.767	A0A0C4DGH0	CD276 antigen	909	CD276
27.3914	28.4102	27.9271	26.05	NaN	NaN	11.2	83.998	Q68BL8	Olfactomedin-like protein 2B	910	OLFML2B

25.1554	25.126	25.1606	25.6727	24.9015	24.844	10.5	34.596	Q96GK7	Fumarylacetoacetate hydrolase domain-containing protein 2A	913	FAHD2A
27.2708	26.6787	25.7442	24.7668	24.7539	25.4804	4	250.53	Q6UVK1	Chondroitin sulfate proteoglycan 4	914	CSPG4
NaN	23.8305	23.8911	23.9591	23.7403	24.7806	2.1	152.66	Q6YHK3-2	Isoform 2 of CD109 antigen	917	CD109
27.7984	27.4283	27.4559	27.8718	27.9266	27.8785	5.8	282.86	Q6ZNJ1-2	Isoform 2 of Neurobeachin-like protein 2	918	NBEAL2
26.1383	26.1464	25.948	25.4531	24.8257	25.1652	14.9	24.486	Q6ZUX7	LHFPL tetraspan subfamily member 2 protein	919	LHFPL2
33.798	33.4893	33.5135	34.0447	34.6089	34.3069	65	50.135	Q71U36	Tubulin alpha-1A chain	921	TUBA1A
26.1907	26.5858	27.2837	27.0529	27.349	26.5645	10.4	102	Q7KZF4	Staphylococcal nuclease domain-containing protein 1	924	SND1
26.1951	26.3185	26.3075	26.6976	26.571	26.3911	21.4	42.502	Q7L2H7	Eukaryotic translation initiation factor 3 subunit M	925	EIF3M
25.9727	NaN	25.6644	26.1201	25.5849	25.5976	7.2	94.466	Q7L576-2	Isoform 2 of Cytoplasmic FMR1-interacting protein 1	926	CYFIP1
25.9046	25.7138	26.0383	26.1633	26.7261	26.2842	9.5	69.248	Q7LDG7	RAS guanyl-releasing protein 2	927	RASGRP2
30.5383	30.5169	30.5842	24.6773	25.6852	26.0515	40.7	77.555	Q7Z304	MAM domain-containing protein 2	928	MAMDC2
NaN	26.9714	26.892	27.3946	27.0488	26.7899	23	10.694	J3QS45	L-xylulose reductase (Fragment)	931	DCXR
31.4432	31.5957	31.8236	31.8616	31.4393	31.3582	28.5	75.429	Q86UX7-2	Isoform 2 of Fermitin family homolog 3	934	FERMT3
27.3468	27.2468	27.3307	27.595	27.321	27.7037	15.7	117.89	Q86VP6-2	Isoform 2 of Cullin-associated NEDD8-dissociated protein 1	935	CAND1
25.9563	23.5942	26.3267	25.4284	24.1323	23.5863	11.3	282.39	Q86YZ3	Hornerin	938	HRNR
25.8649	27.0178	27.1103	NaN	25.1048	NaN	7.5	100.16	Q8IWU5-2	Isoform 2 of Extracellular sulfatase Sulf-2	940	SULF2
32.4954	32.1012	32.1533	32.4294	33.7177	NaN	3.7	57.296	Q8IXS2	Dynein regulatory complex subunit 2	943	CCDC65
25.5597	25.7027	25.6264	26.9235	27.8039	27.7083	8.1	79.917	Q8IZ83-3	Isoform 3 of Aldehyde dehydrogenase family 16 member A1	945	ALDH16A1

27.7898	27.6193	27.9979	28.3695	27.7917	27.4261	24.8	47.576	Q8NBZ7	UDP-glucuronic acid decarboxylase 1	951	UXS1
28.8679	29.306	28.8065	28.64	28.7561	28.413	22.6	30.69	Q8NG11	Tetraspanin-14	954	TSPAN14
26.3885	26.4929	26.4021	26.538	26.2464	26.4091	12.9	53.579	Q8WTV0-4	Isoform 4 of Scavenger receptor class B member 1	960	SCARB1
28.4791	28.6365	29.0483	28.9756	28.154	28.1473	26.9	37.807	Q8WUA8	Tsukushin	961	TSKU
28.1752	28.9208	28.4687	29.5049	28.6006	28.4161	20.1	153	Q8WUJ3	Cell migration-inducing and hyaluronan-binding protein	962	CEMIP
29.2268	28.9351	29.2982	29.2314	29.0373	28.7621	26.7	96.022	Q8WUM4	Programmed cell death 6-interacting protein	963	PDCD6IP
28.3893	28.3538	28.2721	28.1119	27.6685	28.0689	10.9	73.011	Q8WWI5-3	Isoform 3 of Choline transporter-like protein 1	964	SLC44A1
25.0247	NaN	NaN	24.9918	25.2519	25.674	4.2	60.021	Q8WWZ8	Oncoprotein-induced transcript 3 protein	965	OIT3
28.5069	27.5019	27.6992	27.7628	28.4871	28.3935	28.2	22.487	Q92522	Histone H1x	967	H1FX
31.7791	32.0019	31.5501	31.208	31.3343	31.3882	39.7	165.27	Q92626	Peroxidasin homolog	969	PXDN
25.255	25.2641	25.3495	25.1625	24.9605	NaN	17.1	22.642	K7EKE8	Nectin-2 (Fragment)	970	NECTIN2
31.0099	30.158	30.5358	29.3827	30.2586	30.431	35	51.286	Q92743	Serine protease HTRA1	971	HTRA1
26.7138	26.8367	27.1504	27.4565	27.2123	27.1111	14.8	72.371	Q92841-1	Isoform 2 of Probable ATP-dependent RNA helicase DDX17	974	DDX17
26.3963	26.4447	26.5772	27.0517	26.5835	26.2839	1.4	290.46	Q93008-1	Isoform 2 of Probable ubiquitin carboxyl-terminal hydrolase FAF-X	978	USP9X
28.0501	28.0024	28.2581	28.3683	28.0466	27.2062	17.8	82.254	Q93063	Exostosin-2	979	EXT2
31.7953	31.8047	31.8254	31.0604	31.0901	31.1672	50.4	65.033	Q969P0	Immunoglobulin superfamily member 8	983	IGSF8
25.8443	25.2862	25.5442	26.0637	26.1022	25.7382	10.2	49.389	Q96CW1-2	Isoform 2 of AP-2 complex subunit mu	988	AP2M1
NaN	25.7011	25.7115	25.4761	25.5883	25.0377	8.6	21.522	Q96DD7	Protein shisa-4	989	SHISA4
25.407	25.4882	25.6235	25.85	NaN	25.2321	33.6	12.976	G3V5E4	Glucosamine 6-phosphate N-acetyltransferase	991	GNPNAT1
26.6488	26.4963	27.0476	26.9246	26.0718	26.0466	14.1	28.05	F5GYN4	Ubiquitin thioesterase OTUB1	992	OTUB1
25.1551	24.2117	25.1255	24.5097	25.0278	25.1318	5.4	46.228	Q9Y383-3	Isoform 3 of Putative RNA-binding protein Luc7-like 2	995	LUC7L2

29.9848	29.6026	29.945	29.594	29.1542	29.281	10.4	122.2	Q96KG7	Multiple epidermal growth factor-like domains protein 10	997	MEGF10
27.7741	27.6896	27.9046	28.4275	27.1504	26.8049	20.7	43.833	Q96KP4-2	Isoform 2 of Cytosolic non-specific dipeptidase	998	CNDP2
27.7842	27.7148	27.7573	28.365	27.9228	28.1391	22.4	91.706	Q96QK1	Vacuolar protein sorting-associated protein 35	100 3	VPS35
26.0472	25.7896	26.6946	26.0483	26.6119	25.8882	8.6	82.682	Q96TA1-2	Isoform 2 of Niban-like protein 1	100 4	FAM129B
27.4039	28.1414	27.7684	28.1438	28.363	28.4948	18.2	102.26	Q99460-2	Isoform 2 of 26S proteasome non-ATPase regulatory subunit 1	100 6	PSMD1
26.6747	NaN	26.7788	27.408	26.6629	26.6747	14.5	41.92	Q99536	Synaptic vesicle membrane protein VAT-1 homolog	100 7	VAT1
30.1099	31.4636	31.0101	29.8613	30.7102	31.3031	24.2	333.2	D6RGG3	Collagen alpha-1(XII) chain	100 9	COL12A1
26.0119	25.9896	25.8301	26.4069	26.0886	26.1944	10.6	75.775	Q99805	Transmembrane 9 superfamily member 2	101 0	TM9SF2
29.851	29.9361	29.9835	29.642	29.9456	29.9779	33.5	54.804	Q99832-3	Isoform 3 of T-complex protein 1 subunit eta	101 2	CCT7
27.2506	27.938	28.0632	28.2525	28.2039	27.8943	24.3	37.709	E9PKG1	Protein arginine N-methyltransferase 1	101 3	PRMT1
25.5381	25.4008	NaN	26.2327	25.9265	25.9615	4.1	140.13	I3L1L3	Myb-binding protein 1A (Fragment)	101 8	MYBBP1A
25.8931	26.4485	26.5617	NaN	25.0051	NaN	8.1	28.949	A0A087W W53	Coronin	101 9	CORO1B
27.184	26.6295	26.7783	26.6158	NaN	NaN	4.9	56.32	B4DKD1	Protein tweety homolog	102 0	TTYH2
26.5912	27.0737	26.9686	27.034	26.6866	26.8346	38.3	49.857	Q9BUF5	Tubulin beta-6 chain	102 3	TUBB6
27.2985	26.3269	25.8944	25.8355	26.4947	27.4192	15.9	26.994	Q9BXJ4	Complement C1q tumor necrosis factor-related protein 3	102 5	C1QTNF3
NaN	25.2311	25.4209	25.5543	25.3501	25.156	3.7	103.13	Q9BZQ8	Protein Niban	102 9	FAM129A

29.4233	29.0223	28.8792	28.2737	28.0883	28.3675	8.8	54.139	Q9C0H2-2	Isoform 2 of Protein tweety homolog 3	1030	TTYH3
26.221	26.5536	26.9602	26.4921	26.7842	26.3368	31.3	22.171	Q9H0U4	Ras-related protein Rab-1B	1031	RAB1B
25.4382	25.3143	25.3545	25.6099	25.5084	25.8711	7.2	57.547	B4DGG1	Protein FAM234A	1032	FAM234A
27.2067	27.1732	27.6113	26.1	25.884	26.6046	32.9	40.323	Q9H1J7	Protein Wnt-5b	1033	WNT5B
25.1617	25.3534	25.9159	25.6159	NaN	25.3338	4.8	72.595	Q9H4A4	Aminopeptidase B	1039	RNPEP
32.3383	31.9755	32.0026	33.0186	33.7366	33.3288	30.2	50.326	Q9H4B7	Tubulin beta-1 chain	1040	TUBB1
30.7992	30.4887	31.0566	30.9964	31.1587	30.9511	50.6	60.626	Q9H4M9	EH domain-containing protein 1	1041	EHD1
NaN	NaN	NaN	24.7693	25.1984	25.2371	15.9	24.605	Q5VXN0	Ribosome production factor 2 homolog (Fragment)	1042	RPF2
28.6435	28.6887	28.767	29.156	29.418	29.2126	28.1	37.538	Q9HBI1-3	Isoform 3 of Beta-parvin	1047	PARVB
29.8885	29.8782	29.8697	30.8567	30.8608	30.6207	27	37.144	F8W914	Reticulon	1052	RTN4
27.2812	27.6464	27.5406	28.2682	27.5245	27.1591	23.4	62.138	Q5T6H7	Xaa-Pro aminopeptidase 1	1053	XPNPEP1
NaN	24.8494	24.8782	25.2306	24.6761	24.7695	4.3	79.656	Q9NR30-2	Isoform 2 of Nucleolar RNA helicase 2	1056	DDX21
26.354	26.1007	26.75	26.499	26.4893	26.243	15.2	22.367	Q9NR31	GTP-binding protein SAR1a	1057	SAR1A
26.7005	26.712	26.6613	27.5886	27.3881	27.0896	22.3	40.307	Q9NR45	Sialic acid synthase	1058	NANS
27.1304	27.704	27.7234	26.0858	26.2415	25.5395	23.2	38.982	B4DNG0	cDNA FLJ58142, highly similar to Olfactomedin-like protein 3	1060	OLFML3
27.1921	27.0131	27.0698	27.654	27.2048	27.124	21.2	66.115	Q9NSD9	Phenylalanine--tRNA ligase beta subunit	1062	FARSB
27.2729	28.1249	27.3705	NaN	NaN	26.1825	7.2	292.26	Q9NT68-2	Isoform 2 of Teneurin-2	1063	TENM2

26.566	26.5995	26.8206	27.0194	27.0316	26.2089	16.7	44.743	Q9NTK5	Obg-like ATPase 1	1065	OLA1
25.7563	25.9693	25.939	26.4366	26.8702	26.295	11.5	49.005	D6RGI3	Septin 11, isoform CRA_b	1066	SEPTIN11
24.4445	25.3983	24.9211	NaN	NaN	NaN	10.3	18.373	Q9NVM1	Protein eva-1 homolog B	1067	EVA1B
29.2031	28.815	29.1534	29.0455	29.4986	29.0069	42.6	60.886	Q9NZN3	EH domain-containing protein 3	1071	EHD3
26.064	25.7514	26.0895	26.4367	25.8919	26.0436	12.2	61.161	Q9NZN4	EH domain-containing protein 2	1072	EHD2
NaN	24.7797	24.6062	25.5659	25.1882	25.1865	6.4	27.893	Q9P0L0	Vesicle-associated membrane protein-associated protein A	1073	VAPA
NaN	25.4366	25.6163	26.1316	26.259	26.2157	9.2	23.096	Q9P126-2	Isoform 2 of C-type lectin domain family 1 member B	1074	CLEC1B
25.9615	25.9681	26.172	26.3393	26.0229	26.0869	11.9	56.084	Q9P258	Protein RCC2	1075	RCC2
30.0738	30.3385	30.1749	29.979	29.3047	29.5502	21.7	300.95	Q9P273	Teneurin-3	1076	TENM3
34.8664	34.855	34.8135	34.0545	33.8719	34.0162	47.3	98.555	Q9P2B2	Prostaglandin F2 receptor negative regulator	1077	PTGFRN
27.6507	27.5453	27.3785	27.7753	27.2558	27.7672	12.4	134.46	Q9P2J5	Leucine--tRNA ligase, cytoplasmic	1079	LARS
28.5826	27.8674	27.7626	28.8689	29.62	29.273	2.9	124.45	Q9UGM3-9	Isoform 9 of Deleted in malignant brain tumors 1 protein	1087	DMBT1
26.5717	26.2341	26.8495	26.9992	26.5586	26.5128	27.2	22.217	K7EK18	Septin-9 (Fragment)	1088	SEPTIN9
NaN	24.4621	24.3732	24.8106	24.3144	24.6732	2.7	147.44	Q9UHN6-2	Isoform 2 of Cell surface hyaluronidase	1090	CEMIP2
24.8393	24.8724	25.1475	25.2061	24.524	24.3259	13.3	21.977	H7C286	N-acetyl-D-glucosamine kinase	1094	NAGK
27.248	27.0251	27.0118	26.273	26.387	26.6846	5.9	192.59	Q9UKK3	Protein mono-ADP-ribosyltransferase PARP4	1096	PARP4
27.0071	27.7397	28.3617	24.8541	NaN	NaN	22.7	57.104	Q9UKU9	Angiopoietin-related protein 2	1097	ANGPTL2

25.5075	25.3606	25.5991	25.7526	25.6856	25.4065	11.6	33.807	C9JYX9	Procollagen C-endopeptidase enhancer 2	1099	PCOLCE2
NaN	24.911	NaN	25.1495	25.5015	25.1162	5.6	44.992	E7EMB6	Aspartyl aminopeptidase	1101	DNPEP
24.8295	24.7619	24.305	24.1724	23.6903	NaN	1.9	94.131	Q9ULF5	Zinc transporter ZIP10	1102	SLC39A10
26.4334	25.3875	25.3849	26.9545	25.2721	NaN	2.2	147.46	Q9ULI3	Protein HEG homolog 1	1103	HEG1
28.0554	27.9159	28.2593	28.7039	28.2551	27.9186	20.5	53.248	Q9ULV4	Coronin-1C	1104	CORO1C
27.8257	28.0513	27.8725	27.9655	27.2963	27.5756	21	55.18	Q9UMS4	Pre-mRNA-processing factor 19	1105	PRPF19
25.4191	NaN	25.365	26.2713	25.0016	24.9923	16.9	33.57	Q9UNH7-2	Isoform 2 of Sorting nexin-6	1108	SNX6
28.583	28.4457	28.3615	28.5416	28.5177	28.7705	36.2	42.945	Q9UNM6	26S proteasome non-ATPase regulatory subunit 13	1110	PSMD13
NaN	23.4853	23.4754	24.7181	23.5337	24.1744	4.6	55.422	Q9UPY5	Cystine/glutamate transporter	1114	SLC7A11
27.7405	27.4902	27.5218	27.8845	27.9021	27.3307	28.2	38.059	Q9UQ80-2	Isoform 2 of Proliferation-associated protein 2G4	1117	PA2G4
NaN	24.4115	24.9697	25.1599	24.9358	25.0111	5.9	66.784	Q9Y223-5	Isoform 5 of Bifunctional UDP-N-acetylglucosamine 2-epimerase/N-acetylmannosamine kinase	1118	GNE
28.2067	28.1509	28.2135	28.518	28.7675	29.0614	35	51.156	Q9Y230	RuvB-like 2	1119	RUVBL2
25.484	25.4481	25.5231	26.4064	26.1561	25.7084	11.1	35.694	Q9Y240	C-type lectin domain family 11 member A	1120	CLEC11A
28.2545	27.9265	28.1114	28.7417	28.2229	28.0643	25.2	66.726	Q9Y262	Eukaryotic translation initiation factor 3 subunit L	1121	EIF3L
28.2021	28.2089	28.1537	28.7295	28.7083	28.8787	33.8	50.227	Q9Y265	RuvB-like 1	1122	RUVBL1
27.6969	27.5033	27.7829	28.4094	27.9155	27.7754	26.2	57.563	Q9Y285	Phenylalanine--tRNA ligase alpha subunit	1123	FARSA
25.8522	25.9049	25.7522	27.1223	28.1815	27.5767	31.4	35.23	Q9Y315	Deoxyribose-phosphate aldolase	1129	DERA

27.2587	27.5842	27.4104	27.9104	27.4046	27.7705	39.7	38.438	Q9Y3F4	Serine-threonine kinase receptor-associated protein	1132	STRAP
27.6115	27.3075	27.4998	28.3621	27.7126	27.5205	23	55.21	Q9Y3I0	tRNA-splicing ligase RtcB homolog	1133	RTCB
27.6538	27.3094	27.0855	28.0062	27.802	27.984	21	12.254	Q9Y3U8	60S ribosomal protein L36	1134	RPL36
33.0755	32.8297	32.825	33.2771	33.2154	33.0662	41.6	269.76	Q9Y490	Talin-1	1135	TLN1
29.2435	29.2315	28.8793	28.665	28.6307	29.5781	24.9	86.724	Q9Y4K0	Lysyl oxidase homolog 2	1136	LOXL2
28.6511	28.6757	28.7426	28.6817	27.8972	27.981	22.1	104.78	A0A087X054	Hypoxia up-regulated protein 1	1137	HYOU1
26.6252	26.7627	26.9796	26.8038	26.7388	26.8489	15.8	39.834	Q9Y5P6	Mannose-1-phosphate guanyltransferase beta	1138	GMPPB
30.2028	30.317	30.3367	30.8713	30.072	29.5741	34.4	56.794	Q9Y5X9	Endothelial lipase	1139	LIPG
26.2532	25.8977	NaN	25.9481	27.1852	26.6691	8.8	97.717	Q9Y678	Coatomer subunit gamma-1	1143	COPG1
25.8545	26.0069	26.0512	26.4054	25.6845	25.1641	21.7	28.772	Q9Y696	Chloride intracellular channel protein 4	1144	CLIC4
33.0233	33.1163	32.9644	29.0736	28.5236	28.7733	51.3	106.69	Q9Y6C2	EMILIN-1	1145	EMILIN1
27.8518	27.5216	27.4633	27.9725	27.6867	27.7353	15.7	56.578	Q9Y6G9	Cytoplasmic dynein 1 light intermediate chain 1	1146	DYNC1LI1
30.3945	30.8122	30.1788	30.2181	30.1207	31.1087	25.7	201.82	A0A0A0MTC7	Laminin subunit alpha-4	1148	LAMA4
28.8246	28.6376	28.9557	28.9832	29.1767	29.3218	23.6	27.168	H3BN98	Uncharacterized protein (Fragment)	1159	Uncharacterized
30.8089	30.5028	30.5316	30.8799	31.2078	30.9126	25	15.43	Q5TEC6	Histone H3	1161	HIST2H3PS2

Supplemental PCA analysis for Figure 4

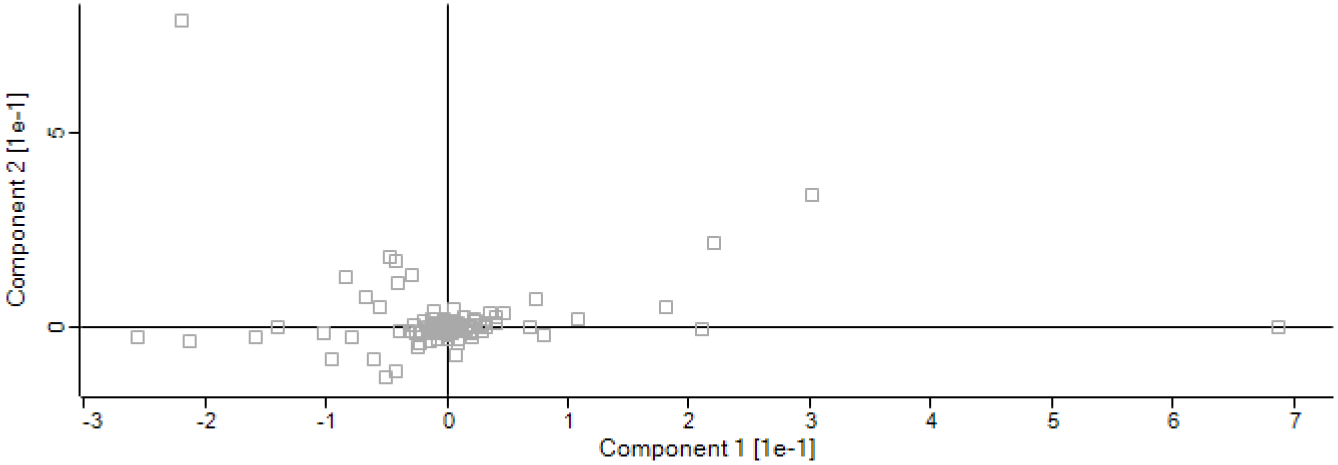

Data matrix of PCA analysis

Component 1	Component 2	Component 3	Component 4	Component 5	T: UNIPROT	T: Protein names	T: id
-4.82E-05	0.000190472	2.73E-05	-0.000274811	0.000350752	H0Y8S8	Ubiquitin-like modifier-activating enzyme 6 (Fragment)	11
0.00258146	-0.000214176	-0.00134179	-0.000325946	0.00123104	A1L4H1-2	Isoform 2 of Soluble scavenger receptor cysteine-rich domain-containing protein SSC5D	12
-0.000556794	0.00220131	0.00031542	-0.00317603	0.00405368	A6NHL2-2	Isoform 2 of Tubulin alpha chain-like 3	13
7.29E-05	0.000480408	0.000165966	0.00128052	-0.00251292	Q49AN9	Small nuclear ribonucleoprotein G	14
-0.000558432	-0.000556809	0.000717079	-0.00219872	0.00222182	Q99613-2	Isoform 2 of Eukaryotic translation initiation factor 3 subunit C	15
-0.000237192	0.000362523	-0.000774949	-0.000147103	-0.000691581	B9A064	Immunoglobulin lambda-like polypeptide 5	16
-1.71E-05	6.77E-05	9.70E-06	-9.76E-05	0.000124619	O00151	PDZ and LIM domain protein 1	17
-0.000178296	0.000704903	0.000101003	-0.00101703	0.00129807	K7EKP8	Cytosolic acyl coenzyme A thioester hydrolase (Fragment)	18
-0.000503189	-0.000854147	-0.000100586	-0.000498626	-0.00129784	O00159-2	Isoform 2 of Unconventional myosin-Ic	19
-3.78E-05	0.000149488	2.14E-05	-0.00021568	0.00027528	H3BUD9	Ras-related protein Rab-27A (Fragment)	20
-0.00144509	0.000492281	0.00234962	-0.00757753	0.00889073	O00231	26S proteasome non-ATPase regulatory subunit 11	21
-0.000516665	0.000692675	0.000218233	-0.00187023	-0.000372182	O00232	26S proteasome non-ATPase regulatory subunit 12	22
-0.000603071	-0.00512607	0.00756368	0.00246344	0.00306071	O00299	Chloride intracellular channel protein 1	23
-0.000245768	-3.69E-05	-0.000157624	-0.000563438	0.0010749	O00303	Eukaryotic translation initiation factor 3 subunit F	24
4.57E-05	0.000248082	3.51E-05	0.000576876	-0.000893636	B8ZZF7	Sulfotransferase	25
0.00019265	-0.00041518	0.00178589	0.00230945	-0.00378291	O00391-2	Isoform 2 of Sulfhydryl oxidase 1	26
-0.000936761	-0.000201957	-0.000372694	0.0017562	0.00162865	O00429-4	Isoform 3 of Dynamin-1-like protein	27
-0.00309608	-0.00138735	0.0028432	-0.000911464	-0.00430416	O00443	Phosphatidylinositol 4-phosphate 3-kinase C2 domain-containing subunit alpha	28

0.0350335	0.0360535	-0.0044624	-0.0152111	-0.02132	O00468-6	Isoform 6 of Agrin	29
-6.13E-05	0.000242379	3.47E-05	-0.000349702	0.000446337	O00469-3	Isoform 3 of Procollagen-lysine,2-oxoglutarate 5-dioxygenase 2	30
-0.000225175	0.000609614	0.00190214	-0.00238136	0.00355712	O00487	26S proteasome non-ATPase regulatory subunit 14	31
0.000111577	-0.001531	0.000804925	0.00205646	-0.00295816	O00560-3	Isoform 3 of Syntenin-1	32
-1.22E-05	0.000179784	0.000290281	0.000900953	3.03E-05	O00567	Nucleolar protein 56	33
-0.00076421	-0.000913726	0.00088189	-0.00225059	-6.12E-05	O00592-2	Isoform 2 of Podocalyxin	34
0.00256329	0.00434639	0.000175746	0.0148097	0.0246746	O00622	Protein CYR61	35
2.66E-05	7.69E-05	-0.000901986	-0.000545334	0.00255201	O00743-2	Isoform 2 of Serine/threonine-protein phosphatase 6 catalytic subunit	36
-2.34E-05	9.26E-05	1.33E-05	-0.000133587	0.000170502	H0YN67	Immunoglobulin superfamily-containing leucine-rich repeat protein (Fragment)	37
0.000654236	-0.000533066	0.000530879	0.00212816	-0.000797228	O14657	Torsin-1B	38
0.00532841	-0.00158838	-0.000697698	0.0139902	-0.0241507	O14672	Disintegrin and metalloproteinase domain-containing protein 10	39
3.20E-05	-0.000208571	-0.00010927	0.000323204	0.000706973	O14744-2	Isoform 2 of Protein arginine N-methyltransferase 5	40
0.00320172	0.000312044	-0.000943831	-0.00079645	0.000645605	E9PEP6	Neuropilin	41
0.00940309	-0.000308496	-0.00584304	0.00426497	0.0246485	E9PN41	Tetraspanin-4 (Fragment)	42
-2.37E-05	0.000762017	0.000873971	-0.00519972	0.000967643	O14818	Proteasome subunit alpha type-7	43
0.000633267	0.00200273	-0.000473331	-0.0019447	-0.00151727	O14828	Secretory carrier-associated membrane protein 3	44
-0.000212646	0.000248765	0.00125884	-0.000705948	-0.00118057	O14929	Histone acetyltransferase type B catalytic subunit	45
-6.32E-05	-0.000441668	-0.000787082	-0.00220022	-0.0011246	P19105	Myosin regulatory light chain 12A	46
-1.47E-05	5.81E-05	8.33E-06	-8.39E-05	0.000107048	F8VW28	Protein phosphatase 1 regulatory subunit 12A (Fragment)	47
0.000466151	-0.00135393	0.00161045	0.00235293	0.000926016	O14980	Exportin-1	48
-0.000536899	0.000400543	-0.000713872	-0.00104404	0.000747454	A0A3F2YNX0	Protein transport protein sec16	49
-6.20E-05	-0.000192863	0.000517016	-0.000613161	-0.000147191	O15031	Plexin-B2	50
-1.20E-05	-1.16E-05	-2.81E-05	7.43E-07	-2.10E-05	O15067	Phosphoribosylformylglycinamidine synthase	51

**-6.79E-05**	**0.000268332**	**3.84E-05**	**-0.000387147**	**0.00049413**	**O15118**	**NPC intracellular cholesterol transporter 1**	**52**
**-0.000166597**	**-0.000432549**	**-0.000299056**	**-0.00331284**	**-0.00051138**	**A0A087WU14**	**Secretory carrier-associated membrane protein (Fragment)**	**53**
**-0.000112482**	**0.000444704**	**6.37E-05**	**-0.000641614**	**0.000818915**	**H3BV04**	**Secretory carrier-associated membrane protein (Fragment)**	**54**
**-0.000112826**	**-0.000481859**	**0.00249873**	**0.00138344**	**-0.00212752**	**O15143**	**Actin-related protein 2/3 complex subunit 1B**	**55**
**2.98E-05**	**-0.00107861**	**0.000278956**	**0.00165598**	**-0.00237335**	**O15144**	**Actin-related protein 2/3 complex subunit 2**	**56**
**0.000239031**	**-0.000909595**	**5.94E-05**	**0.000358682**	**0.00207562**	**O15145**	**Actin-related protein 2/3 complex subunit 3**	**57**
**0.0321092**	**0.0127249**	**-0.00868587**	**0.0200157**	**-0.0266103**	**O15230**	**Laminin subunit alpha-5**	**58**
**-0.00031902**	**-0.000285366**	**0.000424965**	**-0.000866706**	**-0.000285195**	**O15371-2**	**Isoform 2 of Eukaryotic translation initiation factor 3 subunit D**	**59**
**0.000117232**	**-0.000166207**	**0.000285526**	**0.000308872**	**0.000200702**	**O15460-2**	**Isoform IIa of Prolyl 4-hydroxylase subunit alpha-2**	**60**
**-5.89E-05**	**-0.000431556**	**0.000988283**	**0.000847239**	**-0.00105771**	**O43143**	**Pre-mRNA-splicing factor ATP-dependent RNA helicase DHX15**	**61**
**-0.000617287**	**0.000319916**	**-0.00090387**	**-0.00103556**	**0.000601264**	**O43149**	**Zinc finger ZZ-type and EF-hand domain-containing protein 1**	**62**
**-7.62E-06**	**3.01E-05**	**4.32E-06**	**-4.35E-05**	**5.55E-05**	**O43150-2**	**Isoform 2 of Arf-GAP with SH3 domain, ANK repeat and PH domain-containing protein 2**	**63**
**-0.00304185**	**0.00393581**	**0.00220756**	**0.000989243**	**0.00442408**	**A0A286YF22**	**D-3-phosphoglycerate dehydrogenase**	**64**
**-0.000928711**	**0.0010411**	**-0.00225657**	**-0.00189614**	**0.00294625**	**O43242**	**26S proteasome non-ATPase regulatory subunit 3**	**65**
**-0.000271071**	**0.00107169**	**0.00015356**	**-0.00154622**	**0.0019735**	**O43286**	**Beta-1,4-galactosyltransferase 5**	**66**
**-0.000222258**	**0.000878707**	**0.000125907**	**-0.00126779**	**0.00161813**	**O43294-2**	**Isoform 2 of Transforming growth factor beta-1-induced transcript 1 protein**	**67**
**-0.000248105**	**8.50E-05**	**-6.72E-05**	**-0.000690291**	**0.000539392**	**O43324**	**Eukaryotic translation elongation factor 1 epsilon-1**	**68**
**-0.000462238**	**-0.00129915**	**0.000809147**	**-0.000776637**	**-0.0002215**	**H3BLU7**	**Aflatoxin B1 aldehyde reductase member 2 (Fragment)**	**69**

0.00263598	0.000301529	-0.00163529	0.00558363	-0.00742731	A0A087WZU5	Tetraspanin-6	70
-0.000299231	-0.000386489	0.000895851	-0.00145776	-0.00278469	J3QT28	Mitotic checkpoint protein BUB3 (Fragment)	71
-0.000189174	-0.00011444	-0.000220587	-0.00103644	0.00198642	O43747	AP-1 complex subunit gamma-1	72
-7.77E-05	0.000307207	4.40E-05	-0.000443235	0.000565716	K7ELD9	Synaptogyrin-2	73
-0.00159895	-0.0023752	0.00346507	0.00059081	-0.00257015	O43776	Asparagine--tRNA ligase, cytoplasmic	74
0.000789103	0.000114474	-0.000281328	0.00182473	0.000333641	E9PHS0	Glutathione S-transferase LANCL1 (Fragment)	75
0.220057	0.215815	-0.172909	-0.310405	-0.0263039	O43854-2	Isoform 2 of EGF-like repeat and discoidin I-like domain-containing protein 3	76
-0.000391634	-0.000378244	-0.000917036	2.42E-05	-0.000685497	O60218	Aldo-keto reductase family 1 member B10	77
-0.000113191	0.000447507	6.41E-05	-0.000645659	0.000824078	G3V1B8	Sjoegren syndrome/scleroderma autoantigen 1	78
-8.47E-05	-6.52E-05	5.24E-05	0.000238953	0.000804202	O60506-4	Isoform 4 of Heterogeneous nuclear ribonucleoprotein Q	79
0.00102098	0.00076201	0.000995454	0.0026059	-0.000433722	O60568	Multifunctional procollagen lysine hydroxylase and glycosyltransferase LH3	80
-4.64E-05	-0.000172952	0.000346061	0.000460141	0.00125657	O60684	Importin subunit alpha-7	81
-0.00259418	-0.00119006	-4.37E-05	-0.00426534	0.00576834	O60701	UDP-glucose 6-dehydrogenase	82
0.000181268	-0.000843237	0.00159351	-0.000159628	-0.00231072	O60763	General vesicular transport factor p115	83
-0.0553345	0.0529314	-0.10183	-0.128789	0.0488408	Q99880	Histone H2B type 1-L	84
-0.000258232	-0.000490119	0.000713346	-0.00150071	0.00089901	H7BZF2	H/ACA ribonucleoprotein complex subunit DKC1 (Fragment)	85
-4.58E-05	0.000181049	2.59E-05	-0.000261215	0.000333398	A0A087WT48	DnaJ homolog subfamily A member 2 (Fragment)	86
-4.06E-05	-0.000200554	0.000377256	-0.000229666	-0.00025384	O75054	Immunoglobulin superfamily member 3	87
0.0034158	-0.0162865	0.0119113	0.00880316	-0.00248513	O75083	WD repeat-containing protein 1	88
-0.00153519	0.00181838	0.00108084	-0.00123	0.00314212	O75131	Copine-3	89
-0.00100675	-0.000243451	-0.000955239	-0.00245822	0.00035457	O75165	DnaJ homolog subfamily C member 13	90

-6.88E-05	-0.000222434	-5.98E-06	-0.000755346	0.00181248	O75191-2	Isoform 2 of Xylulose kinase	91
-0.000431903	0.000213934	1.30E-05	-0.000333189	0.000612409	O75340	Programmed cell death protein 6	92
-5.97E-05	0.000339889	9.13E-05	2.39E-05	0.00204965	O75351	Vacuolar protein sorting-associated protein 4B	93
-0.0114059	0.00568253	-0.00853434	-0.0170944	0.00244482	O75367-2	Isoform 1 of Core histone macro-H2A.1	94
-0.000114381	-0.000370925	-8.06E-05	0.000266158	-0.00031561	O75369-5	Isoform 5 of Filamin-B	95
-0.000286867	0.000172631	0.00192521	-0.000807119	-3.15E-05	A0A0C4DGI3	Citrate synthase	96
-2.53E-05	-6.81E-05	0.000634179	0.000267589	-0.00156283	O75400-2	Isoform 2 of Pre-mRNA-processing factor 40 homolog A	97
0.000781585	0.010208	-0.00500463	0.00189659	-0.0122377	O75531	Barrier-to-autointegration factor	98
-1.09E-05	-0.000515487	0.000453867	-9.35E-05	-0.0003675	O75533	Splicing factor 3B subunit 1	99
-2.85E-05	0.000112785	1.62E-05	-0.000162724	0.000207691	E9PLT0	Cold shock domain-containing protein E1	100
-0.000166743	-0.00146207	0.00238067	-0.000704747	-0.0012716	O75643	U5 small nuclear ribonucleoprotein 200 kDa helicase	101
0.00903371	-0.000902006	0.000382331	0.027332	0.00429578	O75718	Cartilage-associated protein	102
-0.000311536	0.00123167	0.000176483	-0.00177704	0.00226811	O75828	Carbonyl reductase [NADPH] 3	103
-0.000116533	0.00046072	6.60E-05	-0.000664722	0.000848409	O75829-2	Isoform 2 of Leukocyte cell-derived chemotaxin 1	104
1.64E-05	-0.000347895	-5.70E-05	-0.000644556	0.00108095	B1AJY5	26S proteasome non-ATPase regulatory subunit 10	105
0.00113059	-0.00102766	0.000162807	0.00102575	-0.00427413	O75874	Isocitrate dehydrogenase [NADP] cytoplasmic	106
-0.000118945	0.000470255	6.74E-05	-0.000678478	0.000865967	O75891-2	Isoform 2 of Cytosolic 10-formyltetrahydrofolate dehydrogenase	107
-0.000946241	-0.00175595	0.000249159	-0.00115313	-0.00261022	O75897-2	Isoform 2 of Sulfotransferase 1C4	108
-9.21E-05	0.000363971	5.22E-05	-0.000525133	0.000670247	F8WF90	PRA1 family protein	109
0.00325061	0.000941947	-0.00323709	0.00547383	0.00664927	A0A3B3IST1	Tetraspanin-9 (Fragment)	110
-0.000502579	0.000235751	0.000979083	-0.00183144	0.00056038	I3L3C4	Ribosomal L1 domain-containing protein 1 (Fragment)	111
-1.34E-05	5.28E-05	7.57E-06	-7.62E-05	9.73E-05	F6S2S5	LIM and SH3 domain protein 1	112
-6.84E-05	1.28E-05	-0.000153421	7.09E-05	-0.000317439	H0YB13	Stanniocalcin-2 (Fragment)	113
-0.000278102	-0.000204958	0.000853975	2.65E-05	-0.00254415	G5E9C5	Phosphodiesterase	114

-0.000265942	0.00105141	0.000150654	-0.00151697	0.00193616	O94955	Rho-related BTB domain-containing protein 3	115
-0.000569055	0.000372038	7.23E-05	-0.00101261	0.00184822	D6RHZ5	Protein transport protein Sec31A	116
0.0243491	0.0168504	-0.0188181	0.0631388	-0.00449434	O95084-2	Isoform 2 of Serine protease 23	117
-9.70E-05	0.000383346	5.49E-05	-0.000553087	0.000705925	O95154	Aflatoxin B1 aldehyde reductase member 3	118
-0.000822911	0.000169526	-0.000929552	-0.000639613	-0.000413984	O95197-3	Isoform 3 of Reticulon-3	119
0.000282888	6.44E-05	-7.13E-05	0.00088459	0.0001768	D6RDI2	Luc7-like protein 3 (Fragment)	120
0.00214814	0.000742352	-0.000754657	0.00454406	-0.00293545	Q9UEL6	Myelin protein zero-like protein 1 (Fragment)	121
8.80E-06	-4.30E-05	-0.00109295	-7.53E-05	0.00368273	O95372	Acyl-protein thioesterase 2	122
0.000833888	-0.00138763	0.00173584	0.00363985	0.000370895	O95373	Importin-7	123
-6.94E-05	-0.000275231	0.00109404	0.00358103	-0.00438607	O95445-2	Isoform 2 of Apolipoprotein M	124
2.78E-05	0.000183225	6.33E-05	0.000488384	-0.000958417	O95490-3	Isoform 3 of Adhesion G protein-coupled receptor L2	125
-0.000146393	0.000578773	8.29E-05	-0.000835047	0.0010658	O95497	Pantetheinase	126
-0.000210723	-8.57E-05	-0.00100297	-0.00036974	0.00135924	O95782-2	Isoform B of AP-2 complex subunit alpha-1	127
0.00235889	-0.00761205	0.00368197	0.00854684	-0.00787561	O95834	Echinoderm microtubule-associated protein-like 2	128
0.00308888	0.00313525	0.00141216	0.00797593	-0.00851252	E9PRU1	EGF-containing fibulin-like extracellular matrix protein 2	129
-0.000164253	-0.000686121	0.00108624	-0.000512098	-0.00118785	O96019-2	Isoform 2 of Actin-like protein 6A	130
-0.00828814	0.00472056	-0.00713751	-0.00812884	0.0088862	P00338	L-lactate dehydrogenase A chain	131
-4.78E-05	-0.000845853	0.000629366	-0.000305933	-0.000997055	P00352	Retinal dehydrogenase 1	132
-4.08E-05	0.000161258	2.31E-05	-0.000232661	0.000296953	P00387-2	Isoform 2 of NADH-cytochrome b5 reductase 3	133
-0.00221155	0.00874349	0.00125283	-0.012615	0.016101	H7BYH4	Superoxide dismutase [Cu-Zn]	134
-0.000603664	-0.000920466	0.000294183	-0.000427994	-0.00281627	P00450	Ceruloplasmin	135
0.00185372	-0.00765285	0.0127266	0.00539199	-0.0117221	P00488	Coagulation factor XIII A chain	136
0.000596734	-0.000110504	0.000966902	-2.38E-05	-0.0051662	P00491	Purine nucleoside phosphorylase	137
0.000377945	0.000504684	0.000381608	-0.000866247	-9.42E-05	P00533-4	Isoform 4 of Epidermal growth factor receptor	138
-0.00252753	-0.0152292	0.00695824	0.00140113	-0.0121924	P00558	Phosphoglycerate kinase 1	139
-0.0225399	-0.0391567	-0.000109556	-0.0559157	0.0963179	E9PIT3	Prothrombin	140

-0.000736667	0.000642376	0.000265493	-0.00151245	-0.000493743	F5H2D0	Complement C1r subcomponent	141
-0.00343483	0.00199484	-0.00203288	-0.00522458	-0.00332357	A0A0C4DGL8	Haptoglobin	142
-0.000614372	-0.000403879	0.00155238	-0.00324652	0.000846773	P00742	Coagulation factor X	143
-0.00115956	0.00221454	-0.0028813	-0.000478196	-0.00558959	P00747	Plasminogen	144
0.000277773	0.000240295	-0.000103844	0.000358117	-0.00175914	E7ET40	Urokinase-type plasminogen activator	145
-0.00166617	-0.00185188	0.000770565	-0.00363751	-0.000279254	P00966	Argininosuccinate synthase	146
-0.00542784	0.00960823	-0.00911618	0.0208026	-0.00646952	P01008	Antithrombin-III	147
-0.00044675	-0.000212812	-0.00137691	0.000223822	-0.000474233	P01011	Alpha-1-antichymotrypsin	148
-0.0838907	0.129096	0.00601004	-0.349389	-0.0727229	P01023	Alpha-2-macroglobulin	149
-0.0126859	0.0203545	0.00385641	-0.0176855	-0.00159993	P01024	Complement C3	150
-0.000304243	0.00133376	0.000198628	-0.00289966	0.00058534	P01031	Complement C5	151
-0.00068537	-0.00109074	-0.00207	0.0045742	0.0101012	P01042-3	Isoform 3 of Kininogen-1	152
0.00368632	-0.00330091	0.00238328	0.00166799	0.00448385	A6XGL2	Insulin	153
-0.000121648	0.000480941	6.89E-05	-0.000693897	0.000885646	P01344	Insulin-like growth factor II	154
-0.000177256	-0.000548799	0.00393682	0.00135478	-0.00916921	D6RHJ6	Immunoglobulin J chain (Fragment)	155
-0.00013965	0.000552112	7.91E-05	-0.000796581	0.00101671	P01834	Immunoglobulin kappa constant	156
-0.00140425	-0.00122541	-0.00359303	-0.00179208	0.0021214	A0A0A0MS07	Immunoglobulin heavy constant gamma 1 (Fragment)	157
3.89E-05	-0.00266075	-0.00244154	-0.000108821	0.00833474	P02100	Hemoglobin subunit epsilon	158
0.06782	0.00090747	-0.0349397	0.0356901	-0.0112305	P02452	Collagen alpha-1(I) chain	159
0.00513904	0.00225584	-0.00163616	0.0120455	0.00199863	P02461	Collagen alpha-1(III) chain	160
-0.000627301	0.000437555	0.000997172	-0.00199293	-0.000829866	A0A3B3ISV3	Collagen alpha-1(IV) chain (Fragment)	161
-7.16E-05	0.000283238	4.06E-05	-0.000408653	0.000521579	E9PNH7	Alpha-crystallin B chain (Fragment)	162
-0.00098779	-0.0014511	-0.000928243	0.00725422	0.00890239	P02533	Keratin, type I cytoskeletal 14	163
-0.000190274	-0.00135409	0.000157828	0.00549425	0.00695408	P48668	Keratin, type II cytoskeletal 6C	164
-8.19E-05	0.000323931	4.64E-05	-0.000467364	0.000596513	Q3BDU5	Prelamin-A/C	165
-0.022273	-0.0125008	-0.061492	-0.00417836	-0.0259055	P02647	Apolipoprotein A-I	166
0.0196404	-0.00734163	-0.00917767	-0.0396582	-0.0384997	P02649	Apolipoprotein E	167
0.000642726	5.07E-05	-0.000517307	-0.00275963	0.000540078	P02656	Apolipoprotein C-III	168
-0.000164005	0.000648403	9.29E-05	-0.000935508	0.00119402	A0A087WUA0	Fibrinogen alpha chain	169
-0.00475526	-0.00953654	-0.0108463	-0.000522729	-0.00497091	P02675	Fibrinogen beta chain	170
-0.00340243	0.00110295	0.000128236	-0.00421457	-0.00707939	P02679-2	Isoform Gamma-A of Fibrinogen gamma chain	171

-0.00122998	-0.000539418	-0.00294418	-0.00045555	-0.000967237	E9PMR5	Myelin basic protein	172
-0.00335879	0.00259986	-0.0041119	-0.00349283	-0.00712565	P02748	Complement component C9	173
-0.00175293	-0.00350285	0.00304165	-0.00442284	-0.00640228	J3KS17	Beta-2-glycoprotein 1 (Fragment)	174
0.300878	0.341271	0.14253	-0.107594	-0.471289	P02751	Fibronectin	175
-0.00119035	0.000861629	-0.000244645	0.00355266	0.000279448	Q5VY30	Retinol-binding protein	176
0.00593819	0.0462699	-0.0240456	0.161698	-0.0871971	P02765	Alpha-2-HS-glycoprotein	177
-0.254776	-0.0249959	-0.67869	0.0438459	-0.290282	P02768	Serum albumin	178
-0.0022817	-0.00249337	-0.0114349	-0.0332042	0.0732464	P02771	Alpha-fetoprotein	179
0.00671694	-0.00621748	-0.0158245	0.0199714	0.0201751	P02774	Vitamin D-binding protein	180
-0.0107369	-0.00443222	0.00936518	-0.030694	-0.000499798	P02786	Transferrin receptor protein 1	181
-0.00023791	-0.000229776	-0.000557082	1.47E-05	-0.000416426	C9JVG0	Serotransferrin (Fragment)	182
-0.00834943	0.00968936	-0.0377787	0.0204875	-0.0160573	P02788-2	Isoform DeltaLf of Lactotransferrin	183
-0.00156461	-0.000545662	-0.00389755	0.00074351	-0.00193933	P02790	Hemopexin	184
-0.00160058	0.000612259	-0.00300347	0.00129708	-0.00841242	E9PRK8	Ferritin	185
-4.62E-05	0.000182686	2.62E-05	-0.000263578	0.000336414	Q8N339	Metallothionein-1M	186
-2.47E-05	-0.000122179	0.000229827	-0.000139914	-0.000154641	P03971	Muellerian-inhibiting factor	187
-0.00637699	-0.00819816	-0.00262536	0.00662952	-0.0228829	P04004	Vitronectin	188
0.000557627	-0.000764029	0.000377491	5.87E-05	0.00226031	A0A0G2JNZ5	Glucosylceramidase	189
0.000362764	-0.000352278	0.00054905	0.000625201	7.16E-05	P04066	Tissue alpha-L-fucosidase	190
-0.000823691	0.00103089	0.00265053	-0.00468696	0.00261024	P04075	Fructose-bisphosphate aldolase A	191
-0.00172854	-0.003649	0.0088723	-0.00744444	-0.00412362	P04083	Annexin A1	192
0.00582472	-0.00201899	0.00160142	-0.00713721	0.01328	P04114	Apolipoprotein B-100	193
-0.000276831	0.000253017	0.000423554	-0.00103442	0.000424912	A2A2V1	Major prion protein (Fragment)	194
-0.000966085	-0.000933054	-0.00226215	5.98E-05	-0.00169099	J3QKT0	Phosphatidylcholine-sterol acyltransferase (Fragment)	195
-0.000274719	4.21E-06	0.000293137	0.000240013	0.000470269	P04181	Ornithine aminotransferase, mitochondrial	196
-2.98E-06	1.18E-05	1.69E-06	-1.70E-05	2.17E-05	P04196	Histidine-rich glycoprotein	197
0.000868457	0.00523666	0.00545697	-0.00471298	-0.016895	E9PIM6	Thy-1 membrane glycoprotein (Fragment)	198
-0.0136895	-0.0344238	0.0176922	0.175442	0.158601	P04264	Keratin, type II cytoskeletal 1	199
-5.95E-05	-0.000294045	0.000553121	-0.000336728	-0.000372172	P04350	Tubulin beta-4A chain	200
-0.0602094	-0.0791669	0.132241	-0.183607	-0.00487759	P04406	Glyceraldehyde-3-phosphate dehydrogenase	201
-0.000463472	0.000532683	3.39E-05	-0.000605099	-0.000817004	P04424	Argininosuccinate lyase	202

-7.54E-05	8.72E-05	0.000127176	-0.000200318	0.000548615	P19961-2	Isoform 2 of Alpha-amylase 2B	203
0.000371852	0.000888472	0.00265971	0.000322669	-0.000146362	P04792	Heat shock protein beta-1	204
-0.000871537	-0.00321849	0.00499434	-0.00418297	-0.00628462	P04899	Guanine nucleotide-binding protein G(i) subunit alpha-2	205
0.00654916	-0.00184664	-0.00592567	-0.0129131	-0.00455203	Q99878	Histone H2A type 1-J	206
-0.0018105	0.000140422	0.00266895	-0.00607836	-0.0040864	P05023-3	Isoform 3 of Sodium/potassium-transporting ATPase subunit alpha-1	207
-1.53E-05	3.43E-05	-0.000275694	-0.000391007	0.000801744	P05026-2	Isoform 2 of Sodium/potassium-transporting ATPase subunit beta-1	208
-0.000130199	0.000107158	-0.00115865	-0.000827187	0.00237182	P05067-2	Isoform APP305 of Amyloid-beta precursor protein	209
-0.00612788	0.00673072	0.0130957	-0.0373834	-0.0548309	P05106	Integrin beta-3	210
-0.00862148	0.00714397	0.00253966	-0.0173582	0.0240238	P05121	Plasminogen activator inhibitor 1	211
0.00106589	-0.000239321	0.00132521	0.00134125	0.000763731	P05155-2	Isoform 2 of Plasma protease C1 inhibitor	212
-0.000174224	0.000688804	9.87E-05	-0.000993799	0.00126842	A0A087X0I2	Complement factor I	213
0.000102289	1.15E-05	0.000372866	0.00069941	0.000215009	H0YJS4	Eukaryotic translation initiation factor 2 subunit 1 (Fragment)	214
-0.00119955	0.00255369	-0.000163738	-0.00542817	-0.0128112	P05387	60S acidic ribosomal protein P2	215
-0.00186289	0.000586658	0.000652628	-0.00274454	-0.00137295	P05388	60S acidic ribosomal protein P0	216
9.52E-05	0.000627929	0.00021693	0.00167374	-0.00328458	S4R371	Fatty acid-binding protein, heart (Fragment)	217
0.000263886	0.000461985	-0.000965186	0.00248712	-0.00101259	P05452	Tetranectin	218
-7.83E-05	0.000309539	4.44E-05	-0.000446599	0.00057001	P05455	Lupus La protein	219
-1.56E-06	0.00281526	0.011175	-0.0116519	-0.0191466	P05534	HLA class I histocompatibility antigen, A-24 alpha chain	220
0.00278656	0.00707189	-0.00684369	0.0119721	-0.00797948	P05546	Heparin cofactor 2	221
0.00390366	0.0178498	-0.000531916	-0.049573	0.0380626	P05556	Integrin beta-1	222
-2.72E-05	-0.000134185	0.000252411	-0.000153663	-0.000169837	P05976-2	Isoform MLC3 of Myosin light chain 1/3, skeletal muscle isoform	223
0.000377493	-4.28E-05	-0.000334219	-0.000506518	-0.00017224	A0A087WYX9	Collagen alpha-2(V) chain	224
-0.000455184	-0.00101879	-0.000667928	-0.000463759	0.00331303	P06132	Uroporphyrinogen decarboxylase	225
0.00205051	-3.49E-05	-0.00197542	0.00330893	-0.0172953	P06396-2	Isoform 2 of Gelsolin	226
-1.67E-05	-1.61E-05	-3.90E-05	1.03E-06	-2.92E-05	B8ZZQ6	Prothymosin alpha	227

-0.000802844	-0.00139623	0.00269799	-0.00251144	6.12E-05	P06576	ATP synthase subunit beta, mitochondrial	228
-0.000122158	-0.000117981	-0.00028604	7.56E-06	-0.000213819	P06727	Apolipoprotein A-IV	229
-0.000594602	0.000150338	-0.00171359	-0.00106102	0.00244377	P06730	Eukaryotic translation initiation factor 4E	230
-0.00400835	-0.00564455	0.007072	0.00392471	-0.0147186	P06733	Alpha-enolase	231
-0.00232051	-0.00258674	-0.00175078	0.0010997	-0.00891644	P06737-2	Isoform 2 of Glycogen phosphorylase, liver form	232
-4.47E-05	0.000176788	2.53E-05	-0.000255068	0.000325552	A0A0J9YXP8	Glucose-6-phosphate isomerase (Fragment)	233
-0.000414926	0.000836366	0.000627877	-8.50E-05	0.00368658	P06748-2	Isoform 2 of Nucleophosmin	234
-9.88E-05	0.000390577	5.60E-05	-0.00056352	0.000719241	H0YL80	Tropomyosin alpha-1 chain (Fragment)	235
-0.000631245	-0.000316335	0.00794041	-0.00918921	-0.00103389	P06756-3	Isoform 3 of Integrin alpha-V	236
2.95E-05	-0.000437897	0.000696743	2.87E-05	0.000319619	P06858	Lipoprotein lipase	237
0.00152898	-0.0207108	0.00292526	0.0352122	-0.0559837	Q16778	Histone H2B type 2-E	238
-5.69E-05	0.000120637	-0.000221709	0.0016616	-0.000837435	P07093-2	Isoform 2 of Glia-derived nexin	239
-0.00108608	0.00155827	-0.00106228	-0.00475373	0.00320923	P07099	Epoxide hydrolase 1	240
-0.0119283	-0.00404576	-0.0128107	0.0177435	0.0118877	P07195	L-lactate dehydrogenase B chain	241
-0.000213633	-0.000568635	0.000566583	8.26E-05	-0.00131314	A0A087WUQ6	Glutathione peroxidase	242
-0.00110447	0.00341517	0.000282219	0.00560346	-0.00609476	P07305	Histone H1.0	243
-0.00023332	-0.000108784	0.000895348	-0.000748236	-0.000220157	H7C469	Uncharacterized protein (Fragment)	244
-0.00615636	-0.00707664	0.0207951	-0.0207181	-0.0118327	P07355	Annexin A2	245
-0.0106051	-0.0105557	-0.00159288	-0.031146	0.00970537	Q5JP53	Tubulin beta chain	246
0.00243327	-0.00135311	0.00335596	0.0141148	-0.00528468	P07737	Profilin-1	247
0.00191115	-0.00110158	0.00231847	0.00345191	-0.00260836	P07738	Bisphosphoglycerate mutase	248
-0.00048016	0.00189834	0.000272007	-0.0027389	0.00349576	P07741-2	Isoform 2 of Adenine phosphoribosyltransferase	249
-0.000746961	0.00113954	-0.00157192	-0.00239378	0.00650578	P07814	Bifunctional glutamate/proline--tRNA ligase	250
-3.23E-05	0.00012764	1.83E-05	-0.000184158	0.000235048	P07858	Cathepsin B	251
0.00131523	-0.00797744	0.0131025	0.00954143	-0.0121592	P07900	Heat shock protein HSP 90-alpha	252
-0.000403059	0.00113154	0.000198471	-0.0018645	0.0030269	G3V576	Heterogeneous nuclear ribonucleoproteins C1/C2	253
0.0230393	0.0223678	-0.0113366	-0.0233399	0.00768762	P07942	Laminin subunit beta-1	254

0.686192	-0.000592957	-0.443448	-0.131467	0.214344	P07996	Thrombospondin-1	255
0.0202676	0.00300882	-0.00992873	0.0214127	-0.00847455	A0A087WTA8	Collagen alpha-2(I) chain	256
-0.00381107	-0.00439508	0.0137249	-0.0146789	-0.0031435	P08133	Annexin A6	257
-9.31E-05	0.000368024	5.27E-05	-0.00053098	0.00067771	H3BLV0	Complement decay-accelerating factor (Fragment)	258
-0.000640065	-0.00542604	0.0225129	-0.0270835	0.00977409	P08195-2	Isoform 2 of 4F2 cell-surface antigen heavy chain	259
0.0188967	-0.0162288	-0.00124849	0.032305	0.0127906	P08238	Heat shock protein HSP 90-beta	260
0.00158191	-0.000406193	-0.000650655	-0.000480799	-0.000871404	P08253-2	Isoform 2 of 72 kDa type IV collagenase	261
2.99E-05	0.000196862	6.80E-05	0.000524733	-0.00102975	P09210	Glutathione S-transferase A2	262
0.000277617	-0.000149038	-0.00017595	0.000197069	0.00114565	P08476	Inhibin beta A chain	263
3.17E-05	-0.000881899	0.00941219	-0.00408649	-0.014829	A0A3B3IU79	Integrin alpha-IIb (Fragment)	264
0.00134666	-0.00204699	-0.00169522	0.00593239	0.0054467	P08567	Pleckstrin	265
-0.000145684	0.00057597	8.25E-05	-0.000831002	0.00106064	P08572	Collagen alpha-2(IV) chain	266
-0.000346935	-0.000538026	0.00114249	0.00083201	-0.00168457	P08621	U1 small nuclear ribonucleoprotein 70 kDa	267
0.00345765	0.000577805	0.00129722	0.00830252	0.00653493	P08648	Integrin alpha-5	268
-0.000895441	-0.000863006	0.00100951	-0.00423679	-0.00578908	B0YJC4	Vimentin variant 3	269
-0.00176579	-0.00234946	0.000559008	-0.00753711	0.000351086	C9JMH6	Alpha-2-antiplasmin (Fragment)	270
-0.00148744	0.00409974	-0.00139774	-0.0103086	0.00822005	P08708	40S ribosomal protein S17	271
-0.00259455	-0.00384848	0.0102374	-0.00804615	-0.00838837	P08758	Annexin A5	272
-0.00432345	-0.00643486	0.0155419	-0.00980412	-0.00268898	C9J9K3	40S ribosomal protein SA (Fragment)	273
0.000561534	-0.00215027	8.56E-05	0.00450758	0.00192697	P09211	Glutathione S-transferase P	274
0.000244152	0.00315953	-0.00093244	-0.000683784	-0.00149052	P09382	Galectin-1	275
0.000514779	-0.000974132	0.00195877	0.000598696	-0.00160201	P09417	Dihydropteridine reductase	276
-0.000193084	6.57E-05	-0.000259912	-0.000275758	0.000562723	Q5T7C4	High mobility group protein B1	277
-0.00015222	0.000601812	8.62E-05	-0.000868287	0.00110823	Q5VZC3	Fructose-1,6-bisphosphatase 1 (Fragment)	278
0.000228578	0.000166597	-0.000505341	0.00252389	-0.000853733	P09486	SPARC	279
-0.00306452	0.00704831	-0.00282488	0.00631107	-0.0106683	P09496-2	Isoform Non-brain of Clathrin light chain A	280
3.80E-05	0.000827059	-4.66E-05	0.000878759	-0.00244164	P09497-2	Isoform Non-brain of Clathrin light chain B	281

**-6.11E-05**	**0.00024163**	**3.46E-05**	**-0.00034862**	**0.000444957**	**P09543-2**	**Isoform CNPI of 2,3-cyclic-nucleotide 3-phosphodiesterase**	**282**
**0.00443131**	**0.000302016**	**-0.000124364**	**0.00822505**	**-0.00469056**	**P09619**	**Platelet-derived growth factor receptor beta**	**283**
**-4.26E-05**	**0.000105803**	**-0.000263057**	**-0.000212349**	**-0.000285496**	**F8VZ49**	**Heterogeneous nuclear ribonucleoprotein A1 (Fragment)**	**284**
**-0.000115891**	**0.00045818**	**6.57E-05**	**-0.000661057**	**0.000843732**	**H0YMA0**	**U2 small nuclear ribonucleoprotein A (Fragment)**	**285**
**-0.00491384**	**0.00472547**	**-0.00298765**	**-0.0114868**	**-0.00637108**	**F5GXS0**	**Complement C4-B**	**286**
**-0.00899118**	**0.00302048**	**-0.00126731**	**-0.0110898**	**-0.014605**	**Q71UI9**	**Histone H2A.V**	**287**
**-0.000665806**	**-0.0113752**	**0.0114949**	**0.0128883**	**-0.0220308**	**P0DMV9**	**Heat shock 70 kDa protein 1B**	**288**
**-0.000190229**	**-0.000412196**	**0.000552231**	**-0.00103459**	**-0.00228496**	**P0DP25**	**Calmodulin-3**	**289**
**-0.000143374**	**0.000566837**	**8.12E-05**	**-0.000817826**	**0.00104382**	**P10124**	**Serglycin**	**290**
**-0.00068832**	**0.000302204**	**-0.00104011**	**-0.00109082**	**0.000570358**	**P10599**	**Thioredoxin**	**291**
**-0.000130949**	**0.000173112**	**0.00178929**	**0.00114359**	**0.00167593**	**K7EPB2**	**cAMP-dependent protein kinase type I-alpha regulatory subunit (Fragment)**	**292**
**0.000402571**	**0.000208463**	**-8.10E-06**	**5.50E-05**	**0.00121274**	**P10646**	**Tissue factor pathway inhibitor**	**293**
**-0.000112285**	**-0.000440199**	**0.000942767**	**0.000736336**	**-0.00123432**	**P10768**	**S-formylglutathione hydrolase**	**294**
**-0.000514243**	**1.84E-05**	**-0.000637688**	**-0.000928536**	**0.000298489**	**P10809**	**60 kDa heat shock protein, mitochondrial**	**295**
**0.000205441**	**-0.00466776**	**0.00189476**	**-0.00477925**	**-0.00669491**	**P10909-4**	**Isoform 4 of Clusterin**	**296**
**0.000358038**	**-0.000100954**	**-0.000323952**	**-0.000705953**	**-0.000248857**	**P10915**	**Hyaluronan and proteoglycan link protein 1**	**297**
**5.22E-05**	**-0.000131052**	**1.73E-05**	**0.000613459**	**-0.000895485**	**P11021**	**Endoplasmic reticulum chaperone BiP**	**298**
**0.0224088**	**0.0243952**	**-0.0067414**	**-0.0166549**	**-0.0200147**	**P11047**	**Laminin subunit gamma-1**	**299**
**0.00137102**	**-0.0314051**	**0.030796**	**0.00032895**	**-0.0218832**	**P11142**	**Heat shock cognate 71 kDa protein**	**300**
**-0.000704451**	**-0.000798168**	**0.000473847**	**-0.00161799**	**-0.00399931**	**P11166**	**Solute carrier family 2, facilitated glucose transporter member 1**	**301**
**-0.000176059**	**-0.000616478**	**0.000789964**	**0.00187565**	**0.000794602**	**A0A2R8Y6D0**	**Protein 4.1**	**302**
**-0.00108544**	**-0.000596155**	**-0.00105882**	**-0.000806374**	**-0.00222769**	**P11216**	**Glycogen phosphorylase, brain form**	**303**
**-5.68E-05**	**0.000873886**	**-0.00114821**	**0.000696841**	**0.00164989**	**P11234**	**Ras-related protein Ral-B**	**304**
**-0.00567385**	**0.000366931**	**-0.00103764**	**-0.00396282**	**-0.0102628**	**P11387**	**DNA topoisomerase 1**	**305**

-7.86E-06	3.11E-05	4.45E-06	-4.48E-05	5.72E-05	P11388	DNA topoisomerase 2-alpha	306
-0.0114105	-0.0128916	0.0171064	-0.0259292	0.00198393	P11413	Glucose-6-phosphate 1-dehydrogenase	307
-0.00038993	0.000257334	-6.64E-05	0.000903718	0.000665898	P11586	C-1-tetrahydrofolate synthase, cytoplasmic	308
-0.000220259	-0.00182379	0.00119362	-0.00304846	0.00329821	P11717	Cation-independent mannose-6-phosphate receptor	309
-0.000119996	-0.00138724	0.00235153	0.00259238	-0.00400661	P11766	Alcohol dehydrogenase class-3	310
-0.000240877	0.000789571	0.000260278	-0.00122431	0.00107453	A0A087WTT1	Polyadenylate-binding protein	311
-0.000228915	0.000736132	0.00142322	-0.000285454	-0.00228819	P12004	Proliferating cell nuclear antigen	312
-0.0471867	0.182768	-0.0423593	0.0847907	-0.000369471	A0A087X0S5	Collagen alpha-1(VI) chain	313
-0.0417737	0.172145	-0.0254339	0.0708496	-0.0111187	P12110	Collagen alpha-2(VI) chain	314
-0.219749	0.792203	-0.0524775	0.1258	0.222885	P12111	Collagen alpha-3(VI) chain	315
0.00823806	-0.0394124	0.0211965	0.00328062	-0.0138127	P12259	Coagulation factor V	316
0.000246503	2.63E-05	-1.45E-05	0.000797807	2.18E-05	H0Y4R1	Inosine-5-monophosphate dehydrogenase 2 (Fragment)	317
-8.75E-05	0.000345887	4.96E-05	-0.000499042	0.000636945	G3V461	Creatine kinase B-type (Fragment)	318
-6.64E-05	4.71E-05	-2.71E-05	-0.00015097	-0.0018122	H9KV75	Alpha-actinin-1	319
-0.00493609	0.00078856	-0.00582759	-0.0139567	0.00251208	P12956	X-ray repair cross-complementing protein 6	320
-0.00791485	0.00506516	-0.00542753	-0.021959	0.00648403	P13010	X-ray repair cross-complementing protein 5	321
-0.000972043	0.00109777	0.000566858	-0.00403724	0.0006529	P13224	Platelet glycoprotein Ib beta chain	322
-9.52E-05	0.000716988	0.000242463	0.000653882	0.00599394	P13473-2	Isoform LAMP-2B of Lysosome-associated membrane glycoprotein 2	323
0.000497041	-0.000713107	-0.000197711	0.00291903	-0.00366092	P13489	Ribonuclease inhibitor	324
8.78E-05	0.000397502	-0.000283944	0.000848745	2.86E-05	P13497-5	Isoform BMP1-6 of Bone morphogenetic protein 1	325
0.0803761	-0.018499	-0.0199343	-0.086272	-0.0289192	P13611	Versican core protein	326
0.0121625	-0.00117456	-0.00345043	0.0176881	-0.00103507	P13612	Integrin alpha-4	327
-0.00191436	-0.0135485	0.0157354	-0.00322784	0.0130626	P13639	Elongation factor 2	328
-0.00896579	-0.00735134	-0.00840011	0.0324077	-0.00290825	P13645	Keratin, type I cytoskeletal 10	329
-0.000149459	-0.000660002	0.000299359	0.00292927	0.00199433	P13647	Keratin, type II cytoskeletal 5	330
-0.000253343	0.0010016	0.000143517	-0.0014451	0.00184444	P13671	Complement component C6	331

**-0.000403561**	**-7.01E-05**	**0.000442569**	**-0.00110389**	**-0.000444398**	**P13674-3**	**Isoform 3 of Prolyl 4-hydroxylase subunit alpha-1**	**332**
**-0.000193087**	**-0.000954042**	**0.00179462**	**-0.00109253**	**-0.00120753**	**P13693**	**Translationally-controlled tumor protein**	**333**
**0.0010306**	**0.000857655**	**-0.000459875**	**0.000958115**	**0.00402497**	**P13726-2**	**Isoform 2 of Tissue factor**	**334**
**4.44E-05**	**0.000544703**	**0.000659652**	**-0.00068487**	**0.000955731**	**P13807-2**	**Isoform 2 of Glycogen [starch] synthase, muscle**	**335**
**-0.000130298**	**0.000515139**	**7.38E-05**	**-0.000743236**	**0.00094862**	**H7C330**	**cAMP-dependent protein kinase type II-alpha regulatory subunit (Fragment)**	**336**
**-0.000202624**	**-0.000195696**	**-0.000474456**	**1.25E-05**	**-0.000354662**	**P13929-3**	**Isoform 3 of Beta-enolase**	**337**
**0.00719742**	**0.00634305**	**-0.00831476**	**-0.00384629**	**0.0156008**	**E9PNW4**	**Uncharacterized protein**	**338**
**0.000715731**	**-0.000607058**	**0.000261299**	**0.0021161**	**0.000706215**	**P14550**	**Aldo-keto reductase family 1 member A1**	**339**
**-0.0240768**	**-0.0495124**	**0.0457556**	**-0.0247139**	**-0.0183932**	**P14618**	**Pyruvate kinase PKM**	**340**
**0.000583692**	**0.000221202**	**-0.000332472**	**0.00218799**	**0.00119249**	**P14625**	**Endoplasmin**	**341**
**0.000284717**	**-0.000164772**	**-0.000807323**	**0.00159378**	**-9.94E-05**	**J3QLE5**	**Small nuclear ribonucleoprotein-associated protein N (Fragment)**	**342**
**-0.000105928**	**0.000418792**	**6.00E-05**	**-0.000604229**	**0.000771199**	**M0QXS5**	**Heterogeneous nuclear ribonucleoprotein L (Fragment)**	**343**
**-0.00168336**	**0.000189782**	**0.00190773**	**-0.00299731**	**-0.000762841**	**P14868-2**	**Isoform 2 of Aspartate--tRNA ligase, cytoplasmic**	**344**
**-0.00029245**	**-0.00339035**	**0.0055432**	**0.00193245**	**-0.00677475**	**P15121**	**Aldose reductase**	**345**
**0.000870115**	**-9.42E-05**	**-0.000345058**	**0.00197377**	**0.00206818**	**P15144**	**Aminopeptidase N**	**346**
**0.00029274**	**0.0013221**	**0.00101983**	**-0.000414213**	**-0.00151972**	**P15151-3**	**Isoform Gamma of Poliovirus receptor**	**347**
**0.000175772**	**-0.00170756**	**-0.00299134**	**0.00198677**	**0.00506692**	**P15153**	**Ras-related C3 botulinum toxin substrate 2**	**348**
**-0.0001014**	**0.000400889**	**5.74E-05**	**-0.000578398**	**0.00073823**	**B1AP58**	**Carboxypeptidase N catalytic chain (Fragment)**	**349**
**-0.000495805**	**-0.000651498**	**2.38E-05**	**0.000229823**	**-0.00176752**	**H3BR35**	**Eukaryotic peptide chain release factor GTP-binding subunit ERF3A (Fragment)**	**350**
**-0.000117527**	**0.000464648**	**6.66E-05**	**-0.000670389**	**0.000855641**	**C9JNS9**	**V-type proton ATPase subunit B, kidney isoform (Fragment)**	**351**

**0.00122781**	**-9.65E-06**	**-0.0011616**	**-0.000369444**	**0.00303465**	**P15529-15**	**Isoform K of Membrane cofactor protein**	**352**
**-0.014779**	**-0.00221858**	**0.00582219**	**-0.0327039**	**-0.00394453**	**P15559-2**	**Isoform 2 of NAD(P)H dehydrogenase [quinone] 1**	**353**
**-0.0123942**	**0.00404702**	**-0.0151826**	**-0.020961**	**0.0142838**	**P15880**	**40S ribosomal protein S2**	**354**
**-0.000138554**	**0.000547782**	**7.85E-05**	**-0.000790333**	**0.00100873**	**Q5TEJ0**	**Replication protein A 32 kDa subunit (Fragment)**	**355**
**-0.00417101**	**-0.00101049**	**0.00821788**	**-0.0143315**	**-0.0102324**	**H0YD13**	**CD44 antigen**	**356**
**-0.00164097**	**-0.00150892**	**0.000661236**	**-0.00205421**	**-0.00088606**	**P16152**	**Carbonyl reductase [NADPH] 1**	**357**
**0.00133231**	**0.00291758**	**-0.000330978**	**0.000858016**	**0.00690243**	**P16402**	**Histone H1.3**	**358**
**-0.0670719**	**0.0784288**	**-0.0399645**	**-0.155486**	**0.148299**	**P16403**	**Histone H1.2**	**359**
**-4.68E-05**	**-8.69E-05**	**0.000183869**	**-0.000191385**	**-9.24E-05**	**H3BQV5**	**1-phosphatidylinositol 4,5-bisphosphate phosphodiesterase gamma-2 (Fragment)**	**360**
**-0.000916475**	**0.00362333**	**0.000519177**	**-0.0052277**	**0.00667231**	**P17066**	**Heat shock 70 kDa protein 6**	**361**
**0.000501972**	**-0.000141539**	**-0.000454183**	**-0.000989752**	**-0.000348899**	**P17096**	**High mobility group protein HMG-I/HMG-Y**	**362**
**-0.000301057**	**-0.000697698**	**0.00159178**	**-0.00124958**	**-0.00149361**	**E7ESP4**	**Integrin alpha-2**	**363**
**-3.47E-05**	**0.000137096**	**1.96E-05**	**-0.000197801**	**0.00025246**	**Q15136**	**Protein kinase A-alpha (Fragment)**	**364**
**0.000259555**	**0.000843484**	**-7.86E-05**	**-0.000288902**	**-0.00144533**	**P17655-2**	**Isoform 2 of Calpain-2 catalytic subunit**	**365**
**1.99E-05**	**-0.000548118**	**0.00304651**	**0.0031056**	**-0.00362791**	**P17693-5**	**Isoform 5 of HLA class I histocompatibility antigen, alpha chain G**	**366**
**-0.00026326**	**-0.000367205**	**-0.000648144**	**-0.000564874**	**0.000171071**	**A0A3B3IRI2**	**CTP synthase**	**367**
**0.00172249**	**-0.000338521**	**-0.000731176**	**-0.00186376**	**0.00371168**	**P17813-2**	**Isoform Short of Endoglin**	**368**
**-0.000125071**	**0.000183719**	**0.00119455**	**0.000334976**	**0.000496664**	**J3KTA4**	**Probable ATP-dependent RNA helicase DDX5**	**369**
**-0.000942451**	**-0.00015172**	**0.000907522**	**-0.00161767**	**0.000387214**	**P17858**	**ATP-dependent 6-phosphofructokinase, liver type**	**370**
**-0.000360323**	**-0.000562866**	**-0.000182965**	**-0.00418055**	**0.00253459**	**R4GNH3**	**26S proteasome regulatory subunit 6A**	**371**
**0.00374963**	**-0.00724165**	**-0.0059165**	**-0.00170414**	**0.0144506**	**P17987**	**T-complex protein 1 subunit alpha**	**372**
**-0.000981504**	**0.000649087**	**-0.00153456**	**-0.00236333**	**-0.00105648**	**F8WBS5**	**60S ribosomal protein L35a**	**373**
**-0.00244348**	**0.000233378**	**0.00420745**	**-0.00858876**	**-0.00454724**	**P18084**	**Integrin beta-5**	**374**

-0.000955404	0.000451749	-0.00373658	0.0198438	0.00476497	P18085	ADP-ribosylation factor 4	375
-0.00131378	0.00587888	-0.00517788	-0.00441163	-0.00273334	P18124	60S ribosomal protein L7	376
-0.000420521	6.17E-05	0.000221289	-0.00102923	-0.00137742	P18206-2	Isoform 1 of Vinculin	377
-0.00163917	0.00648056	0.00092858	-0.00935008	0.0119338	P18564-2	Isoform 2 of Integrin beta-6	378
-0.00162435	0.000243539	-0.000613253	-0.00393513	-0.00150923	A0A087WXM6	60S ribosomal protein L17 (Fragment)	379
0.00167485	-0.00354374	0.00429889	0.00275446	-0.00330081	P18669	Phosphoglycerate mutase 1	380
-5.39E-05	0.000106034	0.00082192	0.000607191	0.000605567	C9JRH2	Regulator of chromosome condensation (Fragment)	381
-4.98E-05	0.000197023	2.82E-05	-0.000284263	0.000362815	E9PHH3	Syndecan-1	382
1.22E-06	-0.000210829	-0.000128942	0.00105579	0.00197793	P19013	Keratin, type II cytoskeletal 4	383
-4.38E-05	0.000172985	2.48E-05	-0.000249581	0.000318549	H0YDI1	Lymphocyte function-associated antigen 3 (Fragment)	384
-0.000147886	-0.00044635	-0.000313818	0.000381265	0.00130638	P19623	Spermidine synthase	385
-0.00325853	0.017931	-0.0281142	-0.0376774	0.0245156	Q5T985	Inter-alpha-trypsin inhibitor heavy chain H2	386
0.000109705	0.00144403	-0.00141731	-0.000823114	-0.000649343	P19827	Inter-alpha-trypsin inhibitor heavy chain H1	387
-4.88E-05	-0.00099383	0.000892227	0.00403354	0.00814031	P20340-2	Isoform 2 of Ras-related protein Rab-6A	388
0.000664056	0.00147257	0.00180389	-0.00204557	0.00216496	P20618	Proteasome subunit beta type-1	389
-0.000552918	-0.000524215	0.000704067	-0.00553512	-0.010426	P20645	Cation-dependent mannose-6-phosphate receptor	390
-2.01E-05	7.95E-05	1.14E-05	-0.000114676	0.000146366	A0A0D9SFE5	Lamin B1, isoform CRA_a	391
0.00700246	-0.0727053	-0.0137686	-0.00823146	-0.220815	P20742	Pregnancy zone protein	392
0	0	0	0	0	P20774	Mimecan	393
0.00370162	-0.000542969	-0.00231846	-0.000692253	0.00175812	P20908	Collagen alpha-1(V) chain	394
-0.000532041	-0.000923876	-0.00150071	0.00207498	0.00288994	A0A0A0MTN3	Glutathione S-transferase	395
-0.000852483	0.00034073	-0.00130801	-0.00131165	0.000644798	E9PP21	Cysteine and glycine-rich protein 1	396
0.000258797	-0.00331145	0.00260657	-0.00420364	-0.0622482	Q60FE5	Filamin-A	397
-0.00149541	-0.00165133	-0.00119644	0.000397052	0.00249532	P21399	Cytoplasmic aconitate hydratase	398
-0.00342043	0.00349823	0.00426068	-0.0152202	0.00582493	P21589	5-nucleotidase	399
0.000655697	0.00141774	0.000396874	0.00541295	-0.00209472	P21741-2	Isoform 2 of Midkine	400
0.0315963	0.00256584	-0.0133541	0.0462893	-0.00438482	P21810	Biglycan	401
0.00392042	0.00237717	0.0080989	0.000762675	0.0157322	A6NNI4	Tetraspanin	402

-8.57E-05	0.000338934	4.86E-05	-0.00048901	0.000624141	H7BZ45	Catechol O-methyltransferase (Fragment)	403
-0.000177127	0.000700282	0.000100341	-0.00101036	0.00128956	M0R0P1	rRNA 2-O-methyltransferase fibrillarin (Fragment)	404
-0.000125156	8.37E-05	0.000421529	0.000220956	0.000378545	P22102	Trifunctional purine biosynthetic protein adenosine-3	405
0.000148174	0.000268136	-0.000143689	-0.000659281	0.00156253	D6RF62	Multifunctional protein ADE2	406
0.000507206	-0.00805502	0.00947035	0.00176248	-0.00659693	P22314-2	Isoform 2 of Ubiquitin-like modifier-activating enzyme 1	407
-0.00111324	0.00440124	0.00063064	-0.00635006	0.00810481	A0A087X1J7	Glutathione peroxidase	408
-0.000361304	-0.000511376	4.86E-05	-0.00223115	0.00237054	P22392	Nucleoside diphosphate kinase B	409
-0.000155689	-0.000236968	-0.000123927	-0.00108883	0.00192199	A0A087WUI2	Heterogeneous nuclear ribonucleoproteins A2/B1	410
-8.88E-05	0.000351203	5.03E-05	-0.000506711	0.000646734	B1APF7	cAMP-dependent protein kinase catalytic subunit beta (Fragment)	411
0.00128237	-0.00178938	-0.0206192	-0.0121759	0.000627283	P23142	Fibulin-1	412
0.001369	-0.000878347	-0.000496907	-0.00233573	0.000223273	P23142-4	Isoform C of Fibulin-1	413
-0.000246425	0.000192904	0.000766388	-0.000915628	0.000282247	P23229-4	Isoform Alpha-6X2A of Integrin alpha-6	414
9.09E-05	-0.00016587	0.000282738	-0.000250301	0.000243721	P23246	Splicing factor, proline- and glutamine-rich	415
-0.000263475	-0.000178906	-0.000411154	-0.0022312	0.00271923	P23258	Tubulin gamma-1 chain	416
-0.000167569	-0.00016184	-0.000392374	1.04E-05	-0.000293305	P23297	Protein S100-A1	417
0.000312827	-0.000882069	0.00115469	0.00107955	-0.000192598	P23381-2	Isoform 2 of Tryptophan--tRNA ligase, cytoplasmic	418
-0.00268415	-0.000285256	-0.00404526	-0.0123256	0.0135266	P23396	40S ribosomal protein S3	419
0.000793921	-0.00805981	0.00807013	0.0103033	-0.00854854	P23526	Adenosylhomocysteinase	420
0.00234441	0.00223027	-0.00295445	0.00394985	-0.00957879	P23528	Cofilin-1	421
0.000433886	-0.00133423	0.0023711	0.0023723	-0.00280525	P23921	Ribonucleoside-diphosphate reductase large subunit	422
0.000720373	-0.000376757	7.99E-05	0.00090554	-0.00019985	P24534	Elongation factor 1-beta	423
-5.83E-05	0.000230346	3.30E-05	-0.000332341	0.000424179	F2Z2Q9	Low molecular weight phosphotyrosine protein phosphatase	424
0.0118352	0.00583583	-0.00451759	0.0154694	-0.0391734	P24821-4	Isoform 4 of Tenascin	425

-4.04E-05	0.000159592	2.29E-05	-0.000230258	0.000293886	P25205	DNA replication licensing factor MCM3	426
-0.000391624	-0.000947597	0.000917705	0.00198902	-0.00137556	B1AH49	3-mercaptopyruvate sulfurtransferase	427
-0.00272745	0.00551382	-0.00673432	-0.00944982	0.000919507	P25398	40S ribosomal protein S12	428
-0.000148556	-0.000220118	-0.000343804	-0.000302153	0.000204599	P25705-2	Isoform 2 of ATP synthase subunit alpha, mitochondrial	429
-0.000525288	0.00130602	0.000828324	-0.00445691	0.00390004	P25786	Proteasome subunit alpha type-1	430
-0.000146379	0.000578718	8.29E-05	-0.000834967	0.0010657	A0A024RA52	Proteasome subunit alpha type	431
-0.000141279	0.000296239	0.000744071	-0.00299152	0.00101955	P25788-2	Isoform 2 of Proteasome subunit alpha type-3	432
-0.000591443	0.000409149	-0.000950296	-0.0031126	0.000469339	H0YMZ1	Proteasome subunit alpha type (Fragment)	433
-0.00312202	0.0211348	0.0163223	-0.0519769	0.101985	P26006	Integrin alpha-3	434
-2.82E-05	-0.000139227	0.000261896	-0.000159437	-0.000176219	P26022	Pentraxin-related protein PTX3	435
-0.000378238	-0.000369763	0.00031467	0.000209084	-0.00132452	P26038	Moesin	436
-0.000973695	0.00317848	0.000902709	-0.00206771	-0.00788684	P26373	60S ribosomal protein L13	437
0.000109404	0.000103036	-0.000343544	0.000419838	0.0017239	P26639	Threonine--tRNA ligase, cytoplasmic	438
-0.000498269	0.000418893	9.76E-05	-6.00E-05	0.00175574	P26640	Valine--tRNA ligase	439
0.00342722	-0.00569038	0.0027879	0.0179551	0.00176679	P26641	Elongation factor 1-gamma	440
-0.000551578	-0.000654734	-0.00028355	0.000236889	-0.0027939	P27105	Erythrocyte band 7 integral membrane protein	441
4.89E-05	-5.90E-06	0.00196957	0.000269034	-0.00422187	P27348	14-3-3 protein theta	442
0.000131359	-0.000257404	0.000235116	-0.000580924	-0.000384485	E9PJF0	Mitogen-activated protein kinase	443
0.000205198	0.000366958	-0.0022417	-0.000925041	-0.000728712	X1WI28	60S ribosomal protein L10 (Fragment)	444
-0.000322121	4.52E-05	-0.00060004	5.46E-06	-0.000684509	E9PJC7	Tetraspanin (Fragment)	445
-0.00205956	-4.01E-05	-0.00206393	-0.00213528	0.00020478	F8VPD4	CAD protein	446
-1.45E-05	5.75E-05	8.24E-06	-8.30E-05	0.000105875	P27797	Calreticulin	447
-0.000133391	-0.000247602	0.00145492	-0.00512532	0.000102397	P28066	Proteasome subunit alpha type-5	448
-1.61E-05	-7.93E-05	0.00014919	-9.08E-05	-0.000100384	P28070	Proteasome subunit beta type-4	449
3.49E-05	-9.10E-05	-0.00039409	-0.00219355	0.00228768	P28074	Proteasome subunit beta type-5	450
3.56E-05	-0.000847045	0.0007451	0.00176784	-0.000462337	P28482	Mitogen-activated protein kinase 1	451
0.0155405	-0.00496587	-0.0298981	0.0505835	-0.0263148	P29144	Tripeptidyl-peptidase 2	452

0.0013895	-2.11E-05	-0.000530151	0.0013126	0.000964419	P29317	Ephrin type-A receptor 2	453
0.000375656	0.000387817	6.18E-05	0.0007973	-0.00214009	B1AKC9	Ephrin type-B receptor 2	454
-0.00193128	-0.00226918	0.00300909	-0.00458902	-0.00210941	P29401	Transketolase	455
0.00154811	0.000284077	0.000102485	0.00276412	-0.00303183	E9PPR1	Elongation factor 1-delta (Fragment)	456
0.000129585	-0.000338486	0.000576019	0.000583049	-0.000147799	A0A0C4DFS0	MAWD binding protein, isoform CRA_d	457
0.000153391	-0.000143241	-0.000341962	0.000213431	-0.00132246	P30041	Peroxiredoxin-6	458
-0.000232161	0.000917859	0.000131517	-0.00132428	0.00169022	A0A2R8Y7Y9	Flavin reductase (NADPH)	459
0.000250978	0.00152183	0.000437412	0.00269104	-0.00593409	P30050	60S ribosomal protein L12	460
-0.000396233	0.000177208	0.000973638	0.00072823	-0.00346125	P30153	Serine/threonine-protein phosphatase 2A 65 kDa regulatory subunit A alpha isoform	461
-0.000366641	-0.00132003	0.00129242	0.000995608	-0.000850937	P30419-2	Isoform Short of Glycylpeptide N-tetradecanoyltransferase 1	462
0.000834071	0.00026276	0.00309486	-0.00128591	-0.0033608	P30685	HLA class I histocompatibility antigen, B-35 alpha chain	463
-6.42E-05	0.000129774	0.000516765	-0.00111701	0.000884443	P30501	HLA class I histocompatibility antigen, Cw-2 alpha chain	464
-0.000194739	3.79E-05	0.000828642	-0.00110704	0.000303882	P30504	HLA class I histocompatibility antigen, Cw-4 alpha chain	465
-8.04E-06	-3.97E-05	7.48E-05	-4.55E-05	-5.03E-05	A0A096LNY6	Adenylosuccinate lyase (Fragment)	466
-0.00105741	-0.00185641	0.00296795	-0.00299726	-0.00319544	C9J0K6	Sorcin	467
-8.37E-06	-2.09E-05	-3.41E-05	0.000420204	0.00109874	P30825	High affinity cationic amino acid transporter 1	468
0.000232212	-0.000259928	0.00344518	0.00641846	-0.00754402	P31146	Coronin-1A	469
-6.61E-05	-0.000326576	0.000614313	-0.000373981	-0.000413346	P31150	Rab GDP dissociation inhibitor alpha	470
-0.000783763	-0.000204662	-0.000757633	-4.64E-05	-0.000365702	P31153-2	Isoform 2 of S-adenosylmethionine synthase isoform type-2	471
-9.87E-05	-0.000487764	0.00091752	-0.000558567	-0.00061736	A0A087WYN0	Transporter	472
-8.32E-05	-0.000359595	0.000685407	-0.000628445	-0.000364698	P31689-2	Isoform 2 of DnaJ homolog subfamily A member 1	473
-0.00301567	0.00225661	0.000621432	-0.000233808	0.00990428	P31939	Bifunctional purine biosynthesis protein PURH	474

-0.000395747	0.000540743	-0.000130232	-0.00045694	-0.00141471	E9PCY7	Heterogeneous nuclear ribonucleoprotein H	475
-2.41E-05	-0.000118508	2.83E-05	-0.000143892	-0.000743142	P31946-2	Isoform Short of 14-3-3 protein beta/alpha	476
0.000159655	1.54E-05	-0.00124994	0.000276607	-0.000459509	P31948	Stress-induced-phosphoprotein 1	477
-0.000487304	-0.000298294	0.000298642	-0.000480412	0.00174825	P31949	Protein S100-A11	478
-8.13E-05	0.000321363	4.60E-05	-0.000463659	0.000591785	P32119	Peroxiredoxin-2	479
-0.000980954	0.00128884	-0.00107892	-0.00131574	-0.00578951	P32969	60S ribosomal protein L9	480
2.06E-05	0.000118023	-0.000132787	0.000287886	-0.00031135	P33176	Kinesin-1 heavy chain	481
0.000362933	-0.00113755	0.00114107	-0.000231573	-0.00156273	P33908	Mannosyl-oligosaccharide 1,2-alpha-mannosidase IA	482
-3.01E-05	0.000119109	1.71E-05	-0.000171849	0.000219337	C9J8M6	DNA replication licensing factor MCM7 (Fragment)	483
5.95E-05	0.000392491	0.000135593	0.00104618	-0.00205305	P34096	Ribonuclease 4	484
-0.000508234	-2.11E-05	0.000379604	0.000610003	0.000239485	P34896-2	Isoform 2 of Serine hydroxymethyltransferase, cytosolic	485
-0.00179493	-0.00188113	0.00160695	-0.00154557	0.00140183	P34897-3	Isoform 3 of Serine hydroxymethyltransferase, mitochondrial	486
-0.000142796	-0.000314244	0.000416046	0.000541774	-0.000171865	A0A087WTS8	Heat shock 70 kDa protein 4	487
-0.000920647	-0.000938896	0.00122714	-0.00343356	0.000530534	P35052	Glypican-1	488
-5.62E-05	0.000222324	3.19E-05	-0.000320767	0.000409406	C9J712	Profilin	489
-3.95E-05	-0.000195016	0.00036684	-0.000223325	-0.000246831	A0A2R8Y804	Catenin beta-1	490
-0.000341785	0.00135127	0.000193619	-0.00194959	0.00248833	K7EJT5	60S ribosomal protein L22 (Fragment)	491
0.000230054	0.000711974	-0.00103517	-0.00222528	0.00170773	E7ES19	Thrombospondin-4	492
0.000541448	-0.0106864	0.0140618	0.082524	0.0742291	P35527	Keratin, type I cytoskeletal 9	493
0.000356493	0.000341577	0.000223561	9.41E-05	2.19E-05	P35555	Fibrillin-1	494
-4.32E-05	0.00017064	2.45E-05	-0.000246197	0.00031423	P35558-2	Isoform 2 of Phosphoenolpyruvate carboxykinase, cytosolic [GTP]	495
0.00169781	-0.0130231	0.0057211	0.057676	-0.0612535	P35579	Myosin-9	496
-0.00142463	0.00114462	0.00126976	0.000836789	0.00148829	P35580	Myosin-10	497
-2.28E-05	-0.00064068	0.00168504	0.000324754	-0.00107791	P35606-2	Isoform 2 of Coatomer subunit beta	498
0.00311595	-0.000272347	0.00267249	-0.00460643	0.00143091	P35613-3	Isoform 3 of Basigin	499

0.00134886	0.00347131	-0.00186842	0.00789511	0.00514604	P35625	Metalloproteinase inhibitor 3	500
1.29E-05	-0.00200543	0.00283353	-0.00133067	-0.0011976	P35858	Insulin-like growth factor-binding protein complex acid labile subunit	501
-0.00608186	-0.00865754	-0.00842736	0.038729	0.0161971	P35908	Keratin, type II cytoskeletal 2 epidermal	502
-0.00101905	0.000654054	-0.000100962	-0.00322531	-0.000289995	P35998	26S proteasome regulatory subunit 7	503
-0.00014381	-0.000138893	-0.00033674	8.90E-06	-0.000251718	P36406-3	Isoform Gamma of E3 ubiquitin-protein ligase TRIM23	504
-0.00828812	0.00013254	-0.00702169	-0.0239469	-0.0120678	P36578	60S ribosomal protein L4	505
0.00156167	-0.00493073	-0.00275324	0.0060281	-0.00162022	P36871	Phosphoglucomutase-1	506
0.00122487	-7.44E-05	-0.00551267	0.00522438	0.00820362	P36955	Pigment epithelium-derived factor	507
0.00168114	-0.00237185	0.00350885	0.00319349	-0.00642888	P37802	Transgelin-2	508
-0.000248851	-0.000453221	-0.000166447	-0.000666721	0.000580141	P37837	Transaldolase	509
-0.000144263	0.000443973	0.00139365	-2.83E-05	-0.00269281	P38606-2	Isoform 2 of V-type proton ATPase catalytic subunit A	510
-0.00134581	0.000516034	-0.00117183	-0.00390657	0.000999897	P38646	Stress-70 protein, mitochondrial	511
-0.000325993	0.00172957	-0.00196748	-0.000718081	0.000719154	P39019	40S ribosomal protein S19	512
-0.00219734	0.00141932	-0.000779936	-0.00969141	-0.000381856	P39023	60S ribosomal protein L3	513
-0.00068889	0.00286275	-0.00133052	-0.00211376	-0.00314131	A0A087X0K0	Collagen alpha-1(XV) chain	514
0.000313859	0.000643477	0.000402506	-0.00131756	0.00139722	P39060-2	Isoform 3 of Collagen alpha-1(XVIII) chain	515
-0.00122982	0.000734347	-0.00693946	-0.00355655	0.00560835	P40227	T-complex protein 1 subunit zeta	516
-0.00124811	0.00493446	0.000707044	-0.00711939	0.00908673	P40261	Nicotinamide N-methyltransferase	517
-0.001217	0.00180836	-0.000395936	-0.00205413	-0.00313134	P40429	60S ribosomal protein L13a	518
2.47E-05	-2.92E-05	-2.66E-05	0.00125048	-0.00204775	P40763-3	Isoform 3 of Signal transducer and activator of transcription 3	519
-6.09E-05	0.00024063	3.45E-05	-0.000347179	0.000443117	C9JRL4	Malate dehydrogenase, cytoplasmic (Fragment)	520
-0.000231767	-0.00130719	0.00212447	0.00172085	-0.00440124	P41091	Eukaryotic translation initiation factor 2 subunit 3	521
0.00642236	0.000137318	0.00052837	0.0294815	0.00953408	P41221-2	Isoform 2 of Protein Wnt-5a	522
-0.00200845	0.00290968	-0.000855069	-0.0077803	0.00953356	P41250	Glycine--tRNA ligase	523
-0.000549324	-0.000927338	0.00105799	-0.00240224	0.00225838	P41252	Isoleucine--tRNA ligase, cytoplasmic	524

-7.71E-05	-7.44E-05	-0.000180497	4.77E-06	-0.000134924	P42025	Beta-centractin	525
-8.07E-05	-0.000126028	3.19E-05	-0.00167776	-0.00284583	P42224	Signal transducer and activator of transcription 1-alpha/beta	526
-7.97E-05	0.00031509	4.51E-05	-0.000454608	0.000580233	K7EK35	Signal transducer and activator of transcription	527
-0.00296821	-0.00402216	0.00391309	-0.00729137	-0.00497049	A0A0A0MSS8	Aldo-keto reductase family 1 member C3	528
-0.000908371	0.00139398	-0.000644992	-0.00262804	0.00330112	Q71UM5	40S ribosomal protein S27-like	529
-0.000411302	-0.00150031	0.000505152	-0.00474427	0.00713232	P42766	60S ribosomal protein L35	530
-0.000284874	0.000195913	0.0010743	-0.00162016	0.000658349	B4DKB2	Endothelin-converting enzyme 1	531
0.000629533	-0.00192814	0.00262303	0.00173489	-0.00281063	P43034	Platelet-activating factor acetylhydrolase IB subunit alpha	532
0.00125711	0.000874874	-0.000832776	0.000893041	-0.00198137	P43121	Cell surface glycoprotein MUC18	533
-4.42E-05	-0.000218196	0.000410443	-0.000249869	-0.00027617	C9JXG8	Ran-specific GTPase-activating protein (Fragment)	534
-0.000687873	4.21E-05	0.000308917	-0.00277042	-0.00258435	A0A0C4DFS8	Nicotinamide phosphoribosyltransferase	535
-0.00109504	-0.00045198	0.000924894	-0.00411267	0.00138969	P43686	26S proteasome regulatory subunit 6B	536
-8.88E-05	0.000350967	5.03E-05	-0.000506371	0.000646299	P45974-2	Isoform Short of Ubiquitin carboxyl-terminal hydrolase 5	537
0.000363789	0.000457328	0.000838874	-0.000175276	0.00133838	P46063	ATP-dependent DNA helicase Q1	538
-0.000332447	-0.000565642	0.000461455	-0.000648068	-0.000827115	P46087-3	Isoform 3 of Probable 28S rRNA (cytosine(4447)-C(5))-methyltransferase	539
-5.55E-05	0.000219313	3.14E-05	-0.000316422	0.00040386	F6S6P2	Large proline-rich protein BAG6 (Fragment)	540
-0.000243628	0.000314292	4.21E-05	-0.000237968	0.000562215	P46459-2	Isoform 2 of Vesicle-fusing ATPase	541
-1.67E-05	-1.61E-05	-3.91E-05	1.03E-06	-2.92E-05	E9PRZ0	Dual-specificity mitogen-activated protein kinase kinase 3 (Fragment)	542
-0.00174236	0.0017043	-0.00105069	-0.00373204	-0.00354367	P46776	60S ribosomal protein L27a	543
-0.000648168	-0.00657359	-0.00478264	0.00219181	0.00704347	A0A2R8Y6J3	60S ribosomal protein L5 (Fragment)	544
-0.00316509	0.00111462	0.00320598	-0.00507483	-0.0121254	G3V1B3	60S ribosomal protein L21	545
-6.18E-05	0.000160186	-0.000750391	0.000116105	-0.00112699	P46779	60S ribosomal protein L28	546

-0.00669759	0.0103097	-0.00503478	-0.0134573	0.0221304	A0A024R4M0	40S ribosomal protein S9	547
-0.00115798	-0.000256128	-0.00283411	-0.00702683	0.0118005	M0R0F0	40S ribosomal protein S5 (Fragment)	548
-0.000171726	-5.08E-05	-0.000277322	-0.000885869	0.00236597	P46783	40S ribosomal protein S10	549
0.00012786	-0.00126785	0.0006544	0.000998697	-0.00188405	P46940	Ras GTPase-activating-like protein IQGAP1	550
-2.41E-05	9.53E-05	1.36E-05	-0.000137438	0.000175417	C9J8R8	Glycogenin-1 (Fragment)	551
2.86E-05	-0.000651841	0.00100345	0.000118034	-0.000590282	P47755	F-actin-capping protein subunit alpha-2	552
-0.000114696	-0.00168583	0.0019747	0.000175876	-0.00048998	B1AK87	F-actin-capping protein subunit beta	553
-0.000898344	0.000195569	0.000843327	-0.00473381	0.00396027	P47897-2	Isoform 2 of Glutamine--tRNA ligase	554
0.000142199	0.000937665	0.000323934	0.00249933	-0.00490475	P47914	60S ribosomal protein L29	555
-0.0111776	-0.00291064	-0.0107646	-0.000300648	-0.00364267	P48059	LIM and senescent cell antigen-like-containing domain protein 1	556
-3.73E-05	0.000147462	2.11E-05	-0.000212757	0.000271549	B4E3S5	cDNA FLJ50386, highly similar to Glioma pathogenesis-related protein 1	557
1.01E-05	-0.000856611	0.00104991	-0.000570217	-0.000392529	P48147	Prolyl endopeptidase	558
-3.21E-05	0.000126905	1.82E-05	-0.000183097	0.000233693	P48426-2	Isoform 2 of Phosphatidylinositol 5-phosphate 4-kinase type-2 alpha	559
1.94E-05	0.000279643	-0.000308737	-8.65E-05	-0.000974155	P48444	Coatomer subunit delta	560
0.00664156	0.0126131	0.0231052	0.0398904	-0.059333	K4DIA7	Tetraspanin (Fragment)	561
-0.000251993	0.000443746	-0.000586202	7.26E-05	0.000620305	R4GMR5	26S proteasome non-ATPase regulatory subunit 8	562
0.000365298	-0.000573597	-0.00649734	-0.00185673	0.0123381	P48643	T-complex protein 1 subunit epsilon	563
0	0	0	0	0	P48723	Heat shock 70 kDa protein 13	564
-1.95E-05	7.72E-05	1.11E-05	-0.000111354	0.000142126	P48735-2	Isoform 2 of Isocitrate dehydrogenase [NADP], mitochondrial	565
-0.000172307	0.000681226	9.76E-05	-0.000982865	0.00125447	C9JMA2	Mannan-binding lectin serine protease 1 (Fragment)	566
-0.000214878	-0.000316842	0.00135293	-0.000674262	-0.00161356	P48960-2	Isoform 2 of CD97 antigen	567

9.24E-05	-0.00306941	0.00316814	-0.000512024	0.0031285	P49189	4-trimethylaminobutyraldehyde dehydrogenase	568
-0.000215835	1.48E-05	-0.000275733	-0.00092435	0.00110379	P49207	60S ribosomal protein L34	569
0.00846404	-0.0282346	0.032513	0.0197997	-0.00333645	P49327	Fatty acid synthase	570
0.00513127	0.000604335	-0.00819816	-0.00182551	0.0074268	P49368	T-complex protein 1 subunit gamma	571
-0.000102087	0.000268205	-2.16E-05	-0.000668933	0.000800732	P49411	Elongation factor Tu, mitochondrial	572
-0.000213762	0.000845121	0.000121095	-0.00121933	0.00155628	A0A1B0GVU0	Alpha-aminoadipic semialdehyde dehydrogenase (Fragment)	573
-0.00014464	-0.000386234	0.00119995	0.00074702	-0.0029128	P49588	Alanine--tRNA ligase, cytoplasmic	574
0.000116084	-0.00112167	0.0015137	0.00267772	0.00110171	P49591	Serine--tRNA ligase, cytoplasmic	575
0.00027776	-0.000396565	0.00086151	-0.00242372	-0.000432438	P49720	Proteasome subunit beta type-3	576
-0.000233183	0.000348566	0.00102167	-0.000577295	0.00381694	P49721	Proteasome subunit beta type-2	577
0.00107957	0.000127041	-0.000179659	-0.000429757	0.000253217	F5H4Z8	Thrombospondin-3	578
-4.13E-05	0.000508954	0.000913034	-0.00057923	0.000915632	P49748-2	Isoform 2 of Very long-chain specific acyl-CoA dehydrogenase, mitochondrial	579
-4.25E-05	-0.000209954	0.00039494	-0.000240431	-0.000265738	P49756	RNA-binding protein 25	580
0.000208952	-0.000713517	0.0008317	0.00143762	-0.00236944	P49888	Estrogen sulfotransferase	581
-0.000290881	-0.000102926	0.000326029	-0.00115688	0.0010022	P49915-2	Isoform 2 of GMP synthase [glutamine-hydrolyzing]	582
-0.000574189	0.000525339	0.00103117	-0.0033546	0.00224745	P50281	Matrix metalloproteinase-14	583
-4.40E-05	-0.00038388	0.000210113	-0.000664558	0.00106057	P50395	Rab GDP dissociation inhibitor beta	584
4.62E-05	-0.000159352	-0.00013071	0.000698766	-0.00055893	E9PKH2	Serpin H1	585
-2.07E-05	-3.01E-05	0.000697445	0.000402992	-0.00190768	H7C3I1	Hsc70-interacting protein (Fragment)	586
8.01E-06	-0.000255867	0.000530453	0.00064685	-0.00167764	P50552	Vasodilator-stimulated phosphoprotein	587
-0.00155309	0.00163406	0.00210568	-0.000170852	0.0046636	P50570-3	Isoform 3 of Dynamin-2	588
-0.0023565	0.000244536	-0.000172109	-0.00418325	-0.000178878	E7EPB3	60S ribosomal protein L14	589
0.00477588	-0.00199224	-0.00985974	-0.000611044	0.00795378	P50990	T-complex protein 1 subunit theta	590
0.00172196	-0.000962729	-0.0109451	-0.00635759	0.0148984	P50991	T-complex protein 1 subunit delta	591
-0.00111613	-0.000762143	0.00200251	0.00102279	-0.00289913	P51148	Ras-related protein Rab-5C	592
-0.00161368	-0.00047548	0.00226318	-0.0019597	-0.00155434	P51149	Ras-related protein Rab-7a	593
-0.00257115	0.00372862	-0.00649504	0.00635836	0.010619	P51570	Galactokinase	594

-0.00187951	0.001782	-0.000608981	-0.00514826	0.00336035	P51665	26S proteasome non-ATPase regulatory subunit 7	595
-0.000104692	0.000413907	5.93E-05	-0.00059718	0.000762203	D6R9D2	Neuronal membrane glycoprotein M6-a (Fragment)	596
-0.00081748	0.00323195	0.000463097	-0.00466302	0.00595159	P51857-2	Isoform 2 of 3-oxo-5-beta-steroid 4-dehydrogenase	597
-0.00015367	0.000607544	8.71E-05	-0.000876557	0.00111878	P51884	Lumican	598
-0.000170215	-3.44E-06	0.000356112	-0.000864374	0.000305604	P51991-2	Isoform 2 of Heterogeneous nuclear ribonucleoprotein A3	599
0.000106807	-0.00132751	0.000455743	0.00301449	-0.00248637	P52209-2	Isoform 2 of 6-phosphogluconate dehydrogenase, decarboxylating	600
-8.39E-05	0.000331842	4.75E-05	-0.000478777	0.000611081	P52272-2	Isoform 2 of Heterogeneous nuclear ribonucleoprotein M	601
-3.06E-05	0.000121159	1.74E-05	-0.000174807	0.000223112	P52333-4	Isoform 3 of Tyrosine-protein kinase JAK3	602
-0.00117784	0.000169611	0.000408955	-0.00323611	0.000816337	B4DK69	Aldo-keto reductase family 1 member C2	603
0.000416292	-0.00465005	0.00433611	0.00259024	-0.00829485	P52907	F-actin-capping protein subunit alpha-1	604
-7.44E-05	0.000294286	4.22E-05	-0.000424592	0.000541922	P53004	Biliverdin reductase A	605
-0.00112379	-0.00280968	0.000958521	0.000448301	-0.0024019	P53396	ATP-citrate synthase	606
-0.000216167	-0.0010593	0.000827545	-0.00136118	0.00204641	P53618	Coatomer subunit beta	607
-0.00128587	-0.00268714	0.00549321	-0.00187618	-0.00641934	P53621	Coatomer subunit alpha	608
0.000973911	-0.00212789	0.000448538	0.00273051	0.00211953	P53634	Dipeptidyl peptidase 1	609
0.00102644	-0.00114088	-2.34E-05	0.00693421	0.0153942	P53675-2	Isoform 2 of Clathrin heavy chain 2	610
-5.21E-05	0.000206114	2.95E-05	-0.000297379	0.000379555	A8MZH8	Pituitary tumor-transforming gene 1 protein-interacting protein	611
-0.000276016	-0.00107242	0.00140322	-0.000260997	0.00199034	P54136	Arginine--tRNA ligase, cytoplasmic	612
-2.11E-05	-6.46E-05	0.000475627	0.000167902	-0.00111512	P54289-4	Isoform 4 of Voltage-dependent calcium channel subunit alpha-2/delta-1	613
-5.35E-05	0.000211693	3.03E-05	-0.000305428	0.000389829	A0A0C4DGZ5	Tyrosine--tRNA ligase	614
-0.000206388	-0.00045012	0.00051347	-0.000184259	-0.000630301	A6NJA2	Ubiquitin carboxyl-terminal hydrolase 14	615
-0.00142549	-0.000497726	0.00156389	-0.00473398	0.000234056	P54709	Sodium/potassium-transporting ATPase subunit beta-3	616

**-0.00012728**	**-0.000122928**	**-0.000298034**	**7.88E-06**	**-0.000222785**	**P55001-3**	**Isoform B of Microfibrillar-associated protein 2**	**617**
**0.000310553**	**-9.95E-05**	**0.00124483**	**0.00166759**	**-0.000257123**	**P55060-3**	**Isoform 3 of Exportin-2**	**618**
**-0.0040756**	**-0.00093548**	**0.00149395**	**-0.0156844**	**-0.0104416**	**P55072**	**Transitional endoplasmic reticulum ATPase**	**619**
**-0.00123372**	**-0.00244167**	**0.00336591**	**-0.00176676**	**-0.000284094**	**P55083**	**Microfibril-associated glycoprotein 4**	**620**
**-0.000540634**	**-0.00177725**	**0.00171139**	**-0.000479006**	**-0.00374666**	**H0YHC3**	**Nucleosome assembly protein 1-like 1 (Fragment)**	**621**
**-8.96E-05**	**-8.65E-05**	**-0.000209782**	**5.54E-06**	**-0.000156815**	**D6RHU3**	**Caspase-6 (Fragment)**	**622**
**-0.000433473**	**0.000176399**	**-0.000829315**	**0.0038191**	**0.0111935**	**P55263-3**	**Isoform 3 of Adenosine kinase**	**623**
**-3.90E-05**	**0.000154096**	**2.21E-05**	**-0.000222328**	**0.000283766**	**P55290-5**	**Isoform 5 of Cadherin-13**	**624**
**-0.000226412**	**-2.69E-05**	**0.000523468**	**-0.000374855**	**0.000363062**	**P55735**	**Protein SEC13 homolog**	**625**
**0.000169421**	**-0.00021494**	**0.000607697**	**-0.000249323**	**-0.00182664**	**P55786-2**	**Isoform 2 of Puromycin-sensitive aminopeptidase**	**626**
**-0.000751758**	**-0.000385947**	**1.99E-05**	**-0.000694738**	**0.00166223**	**P55884**	**Eukaryotic translation initiation factor 3 subunit B**	**627**
**-0.000752007**	**0.000373795**	**-0.000525599**	**-0.00132897**	**0.00188781**	**P56192**	**Methionine--tRNA ligase, cytoplasmic**	**628**
**0.00012814**	**-0.000796171**	**0.000419027**	**0.00100637**	**-0.000318576**	**P56537**	**Eukaryotic translation initiation factor 6**	**629**
**0.00928817**	**0.0136445**	**0.010005**	**0.083433**	**-0.0406766**	**P58166**	**Inhibin beta E chain**	**630**
**0.000236066**	**-0.000931761**	**0.00117617**	**-0.00172938**	**-0.00131518**	**P58546**	**Myotrophin**	**631**
**-0.000534963**	**-0.000447465**	**-0.000730327**	**-0.000317675**	**0.00211853**	**P59998**	**Actin-related protein 2/3 complex subunit 4**	**632**
**0.0408995**	**0.0113613**	**0.00359708**	**0.0657608**	**-0.129816**	**E9PJK1**	**Tetraspanin**	**633**
**-0.00107951**	**-0.000512231**	**-0.0018456**	**-0.000211023**	**-0.00211713**	**P60174-1**	**Isoform 2 of Triosephosphate isomerase**	**634**
**-0.000554524**	**-0.0010314**	**-0.00157231**	**-0.000707761**	**-0.00179037**	**P60201**	**Myelin proteolipid protein**	**635**
**-0.000581659**	**0.00010606**	**0.00109198**	**-0.00193416**	**0.00118056**	**E5RGA2**	**Eukaryotic translation initiation factor 3 subunit E**	**636**
**-0.00098868**	**0.00144652**	**0.00262593**	**-0.00495585**	**-0.010882**	**F8W1R7**	**Myosin light polypeptide 6**	**637**
**0.0264574**	**0.00532614**	**-0.164526**	**0.0558208**	**0.134401**	**P60709**	**Actin, cytoplasmic 1**	**638**
**-0.00133242**	**0.00060159**	**0.00480489**	**0.00656673**	**0.0134878**	**P60842**	**Eukaryotic initiation factor 4A-I**	**639**
**0.000550992**	**0.000932347**	**-0.000680406**	**-0.00148098**	**0.00235606**	**P60866**	**40S ribosomal protein S20**	**640**

-0.000393484	0.000549603	0.000537521	0.000467843	2.52E-05	B1ALA9	Ribose-phosphate pyrophosphokinase 1	641
-0.000343521	0.00130408	0.00105486	-0.00591611	0.0040721	P60900	Proteasome subunit alpha type-6	642
4.96E-05	-0.000395756	0.00139642	-0.000733598	-0.000168646	P60903	Protein S100-A10	643
-0.00246922	-0.00431271	0.00128994	0.00416926	-0.00491682	P60953	Cell division control protein 42 homolog	644
0.000953165	-0.000552147	-0.000561101	0.0011021	0.00469854	F6RFD5	Destrin	645
-0.000582209	-0.000462383	-0.000831902	-1.53E-05	-0.000560119	P61006	Ras-related protein Rab-8A	646
2.13E-05	-0.0013466	0.00179841	0.00148899	0.000320926	P61019	Ras-related protein Rab-2A	647
-7.63E-05	-0.00128011	0.000599742	-0.000912294	-0.00611732	P61026	Ras-related protein Rab-10	648
0.000206481	-0.000958288	0.000487603	0.000455692	-4.96E-05	P61081	NEDD8-conjugating enzyme Ubc12	649
-0.00030219	-0.00119691	0.00171973	-0.000156237	-0.00127445	X6RFL8	Ras-related protein Rab-14 (Fragment)	650
-0.00172953	-0.00300784	0.000428122	-0.00295157	-0.00392166	P61158	Actin-related protein 3	651
-0.00211198	-0.00341168	0.00265152	-0.000295931	-0.00471461	P61160	Actin-related protein 2	652
2.19E-07	-0.000196712	0.0019389	0.00168463	0.000453339	R4GMT0	Alpha-centractin	653
0.000231565	-0.00279257	-0.00330918	0.00156569	0.0129409	P84077	ADP-ribosylation factor 1	654
-6.40E-05	-0.000270443	-9.63E-05	-0.000754142	0.00168026	P61221	ATP-binding cassette sub-family E member 1	655
-0.00412117	-0.0131427	-0.00342555	0.0162022	0.00775105	P61224-3	Isoform 3 of Ras-related protein Rap-1b	656
-0.00297402	-0.00182105	0.00220802	-0.00767106	0.0109063	D6RG13	40S ribosomal protein S3a (Fragment)	657
-0.00128464	-0.000894897	-0.000116624	-0.00471201	0.00346245	J3QRI7	60S ribosomal protein L26 (Fragment)	658
-2.26E-05	8.92E-05	1.28E-05	-0.000128647	0.000164197	K7EMD0	Proteasome activator complex subunit 3 (Fragment)	659
-0.00527303	0.00281746	0.000446195	-0.0137073	-0.000553897	P61313	60S ribosomal protein L15	660
-0.00123744	-0.000409842	0.000455404	0.00025349	0.0020438	P61353	60S ribosomal protein L27	661
-0.000389636	0.000573525	-0.000114717	-0.00207446	0.00157069	M0R0A1	60S ribosomal protein L37a	662
-0.000197959	-0.000422316	0.00363313	0.00569037	-0.00255189	P61586	Transforming protein RhoA	663
-0.000195139	0.00130451	-0.000649871	-0.00352846	-0.0057187	P61769	Beta-2-microglobulin	664
-0.000176729	-0.000170687	-0.000413823	1.09E-05	-0.000309338	P61812	Transforming growth factor beta-2 proprotein	665
0.00116895	-0.00561698	0.00377061	-0.00268859	0.00607166	P61970	Nuclear transport factor 2	666

0.000104853	0.000626597	-0.00044406	0.000233123	0.00147697	Q5T6W2	Heterogeneous nuclear ribonucleoprotein K (Fragment)	667
5.79E-05	-0.000645705	8.86E-06	-0.00110971	0.00160671	P61981	14-3-3 protein gamma	668
0.000228465	-2.42E-05	6.24E-05	0.000491319	-0.000431659	B7Z317	Tetraspanin	669
-0.000340244	0.000425194	-0.000399054	-0.00436798	0.00743911	A0A2R8Y623	40S ribosomal protein S7 (Fragment)	670
0.000175275	-0.00234458	0.00128996	0.00211407	0.00103322	P62136	Serine/threonine-protein phosphatase PP1-alpha catalytic subunit	671
8.70E-05	0.00134216	0.000475151	0.00298095	-0.000528719	P62140	Serine/threonine-protein phosphatase PP1-beta catalytic subunit	672
-0.00104935	-0.000131697	0.000573222	-0.00402621	-0.00139992	P62191	26S proteasome regulatory subunit 4	673
-0.000724241	0.000604748	-0.000460184	-0.00255271	-0.00158193	P62195-2	Isoform 2 of 26S proteasome regulatory subunit 8	674
-0.0184109	0.0151087	-0.00448683	-0.0459845	0.0325662	P62241	40S ribosomal protein S8	675
-0.00483603	-0.0010922	0.00196692	-0.00973827	0.00446329	P62249	40S ribosomal protein S16	676
0.000151228	-0.000953513	-0.000302792	-3.50E-05	-0.00069498	P62258	14-3-3 protein epsilon	677
-0.000351485	0.00153093	-0.000491866	0.00014203	0.000671711	A0A2R8Y811	40S ribosomal protein S14 (Fragment)	678
-0.000505404	0.00357554	-0.00135317	4.78E-05	-0.00295128	P62266	40S ribosomal protein S23	679
-0.00188061	0.00497433	0.00205144	-0.00987853	0.00467135	P62269	40S ribosomal protein S18	680
-0.000139561	0.00149413	-0.000650913	-0.00139393	-0.00093533	P62273	40S ribosomal protein S29	681
-0.00316114	-0.00355945	0.00027268	-0.00996271	-0.00793716	P62277	40S ribosomal protein S13	682
-0.00776242	0.00709466	-0.0104211	-0.0173177	0.00893908	P62280	40S ribosomal protein S11	683
-0.000153133	0.00060542	8.67E-05	-0.000873493	0.00111487	P62304	Small nuclear ribonucleoprotein E	684
-9.57E-05	0.000378391	5.42E-05	-0.000545938	0.000696801	J3QLR7	Small nuclear ribonucleoprotein Sm D1	685
0.000233774	-0.00105606	-7.92E-05	-9.84E-05	0.0014101	K7ERG4	Small nuclear ribonucleoprotein Sm D2	686
-0.000136391	-0.000131727	-0.000319367	8.44E-06	-0.000238731	P62318-2	Isoform 2 of Small nuclear ribonucleoprotein Sm D3	687
-0.000512304	-4.99E-05	-0.000728197	0.000624663	0.00107447	P62330	ADP-ribosylation factor 6	688

-0.00150096	-0.00112016	0.000279486	-0.00660007	0.00472606	P62333	26S proteasome regulatory subunit 10B	689
-0.00365194	0.00529983	0.00286754	-0.00706955	-0.0140037	P62424	60S ribosomal protein L7a	690
-0.00481516	-0.00079152	-0.00437062	-0.014895	0.0187863	P62701	40S ribosomal protein S4, X isoform	691
0.0211151	-0.0233888	0.0206904	0.0537907	-0.0396956	P62736	Actin, aortic smooth muscle	692
-0.000402727	0.000479655	-0.00028899	-0.00080347	-0.000756753	K7EMA7	60S ribosomal protein L23a	693
0.000194597	-0.000364076	-0.00078066	-0.004494	0.00123899	A2A3R5	40S ribosomal protein S6	694
-0.158482	-0.0267038	-0.159055	-0.0658778	-0.0250197	P62805	Histone H4	695
-0.000690345	0.000370666	0.00151552	0.00448476	0.000725481	E7END7	Ras-related protein Rab-1A	696
-0.00885132	-0.0023563	0.00632383	0.0167978	-0.0103775	P62826	GTP-binding nuclear protein Ran	697
-0.000373657	0.000415712	0.000134389	-0.000478436	-0.000956806	C9JD32	60S ribosomal protein L23 (Fragment)	698
-0.000253588	0.00100258	0.000143656	-0.0014465	0.00184623	P62834	Ras-related protein Rap-1A	699
-0.000562767	0.000724265	-0.00047468	-0.0026278	0.0029748	K7EM56	40S ribosomal protein S15	700
-0.00242363	0.00106719	-0.00267422	-0.00246863	0.00319291	P62847-2	Isoform 2 of 40S ribosomal protein S24	701
0.00107186	0.00172866	0.00215377	-0.000144754	2.36E-05	P62851	40S ribosomal protein S25	702
-0.00238897	0.0034885	-0.00413858	-0.00203636	0.00331758	P62854	40S ribosomal protein S26	703
-0.000277168	-0.00136948	0.0025761	-0.00156828	-0.00173335	P62861	40S ribosomal protein S30	704
-0.00167053	-0.00352021	0.00422733	-0.00224042	-0.0041409	P62873-2	Isoform 2 of Guanine nucleotide-binding protein G(I)/G(S)/G(T) subunit beta-1	705
-0.000864494	-0.00107563	0.00148414	0.000478013	-0.00537617	P62879	Guanine nucleotide-binding protein G(I)/G(S)/G(T) subunit beta-2	706
-0.00134521	0.00194694	3.01E-05	0.00146316	-0.00618978	E5RI99	60S ribosomal protein L30 (Fragment)	707
-0.000366492	0.00144895	0.000207615	-0.00209052	0.00266821	H7C2W9	60S ribosomal protein L31 (Fragment)	708
-1.10E-05	-0.000747188	-0.000920935	0.00122112	-0.00214617	P62906	60S ribosomal protein L10a	709
-0.00139824	0.0015773	-0.000123848	-0.00086262	-0.00408604	D3YTB1	60S ribosomal protein L32 (Fragment)	710
-0.000458238	-0.0021952	-0.00226578	-0.00651348	0.00899428	Q5VVC8	60S ribosomal protein L11	711
-0.00157967	-0.000360099	-0.00280019	-0.00504416	-0.00726231	E9PKZ0	60S ribosomal protein L8 (Fragment)	712

-0.00273477	-0.00532688	0.00599916	-0.00411179	-0.00999702	P62937	Peptidyl-prolyl cis-trans isomerase A	713
-2.95E-05	0.000116812	1.67E-05	-0.000168535	0.000215108	Q1JUQ5	Peptidylprolyl isomerase	714
-0.00249691	-0.00233508	0.000283567	0.00545894	-0.00275619	P62979	Ubiquitin-40S ribosomal protein S27a	715
-0.000410967	0.000235518	0.000392817	-0.00132711	0.000566218	P62995-3	Isoform 3 of Transformer-2 protein homolog beta	716
-0.00348594	0.000425086	0.00322714	0.00220561	-0.00314369	P63000	Ras-related C3 botulinum toxin substrate 1	717
-0.000667972	0.000800546	-0.000410635	-0.000865472	0.000727395	A0A087WYD1	AP-2 complex subunit beta (Fragment)	718
-0.000829379	-0.00131254	0.00174637	-0.00319637	-0.00441142	P63104	14-3-3 protein zeta/delta	719
0.000136643	0.000360084	0.000485378	-0.000514446	-0.00150235	P63151	Serine/threonine-protein phosphatase 2A 55 kDa regulatory subunit B alpha isoform	720
5.92E-05	0.00018999	-0.000162658	0.00191479	0.000588323	Q96FJ2	Dynein light chain 2, cytoplasmic	721
-0.000421573	3.15E-05	-0.000256583	-0.000684896	4.88E-05	J3KT73	60S ribosomal protein L38	722
-0.000909753	0.000418264	0.000253203	7.72E-05	-0.000340928	Q8WVC2	40S ribosomal protein S21	723
0.000305386	-0.000325857	-0.000678768	-0.00222137	0.00460574	I3L397	Eukaryotic translation initiation factor 5A (Fragment)	724
-0.00978577	0.000522391	0.0069345	-0.0220605	0.0286149	P63244	Receptor of activated protein C kinase 1	725
-4.58E-05	0.000181063	2.59E-05	-0.000261235	0.000333424	P63313	Thymosin beta-10	726
-0.000820702	-0.00355326	0.00346904	0.00402834	0.00122889	P67775	Serine/threonine-protein phosphatase 2A catalytic subunit alpha isoform	727
0.0020137	0.000264964	-0.000673399	0.000627862	-0.00770148	P67809	Nuclease-sensitive element-binding protein 1	728
-3.82E-05	-0.000188677	0.000354915	-0.000216065	-0.000238807	P67870	Casein kinase II subunit beta	729
-0.00800868	-0.02736	0.0158543	0.00185265	-0.0349389	P68104	Elongation factor 1-alpha 1	730
-0.0103752	0.0410187	0.00587745	-0.0591814	0.0755354	P68363	Tubulin alpha-1B chain	731
-0.0382185	-0.0088417	-0.030438	-0.0301562	-0.0646425	P68366	Tubulin alpha-4A chain	732
8.16E-05	-0.000142579	5.34E-05	0.00109732	0.00220556	P68366-2	Isoform 2 of Tubulin alpha-4A chain	733
-0.0783275	-0.0235276	-0.0292544	-0.114708	0.0466674	P68371	Tubulin beta-4B chain	734
-9.71E-05	-3.04E-05	0.000150611	-0.000274491	0.000194291	A0A2R8YDP2	Casein kinase II subunit alpha	735

**-0.000323979**	**-0.000178165**	**0.000176676**	**-0.000772885**	**-0.000148795**	**P68402**	**Platelet-activating factor acetylhydrolase IB subunit beta**	**736**
**-0.0407334**	**0.116484**	**-0.179505**	**0.102883**	**-0.105707**	**K7EK07**	**Histone H3 (Fragment)**	**737**
**-0.0947841**	**-0.0806933**	**-0.175884**	**-0.014778**	**-0.102094**	**P68871**	**Hemoglobin subunit beta**	**738**
**-0.0425067**	**-0.11453**	**-0.114956**	**-0.0999008**	**0.120734**	**P69905**	**Hemoglobin subunit alpha**	**739**
**-0.000162736**	**-0.000157172**	**-0.000381056**	**1.01E-05**	**-0.000284845**	**H7C521**	**SRSF protein kinase 2 (Fragment)**	**740**
**0.00127474**	**0.000570806**	**-0.0102054**	**-0.00695463**	**0.00864284**	**P78371-2**	**Isoform 2 of T-complex protein 1 subunit beta**	**741**
**0.000805618**	**-0.000250662**	**0.00137529**	**0.002148**	**-0.00322496**	**Q5TA02**	**Glutathione S-transferase omega-1 (Fragment)**	**742**
**-4.85E-05**	**0.00794605**	**0.00320568**	**0.00634957**	**0.00873193**	**P78527-2**	**Isoform 2 of DNA-dependent protein kinase catalytic subunit**	**743**
**-0.000101247**	**-0.000766988**	**0.00112736**	**0.000700872**	**0.00209181**	**P78539-4**	**Isoform 4 of Sushi repeat-containing protein SRPX**	**744**
**-0.0015157**	**-0.00203808**	**0.000409184**	**-0.00036586**	**-0.0115882**	**P83110**	**Serine protease HTRA3**	**745**
**-0.00140985**	**9.86E-06**	**-2.37E-05**	**-0.00295607**	**-0.00146656**	**C9JXB8**	**60S ribosomal protein L24**	**746**
**3.91E-05**	**0.000375274**	**-0.00024233**	**0.000451768**	**-0.00167066**	**Q969Q0**	**60S ribosomal protein L36a-like**	**747**
**-0.000367791**	**0.00145408**	**0.000208351**	**-0.00209793**	**0.00267767**	**P84085**	**ADP-ribosylation factor 5**	**748**
**-3.11E-05**	**-0.000153739**	**0.000289194**	**-0.000176055**	**-0.000194586**	**G3V279**	**Enhancer of rudimentary homolog**	**749**
**-0.0001051**	**0.000415517**	**5.95E-05**	**-0.000599503**	**0.000765168**	**P84095**	**Rho-related GTP-binding protein RhoG**	**750**
**-0.00254678**	**0.00020559**	**0.000554835**	**-0.00754737**	**-0.000237801**	**J3QR09**	**Ribosomal protein L19**	**751**
**0.000724487**	**0.00733229**	**-0.000371683**	**-0.00692056**	**-0.00126753**	**P98160**	**Basement membrane-specific heparan sulfate proteoglycan core protein**	**752**
**-4.94E-05**	**0.000195399**	**2.80E-05**	**-0.00028192**	**0.000359825**	**H0Y8X9**	**Calcium-transporting ATPase type 2C member 1 (Fragment)**	**753**
**-0.000102088**	**0.000403609**	**5.78E-05**	**-0.000582322**	**0.000743239**	**Q00266**	**S-adenosylmethionine synthase isoform type-1**	**754**
**-0.21297**	**-0.0328548**	**-0.135289**	**0.227081**	**0.045754**	**Q00610-2**	**Isoform 2 of Clathrin heavy chain 1**	**755**
**-0.000264796**	**0.000577718**	**0.000194356**	**-0.00229574**	**-0.00172374**	**Q00765**	**Receptor expression-enhancing protein 5**	**756**
**9.13E-05**	**0.000159305**	**0.000543616**	**0.00133033**	**-0.00314038**	**H0YLA4**	**Sorbitol dehydrogenase**	**757**
**-0.000353247**	**0.000347391**	**0.00108926**	**-0.00121265**	**0.00177217**	**A0A1W2PP35**	**Heterogeneous nuclear ribonucleoprotein U (Fragment)**	**758**

-0.000124779	0.000493321	7.07E-05	-0.000711758	0.000908443	Q01082-3	Isoform 2 of Spectrin beta chain, non-erythrocytic 1	759
-2.23E-05	0.000386673	-0.000375262	0.000163081	-0.000436774	J3KP15	Serine/arginine-rich-splicing factor 2 (Fragment)	760
-1.95E-05	-9.62E-05	0.000180929	-0.000110146	-0.000121739	H0YK10	N-acetylgalactosamine kinase (Fragment)	761
-0.000121423	-0.000187411	0.000578797	0.00105192	-0.00279184	Q01518-2	Isoform 2 of Adenylyl cyclase-associated protein 1	762
0.000313563	-0.00202475	0.00239819	0.000611448	0.00129302	Q01581	Hydroxymethylglutaryl-CoA synthase, cytoplasmic	763
-0.000595334	0.00080055	0.00136462	-0.000825055	-0.00159431	Q01813	ATP-dependent 6-phosphofructokinase, platelet type	764
-0.000204902	0.000428928	0.000226747	-0.000584685	0.000465738	Q02388-2	Isoform 2 of Collagen alpha-1(VII) chain	765
-0.00629488	0.00163528	-0.00130399	-0.0121554	0.00312754	Q02543	60S ribosomal protein L18a	766
-0.000641883	-0.00116594	-0.0004635	-0.0019629	0.00645871	Q02750-2	Isoform 2 of Dual specificity mitogen-activated protein kinase kinase 1	767
0.000988434	-0.000550822	-0.000604996	0.000913992	0.00446082	Q02790	Peptidyl-prolyl cis-trans isomerase FKBP4	768
0.0289105	-0.0107061	0.00192791	0.0888258	0.00451829	Q02809	Procollagen-lysine,2-oxoglutarate 5-dioxygenase 1	769
-0.00386058	0.00389122	0.0017402	-0.0112039	-0.00598931	Q02878	60S ribosomal protein L6	770
0.00348636	0.000497396	-8.20E-05	0.00417402	0.000405008	Q03135	Caveolin-1	771
-0.0011838	0.000123692	0.000287324	-0.00283852	-0.000422136	Q03405	Urokinase plasminogen activator surface receptor	772
-2.74E-05	-0.000389944	0.000107166	-0.000523451	0.00151223	Q2TAM5	RELA protein	773
-0.000132496	5.67E-05	0.000533451	-0.000753363	0.0002538	C9J6B6	Eukaryotic translation initiation factor 4 gamma 1 (Fragment)	774
-4.26E-05	0.00016853	2.41E-05	-0.000243153	0.000310345	Q04756	Hepatocyte growth factor activator	775
1.96E-05	-0.000411187	0.00140847	0.00152683	-0.000418005	Q04760-2	Isoform 2 of Lactoylglutathione lyase	776
-0.0306069	-0.0100831	0.0319433	-0.0849338	0.00203887	Q04828	Aldo-keto reductase family 1 member C1	777
0.000262989	-0.000309515	0.000529343	0.000544828	-0.00142391	Q04917	14-3-3 protein eta	778
-3.71E-05	0.000146546	2.10E-05	-0.000211435	0.000269862	Q04941	Proteolipid protein 2	779

**-0.000491771**	**0.000152195**	**0.000359543**	**-0.000537825**	**-0.000613747**	**A0A0D9SFB1**	**Dynamin-1**	**780**
**-4.14E-05**	**0.00016377**	**2.35E-05**	**-0.000236285**	**0.000301579**	**H0YC15**	**Tyrosine-protein phosphatase non-receptor type 12 (Fragment)**	**781**
**-1.85E-05**	**-0.000102272**	**-0.00103063**	**0.000521463**	**0.00251697**	**Q5T760**	**Serine/arginine-rich-splicing factor 11 (Fragment)**	**782**
**-0.000595897**	**0.00204914**	**-0.0025788**	**-0.0098205**	**0.0010565**	**A0A087WW43**	**Inter-alpha-trypsin inhibitor heavy chain H3**	**783**
**-0.000341469**	**0.000562428**	**0.00015557**	**0.000543086**	**-0.000818172**	**Q06210-2**	**Isoform 2 of Glutamine--fructose-6-phosphate aminotransferase [isomerizing] 1**	**784**
**-2.55E-05**	**-0.000126141**	**0.000237281**	**-0.000144452**	**-0.000159656**	**Q06278**	**Aldehyde oxidase**	**785**
**-0.00275086**	**-0.000504359**	**0.00130278**	**-0.0107946**	**0.00346709**	**Q06830**	**Peroxiredoxin-1**	**786**
**-0.00295237**	**-0.000514523**	**-0.00196684**	**-0.00662415**	**-0.00515455**	**Q07020-2**	**Isoform 2 of 60S ribosomal protein L18**	**787**
**-0.000114827**	**0.000453975**	**6.50E-05**	**-0.00065499**	**0.000835988**	**Q07021**	**Complement component 1 Q subcomponent-binding protein, mitochondrial**	**788**
**-0.000127668**	**0.000504744**	**7.23E-05**	**-0.000728238**	**0.000929477**	**Q07666-3**	**Isoform 3 of KH domain-containing, RNA-binding, signal transduction-associated protein 1**	**789**
**-0.000100891**	**0.000271971**	**0.000691513**	**-0.000113997**	**-0.00113019**	**G5E9S8**	**Kinesin light chain 1**	**790**
**-0.00233478**	**0.00206943**	**0.000240177**	**-0.0067316**	**-0.00483748**	**Q07954**	**Prolow-density lipoprotein receptor-related protein 1**	**791**
**0.000110968**	**0.000105608**	**0.000130921**	**0.00164109**	**0.000790979**	**Q07960**	**Rho GTPase-activating protein 1**	**792**
**-7.16E-05**	**-0.00179629**	**0.00231541**	**-0.00188509**	**-0.00161269**	**Q08211**	**ATP-dependent RNA helicase A**	**793**
**-0.000129177**	**0.000312453**	**-0.000314398**	**-0.00071589**	**-0.00353522**	**C9JH92**	**Quinone oxidoreductase (Fragment)**	**794**
**0.211279**	**-0.00418034**	**0.10883**	**0.140857**	**-0.18436**	**Q08380**	**Galectin-3-binding protein**	**795**
**0.0738544**	**0.0732392**	**-0.0223386**	**0.0559499**	**-0.0518927**	**Q08431**	**Lactadherin**	**796**
**-6.34E-05**	**0.000214913**	**0.000221381**	**-0.000715977**	**-0.000108639**	**A0A2R8Y484**	**Leukocyte surface antigen CD47 (Fragment)**	**797**
**0.00127483**	**-0.000140288**	**-0.000789591**	**-0.000217781**	**-0.00097553**	**Q10589-2**	**Isoform 2 of Bone marrow stromal antigen 2**	**798**
**0.000556789**	**0.000760545**	**-0.000148458**	**0.000861083**	**0.00120173**	**A0A0U1RQV3**	**EGF-containing fibulin-like extracellular matrix protein 1 (Fragment)**	**799**

**-5.59E-05**	**0.000221172**	**3.17E-05**	**-0.000319105**	**0.000407285**	**C9J5G4**	**Follistatin-related protein 1 (Fragment)**	**800**
**-5.47E-06**	**2.16E-05**	**3.10E-06**	**-3.12E-05**	**3.99E-05**	**Q12874**	**Splicing factor 3A subunit 3**	**801**
**0.000308937**	**-0.000957276**	**0.000774141**	**0.000111367**	**-0.000253518**	**B4DLR2**	**Prolyl endopeptidase FAP**	**802**
**-2.70E-05**	**0.000106774**	**1.53E-05**	**-0.000154052**	**0.000196622**	**Q12907**	**Vesicular integral-membrane protein VIP36**	**803**
**-6.87E-05**	**3.48E-05**	**0.000271427**	**-0.000390794**	**0.00013982**	**Q13085-3**	**Isoform 3 of Acetyl-CoA carboxylase 1**	**804**
**-5.22E-05**	**0.000206433**	**2.96E-05**	**-0.000297839**	**0.000380143**	**C9JFE4**	**COP9 signalosome complex subunit 1**	**805**
**-0.000160159**	**-0.000634577**	**0.00258605**	**0.000224043**	**-0.00486576**	**F2Z2F3**	**Uncharacterized protein**	**806**
**-0.000171626**	**0.000272081**	**0.000153777**	**-0.00125118**	**0.000664384**	**A8MU58**	**Aminoacyl tRNA synthase complex-interacting multifunctional protein 2**	**807**
**-0.00245294**	**0.00269337**	**-0.00109328**	**-0.00990705**	**0.00837786**	**Q13200**	**26S proteasome non-ATPase regulatory subunit 2**	**808**
**-0.000238259**	**-0.000394335**	**8.89E-06**	**-0.000763046**	**-0.000750821**	**Q13263-2**	**Isoform 2 of Transcription intermediary factor 1-beta**	**809**
**-0.000551303**	**-0.00091626**	**-0.000167456**	**-0.00051816**	**-0.00139978**	**Q13283**	**Ras GTPase-activating protein-binding protein 1**	**810**
**0.000355035**	**0.000529112**	**-0.000144597**	**5.34E-05**	**0.000703132**	**Q13308-5**	**Isoform 5 of Inactive tyrosine-protein kinase 7**	**811**
**-3.36E-05**	**-0.00010572**	**2.40E-05**	**9.91E-05**	**0.000187835**	**Q13325-2**	**Isoform 2 of Interferon-induced protein with tetratricopeptide repeats 5**	**812**
**-0.000402801**	**-0.00125665**	**0.00142564**	**-9.52E-05**	**0.00246026**	**Q13347**	**Eukaryotic translation initiation factor 3 subunit I**	**813**
**-1.73E-05**	**6.86E-05**	**9.82E-06**	**-9.89E-05**	**0.000126236**	**H0YGS3**	**Microfibrillar-associated protein 5 (Fragment)**	**814**
**-0.0160771**	**0.000112011**	**-0.00847303**	**0.00336106**	**0.00717961**	**Q13418**	**Integrin-linked protein kinase**	**815**
**-2.34E-05**	**9.23E-05**	**1.32E-05**	**-0.000133227**	**0.000170042**	**F8WC54**	**Disintegrin and metalloproteinase domain-containing protein 9**	**816**
**-0.000661407**	**0.000577667**	**0.00019124**	**0.000599946**	**0.00472284**	**Q13509**	**Tubulin beta-3 chain**	**817**
**-1.81E-05**	**-8.10E-06**	**7.31E-05**	**-0.00014142**	**-0.000427125**	**Q13547**	**Histone deacetylase 1**	**818**
**1.17E-05**	**7.71E-05**	**2.66E-05**	**0.000205404**	**-0.000403089**	**Q13619-2**	**Isoform 2 of Cullin-4A**	**819**
**-0.00032064**	**-0.000164082**	**0.000216151**	**-0.000158131**	**0.00178792**	**E9PKL9**	**GDP-L-fucose synthase (Fragment)**	**820**

**-0.00292133**	**0.0020157**	**-0.0048638**	**-0.00334181**	**-0.000892072**	**Q13642-1**	**Isoform 1 of Four and a half LIM domains protein 1**	**821**
**0.000968672**	**-0.00162704**	**0.00313308**	**-0.00369985**	**0.001826**	**Q13740-2**	**Isoform 2 of CD166 antigen**	**822**
**-0.000356321**	**-0.000205254**	**1.13E-05**	**0.000311349**	**0.00340074**	**Q5STU3**	**Spliceosome RNA helicase DDX39B**	**823**
**-0.000464598**	**0.000156994**	**-0.000348368**	**0.00045408**	**0.000158112**	**Q13885**	**Tubulin beta-2A chain**	**824**
**-0.00119691**	**0.000132184**	**0.000920108**	**0.00138001**	**0.00215839**	**D6RF44**	**Heterogeneous nuclear ribonucleoprotein D0 (Fragment)**	**825**
**0.000312268**	**0.00140851**	**0.000164731**	**0.00232793**	**-0.000443921**	**A0A1W2PPX5**	**Lysosome membrane protein 2**	**826**
**0.01106**	**0.00244747**	**-0.00491557**	**0.0112321**	**-0.00622462**	**Q14112-2**	**Isoform 2 of Nidogen-2**	**827**
**-5.35E-05**	**3.38E-05**	**-0.000415749**	**-0.000680911**	**0.000652681**	**Q14118**	**Dystroglycan**	**828**
**-0.00143531**	**-0.00146923**	**0.000837247**	**-0.00169275**	**0.00167761**	**Q14152-2**	**Isoform 2 of Eukaryotic translation initiation factor 3 subunit A**	**829**
**-0.00017078**	**-1.99E-05**	**0.000778761**	**-0.000970563**	**0.000185753**	**Q14166**	**Tubulin--tyrosine ligase-like protein 12**	**830**
**-0.000111045**	**-6.24E-05**	**5.78E-05**	**0.000146014**	**-0.000277887**	**Q14195-2**	**Isoform LCRMP-4 of Dihydropyrimidinase-related protein 3**	**831**
**-0.0272666**	**0.00479168**	**-0.0198365**	**-0.0431221**	**-0.019807**	**Q14204**	**Cytoplasmic dynein 1 heavy chain 1**	**832**
**-0.000140026**	**-0.000894923**	**0.00217911**	**0.000701951**	**-0.00115276**	**Q14240**	**Eukaryotic initiation factor 4A-II**	**833**
**-6.81E-05**	**0.000269248**	**3.86E-05**	**-0.000388469**	**0.000495817**	**J3QLD9**	**Flotillin-2**	**834**
**-1.45E-05**	**0.000528284**	**3.75E-05**	**0.000682899**	**-0.00070794**	**Q14289-2**	**Isoform 2 of Protein-tyrosine kinase 2-beta**	**835**
**-8.28E-05**	**-0.000140472**	**0.000191523**	**-0.000554192**	**-0.000214029**	**Q14315-2**	**Isoform 2 of Filamin-C**	**836**
**-7.35E-05**	**0.00029058**	**4.16E-05**	**-0.000419246**	**0.000535099**	**K7EM34**	**Guanidinoacetate N-methyltransferase (Fragment)**	**837**
**-6.96E-05**	**0.000275091**	**3.94E-05**	**-0.000396899**	**0.000506576**	**Q5QPP1**	**UDP-glucose 4-epimerase (Fragment)**	**838**
**0.00016113**	**-0.00132052**	**-0.000178994**	**0.00222204**	**-0.00205531**	**A0A0U1RQH7**	**RNA-binding protein 39 (Fragment)**	**839**
**0.00125364**	**-0.000481454**	**-0.000262727**	**0.00214345**	**-1.05E-06**	**Q14517**	**Protocadherin Fat 1**	**840**
**-0.000488183**	**0.000883594**	**0.00141686**	**0.0013774**	**-0.00773245**	**Q14520-2**	**Isoform 2 of Hyaluronan-binding protein 2**	**841**
**-0.00156636**	**0.00298148**	**-0.00276724**	**-0.0010942**	**-0.00623969**	**Q14624-4**	**Isoform 4 of Inter-alpha-trypsin inhibitor heavy chain H4**	**842**
**0.0062588**	**-0.00078152**	**0.00143376**	**0.0210114**	**-0.00300064**	**Q14697-2**	**Isoform 2 of Neutral alpha-glucosidase AB**	**843**

**0.000407501**	**-5.41E-05**	**0.000992048**	**0.000259493**	**-0.0046045**	**Q14703**	**Membrane-bound transcription factor site-1 protease**	**844**
**0.0137395**	**0.0282576**	**-0.0138646**	**0.0331619**	**-0.0471133**	**Q14764**	**Major vault protein**	**845**
**-7.91E-05**	**-0.000729631**	**0.00156366**	**0.00111485**	**-0.0039949**	**F6XGT7**	**Prostaglandin reductase 1**	**846**
**4.13E-06**	**-0.000267605**	**0.00100902**	**0.000431459**	**0.000644652**	**Q14974**	**Importin subunit beta-1**	**847**
**-1.07E-05**	**-5.29E-05**	**9.95E-05**	**-6.06E-05**	**-6.69E-05**	**Q14997-3**	**Isoform 3 of Proteasome activator complex subunit 4**	**848**
**-0.000219657**	**-0.000209664**	**0.000692071**	**-0.00258023**	**0.00327077**	**Q15008**	**26S proteasome non-ATPase regulatory subunit 6**	**849**
**-0.00121069**	**-0.00328656**	**0.00292795**	**-0.00243389**	**-0.000329233**	**B5MCX3**	**Septin-2**	**850**
**-0.000118622**	**-9.76E-05**	**-4.78E-05**	**-0.00116341**	**-6.72E-05**	**Q15029-2**	**Isoform 2 of 116 kDa U5 small nuclear ribonucleoprotein component**	**851**
**0.0012854**	**-0.000389861**	**-0.00028998**	**0.00247417**	**0.000835211**	**E5RIP4**	**Zinc transporter ZIP14 (Fragment)**	**852**
**-0.000310247**	**0.00122658**	**0.000175753**	**-0.00176969**	**0.00225873**	**Q15063**	**Periostin**	**853**
**-6.23E-05**	**0.000246404**	**3.53E-05**	**-0.000355509**	**0.000453749**	**Q15063-2**	**Isoform 2 of Periostin**	**854**
**0.000770606**	**-0.000546191**	**-0.000841845**	**-0.0016867**	**0.00166572**	**Q15063-3**	**Isoform 3 of Periostin**	**855**
**0.0410765**	**0.0277243**	**-0.00835058**	**0.11146**	**-0.0957673**	**Q15063-5**	**Isoform 5 of Periostin**	**856**
**-3.86E-05**	**0.000152542**	**2.19E-05**	**-0.000220085**	**0.000280903**	**M0R389**	**Platelet-activating factor acetylhydrolase IB subunit gamma (Fragment)**	**857**
**0.00396841**	**0.000880417**	**-0.00165463**	**0.00595427**	**-0.00252936**	**Q15113**	**Procollagen C-endopeptidase enhancer 1**	**858**
**0.000255151**	**-0.000933914**	**-0.000170063**	**0.00212137**	**-0.000392392**	**Q15149**	**Plectin**	**859**
**0.000422767**	**-0.000270667**	**0.000781287**	**-0.000439994**	**-0.00327387**	**Q15181**	**Inorganic pyrophosphatase**	**860**
**-0.00166102**	**-0.00219733**	**-0.000648788**	**-0.00101432**	**-0.00276436**	**Q15185-4**	**Isoform 4 of Prostaglandin E synthase 3**	**861**
**-5.61E-05**	**-0.000859018**	**6.53E-05**	**-0.00168209**	**0.00225639**	**C9JYS8**	**Non-POU domain-containing octamer-binding protein (Fragment)**	**862**
**0**	**0**	**0**	**0**	**0**	**Q15286-2**	**Isoform 2 of Ras-related protein Rab-35**	**863**
**-0.000152137**	**0.000144847**	**0.000372336**	**-0.00128597**	**-0.000526397**	**Q15365**	**Poly(rC)-binding protein 1**	**864**
**-0.000435455**	**-0.000149658**	**0.00111257**	**-0.00162836**	**0.000949351**	**F8VXH9**	**Poly(rC)-binding protein 2 (Fragment)**	**865**

-0.000561862	-0.000150292	0.000229159	-0.00139944	-9.03E-07	R4GMY8	Elongin-C	866
0.000299653	-0.000233259	0.000339334	-0.000808474	0.00136801	B8ZZU8	Elongin-B	867
-0.000330485	-0.000489976	-0.000724904	0.00325209	0.000562781	Q15389-2	Isoform 2 of Angiopoietin-1	868
-0.000135614	-0.000580677	0.00131038	-0.000860052	-0.000642175	Q15393	Splicing factor 3B subunit 3	869
-0.000167345	-5.66E-05	-0.000256849	-0.000318202	0.00173112	Q15404-2	Isoform 2 of Ras suppressor protein 1	870
-5.98E-05	-0.000175576	0.00139617	0.000526758	-0.00333227	C9J177	Protein phosphatase 1 regulatory subunit 7 (Fragment)	871
0.000775465	-0.000976305	0.0013515	7.03E-06	-0.00139695	Q15493	Regucalcin	872
0.0026651	-0.00728576	0.000702986	0.00643828	0.0075648	Q15555-4	Isoform 4 of Microtubule-associated protein RP/EB family member 2	873
0.0473701	0.037648	0.0222245	0.195614	0.0386706	Q15582	Transforming growth factor-beta-induced protein ig-h3	874
0.00360096	-0.00170545	0.00460698	0.0139342	0.000746193	Q15758	Neutral amino acid transporter B(0)	875
0.000105652	-0.000105616	-0.000185933	-0.000833217	9.22E-05	Q15836	Vesicle-associated membrane protein 3	876
-0.000548127	-0.00194606	0.00259256	0.00135643	0.00101657	Q15907	Ras-related protein Rab-11B	877
-0.000248474	0.000982354	0.000140759	-0.00141733	0.00180899	Q15916	Zinc finger and BTB domain-containing protein 6	878
-0.000499443	-0.00138537	0.00353017	-0.000363532	-0.00399402	E7ES33	Septin-7	879
-5.15E-05	0.000203477	2.92E-05	-0.000293574	0.000374699	Q16186	Proteasomal ubiquitin receptor ADRM1	880
0.00115047	0.000549597	-0.000567909	0.00428972	-0.00588639	Q16270-2	Isoform 2 of Insulin-like growth factor-binding protein 7	881
-0.000194975	-0.00468781	0.00105735	0.00637584	-0.00580472	Q16394	Exostosin-1	882
-0.000267303	2.87E-05	0.00064589	8.42E-05	0.00137799	Q16401-2	Isoform 2 of 26S proteasome non-ATPase regulatory subunit 5	883
-6.28E-05	-0.00031037	0.000583829	-0.000355423	-0.000392834	F5GY55	DNA damage-binding protein 1	884
-0.000659637	-0.0051764	0.0107531	0.00213089	-0.0130456	Q16555-2	Isoform 2 of Dihydropyrimidinase-related protein 2	885
-9.01E-05	0.000356324	5.11E-05	-0.0005141	0.000656165	A0A0U1RQT9	Synaptophysin-like protein 1 (Fragment)	886
-2.37E-05	0.000144204	0.000477191	0.000827307	-0.0011132	E9PC52	Histone-binding protein RBBP7	887
-0.000897596	0.000525398	-0.001979	-0.00207104	0.00223636	Q16610	Extracellular matrix protein 1	888

-0.00030803	-0.000494742	-0.000332932	-0.00277817	-0.000624761	Q16629-3	Isoform 3 of Serine/arginine-rich splicing factor 7	889
1.96E-05	-3.43E-05	1.28E-05	0.000264247	0.000531125	D6RCR4	Drebrin (Fragment)	890
0.00125033	-5.02E-05	-0.000168711	0.00399935	-0.000931048	Q16658	Fascin	891
-5.10E-05	0.000201464	2.89E-05	-0.000290671	0.000370993	Q16719	Kynureninase	892
-4.51E-05	0.000178453	2.56E-05	-0.000257471	0.000328619	Q16772	Glutathione S-transferase A3	893
-0.0282338	0.133695	0.176505	-0.13948	-0.355331	Q16777	Histone H2A type 2-C	894
-0.000461103	0.000574311	-0.000293338	-0.00041756	-0.00138179	Q16853	Membrane primary amine oxidase	895
-1.05E-05	4.16E-05	5.96E-06	-6.00E-05	7.66E-05	E9PQI3	Thioredoxin reductase 1, cytoplasmic (Fragment)	896
-0.000334083	0.00132082	0.000189256	-0.00190566	0.00243226	H0YLX2	DNA-binding protein RFX7	897
-3.92E-05	0.000154832	2.22E-05	-0.000223389	0.00028512	Q31612	HLA class I histocompatibility antigen, B-73 alpha chain	898
0.00216378	-0.000987984	0.00241549	0.00574517	-0.00225771	Q32P28-4	Isoform 4 of Prolyl 3-hydroxylase 1	899
-0.00535266	-0.000359327	-0.00124944	-0.0156461	-0.00857183	Q3ZCM7	Tubulin beta-8 chain	900
-0.000118149	-0.000358394	0.000400303	-0.000721827	0.00213382	Q3ZCW2	Galectin-related protein	901
0.00039602	0.000515328	0.000991018	0.000207659	0.00161953	Q53H96-2	Isoform 2 of Pyrroline-5-carboxylate reductase 3	902
8.90E-05	-0.00139113	0.0013694	0.000495631	-0.0025427	Q58FF8	Putative heat shock protein HSP 90-beta 2	903
-8.81E-06	3.48E-05	4.99E-06	-5.03E-05	6.42E-05	Q5JTH9-2	Isoform 2 of RRP12-like protein	904
-0.000227387	6.21E-05	-1.25E-05	-0.00139921	-0.0024036	Q5SSJ5	Heterochromatin protein 1-binding protein 3	905
-0.00539244	0.00171473	-0.00465109	-0.00725464	-0.00681001	Q5T4S7-3	Isoform 3 of E3 ubiquitin-protein ligase UBR4	906
-6.30E-05	0.00024893	3.57E-05	-0.000359153	0.0004584	A0A2R8YCY2	Protein furry homolog	907
-8.15E-06	3.22E-05	4.62E-06	-4.65E-05	5.93E-05	H3BLS7	Vacuolar protein sorting-associated protein 13D (Fragment)	908
-0.000154589	-0.000436834	0.000877524	-0.00182025	3.76E-05	A0A0C4DGH0	CD276 antigen	909
0.00390547	-0.000929137	-0.000853464	0.00338448	0.00194472	Q68BL8	Olfactomedin-like protein 2B	910
-6.77E-05	0.000267694	3.84E-05	-0.000386226	0.000492954	F5H6I7	Atlastin-3	911
-2.28E-05	9.00E-05	1.29E-05	-0.000129803	0.000165672	Q6NZI2-2	Isoform 2 of Caveolae-associated protein 1	912
2.89E-05	-0.000217768	0.000391921	-2.29E-05	-0.000301277	Q96GK7	Fumarylacetoacetate hydrolase domain-containing protein 2A	913

**0.00090304**	**0.00113051**	**-0.000183067**	**0.00203235**	**-0.00475207**	**Q6UVK1**	**Chondroitin sulfate proteoglycan 4**	**914**
**7.06E-05**	**-2.19E-05**	**0.000308014**	**-0.00100699**	**6.11E-05**	**Q6UXN9**	**WD repeat-containing protein 82**	**915**
**-5.75E-05**	**0.000227196**	**3.26E-05**	**-0.000327795**	**0.000418377**	**Q6UY14-2**	**Isoform 2 of ADAMTS-like protein 4**	**916**
**-6.39E-05**	**5.18E-05**	**2.39E-05**	**-0.000628344**	**0.00106963**	**Q6YHK3-2**	**Isoform 2 of CD109 antigen**	**917**
**-0.000810288**	**0.00022067**	**0.000278508**	**-0.000699359**	**-0.00229574**	**Q6ZNJ1-2**	**Isoform 2 of Neurobeachin-like protein 2**	**918**
**0.000480435**	**0.00010353**	**0.000120677**	**0.000780015**	**-0.000508059**	**Q6ZUX7**	**LHFPL tetraspan subfamily member 2 protein**	**919**
**-3.07E-05**	**0.000121305**	**1.74E-05**	**-0.000175017**	**0.000223381**	**K7EQ37**	**Protein unc-13 homolog D (Fragment)**	**920**
**-0.139048**	**-5.62E-05**	**-0.100542**	**-0.169364**	**-0.132406**	**Q71U36**	**Tubulin alpha-1A chain**	**921**
**0.000124652**	**0.000352981**	**0.000879184**	**-0.00108556**	**-0.00170407**	**Q76LX8-3**	**Isoform 3 of A disintegrin and metalloproteinase with thrombospondin motifs 13**	**922**
**0.000133134**	**-0.000232753**	**8.71E-05**	**0.00179131**	**0.00360046**	**Q76M96**	**Coiled-coil domain-containing protein 80**	**923**
**-0.00047895**	**-0.00130244**	**-0.000438859**	**0.000673555**	**0.00297483**	**Q7KZF4**	**Staphylococcal nuclease domain-containing protein 1**	**924**
**-0.000188765**	**-0.000356451**	**0.000191503**	**-0.0005424**	**-0.000119329**	**Q7L2H7**	**Eukaryotic translation initiation factor 3 subunit M**	**925**
**-0.000489126**	**4.15E-05**	**0.000896779**	**0.00109626**	**-0.000513783**	**Q7L576-2**	**Isoform 2 of Cytoplasmic FMR1-interacting protein 1**	**926**
**-0.000491407**	**-0.000187832**	**-0.000454522**	**-0.000197312**	**0.000100678**	**Q7LDG7**	**RAS guanyl-releasing protein 2**	**927**
**0.0187926**	**0.00464024**	**-0.00642517**	**0.0482842**	**0.00246749**	**Q7Z304**	**MAM domain-containing protein 2**	**928**
**-5.62E-05**	**0.000222296**	**3.19E-05**	**-0.000320727**	**0.000409355**	**Q7Z3C6-3**	**Isoform 3 of Autophagy-related protein 9A**	**929**
**-2.77E-05**	**-0.000136948**	**0.00025761**	**-0.000156828**	**-0.000173335**	**Q7Z3U7-2**	**Isoform 2 of Protein MON2 homolog**	**930**
**-0.000275789**	**-0.00184513**	**0.000275922**	**-0.00346564**	**0.00464868**	**J3QS45**	**L-xylulose reductase (Fragment)**	**931**
**-3.98E-05**	**-0.000196482**	**0.000369598**	**-0.000225004**	**-0.000248687**	**Q96S86**	**Hyaluronan and proteoglycan link protein 3**	**933**
**0.00339146**	**-0.0158036**	**0.0164703**	**0.00895639**	**0.0270915**	**Q86UX7-2**	**Isoform 2 of Fermitin family homolog 3**	**934**
**-0.000321118**	**0.000404296**	**0.000835991**	**-0.00116293**	**0.000835667**	**Q86VP6-2**	**Isoform 2 of Cullin-associated NEDD8-dissociated protein 1**	**935**
**-7.96E-06**	**3.15E-05**	**4.51E-06**	**-4.54E-05**	**5.80E-05**	**Q86X95**	**Corepressor interacting with RBPJ 1**	**936**

0	0	0	0	0	C9JJ68	Dihydrofolate reductase 2, mitochondrial (Fragment)	937
0.000101941	-0.000139506	0.000760167	0.00271162	0.000811857	Q86YZ3	Hornerin	938
-5.75E-05	0.000227307	3.26E-05	-0.000327956	0.000418582	Q8IWA5-3	Isoform 3 of Choline transporter-like protein 2	939
0.00136996	-0.000549548	-0.00137188	0.00269532	0.00337379	Q8IWU5-2	Isoform 2 of Extracellular sulfatase Sulf-2	940
-1.70E-05	6.73E-05	9.64E-06	-9.71E-05	0.000123911	Q9NQ36-3	Isoform 3 of Signal peptide, CUB and EGF-like domain-containing protein 2	941
-0.000186261	0.000736394	0.000105516	-0.00106246	0.00135606	K7EQL5	Pseudokinase FAM20A (Fragment)	942
-0.0510054	-0.1282	-0.139679	0.121418	-0.307827	Q8IXS2	Dynein regulatory complex subunit 2	943
-0.000110563	0.00025367	0.00142418	0.000867018	0.000486984	E9PCT1	Serine/arginine repetitive matrix protein 1	944
-0.0020023	0.000306684	-0.00130244	-0.00423719	0.0011728	Q8IZ83-3	Isoform 3 of Aldehyde dehydrogenase family 16 member A1	945
-0.000326969	-0.000745046	0.000490878	-0.000597445	-0.00105861	Q8N0Y7	Probable phosphoglycerate mutase 4	946
-2.23E-05	8.81E-05	1.26E-05	-0.000127159	0.000162298	A0A075B6E5	Protein enabled homolog (Fragment)	947
-6.31E-05	0.000249291	3.57E-05	-0.000359674	0.000459065	Q8NBJ5	Procollagen galactosyltransferase 1	948
-4.11E-05	-0.000577606	0.000938963	0.00020434	0.000907673	Q8NBM8	Prenylcysteine oxidase-like	949
-0.000142816	0.000564631	8.09E-05	-0.000814643	0.00103976	Q8NBN3-3	Isoform 3 of Transmembrane protein 87A	950
-0.000222407	-0.0019093	0.00229343	0.00113732	-0.000729115	Q8NBZ7	UDP-glucuronic acid decarboxylase 1	951
0.000221945	-0.000120411	-0.000139325	0.000170812	0.000939755	Q8NCL9	Protein APCDD1-like	952
-3.73E-05	0.000147531	2.11E-05	-0.000212857	0.000271677	F5GWG3	Retinoic acid-induced protein 3 (Fragment)	953
0.00244491	-0.00126907	-0.0024842	-0.0002679	-0.00422031	Q8NG11	Tetraspanin-14	954
-0.000331108	-0.000319788	-0.000775312	2.05E-05	-0.000579556	Q8NHP1	Aflatoxin B1 aldehyde reductase member 4	955
-0.000128546	0.000508213	7.28E-05	-0.000733244	0.000935867	Q8TB61-5	Isoform 5 of Adenosine 3-phospho 5-phosphosulfate transporter 1	956

-0.000183414	0.000725139	0.000103903	-0.00104622	0.00133533	H0YNA7	Signal peptide peptidase-like 2A (Fragment)	957
-0.000157075	0.000621006	8.90E-05	-0.000895981	0.00114357	Q8TD08-2	Isoform 2 of Mitogen-activated protein kinase 15	958
-9.65E-06	3.81E-05	5.47E-06	-5.50E-05	7.02E-05	Q8TDN6	Ribosome biogenesis protein BRX1 homolog	959
9.73E-05	-4.15E-05	0.000262674	-0.000237265	1.40E-05	Q8WTV0-4	Isoform 4 of Scavenger receptor class B member 1	960
0.00124216	-0.00279543	0.0038201	0.0036413	0.00614668	Q8WUA8	Tsukushin	961
0.000194654	-0.00525422	0.00530046	-0.00826119	-0.00340628	Q8WUJ3	Cell migration-inducing and hyaluronan-binding protein	962
0.000138972	-0.00169966	0.00197733	0.00634084	-0.00109786	Q8WUM4	Programmed cell death 6-interacting protein	963
0.00121112	0.000692799	0.00103331	0.00148869	-0.00068045	Q8WWI5-3	Isoform 3 of Choline transporter-like protein 1	964
-0.000490749	0.000441281	0.000172843	-0.000631572	-0.00111571	Q8WWZ8	Oncoprotein-induced transcript 3 protein	965
-5.85E-05	0.000231415	3.32E-05	-0.000333883	0.000426147	Q92499-3	Isoform 3 of ATP-dependent RNA helicase DDX1	966
-0.00164901	0.00220848	-0.00142911	0.0016113	-0.00598895	Q92522	Histone H1x	967
-1.90E-05	-9.40E-05	0.000176846	-0.00010766	-0.000118992	C9JIW2	Engulfment and cell motility protein 1 (Fragment)	968
0.0170653	0.00664547	-0.0139157	0.00550512	-0.0272325	Q92626	Peroxidasin homolog	969
0.000205187	-0.000490534	-2.74E-06	0.000892084	-0.00076204	K7EKE8	Nectin-2 (Fragment)	970
0.00148878	0.0151018	-0.00682679	0.0325853	-0.01428	Q92743	Serine protease HTRA1	971
-4.82E-05	0.000190597	2.73E-05	-0.000274992	0.000350982	Q92769-3	Isoform 2 of Histone deacetylase 2	972
-0.000303932	0.00120161	0.000172175	-0.00173367	0.00221275	Q92820	Gamma-glutamyl hydrolase	973
-0.000425512	-0.000715037	0.000667042	-0.000695157	0.00135655	Q92841-1	Isoform 2 of Probable ATP-dependent RNA helicase DDX17	974
-3.28E-05	-0.000133467	0.00050544	2.09E-05	-0.000911143	H3BM42	Golgi apparatus protein 1, isoform CRA_c	975
-3.66E-05	0.000144659	2.07E-05	-0.000208712	0.000266386	Q92900-2	Isoform 2 of Regulator of nonsense transcripts 1	976
-2.94E-05	0.000116195	1.66E-05	-0.000167644	0.00021397	Q92905	COP9 signalosome complex subunit 5	977

-0.000114148	-0.000725683	0.000720724	-0.00018912	-0.000364504	Q93008-1	Isoform 2 of Probable ubiquitin carboxyl-terminal hydrolase FAF-X	978
0.000297986	-0.00250363	0.00117451	0.00296438	-0.00170384	Q93063	Exostosin-2	979
-0.000163465	0.000646266	9.26E-05	-0.000932425	0.00119009	Q93088	Betaine--homocysteine S-methyltransferase 1	980
-0.000550233	-0.000998966	-0.00105028	-0.00065746	-0.00166185	Q93099	Homogentisate 1,2-dioxygenase	981
-6.99E-05	0.00027641	3.96E-05	-0.000398801	0.000509004	Q969E2-2	Isoform 2 of Secretory carrier-associated membrane protein 4	982
0.0181308	0.00595418	-0.00666751	0.0441678	0.00672805	Q969P0	Immunoglobulin superfamily member 8	983
-3.81E-05	-3.68E-05	-8.92E-05	2.36E-06	-6.67E-05	A0A0U1RRM8	Fermitin family homolog 2 (Fragment)	984
-7.37E-05	0.000291524	4.18E-05	-0.000420607	0.000536836	Q96BI3-3	Isoform 3 of Gamma-secretase subunit APH-1A	985
-5.88E-05	0.000232317	3.33E-05	-0.000335184	0.000427808	Q9NVJ2	ADP-ribosylation factor-like protein 8B	986
-0.00166192	0.00657049	0.000941466	-0.00947983	0.0120995	Q96C12-3	Isoform 3 of Armadillo repeat-containing protein 5	987
-0.000289487	-0.000148887	0.000129913	4.11E-05	-0.000840332	Q96CW1-2	Isoform 2 of AP-2 complex subunit mu	988
4.32E-05	-0.000654986	-0.000284639	-0.000780538	0.00216961	Q96DD7	Protein shisa-4	989
6.08E-05	0.000400675	0.000138421	0.00106799	-0.00209585	Q96DG6	Carboxymethylenebutenolidase homolog	990
0.000337042	4.30E-05	0.00100923	3.95E-05	0.000575443	G3V5E4	Glucosamine 6-phosphate N-acetyltransferase	991
0.000291988	-0.000566389	0.00104479	0.00151068	0.00103802	F5GYN4	Ubiquitin thioesterase OTUB1	992
-2.51E-05	-0.000560703	0.000177645	-0.000313565	-0.00318027	Q96G03	Phosphoglucomutase-2	993
0.000902543	-0.000497384	-0.00055835	0.000775936	0.00396777	Q96HF1	Secreted frizzled-related protein 2	994
-0.000117013	0.000196268	-5.02E-05	0.000473111	0.000269224	Q9Y383-3	Isoform 3 of Putative RNA-binding protein Luc7-like 2	995
-0.000101881	0.00040279	5.77E-05	-0.000581141	0.000741731	C9J255	Mannose-1-phosphate guanyltransferase alpha (Fragment)	996
0.00286538	0.000715126	0.00420523	0.0146634	-0.00148609	Q96KG7	Multiple epidermal growth factor-like domains protein 10	997
0.000618389	-0.00235126	0.00369339	0.00152216	-0.00231331	Q96KP4-2	Isoform 2 of Cytosolic non-specific dipeptidase	998

-2.09E-05	8.28E-05	1.19E-05	-0.00011941	0.000152407	Q96ME1-2	Isoform 2 of F-box/LRR-repeat protein 18	999
-0.000279896	0.00110658	0.000158559	-0.00159657	0.00203775	H0Y896	Cilia- and flagella-associated protein 44 (Fragment)	1000
-0.00017448	0.000689817	9.88E-05	-0.00099526	0.00127029	Q96PD5	N-acetylmuramoyl-L-alanine amidase	1001
-0.000205186	0.000811215	0.000116237	-0.00117041	0.00149384	F8VUY8	Sodium-coupled neutral amino acid transporter 2	1002
-0.000729328	-0.000356056	0.00196464	-0.00249131	-0.000227636	Q96QK1	Vacuolar protein sorting-associated protein 35	1003
-0.000238985	-0.000428909	-0.000416716	0.00138018	0.00151453	Q96TA1-2	Isoform 2 of Niban-like protein 1	1004
-9.79E-05	0.00101909	0.000225914	0.000153065	-0.000917997	K7ES69	Calponin-2	1005
-0.00092363	-1.67E-05	-0.00108352	-0.00615256	0.00345016	Q99460-2	Isoform 2 of 26S proteasome non-ATPase regulatory subunit 1	1006
-0.00111781	-0.000442856	0.00224896	0.00136738	0.000229858	Q99536	Synaptic vesicle membrane protein VAT-1 homolog	1007
-5.11E-05	-0.00025248	0.000474934	-0.00028913	-0.000319563	Q99627-2	Isoform 2 of COP9 signalosome complex subunit 8	1008
0.00962338	0.0103427	-0.0242397	-0.0346937	0.0734258	D6RGG3	Collagen alpha-1(XII) chain	1009
-0.000125824	-8.00E-05	0.000353038	-0.00061052	-0.000488411	Q99805	Transmembrane 9 superfamily member 2	1010
-2.78E-05	0.000110077	1.58E-05	-0.000158818	0.000202705	Q99808	Equilibrative nucleoside transporter 1	1011
0.000213037	0.00161616	-0.00371766	0.00105109	0.00755173	Q99832-3	Isoform 3 of T-complex protein 1 subunit eta	1012
-0.000550865	-0.0020178	2.57E-05	-0.00222093	0.00407718	E9PKG1	Protein arginine N-methyltransferase 1	1013
-2.39E-05	-0.000118137	0.000222225	-0.000135286	-0.000149526	Q99985	Semaphorin-3C	1014
2.55E-05	0.000168179	5.81E-05	0.000448279	-0.000879712	Q99988	Growth/differentiation factor 15	1015
-4.29E-05	0.000169585	2.43E-05	-0.000244675	0.000312288	H0Y711	Methylosome protein 50 (Fragment)	1016
-0.000273345	0.00217239	0.000449265	-0.000330391	-0.000826441	Q9BQE3	Tubulin alpha-1C chain	1017
-0.000359415	4.67E-06	0.000322318	-0.00184398	-0.00263708	I3L1L3	Myb-binding protein 1A (Fragment)	1018
0.000888311	-0.00025263	-0.00101288	0.00235306	0.00125782	A0A087WW53	Coronin	1019
0.00118457	-0.000333921	0.0016504	0.00347817	-0.00301746	B4DKD1	Protein tweety homolog	1020

**-3.65E-05**	**0.000144312**	**2.07E-05**	**-0.000208211**	**0.000265747**	**Q9BSJ8**	**Extended synaptotagmin-1**	**1021**
**0**	**0**	**0**	**0**	**0**	**D6RBY3**	**Spondin-2 (Fragment)**	**1022**
**0.000244948**	**-0.000355659**	**0.000299521**	**-0.00073786**	**0.00124233**	**Q9BUF5**	**Tubulin beta-6 chain**	**1023**
**-0.000458209**	**0.00012768**	**-0.000738685**	**-0.000285309**	**0.00128376**	**Q9BWD1**	**Acetyl-CoA acetyltransferase, cytosolic**	**1024**
**-0.000292752**	**0.00227845**	**-0.000215718**	**-0.0002914**	**-0.00224616**	**Q9BXJ4**	**Complement C1q tumor necrosis factor-related protein 3**	**1025**
**-0.000220135**	**-7.45E-05**	**0.00105181**	**-0.0012508**	**0.000165015**	**Q9BXJ9**	**N-alpha-acetyltransferase 15, NatA auxiliary subunit**	**1026**
**-0.000269602**	**0.00120056**	**-0.000847883**	**-0.00148371**	**-0.00053628**	**Q9BXN1**	**Asporin**	**1027**
**-5.26E-05**	**0.000208126**	**2.98E-05**	**-0.000300282**	**0.000383261**	**K7EPR4**	**AP-1 complex subunit mu-2 (Fragment)**	**1028**
**-7.87E-05**	**-0.000514253**	**1.15E-05**	**-0.000852529**	**0.0018852**	**Q9BZQ8**	**Protein Niban**	**1029**
**0.0031064**	**0.00290551**	**0.000245975**	**0.00914652**	**-0.00983187**	**Q9C0H2-2**	**Isoform 2 of Protein tweety homolog 3**	**1030**
**-2.94E-05**	**-0.000552326**	**-0.000407085**	**0.000680569**	**0.00208774**	**Q9H0U4**	**Ras-related protein Rab-1B**	**1031**
**-0.00012306**	**0.000166853**	**0.000129621**	**-0.000380238**	**0.000197917**	**B4DGG1**	**Protein FAM234A**	**1032**
**0.00107**	**0.000562173**	**-2.44E-05**	**0.00331776**	**0.00345934**	**Q9H1J7**	**Protein Wnt-5b**	**1033**
**-0.000222816**	**0.000880914**	**0.000126224**	**-0.00127097**	**0.00162219**	**Q9H223**	**EH domain-containing protein 4**	**1034**
**-9.42E-05**	**0.000372271**	**5.33E-05**	**-0.000537108**	**0.00068553**	**Q9H2A2-2**	**Isoform 2 of 2-aminomuconic semialdehyde dehydrogenase**	**1035**
**-0.000629987**	**0.00249069**	**0.000356883**	**-0.00359353**	**0.00458656**	**Q9H2D6-6**	**Isoform 6 of TRIO and F-actin-binding protein**	**1036**
**-0.000230219**	**0.000910185**	**0.000130418**	**-0.0013132**	**0.00167609**	**Q9H2M3-2**	**Isoform 2 of S-methylmethionine--homocysteine S-methyltransferase BHMT2**	**1037**
**-5.15E-05**	**0.000203699**	**2.92E-05**	**-0.000293894**	**0.000375108**	**Q9H3Z4-2**	**Isoform 2 of DnaJ homolog subfamily C member 5**	**1038**
**0.000319003**	**8.34E-05**	**0.000874935**	**0.000297909**	**0.00171254**	**Q9H4A4**	**Aminopeptidase B**	**1039**
**-0.101362**	**-0.0115343**	**-0.0661876**	**-0.147476**	**-0.0642477**	**Q9H4B7**	**Tubulin beta-1 chain**	**1040**
**-0.00766958**	**-0.00370353**	**0.000292469**	**0.00902918**	**0.0166151**	**Q9H4M9**	**EH domain-containing protein 1**	**1041**
**-0.000467453**	**3.61E-06**	**-2.37E-05**	**-0.00109953**	**0.000177397**	**Q5VXN0**	**Ribosome production factor 2 homolog (Fragment)**	**1042**
**0**	**0**	**0**	**0**	**0**	**I3L4C5**	**Ubiquitin-conjugating enzyme E2 Z (Fragment)**	**1043**

4.47E-05	0.00018535	0.00017512	0.00152766	0.0053104	B8ZZF5	Protein eva-1 homolog A	1044
-6.78E-05	0.000268013	3.84E-05	-0.000386686	0.000493542	Q9H8Y8-2	Isoform 2 of Golgi reassembly-stacking protein 2	1045
-0.000346145	0.000475758	-0.000458069	-2.45E-05	-0.000855265	Q9HAV0	Guanine nucleotide-binding protein subunit beta-4	1046
-0.00297402	-0.00114537	-0.00124612	-0.00523778	0.00112338	Q9HBI1-3	Isoform 3 of Beta-parvin	1047
-2.73E-05	-0.000134739	0.000253455	-0.000154298	-0.000170539	I3L1I0	Glyoxalase domain-containing protein 4 (Fragment)	1048
-3.38E-05	-0.000167221	0.000314554	-0.000191494	-0.00021165	Q9HD45	Transmembrane 9 superfamily member 3	1049
0.00094211	0.000780833	-0.000616288	-0.00115851	0.00831448	Q9HDC9	Adipocyte plasma membrane-associated protein	1050
-0.000125186	0.000494931	7.09E-05	-0.000714081	0.000911408	A0A087X163	Ras-related protein Rab-18	1051
-0.0110389	-0.00673974	0.00328853	-0.0237258	-0.00753596	F8W914	Reticulon	1052
1.31E-05	-0.00220523	0.00210879	-0.0015722	-0.00135351	Q5T6H7	Xaa-Pro aminopeptidase 1	1053
-0.000347838	-0.00171866	0.00323293	-0.00196814	-0.00217531	Q9NQX4	Unconventional myosin-Vc	1054
-3.99E-05	0.000157885	2.26E-05	-0.000227795	0.000290743	F5H4I5	Integral membrane protein 2C (Fragment)	1055
-2.77E-05	-0.000358204	0.000132311	-0.000766659	0.00131045	Q9NR30-2	Isoform 2 of Nucleolar RNA helicase 2	1056
-0.000131901	-0.000289931	0.000167426	0.000980444	0.00111411	Q9NR31	GTP-binding protein SAR1a	1057
-0.00077124	-0.000896132	0.000686307	-0.00179102	-0.00127928	Q9NR45	Sialic acid synthase	1058
0.000225875	-0.000591361	0.000643222	-0.00121621	-0.000859033	H0Y9N2	Platelet-derived growth factor C (Fragment)	1059
0.00178327	-0.000591106	-0.00100104	0.00340516	0.00268372	B4DNG0	cDNA FLJ58142, highly similar to Olfactomedin-like protein 3	1060
0	0	0	0	0	J3KN20	CREB/ATF bZIP transcription factor (Fragment)	1061
-0.000291192	-0.000591904	0.00117686	-0.000507483	-0.0014535	Q9NSD9	Phenylalanine--tRNA ligase beta subunit	1062
0.0030065	0.00113567	-0.00160236	0.00138833	0.00157926	Q9NT68-2	Isoform 2 of Teneurin-2	1063
-0.000263165	0.00104044	0.000149081	-0.00150113	0.00191595	Q9NTK1	Protein DEPP1	1064
-0.000205245	-0.000993578	2.03E-05	0.000488431	-0.000504065	Q9NTK5	Obg-like ATPase 1	1065
-0.000524908	-0.000480546	-0.000467844	-0.000840268	-0.000247849	D6RGI3	Septin 11, isoform CRA_b	1066
0.000503027	-2.14E-05	-0.000263075	0.000544234	0.000309432	Q9NVM1	Protein eva-1 homolog B	1067

**-1.27E-05**	**-1.23E-05**	**-2.98E-05**	**7.86E-07**	**-2.22E-05**	**Q9NY15**	**Stabilin-1**	**1068**
**4.22E-05**	**-7.38E-05**	**2.76E-05**	**0.000568153**	**0.00114196**	**D6RD48**	**Claudin domain-containing protein 1 (Fragment)**	**1069**
**0.000196085**	**-0.0003317**	**0.000235974**	**-0.000115133**	**0.000637143**	**H0YD14**	**Myoferlin (Fragment)**	**1070**
**-0.0019829**	**-0.000960398**	**-0.00246506**	**0.00449619**	**-0.00308229**	**Q9NZN3**	**EH domain-containing protein 3**	**1071**
**-0.000107247**	**-0.000133204**	**0.000630233**	**6.47E-05**	**-3.40E-05**	**Q9NZN4**	**EH domain-containing protein 2**	**1072**
**-0.000185952**	**-0.000383463**	**0.000160227**	**-0.00118102**	**0.00101982**	**Q9P0L0**	**Vesicle-associated membrane protein-associated protein A**	**1073**
**-0.000465467**	**-0.000484778**	**-0.000117946**	**-0.00186573**	**0.00245346**	**Q9P126-2**	**Isoform 2 of C-type lectin domain family 1 member B**	**1074**
**-5.76E-05**	**-0.000166001**	**0.000339802**	**-8.02E-05**	**0.000401716**	**Q9P258**	**Protein RCC2**	**1075**
**0.00691758**	**-0.0017563**	**0.00386661**	**0.00543877**	**0.000649541**	**Q9P273**	**Teneurin-3**	**1076**
**0.18146**	**0.0505546**	**-0.0208616**	**0.400822**	**-0.0518913**	**Q9P2B2**	**Prostaglandin F2 receptor negative regulator**	**1077**
**1.67E-05**	**-2.91E-05**	**1.09E-05**	**0.000224291**	**0.000450816**	**H7C5L5**	**Hexosyltransferase (Fragment)**	**1078**
**4.75E-05**	**0.000561495**	**0.00130137**	**-0.00125516**	**-0.000945981**	**Q9P2J5**	**Leucine--tRNA ligase, cytoplasmic**	**1079**
**-0.000372227**	**0.000195394**	**-0.00054357**	**-0.00062736**	**0.000367124**	**Q9P2R3**	**Rabankyrin-5**	**1080**
**-0.000101814**	**0.000402526**	**5.77E-05**	**-0.00058076**	**0.000741246**	**Q9UBG0**	**C-type mannose receptor 2**	**1081**
**-8.03E-05**	**0.000317449**	**4.55E-05**	**-0.000458012**	**0.000584578**	**Q9UBI6**	**Guanine nucleotide-binding protein G(I)/G(S)/G(O) subunit gamma-12**	**1082**
**-9.96E-05**	**0.000393644**	**5.64E-05**	**-0.000567945**	**0.000724889**	**Q9UBQ0**	**Vacuolar protein sorting-associated protein 29**	**1083**
**-0.000164508**	**-0.000457269**	**0.000489683**	**0.000983322**	**-0.000425517**	**K7ES31**	**Eukaryotic translation initiation factor 3 subunit K**	**1084**
**-5.27E-05**	**0.000208473**	**2.99E-05**	**-0.000300783**	**0.0003839**	**Q9UBQ7**	**Glyoxylate reductase/hydroxypyruvate reductase**	**1085**
**-4.56E-05**	**0.000180466**	**2.59E-05**	**-0.000260374**	**0.000332325**	**H0YD65**	**Cathepsin F (Fragment)**	**1086**
**-0.00582127**	**0.000667262**	**-0.0037414**	**-0.00737025**	**-0.00804193**	**Q9UGM3-9**	**Isoform 9 of Deleted in malignant brain tumors 1 protein**	**1087**
**-0.00020166**	**-0.000406386**	**0.000849735**	**0.00061764**	**0.000710875**	**K7EK18**	**Septin-9 (Fragment)**	**1088**
**-9.31E-05**	**0.000367913**	**5.27E-05**	**-0.00053082**	**0.000677505**	**C9JGT6**	**Prenylcysteine oxidase 1 (Fragment)**	**1089**
**-4.31E-05**	**-0.000195676**	**9.00E-05**	**-0.000723862**	**0.00103496**	**Q9UHN6-2**	**Isoform 2 of Cell surface hyaluronidase**	**1090**

**-6.57E-05**	**0.000259644**	**3.72E-05**	**-0.000374612**	**0.000478131**	**M0QXH1**	**PRA1 family protein (Fragment)**	**1091**
**-3.36E-05**	**0.00013302**	**1.91E-05**	**-0.000191919**	**0.000244954**	**Q9UIA9**	**Exportin-7**	**1092**
**-5.41E-05**	**0.000213692**	**3.06E-05**	**-0.000308312**	**0.00039351**	**Q9UIW2**	**Plexin-A1**	**1093**
**7.59E-05**	**-0.000196522**	**0.000244338**	**0.000250522**	**0.000100316**	**H7C286**	**N-acetyl-D-glucosamine kinase**	**1094**
**-1.29E-05**	**-6.37E-05**	**0.000119876**	**-7.30E-05**	**-8.07E-05**	**Q9UJX2-3**	**Isoform 3 of Cell division cycle protein 23 homolog**	**1095**
**0.000584062**	**0.000688559**	**-0.000217674**	**0.00168658**	**-0.000552457**	**Q9UKK3**	**Protein mono-ADP-ribosyltransferase PARP4**	**1096**
**0.00286689**	**-0.000856807**	**-0.000479069**	**0.00760995**	**0.00984304**	**Q9UKU9**	**Angiopoietin-related protein 2**	**1097**
**0.000791301**	**-0.000809363**	**0.000139461**	**-0.000513553**	**0.00188611**	**Q9UKX5**	**Integrin alpha-11**	**1098**
**-8.75E-05**	**-0.000173237**	**9.76E-05**	**0.00013253**	**-7.85E-05**	**C9JYX9**	**Procollagen C-endopeptidase enhancer 2**	**1099**
**0**	**0**	**0**	**0**	**0**	**G5E9X1**	**Integrator complex subunit 6, isoform CRA_c**	**1100**
**-0.00024338**	**-0.000269995**	**-0.000283596**	**-0.00170865**	**-0.000327107**	**E7EMB6**	**Aspartyl aminopeptidase**	**1101**
**0.000211103**	**-0.00016091**	**6.13E-06**	**0.000504213**	**-0.000886963**	**Q9ULF5**	**Zinc transporter ZIP10**	**1102**
**6.74E-05**	**-0.00102777**	**0.00171588**	**0.000995272**	**-0.00431784**	**Q9ULI3**	**Protein HEG homolog 1**	**1103**
**-0.000678768**	**-0.00227484**	**0.00248618**	**0.000291912**	**-0.000777927**	**Q9ULV4**	**Coronin-1C**	**1104**
**0.000989099**	**-0.000399891**	**0.00125191**	**-8.50E-05**	**-0.000223764**	**Q9UMS4**	**Pre-mRNA-processing factor 19**	**1105**
**-0.000106743**	**0.000422012**	**6.05E-05**	**-0.000608874**	**0.000777129**	**G3V5B5**	**Protein NDRG2 (Fragment)**	**1106**
**-6.05E-05**	**-5.84E-05**	**-0.000141642**	**3.74E-06**	**-0.000105879**	**Q9UN37**	**Vacuolar protein sorting-associated protein 4A**	**1107**
**-0.000373411**	**-0.00031732**	**0.00121208**	**0.000596101**	**-0.000402983**	**Q9UNH7-2**	**Isoform 2 of Sorting nexin-6**	**1108**
**-5.36E-05**	**0.000211776**	**3.03E-05**	**-0.000305549**	**0.000389983**	**Q9UNK4**	**Group IID secretory phospholipase A2**	**1109**
**-0.000497298**	**0.00129593**	**0.00042865**	**-0.00180311**	**-0.000943575**	**Q9UNM6**	**26S proteasome non-ATPase regulatory subunit 13**	**1110**
**-2.07E-05**	**-0.000102466**	**0.000192746**	**-0.00011734**	**-0.000129691**	**A0A0U1RQQ4**	**Endothelial protein C receptor (Fragment)**	**1111**
**-0.000823238**	**0.00325471**	**0.000466358**	**-0.00469586**	**0.0059935**	**E9PKY5**	**Peptidyl-prolyl cis-trans isomerase (Fragment)**	**1112**
**0**	**0**	**0**	**0**	**0**	**H0Y390**	**Microtubule-actin cross-linking factor 1, isoforms 1/2/3/5 (Fragment)**	**1113**
**-7.57E-05**	**-0.000163177**	**0.00026728**	**-0.000601458**	**0.000516995**	**Q9UPY5**	**Cystine/glutamate transporter**	**1114**

-6.48E-05	-0.000320385	0.000602667	-0.000366891	-0.00040551	C9JB30	Microtubule-associated protein RP/EB family member 3 (Fragment)	1115
-1.54E-05	6.09E-05	8.73E-06	-8.79E-05	0.00011221	I3L182	Serine/arginine repetitive matrix protein 2 (Fragment)	1116
-0.000432742	-0.000984161	0.000150852	0.000696671	-0.00344543	Q9UQ80-2	Isoform 2 of Proliferation-associated protein 2G4	1117
-0.000149281	-0.000289195	8.96E-05	-0.00065006	0.00155803	Q9Y223-5	Isoform 5 of Bifunctional UDP-N-acetylglucosamine 2-epimerase/N-acetylmannosamine kinase	1118
-0.00217233	0.00176118	-0.000439185	-0.00554348	0.00380128	Q9Y230	RuvB-like 2	1119
-0.000316444	-0.000497327	0.000350423	-0.000568165	-0.000594091	Q9Y240	C-type lectin domain family 11 member A	1120
-0.000734209	-0.00150371	0.00294293	-0.000255326	-0.00368465	Q9Y262	Eukaryotic translation initiation factor 3 subunit L	1121
-0.00184241	0.000361648	0.000740273	-0.0054788	0.000876409	Q9Y265	RuvB-like 1	1122
-0.000800744	-0.00142304	0.00227282	-0.00095782	-0.00100026	Q9Y285	Phenylalanine--tRNA ligase alpha subunit	1123
-4.02E-05	0.000158787	2.28E-05	-0.000229096	0.000292404	Q9Y289	Sodium-dependent multivitamin transporter	1124
7.89E-05	-0.000429821	0.000583316	-0.000750944	-0.000597087	Q9Y2A7	Nck-associated protein 1	1125
-8.33E-05	0.000329399	4.72E-05	-0.000475253	0.000606583	Q9Y2U5	Mitogen-activated protein kinase kinase kinase 2	1126
-6.08E-05	0.0002402	3.44E-05	-0.000346558	0.000442325	Q9Y2V2	Calcium-regulated heat-stable protein 1	1127
-2.75E-05	0.000108681	1.56E-05	-0.000156803	0.000200134	Q9Y2X3	Nucleolar protein 58	1128
-0.00236654	-0.000561347	-0.00217631	-0.00380524	-0.000736579	Q9Y315	Deoxyribose-phosphate aldolase	1129
-0.000340147	0.000113432	0.00140097	-0.00193389	0.00060274	Q9Y316-2	Isoform 2 of Protein MEMO1	1130
-3.02E-05	-0.000149311	0.000280865	-0.000170985	-0.000188982	A0A087X020	Ribosome maturation protein SBDS	1131
-0.000131851	-0.00024201	0.0012426	-0.00268825	0.00104725	Q9Y3F4	Serine-threonine kinase receptor-associated protein	1132
-0.000733442	-0.00150809	0.00264634	-0.00100931	-0.00279954	Q9Y3I0	tRNA-splicing ligase RtcB homolog	1133
-0.00101803	0.000367775	0.00111115	-0.0026916	-0.00290158	Q9Y3U8	60S ribosomal protein L36	1134
-0.0249736	-0.0119748	0.0201016	-0.0250317	-0.0955227	Q9Y490	Talin-1	1135
0.00106739	0.00636896	-8.67E-05	-0.00459802	0.00226432	Q9Y4K0	Lysyl oxidase homolog 2	1136
0.00186094	-0.00120739	0.00266119	0.0035372	-0.000615272	A0A087X054	Hypoxia up-regulated protein 1	1137

**3.57E-06**	**-7.57E-05**	**9.78E-05**	**5.50E-05**	**0.0015958**	**Q9Y5P6**	**Mannose-1-phosphate guanyltransferase beta**	**1138**
**0.00200477**	**-0.0132153**	**0.0137652**	**0.00166489**	**-0.013393**	**Q9Y5X9**	**Endothelial lipase**	**1139**
**-0.000375816**	**0.000187956**	**-0.000554323**	**-0.000622482**	**0.00035355**	**Q9Y613**	**FH1/FH2 domain-containing protein 1**	**1140**
**-0.000498**	**0.00209177**	**0.0045431**	**0.00294577**	**0.00578498**	**Q9Y617**	**Phosphoserine aminotransferase**	**1141**
**-5.03E-05**	**-4.86E-05**	**-0.000117724**	**3.11E-06**	**-8.80E-05**	**H3BTD2**	**Adhesion G-protein-coupled receptor G1 (Fragment)**	**1142**
**-0.000890876**	**0.000341788**	**-0.00138228**	**-0.00207381**	**-0.00405655**	**Q9Y678**	**Coatomer subunit gamma-1**	**1143**
**0.000151874**	**-0.000601268**	**0.000545232**	**0.00024376**	**-0.000444227**	**Q9Y696**	**Chloride intracellular channel protein 4**	**1144**
**0.108937**	**0.0225149**	**-0.0362577**	**0.234923**	**-0.0279827**	**Q9Y6C2**	**EMILIN-1**	**1145**
**-0.000362758**	**2.99E-05**	**0.00113444**	**-0.000440853**	**-0.00306703**	**Q9Y6G9**	**Cytoplasmic dynein 1 light intermediate chain 1**	**1146**
**0.00290663**	**0.0152315**	**-0.00144001**	**-0.0301263**	**0.014923**	**A0A0A0MTC7**	**Laminin subunit alpha-4**	**1148**
**-7.78E-05**	**0.000307776**	**4.41E-05**	**-0.000444056**	**0.000566764**	**A0A1W2PNV4**	**Uncharacterized protein**	**1149**
**-7.35E-05**	**-0.000245261**	**0.00151057**	**0.000435559**	**-0.00337177**	**A0A1W2PP70**	**HLA class II histocompatibility antigen, DQ alpha 1 chain**	**1150**
**-0.000270056**	**0.00106768**	**0.000152985**	**-0.00154044**	**0.00196612**	**A0A2R8YDT5**	**ATP-dependent RNA helicase DDX3X**	**1152**
**-0.000107359**	**0.000400607**	**0.00237879**	**0.00502226**	**-0.0100031**	**A0A3B3IRI8**	**Putative aldo-keto reductase family 1 member C8**	**1153**
**-0.000100227**	**0.000396253**	**5.68E-05**	**-0.00057171**	**0.000729694**	**A0A3B3IUA2**	**NHP2-like protein 1**	**1154**
**-5.59E-05**	**0.000221089**	**3.17E-05**	**-0.000318985**	**0.000407132**	**E7EUI8**	**Glucose-6-phosphate 1-dehydrogenase (Fragment)**	**1155**
**-0.000743515**	**0.00293953**	**0.000421196**	**-0.00424111**	**0.00541309**	**F6VZ39**	**RNA-binding protein 38**	**1156**
**-0.00342333**	**0.0135343**	**0.00193929**	**-0.0195272**	**0.0249232**	**G3V1N2**	**HCG1745306, isoform CRA_a**	**1157**
**-0.000342589**	**0.00135444**	**0.000194074**	**-0.00195418**	**0.00249419**	**H0Y4K8**	**Fibronectin (Fragment)**	**1158**
**-0.00217453**	**0.00127354**	**-0.000206548**	**-0.00225893**	**0.00540649**	**H3BN98**	**Uncharacterized protein (Fragment)**	**1159**
**-1.62E-05**	**-8.01E-05**	**0.00015069**	**-9.17E-05**	**-0.000101393**	**K7EM38**	**Actin, cytoplasmic 2 (Fragment)**	**1160**
**-0.00888205**	**-0.00102438**	**-0.00521952**	**-0.00551292**	**-0.0197253**	**Q5TEC6**	**Histone H3**	**1161**

Proteins that we identified in EVs that have not been reported on Vesiclepedia database [as of 12th July 2022]

**Gene name**	**Protein encoded**
ATP5F1B	ATP synthase subunit beta, mitochondrial
ATP5F1A	Isoform 2 of ATP synthase subunit alpha, mitochondrial
RACK1	Receptor of activated protein C kinase 1
SEPTIN2	Septin-2
ELOC	Elongin-C
SEPTIN7	Septin-7
P3H1	Isoform 4 of Prolyl 3-hydroxylase 1
PYCR3	Isoform 2 of Pyrroline-5-carboxylate reductase 3
CEMIP	Cell migration-inducing and hyaluronan-binding protein
NECTIN2	Nectin-2
FAM234A	Protein FAM234A
TENM2	Isoform 2 of Teneurin-2
SEPTIN11	Septin 11, isoform CRA_b
EVA1B	Protein eva-1 homolog B
TENM3	Teneurin-3
SEPTIN9	Septin-9
CEMIP2	Isoform 2 of Cell surface hyaluronidase
RTCB	tRNA-splicing ligase RtcB homolog
HIST2H3PS2	Histone H3
